# Transcriptome Analysis of Differentially Expressed Genes Related to the Growth and Development of the Jinghai Yellow Chicken

**DOI:** 10.3390/genes10070539

**Published:** 2019-07-17

**Authors:** Fuxiang Chen, Pengfei Wu, Manman Shen, Mingliang He, Lan Chen, Cong Qiu, Huiqiang Shi, Tao Zhang, Jiahong Wang, Kaizhou Xie, Guojun Dai, Jinyu Wang, Genxi Zhang

**Affiliations:** 1 College of Animal Science and Technology, Yangzhou University, Yangzhou 225009, China; 2 Jiangsu Jinghai Poultry Group Co., Ltd., Nantong 226100, China; 3 Upper School, Rutgers Preparatory School, NJ 08873, USA

**Keywords:** chicken, growth, animal product, transcriptome, upregulated, downregulated, real-time PCR

## Abstract

The growth traits are important traits in chickens. Compared to white feather broiler breeds, Chinese local broiler breeds have a slow growth rate. The main genes affecting the growth traits of local chickens in China are still unclear and need to be further explored. This experiment used fast-growth and slow-growth groups of the Jinghai Yellow chicken as the research objects. Three males and three females with similar body weights were selected from the two groups at four weeks old and eight weeks old, respectively, with a total of 24 individuals selected. After slaughter, their chest muscles were taken for transcriptome sequencing. In the differentially expressed genes screening, all of the genes obtained were screened by fold change ≥ 2 and false discovery rate (FDR) < 0.05. For four-week-old chickens, a total of 172 differentially expressed genes were screened in males, where there were 68 upregulated genes and 104 downregulated genes in the fast-growth group when compared with the slow-growth group. A total of 31 differentially expressed genes were screened in females, where there were 11 upregulated genes and 20 downregulated genes in the fast-growth group when compared with the slow-growth group. For eight-week-old chickens, a total of 37 differentially expressed genes were screened in males. The fast-growth group had 28 upregulated genes and 9 downregulated genes when compared with the slow-growth group. A total of 44 differentially expressed genes were screened in females. The fast-growth group had 13 upregulated genes and 31 downregulated genes when compared with the slow-growth group. Through gene ontology (GO) enrichment analysis and Kyoto Encyclopedia of Genes and Genomes (KEGG) analysis, many genes were found to be related to cell proliferation and differentiation, muscle growth, and cell division such as *SNCG*, *MCL1*, *ARNTL*, *PLPPR4*, *VAMP1*, etc. Real-time PCR results were consistent with the RNA-Seq data and validated the findings. The results of this study will help to understand the regulation mechanism of the growth and development of Jinghai Yellow chicken and provide a theoretical basis for improving the growth rate of Chinese local chicken breeds.

## 1. Introduction

Muscle is an important part of an animal’s body, and skeletal muscle alone accounts for about 40% of the bodyweight [1]. Skeletal muscle plays a crucial role in regulating animal metabolism, exercise capacity, and health [2]. A decrease in muscle mass will result in decreased exercise capacity and increased morbidity and mortality [3,4]. In livestock production, the growth and development of skeletal muscle is closely related to the yield of livestock and poultry. Wang et al. [5] stated that studying the growth and regulation mechanism of the skeletal muscle tissue of poultry will help understand the basic trend and development law of protein deposition in the body and provide a theoretical basis for improving the production performance and feed conversion rate of animal organisms. Ouyang et al. [6] found that 491 differentially expressed proteins were identified in three different broiler developmental groups. These differentially expressed proteins were mainly involved in the ribosome, muscle contraction, and oxidative phosphorylation pathways. In the NCBI database, chickens have the most abundant genetic sequence information compared to ducks, geese, and pigeons, and have the most reference data for later molecular studies. Therefore, the research progress of chickens is faster than that of other poultry. The lack of gene sequencing information will greatly delay the study of the molecular level of the species. Therefore, there is great research value and research significance in the genetic information on chicken. 

Jinghai Yellow chicken is a national yellow feather broiler breed. This chicken breed is characterized by high-quality meat, early maturation, high reproductive performance, and its adaptability to poor quality feeds [7]. This chicken breed is also the first female parent of a national chicken breed—Haiyang Yellow chicken. Compared to white feather broilers, Chinese local yellow feather broilers grow slowly [8]. There have been a few studies related to the regulation mechanism of muscle growth, and further investigation into the regulatory genes related to growth traits is needed. Roelfsema.et al. [9] found that growth and metabolism-related genes such as *GH/GHR*, *IGF-I/IGF-IR*, *Calpain 3*, and *MRFs* play important regulatory roles in avian muscle growth. These are important auxiliary marker genes in poultry genetic breeding and have a high reference value [10]. 

Transcriptome sequencing (RNA-Seq) can explore the differences in gene types and expression levels at a global level, quantitatively describe the differences in gene types and expression levels under specific conditions, and intuitively link changes at the gene level with phenotypic changes. Through this research method, the process of livestock genetics and breeding has been greatly improved. In recent years, to further explore the molecular mechanism regulating the important economic traits of livestock and poultry, a large number of livestock and poultry transcriptome studies have been carried out using the RNA-Seq technology [11]. Miriam et al. [12] found that some differentially expressed transcription factors such as *FOXOs, MEF2D, MYOD1,* and other genes were presumed to play an important role in the growth and regulation of intramuscular fat deposition in two pig breeds by using RNA-Seq technology. Yun et al. [13] found that *HTR2A* was a potential regulator of adipocyte differentiation during fat differentiation in yellow cattle by RNA-Seq analysis, which can regulate fat production by phosphorylating the AKT signaling pathway. Wang et al. [14] analyzed the transcriptome changes of *MSTN* knockout goats by RNA-Seq and found that the expression of genes related to knockout genes in goats and fatty acid metabolism changed greatly, indicating that the *MSTN* gene may be involved in fatty acid metabolism. Dong et al. [15] analyzed the transcriptome data of the ovary tissue of Jinghai Yellow chicken using RNA-Seq and found 4431 new transcripts. This provides a data basis for the further improvement of the chicken genome and the mining of functional genes.

Animal and poultry production performance indicators reflect the growth and development of animals, and the economic value of animals with good production performance is higher. Usually, the production performance includes growth performance and slaughter performance [16]. In the experiment, at four and eight weeks of age, the fast-growth and slow-growth groups of the Jinghai Yellow chicken were selected as the research objects. After slaughter, the chest muscles were collected for transcriptome sequencing to find the genes and pathways associated with growth. Next, quantitative real-time PCR (QRT-PCR) was used to quantitatively analyze the differentially expressed genes [17]. The aim of this research is to screen some genes related to growth traits as auxiliary marker genes, provide a theoretical basis for revealing the molecular mechanism of chicken growth, and improve the growth rate of Jinghai Yellow Chicken.

## 2. Materials and Methods 

### 2.1. Ethics Statement

The study protocol was approved by the Animal Care Committee of the Department of Animal Science and Technology of Yangzhou University, Yangzhou, China (permit number SYXK (Su) 2012-0029). It was conducted in accordance with the guidelines of the Animal Use Committee of the Chinese Ministry of Agriculture (Beijing, China). 

### 2.2. Test Animal and Sample Collection

The chicken species used in this experiment was the Jinghai Yellow Chicken from Jiangsu Province, China (Jinghai Poultry Group Co. Ltd., Haimen, China). In our previous research, the molecular marker J band related to the body weight of Jinghai Yellow chicken was detected by DNA fingerprinting. DNA fingerprinting was performed using EAV (endogenous avian retrovirus) fragment as a probe and EcoRI as a restriction enzyme, and a J-band about 3.48 kb in length was found in the DNA fingerprint. The average body weight of the Jinghai Yellow chickens without the J band was significantly higher than those with the J band [18,19]. Based on the molecular marker J and divergent selection, fast-growth and show-growth strains were established as these two strains can meet the requirements of different markets. In this research, they were hatched on the same day and had access to feed and water ad libitum. Full-price granule compound feed was used during feeding process, and the nutrient level was based on the Jiangsu Provincial Standards (DB32/T 1838-2011). The fast-growth and the slow-growth strains were raised separately on the floor in the same chicken house until transferring them to laying cages at 14-week age. At four and eight weeks old, three females with similar body weights and three males with similar body weights were selected from the fast-growth and slow-growth strains of Jinghai Yellow chicken, respectively. A total of 24 chickens were sampled for RNA-Seq. After slaughter, the chest muscles were collected and stored in liquid nitrogen.

### 2.3. Total RNA Extraction

We used the method for RNA extraction referred to in Xue et al. [20] where total RNA from the chest muscles was isolated using the TRIzol total RNA extraction kit (Invitrogen, Carlsbad, CA, USA), in accordance with the manufacturer’s instructions. RNA quality was checked with a 1% agarose gel. RNA concentration and purity were determined using NanoDrop (IMPLEN, Los Angeles, CA, USA). Sample integrity was measured using an Agilent Bioanalyzer 2100 (Agilent Technologies, Santa Clara, CA, USA).

### 2.4. Construction and Detection of Transcriptome Sequencing Library

Eukaryotic mRNA was enriched through the binding of the A–T complementary pairing to the poly-A tail of mRNA using magnetic beads with Oligo (dT). Subsequently, one-strand cDNA and two-strand cDNA were sequentially synthesized under different reaction conditions using mRNA as a template, and then the double-stranded cDNA was purified using AMPure XP beads (BeckMan Coulter, Brea, CA, USA). Purified double-stranded cDNA was subjected to end-repair and A-tail and ligation to the sequencing linker, then AMPure XP beads were used for fragment size selection, and finally, PCR enrichment was performed to obtain the final cDNA library [21]. 

After the library was constructed, preliminary quantification was performed using a Qubit 2.0 fluorescence meter (Thermo, Waltham, MA, USA). The library was diluted to 1 ng/uL, and then the insert length of the library was detected using an Agilent Bioanalyzer 2100. After the insert size was as expected, the effective concentration of the library was accurately quantified using the QRT-PCR (library effective concentration > 2 nM) to guarantee the library quality [22]. After the library passed the quality test, it was sequenced on an Illumina NovaSeq 5000 (Novogene Co. Ltd., Beijing, China) and 150 bp paired-end reads were generated. The raw transcriptome read data are available in the SRA database with accession number PRJNA528681.

### 2.5. Gene Expression Level Analysis

The direct expression of a gene’s expression level is the abundance of its transcript. The higher the transcript abundance, the higher the gene expression level. FPKM is the number of fragments per kilobase length from a gene per million fragments and also considers the effect of sequencing depth and gene length on the count of fragments. It is the most commonly used method for estimating gene expression level [23,24]. 

In this research, the reference genome used is Gallus gallus-5.0. Paired-end clean reads were aligned to the reference genome using TopHat v2.0.12. HTSeq v0.6.1 was used to count the read numbers mapped into each gene and the parameters were -m union. The differentially expressed genes analysis was performed using the DESeq R package (1.18.0) and the criteria for screening differentially expressed genes were fold change ≥ 2 and false discovery rate (FDR) < 0.05.

### 2.6. Differentially Expressed Genes GO Enrichment Analysis

Gene ontology (referred to as GO, http://www.geneontology.org/) is an international standard classification system for gene function. The method used in the GO enrichment analysis in the experiment is GOseq [25], which is based on the Wallenius non-central hyper-geometric distribution. Compared with ordinary hypergeometric distribution, the characteristic of this distribution is that the probability of extracting an individual from a certain category is different from the probability of extracting an individual from outside a certain category. This difference in probability is obtained by estimating the preference for gene length so that the probability that the GO term is enriched by the differential gene can be estimated more accurately, according to the differential gene GO enrichment mapping.

### 2.7. Differentially Expressed Genes KEGG Enrichment Analysis

The Kyoto Encyclopedia of Genes and Genomes (referred to as KEGG, http://www.genome.jp/kegg/) is a systematic analysis of gene function and genomic information databases that helps researchers study genes and expressions as a whole network. The pathway significant enrichment analysis in the experiment used the pathway in the KEGG database, and the hypergeometric test was applied to find the pathway that was significantly enriched in the differentially expressed genes compared to the entire genomic background [26]. The calculation for this is as follows:

(1) $$p=1-\overset{m-1}{\underset{i=0}{\sum }}\frac{(\begin{array}{c} M\\ i \end{array})(\begin{array}{c} N\\ n \end{array}\begin{array}{c} -\\ - \end{array}\begin{array}{c} M\\ i \end{array})}{(\begin{array}{c} N\\ n \end{array})}$$

where *N* is the number of genes with a pathway annotation in all genes; *n* is the number of differentially expressed genes in *N*; *M* is the number of genes annotated as a particular pathway in all genes; and *m* is the number of differentially expressed genes annotated as a particular pathway. When FDR < 0.05, it means that the differential gene is significantly enriched in the pathway. We used KOBAS 2.0 for the pathway enrichment analysis.

### 2.8. Verification of RNA-Seq Results Using QRT-PCR

At four weeks and eight weeks of age, 24 Jinghai Yellow chickens were selected and divided into eight groups according to body weight, gender, and growth period. The chest muscles were collected, and RNA was extracted. The RNA-Seq results were verified by QRT-PCR. The mRNA was reverse transcribed into cDNA using the PrimeScript RT Master Mix (Perfect Real Time) kit (Vazyme Biotechnology Co. Ltd., Nanjing, China). The primers used for quantification in the study were designed using Primer-BLAST on the NCBI website (https://www.ncbi.nlm.nih.gov/tools/primer-blast/). To avoid the effects of genomic DNA, primers needed to be separated by at least one intron in the corresponding gene (Table 1). In the study, *β-actin* was used as the housekeeping gene [20,27].

## 3. Results

### 3.1. Comparison of Body Weight between Fast-growth and Slow-Growth Groups

SPSS 19.0 (IBM, Among, New York, USA) software was used to analyze the difference in body weight between the fast-growth group and the slow-growth group, where the results showed that the body weight of the fast-growth group in both periods was significantly larger than those in the slow-growth group (*p* < 0.01) (Table 2).

### 3.2. Measurement Data Quality Assessment Results

In the sequencing data, M4F, F4F, M4S, and F4S were used to denote four-week-old fast-growth males and females, and slow-growth males and female, respectively. M8F, F8F, M8S, and F8S were used to denote the eight-week-old fast-growth males and females, and slow-growth males and females, respectively. The above symbols are used to represent the corresponding individual in the full text.

After the quality control of sequencing data, the GC content of the 12 samples at the four-week old was 50.82–54.90%, the base percentage of Q20 was above 95.55%, and the percentage of the Q30 base was above 90.24% (Table 3). The GC content of the 12 samples at eight-week-old was 52.85–54.64%, the base percentage of Q20 was above 96.41%, and the base percentage of Q30 was above 91.80% (Table 4). In summary, the sequencing data can be used for the subsequent data analysis.

### 3.3. Transcriptome Data Alignment with Reference Genome Sequences

The valid data was compared to the reference genome and the total reads of the 12 samples at four weeks old were between 73.63% and 85.89% on the reference genome. The percentage of reads aligned to the unique location of the reference genome was 69.49% to 81.94% in the clean reads. The efficiency of the total reads aligned to the reference genome of the 12 samples at eight weeks old was between 75.60% and 79.74% and the percentage of reads aligned to the unique location of the reference genome was between 71.30% and 74.18% in clean reads. The statistics showed that the results were reliable. In theory, reads from mature mRNA should be aligned to the exon region, but the results indicated that this was not the case. In fact, 88.40~93.20% of the sequencing data of the four-week-old samples aligned with the exon region, 0.9~1.9% aligned with the introns, and 5.5~10.6% aligned with the intergenic regions. Approximately 88.90~95.40% of the sequencing data of the eight-week-old samples were compared to the exon region, 1.3~3.3% aligned with the introns, and 3.7~9.4% were aligned to the intergenic regions. In species with more complete genome annotations, the reads mapped to the exon were the highest. The reads mapped to the intergenic regions might have resulted from incomplete genome annotations. Reads aligned to introns were due to mRNA precursors and different alternative splicing. 

By examining the distribution of the insert on the gene, the randomization of the mRNA fragmentation and the degradation of mRNA were evaluated. The results showed that the randomness was good and there was no degradation. The degree of dispersion of the length of the insert was evaluated by the length distribution of the inserted segments. The mapped data of each sample was used to simulate the saturation of the number of genes detected for different expressions. Next, a graph was drawn that showed that as the amount of sequencing data increased, the number of genes detected for different expression levels eventually became saturated, indicating the validity and reliability of the results of this study.

### 3.4. Differentially Expressed Genes Screening

**Figure 1:**
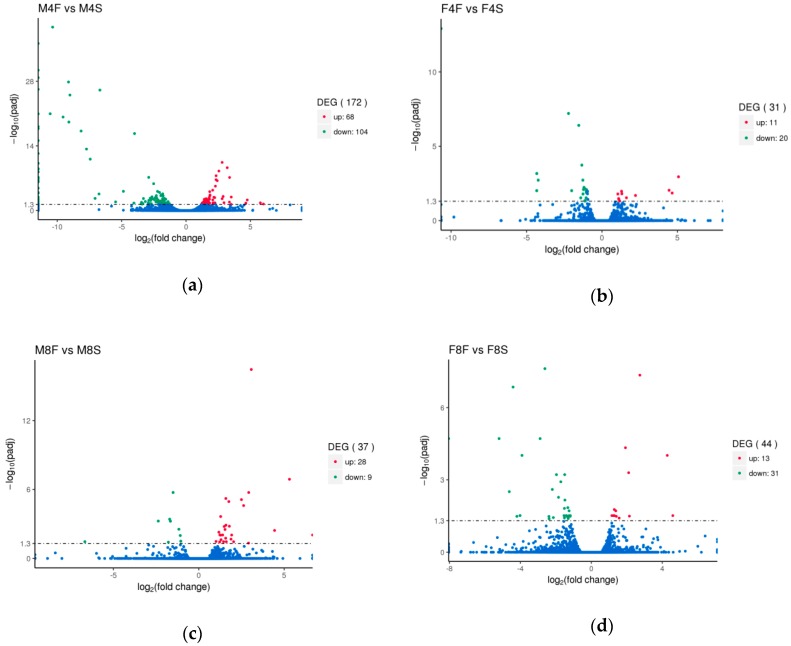
Volcano maps of differentially expressed genes. (**a**) Volcano map of differentially expressed genes for four-week-old males. (**b**) Volcano map of differentially expressed genes for four-week-old females. (**c**) Volcano map of differentially expressed genes for eight-week-old males. (**d**) Volcano map of differentially expressed genes for eight-week-old females.

With a fold change ≥ 2 and FDR < 0.05 as the screening criteria [28], a total of 172 differentially expressed genes were screened in the four-week-old males (Figure 1a). Among them, there were 68 upregulated genes and 104 downregulated genes in the fast-growth group (M4F) when compared with the slow-growth group (M4S) (Supplementary Files S1, S2). A total of 31 differentially expressed genes were screened in the four-week-old females (Figure 1b). Among them, there were 11 upregulated genes and 20 downregulated genes in the fast-growth group (F4S) when compared with the slow-growth group (F4F) (Supplementary Files S3, S4). For the eight-week-old group, a total of 37 differentially expressed genes were screened in the males (Figure 1c) where there were 28 upregulated genes and 9 downregulated genes in the fast-growth group (M8F) when compared with the slow-growth group (M8S) (Supplementary Files S5, S6). A total of 44 differentially expressed genes were screened in the females (Figure 1d). Among them, there were 13 upregulated genes and 31 downregulated genes in the fast-growth group (F8F) and the slow-growth group (F8S) (Supplementary Files S7, S8). 

**Figure 2:**
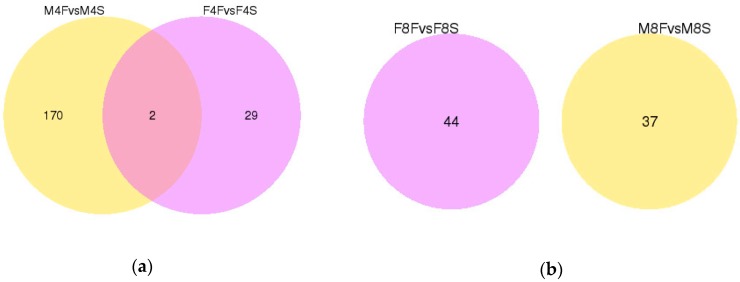

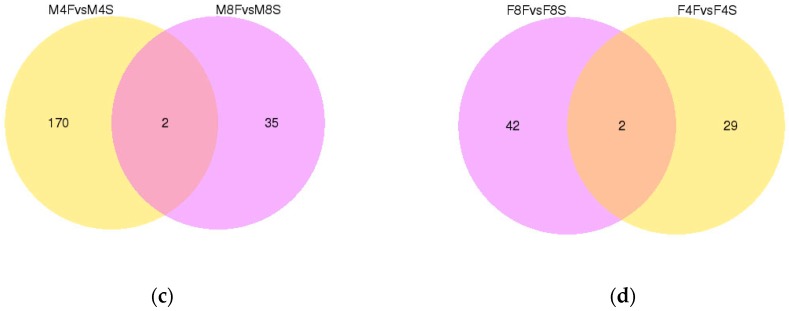
Venn diagram of differentially expressed genes. (**a**) Venn diagram of differentially expressed genes for four-week old males and females. (**b**) Venn diagram of differentially expressed genes for eight-week old males and females. (**c**) Venn diagram of differentially expressed genes for four-week and eight-week-old males. (**d**) Venn diagram of differentially expressed genes for four-week and eight-week-old females.

The Venn diagram was used to display the number of differentially expressed genes between the comparison groups, and the overlapping differentially expressed genes were further analyzed. There were two overlapping differentially expressed genes in the four-week-old males and females (Figure 2a). The two differentially expressed genes belonged to the downregulated genes, namely the *SNCG* gene (ENSGALG00000002015) and the *ARNTL* gene (ENSGALG00000005378). There was no overlapping differentially expressed genes in the eight-week-old males and females (Figure 2b). There were two overlapping differentially expressed genes in male individuals at four weeks and eight weeks of age (Figure 2c). One of them was *VAMP1*, and the other was the *MCL1* gene, which belongs to the upregulated gene. There were two overlapping differentially expressed genes in females at four weeks and eight weeks of age (Figure 2d). One of the genes (*ENSGALG00000035235*) is unannotated and has no transcript, and the other is a novel transcript.

**Figure 3:**
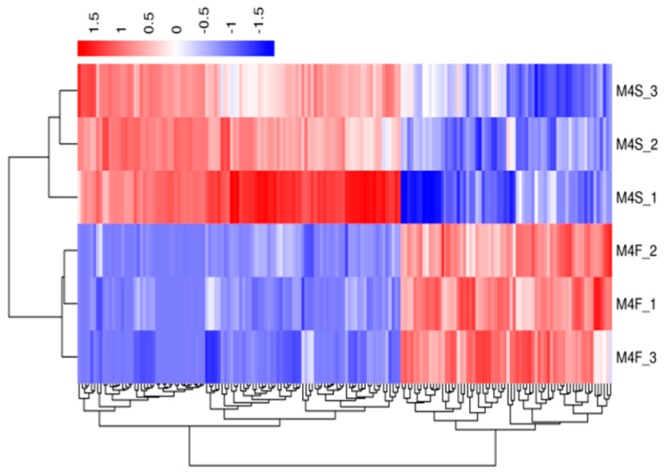
Overall FPKM (fragments per kilobase length from a gene per million fragments) hierarchical clustering map of the four-week-old males.

**Figure 4:**
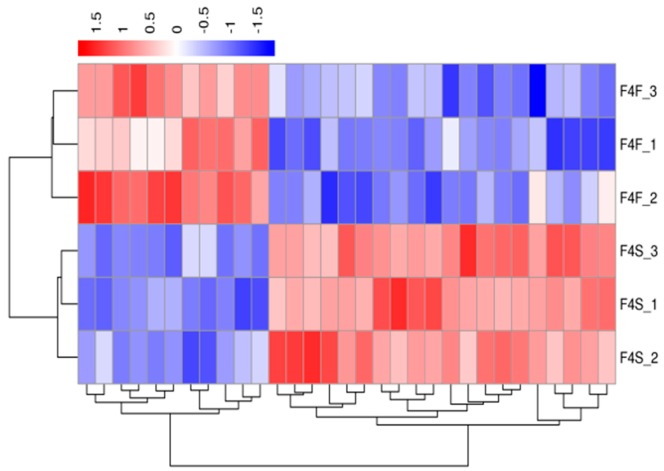
Overall FPKM hierarchical clustering map of the four-week-old females.

**Figure 5:**
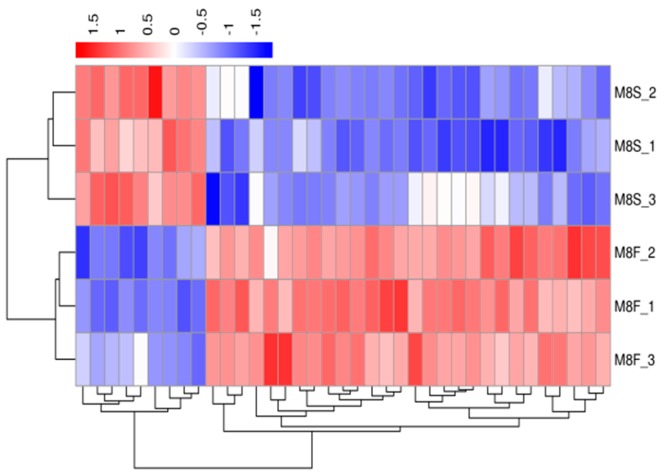
Overall FPKM hierarchical clustering map of the eight-week-old males.

**Figure 6:**
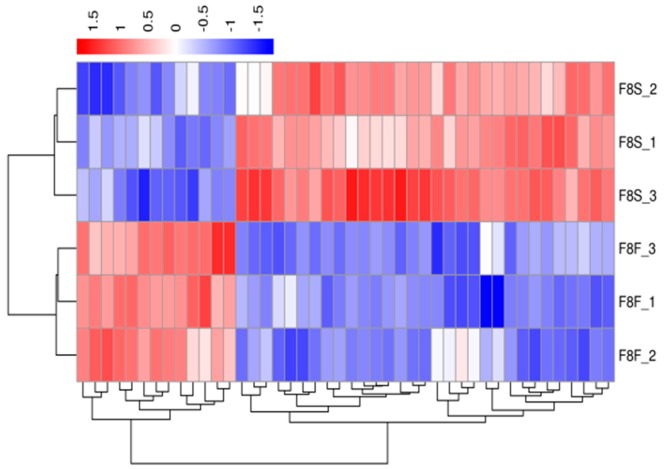
Overall FPKM hierarchical clustering map of the eight-week-old females.

A hierarchical cluster analysis was applied to the differentially expressed genes. We calculated the distance between the samples using the expression of different genes in each sample and determined the correlation between the samples. Figure 3, Figure 4, Figure 5 and Figure 6 show that the fast-growth and slow-growth individuals were all clustered together, respectively, for different genders at different growth stages, which illustrated the accuracy and reliability of the samples.

### 3.5. GO Annotation and Enrichment Analysis of Differentially Expressed Genes

The method used for the GO enrichment analysis in sequencing data analysis was GOseq, which is based on the Wallenius non-central hyper-geometric distribution [25]. The results showed that there were 31 differentially expressed genes in the four-week-old females, of which 16 genes were annotated. A total of 15 genes were unannotated, and there were 5 new transcripts among the unannotated genes. Significant analysis of the GO enrichment of differentially expressed genes revealed that there were 154 significantly enriched entries in the biological process category (*p* < 0.05) (Supplementary File S9). Three biological process enriched items were significantly associated with the growth and development (Table 5). There were 172 differentially expressed genes in the four-week-old males, 104 genes in the differentially expressed genes were annotated, 68 genes were not annotated, and there were 30 new transcripts in the unannotated genes. The analysis showed that there were 328 significantly enriched entries in the biological process category (*p* < 0.05) (Supplementary File S10). Four biological process enriched items were significantly associated with growth and development (Table 6). There were 44 differentially expressed genes in eight-week-old females, 26 genes in the differentially expressed genes were annotated, and 18 genes were unannotated with eight new transcripts in the uncommented. The analysis showed that there were 337 significantly enriched entries in the biological process category (*p* < 0.05) (Supplementary File S11). Four biological process enriched items were significantly associated with growth and development (Table 7). Eight-week-old males had 37 differentially expressed genes, 22 of which were annotated, 15 were unannotated, and there were five new transcripts in the unannotated genes. The analysis showed that there were significantly enriched entries in the biological process category (*p* < 0.05) (Supplementary File S12). Six biological process enriched items were significantly associated with growth and development (Table 8).

### 3.6. Pathway Enrichment Analysis of Differentially Expressed Genes

Pathway significant enrichment analysis was conducted using pathway in the KEGG database and hypergeometric tests were applied to find the pathway that was significantly enriched in the differentially expressed genes when compared to the entire genomic background. The analysis found that four-week-old females had one significantly enriched signaling pathway (*p* < 0.05) (Table 9), which was the herpes simplex infection (gga05168) where two genes were enriched on the pathways. The analysis also found that four-week-old males had four significantly enriched signaling pathways (*p* < 0.05) (Table 10), which were steroidal biosynthesis (gga00100), cell cycle (gga04110), DNA replication (gga03030), and ribosome (gga03010). Correspondingly, six, eight, four, and three genes were enriched on these four pathways. Furthermore, it was found that eight-week-old males had one significantly enriched signaling pathway (*p* < 0.05) (Table 11), which was the P53 signaling pathway (gga04115), where two genes were enriched on the pathways.

### 3.7. Validation of RNA-Seq Data by Quantitative Real-Time PCR

**Figure 7:**
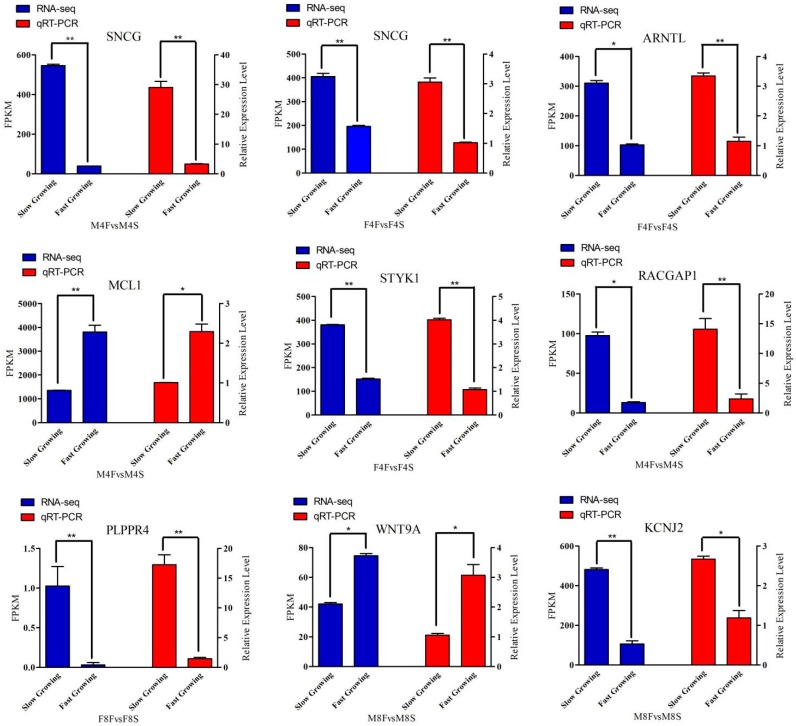
Expression level of eight DEGs detected by RNA-Seq and qRT-PCR. “*”: 0.01 ≤ *p* < 0.05; “**”: *p* < 0.01.

To validate the RNA-seq expression data, eight genes, which may be related to muscle growth, were selected from two groups of male and female for QRT-PCR validation. *SNCG*, *ARNTL*, and *MCL1* were the overlapping genes. *STYK1*, *RACGAP1*, *PLPPR4*, *WNT9A*, and *KCJN2* were the candidate genes selected from the four groups. The results (Figure 7) show that the expression trends between the fast-growth group and the slow-growth group were consistent in the QRT-PCR results, and the reliability of the sequencing data was high.

## 4. Discussion

Growth traits are important economic traits and have always been the focus of attention in the development of the livestock products industry. Studies on genes related to chicken growth traits are a hot topic of interest. Goto et al. [29] believed that growth traits are important quantitative traits that are affected by genes and the environment. Classical quantitative genetics hold that growth traits are controlled by multiple genes, each of which plays a minor role, and are regulated by the additive effects of the genes. With the development of modern molecular biology, it was found that major genes also exist in regulating chicken growth traits. In the present research, transcriptome sequencing and QRT-PCR techniques were used to analyze the differentially expressed genes between fast-growth and slow-growth traits in the Jinghai Yellow chicken, and some candidate genes that might affect growth rate and muscle growth were screened.

In our previous research on Jinghai Yellow chicken, Zhang et al. [30] detected 18 SNPs that were significantly associated with the growth traits, and *LfOC771741* and *FRLY* could be the candidate genes that affected the body weight of four-week-old females, whereas six candidate genes (*SETDB2*, *ATP7B*, *INTS6*, *KPNA3*, *DLEU7*, and *FOXO1A*) surrounding the significant SNPs were obtained by Abdalhag et al. [31]. Among these genes, *INTS6*, *KPNA3*, and *FOXO1A* were the candidate genes that affected the body weight of the eight-week-old females. No common genes were found in a comparison with our present results. One of the reasons for the lack of common genes may be that the gene chip for screening SNP sites is only 60 K, and the range is too small to contain the differentially expressed genes found by transcriptome sequencing. Another reason may be that the differentially expressed genes in transcriptome sequencing affects the growth trait by regulating the expression level of mRNA, while the gene selected in GWAS changes the SNP site, which may affect the amino acid sequence and lead to the conformational change of the protein and finally cause the change in growth trait. As the two modes of regulation are different, the differentially expressed genes detected in the transcriptome sequencing did not include the genes screened in GWAS. 

In the present research, through GO enrichment analysis and KEGG analysis, many genes such as, *SNCG*, *ARNTL*, *DNA2*, *MCL1*, *WNT9A*, *MYOD1*, *KCNJ2*, *GDF8*, *PLK1*, *RACGAP1*, *PLPPR4*, etc., were found to be involved in the skeletal muscle differentiation, cell secretion, cell proliferation, regulation of cell division, and the regulation of metabolic processes. *SNCG* and *ARNTL* were common in both differentially expressed genes in females and males at four-week-old, and the expression difference between the fast-growth and slow-growth groups was extremely significant (*p* < 0.01), while there were no common differentially expressed genes in the eight-week-old males and females. The reason for a small overlap in genes between male and female chickens may be due to the different mechanisms in regulating chicken growth and development in chickens of different genders. Shen et al. [32] found that the *SNCG* gene significantly inhibited the formation and growth of breast cancer (*p* < 0.05). Li et al. [33] stated that the expression of the *SNCG* gene is related to the growth, invasion, metastasis, and prognosis of breast cancer cells. *ARNTL* is a clock gene, and Peng et al. [34] found that *ARNTL* hypermethylation can promote tumorigenesis by activating CDK5 transcription in nasopharyngeal carcinoma. *DNA2* gene mutations in estrogen-dependent cancers are concentrated in the helicase and nuclease structures. Strauss et al. [35] found that mutations in ovarian cancer impaired *DNA2* activity. Miller et al. [36] found that the helicase function of *DNA2* was required in the terminal excision of budding yeast cells lacking exonuclease 1, which is a new role for the *DNA2* transporter function in DNA break resection.

There were two overlapping differentially expressed genes in the male individuals at four weeks and eight weeks of age in the present research. One of them was *VAMP1*, and the other was the *MCL1* gene. There were also two overlapping differentially expressed genes in females at four weeks and eight weeks of age. One of the genes, *ENSGALG00000035235*, was unannotated, and the other was a novel transcript (*Novel01285*). The common genes between the different age groups were very low, which might be because the genes that regulate the quantitative traits are different at different growth stages. For the common gene *MCL1*, studies have found that the gene is closely related to human tumor formation [37,38]. Cui et al. [39] found that *PTBP1* enhanced miR-101-directed AgO_2_ targeting of *MCL1* and promoted miR-101-induced apoptosis. Watson et al. [40] found that survival of endothelial cells during angiogenesis required the pre-survival protein *MCL1*, and the presence of *MCL1* protein may be closely related to vascular density. 

GO enrichment analysis showed that *WNT9A* was present in the four significantly enriched biological process terms (GO:0008285, GO:0008283, GO:2000026, and GO:0045595) for eight-week-old males. Richter et al. [41] found that the regulation of the *WNT9A* gene expression played an important role in the differentiation of human embryonic stem cells into hematopoietic stem cells, indicating that this gene has an effect on embryonic stem cell differentiation. *MYOD1* is a member of the myogenic regulatory factors (MRF) and GO enrichment analysis showed that the gene was present in all of the significantly enriched biological process terms for the eight-week-old males. Many studies have shown that the *MYOD1* gene is an important gene for tissue differentiation during skeletal muscle regeneration, which may be related to skeletal muscle growth and the proliferation of myocytes [42,43].

GO enrichment analysis showed that the *KCNJ2* and *GDF8* genes were in the same biological process (GO:0003012). According to existing research, these genes may be related to muscle protein production, muscle fiber diameter, muscle fiber density, and participate in muscle regulation [44,45,46]. Fernlund et al. [47] found that mutations in the *KCNJ2* gene may be involved in the formation of VAndersen–Tawil syndrome (ATS) syndrome, suggesting that the *KCNJ2* gene has an effect on the regulation of human cardiac activity. The Polo-like kinase (PLK) family is a highly conserved serine/threonine protein kinase that plays a crucial role in cell cycle regulation [48]. Ren et al. [49] described how the *PLK1* gene promoted the M phase from the G2 phase by promoting the cell cycle and promoted chromosome segregation and centrosome maturation to regulate cell cycle. The *PLK1* gene was significantly different in the fast-growth group when compared with the slow-growth group (*p* < 0.01) for the four-week-old males. GO analysis showed that the gene was present in several significant enriched biological process items such as the regulation of cell division (GO:0051302), microtubule cytoskeleton organization involved in mitosis (GO:1902850), and cell cycle (GO:0007049). The enrichment analysis revealed that the gene was significantly enriched in the cell cycle (gga04110) pathway.

The expression difference of the *RACGAP1* gene between the fast-growth group and the slow-growth group was extremely significant (*p* < 0.01). The GO enrichment analysis of the *RACGAP1* gene was present in three items: the assembly of actomyosin apparatus involved in cytokinesis (GO:0000912), actomyosin contractile ring organization (GO:0044837), and microtubule cytoskeleton organization involved in mitosis (GO:1902850). This indicates that the gene may play a role in muscle growth and development, but *RACGAP1* has not been reported in chickens. Li et al. [50] found that *RacGAP1* and *Cyclin D1* were highly expressed in gastric cancer tissues and may play a synergistic role in the development of gastric cancer. The results of Lau et al. [51] showed that *RACGAP1* may promote the proliferation of cancer cells by shortening the cell cycle, but the specific mechanism is still unclear. The gene can be functionally verified in the late stage by combining the fast-growth group and the slow-growth group.

The results of the transcriptome sequencing and QRT-PCR expression analysis showed that the expression of the *PLPPR4* gene between the fast-growth group and slow-growth group was significantly different for the eight-week-old females (*p* < 0.01). Yu et al. [52] found that the *LPPR4* (*PLPPR4*) gene is an important gene in the LPPR family that regulates cell function and participates in synergistic effects. Zhang et al. [53] found that upregulation of the *LPPR4* gene can inhibit the migration of vascular smooth muscle cells, and the downregulation of this gene can promote the migration of vascular smooth muscle. The above studies indicate that the *PLPPR4* gene may play a regulatory role in the growth process.

## 5. Conclusions

In this study, we analyzed the differentially expressed genes between fast-growth and slow-growth groups in four-week- and eight-week-old Jinghai Yellow chickens. Through GO enrichment analysis and KEGG analysis, many genes were found to be related to cell proliferation and differentiation, muscle growth, and cell division such as, *SNCG*, *MCL1*, *ARNTL*, *PLPPR4*, *VAMP1*, etc. The results of this study will help to understand the regulation of the growth and development of Jinghai Yellow chicken and provide a theoretical basis for improving the growth rate of Chinese local chicken breeds.

## Figures and Tables

**Table 1:** Primers for quantitative real-time PCR (QRT-PCR).

Gene Name	Product Size (bp)	TM (°C)	Primer Sequence (5′–3′)
*SNCG*	160	60	F: ATACCAGGGAGCACAGAA R: ATGAGGCGAGTGAAAGAC
*ARNTL*	130	60	F: TTGTTGTGGGCTGTGA R: TTGGCAATGTCTTTCG
*MCL1*	165	60	F: ACGGCGTGATGCAGAAAC R: AGGCACCAAATGAGATGAGC
*STYK1*	136	60	F: TTGGTTGGATGCTGTGTAGA R: CAGTGAGGTCGTAGGGGAAA
*PLPPR4*	204	60	F: AAAGTCATTCCCATCTCAAC R: AGAAAGCCACAGTAAACATC
*RACGAP1*	164	60	F: CCATAGTGGCAAAGACGA R: GGATTCCACCTGCTTAGAG
*WNT9A*	188	60	F: AGAAGCAGCGCAGGAT R: AATGGCGTAGAGGAAGG
*KCNJ2*	187	60	F: GCACCTTGGGATTGGCTTGG R: GAGAGGCTGGAGTCCCCAAA
*β-actin*	169	60	F: CAGCCATCTTTCTTGGGTAT R: CTGTGATCTCCTTCTGCATCC

**Table 2:** Body weights of four-week-old and eight-week-old individuals in the fast- and slow-growth groups.

Sample Group	Fast-Growth Weight (g)	Slow-Growth Weight (g)
M4	316.00 ± 20.07 ^A^	210.00 ± 32.78 ^B^
F4	306.67 ± 5.77 ^A^	203.33 ± 22.55 ^B^
M8	948.33 ± 35.47 ^A^	571.67 ± 40.41 ^B^
F8	761.67 ± 16.07 ^A^	540.00 ± 35.00 ^B^

The difference in the same row with different uppercase letters is very significant (*p* < 0.01). M4: four-week-old male; F4: four-week-old female; M8: eight-week-old male; F8: eight-week-old female.

**Table 3:** Data quality from sequencing the four-week-old samples.

Sample Name	Raw Reads	Clean Reads	Clean Bases	Q20 (%)	Q30 (%)	GC (%)
M4F-1	61,102,148	57,553,102	8.63G	95.78	90.66	54.20
M4F-2	63,629,812	60,018,588	9.00G	95.67	90.43	54.17
M4F-3	62,940,690	59,343,986	8.90G	95.68	90.56	54.90
F4F-1	57,332,426	53,990,618	8.10G	95.55	90.24	54.58
F4F-2	65,164,376	61,663,414	9.25G	95.63	90.36	54.13
F4F-3	51,857,508	49,118,588	7.37G	96.58	92.14	54.51
M4S-1	52,827,100	50,822,036	7.62G	97.28	93.27	50.82
M4S-2	54,511,420	51,581,852	7.74G	96.56	92.07	53.38
M4S-3	50,497,564	47,770,614	7.17G	96.55	92.13	54.80
F4S-1	53,045,954	50,337,158	7.55G	96.46	91.93	54.68
F4S-2	56,550,890	53,588,300	8.04G	96.49	92.03	54.43
F4S-3	59,058,956	56,081,520	8.41G	96.45	91.88	53.89

Clean reads: total number of pair-end reads in the clean data; clean bases: total number of bases in the clean data; GC (%): percentage of G and C bases in the clean data; Q20 (%): the percentage of the Q20 base; Q30 (%): the percentage of the Q30 base. Similarly hereinafter.

**Table 4:** Data quality from sequencing the eight-week-old samples.

Sample Name	Raw Reads	Clean Reads	Clean Bases	Q20 (%)	Q30 (%)	GC (%)
M8F-1	52,721,010	50,382,466	7.56G	96.80	92.54	54.03
M8F-2	57,894,528	54,856,958	8.23G	96.61	92.21	54.64
M8F-3	62,623,374	59,583,598	8.94G	96.59	92.19	54.04
F8F-1	62,961,680	59,233,086	8.88G	96.66	92.22	54.19
F8F-2	52,949,754	50,073,278	7.51G	96.78	92.52	54.58
F8F-3	54,514,850	51,990,782	7.80G	96.91	92.69	52.85
M8S-1	52,367,686	49,864,380	7.48G	96.58	92.12	54.20
M8S-2	56,144,980	53,234,338	7.99G	96.41	91.80	54.21
M8S-3	51,435,032	48,911,326	7.34G	96.54	92.06	53.52
F8S-1	52,731,880	49,998,426	7.50G	96.79	92.51	54.28
F8S-2	52,558,780	49,491,048	7.42G	96.82	92.55	54.40
F8S-3	65,234,778	60,733,042	9.11G	96.55	92.10	53.89

**Table 5:** The significantly enriched biological process terms for the four-week-old females.

Term ID	Functional Description	Number of Genes	*p*-Value	Gene Name
GO:0032940	Secretion by cell	4	0.0020300	*SNCG*, *ARNTL*, *MICAL3*, *STYK1*
GO:1903530	Regulation of secretion by cell	3	0.0065730	*SNCG*, *ARNTL*, *STKY1*
GO:0051046	Regulation of secretion	3	0.0080530	*SNCG*, *ARNTL*, *STKY1*

**Table 6:** The significantly enriched biological process terms for the four-week-old males.

Term ID	Functional Description	Number of Genes	*p*-Value	Gene Name
GO:0035914	Skeletal muscle cell differentiation	3	0.0090062	*ARNTL*, *EGR1*, *NR4A1*
GO:0051302	Regulation of cell division	4	0.0099481	*PLK1*, *CXCR5*, *CDC20*, *TTK*, *RACGAP1*
GO:1902850	Microtubule cytoskeleton organization involved in mitosis	4	0.0005303	*PLK1*, *CDC20*, *RACGAP1*, *ENSGALG00000039964* (ID)
GO:0007049	Cell cycle	10	0.0485280	*DNA2*, *ARNTL*, *PLK1*, *CXCR5GINS1*, *CDC20*, *TTK*, *CCNB3*, *RACGAP1*, *ENSGALG00000039964* (ID)

**Table 7:** The significantly enriched biological process terms for the eight-week-old females.

Term ID	Functional Description	Number of Genes	*p*-Value	Gene Name
GO:1902532	Negative regulation of intracellular signal transduction	3	0.0016997	*DUSP8*, *MYOC*, *DDIT4*
GO:0048638	Regulation of developmental growth	2	0.0049708	*HOPX*, *ARX*
GO:0019222	Regulation of metabolic process	12	0.0071640	*ALDH1A2*, *DUSP8*, *HOPX*, *PRMT8*, *DCUN1D5*, *ARX*, *MYOC*, *TDRKH*, *DDIT4*, *ENSGALG00000016826* (ID), *ENSGALG00000024470* (ID), *ENSGALG00000026776* (ID)
GO:0048639	Positive regulation of developmental growth	2	0.0148180	*HOPX*, *ARX*

**Table 8:** The significantly enriched biological process terms for the eight-week-old males.

Term ID	Functional Description	Number of Genes	*p*-Value	Gene Name
GO:0008285	Negative regulation of cell proliferation	6	0.0000196	*WNT9A*, *MYOD1*, *GDF8*, *PLXNB3*, *CDKN1A*, *DPT*
GO:2000291	Regulation of myoblast proliferation	2	0.0001083	*GDF8*, *MYOD1*
GO:0008283	Cell proliferation	8	0.0002199	*WNT9A*, *MYOD1*, *GDF8*, *PLXNB3*, *CDKN1A*, *CDH2*, *FKBP1B*, *DPT*
GO:2000026	Regulation of multicellular organismal development	8	0.0002504	*SEMA7A*, *WNT9A*, *CDH2*, *MYOD1*, *PLXNB3*, *GDF8*, *ARHGDIB*, *FKBP1B*
GO:0003012	Muscle system process	4	0.0003995	*KCNJ2*, *MYOD1*, *FKBP1B*, *GDF8*
GO:0045595	Regulation of cell differentiation	7	0.0007171	*SEMA7A*, *WNT9A*, *CDH2*, *MYOD1*, *PLXNB3*, *GDF8*, *FKBP1B*

**Table 9:** The significantly enriched pathway of the four-week-old females.

KEGG-Pathway	Signal Path	Number of Genes	*p*-Value	Gene Name
gga05168	Herpes simplex infection	2	0.00780496	*ARNEL*, *PER3*

**Table 10:** The significantly enriched pathway for the four-week-old males.

KEGG-Pathway	Signal Path	Number of Genes	*p*-Value	Gene Name
gga00100	Steroid biosynthesis	6	5.59 × 10^−8^	*LSS*, *DHCR7*, *DHCR24*, *NSDHL*, *CYP51A1*, *SQLE*
gga04110	Cell cycle	8	1.77 × 10^−6^	*MCM2*, *PLK1*, *CDC20*, *MCM5*, *TTK*, *MCM3*, *CCNB3*, *MYC*
gga03030	DNA replication	4	0.00028685	*MCM2*, *MCM3*, *MCM5*, *DNA2*
gga03010	Ribosome	3	0.03040881	*MRPL17*, *RPL22L1*, *ENSGALG00000040263* (ID)

**Table 11:** The significantly enriched pathway for eight-week-old males.

KEGG-Pathway	Signal Path	Number of Genes	*p*-Value	Gene Name
gga04115	p53 signaling pathway	2	0.00323550	*COP1*, *CDKN1A*

## Supplementary Materials

The following are available online at https://www.mdpi.com/2073-4425/10/7/539/s1, File S1: M4FvsM4S differentially expressed genes, File S2: M4FvsM4S differentially expressed new transcripts’ sequences, File S3: F4FvsF4S differentially expressed genes, File S4: F4FvsF4S differentially expressed new transcripts’ sequences, File S5: M8FvsM8S differentially expressed genes, File S6: M8FvsM8S differentially expressed new transcripts’ sequences, File S7: F8FvsF8S differentially expressed genes, File S8: F8FvsF8S differentially expressed new transcripts’ sequences, File S9: Statistics of GO enrichment items in F4FvsF4S, File S10: Statistics of GO enrichment items in M4FvsM4S, File S11: Statistics of GO enrichment items in F8FvsF8S, File S12: Statistics of GO enrichment items in M8FvsM8S.
