# Supplementary material for: Transcriptome Analysis of Differentially Expressed Genes Related to the Growth and Development of the Jinghai Yellow Chicken
Source: Genes (Basel). 2019 Jul 17;10(7):539. doi: 10.3390/genes10070539 (PMC6678745; doi:10.3390/genes10070539)
Supplement: Supplementary file 1 [file genes-10-00539-s001.zip › genes-527003-supplementary/Supplementary files 1-12/File S2 M4FvsM4S differentially expressed new transcriptsí» sequences.doc]

Novel01496

CAAGCACTAGATCAGGTTGCCAAGACCCTCCATCCAGCCTTGCATTGAACACCTCCAGAGATGGAGTATCAACAGCTTCTCTGGGTGTATGATAGAATGGCTTATGCCAAAGGGCTCCTTAAAGATCGTTTAGTTCCAACTCCCTTGCCATCAGCAGGGTTGCAAGTGATAAGATGGGGCTGCCCAGCTTCCATCCAGACCTTGAATGCCTCCAGGAATGGGGCATCAGCAGCTTCTCTGAGCAATTTGTTTCAGTTACTCACCACCATCTGAGTAAAAAATTTCTTTTAACATCTGAACTAAATCTCTCCTCTTTTAGTTTAAAGCCATTCCCCCTTGTCACTATCAGACCCCTCCAGTTTAAAAGCTCCCTTGAAGTACTGGAAGGCCACAATGAGGTCTTTGCAGACCCTCCTCCAAGATAAACCAGGCCAACTCTCTGAGTGTGTCTTTGCAGAAGTCCAGTCCTCTTATCATCCTTGTGGCCCTACTCTAGACCCTTTCCAAAAGCTCCACCTATTTTCTGTGTTGCAGGCCCCATAACTGGGCACAGTACTCTAGACGGGGCCTGAAAACAGCAGAGTACAGAGAGACAATCACCTCCATTGCCCTCCTGGCCTCTCCTGATGCAGCCTGAGATGCTGTTAGCTTTCCAGGCTGCAAGCACACACTGCTGGCTCACGTTGTTTGTCATCTGCCAGGACTCCCAAGTCCTTCTCAGCAGGGCTGCTCTCAAGGAGTTCTTCTCCCAGTCTGCCAGTCAAGTCCAGCCCCCCACTCAAAGCAGCACTCAGGAACTCTGGAACGTGCATCTGCTTCCTACCTTCAGGTCAACACTGTACACAGTGACATTTTTTACATCTGAACTGTACTAAGGGCAAAATACATTAGTAATGCTGGATTCACAAGTGCTTCAATTATCAAATTCTTTGCTGGCTTTGAGAGCCACAGAGCTATTTGTACTGTATCCTGTAGATCCATCAACATCTAACTGTTGCTCTTGAAACCTTTCCATTAGTCAGGTCTCTCAAGGGCAGAGCAGAGAGGGACAATTACCTGCGTTGCCCTCCCGGCCACTCCTCTTTTAATGCAGCCTGAGACACTGTTGGCTTCCAGGCTGCAAGCACGCACTGCTGGCTCACGTTGTTTGTCATCTGCCACGACCCCCAAGTCCTTCTCAGCATGGCTGCTCTCATTGAGATCTTCTCCCACTCCGTACTCATAGCTGGGATTGTGCTGACCCAGGCGTACTTGGCCTCGTTGAACATCATTAGGGTTACATGGACCCAGGGCTGTTCAGGTCCCTCTGGATGGCATCCCTTCCTTCTGTCATATCAACTGCACCACTCAACTCAGTGTCACCTGCAAACTTGCTGAGGGTGCACTCAGTGCCATCATCCAGGACACTGACGAAGATGTTAAAGAGTACTGGTCCCAAGACAGACCTTGGAGGACACCACTAATGACTGGCTTCTACCTGGACGTAGAGCCACTGACCAGAGCTGTCTGGCTGCAACCTTCCAACCAATTTCTTATCCACCCAATAATCCACCCTTTGAAACCATATCTCTCCAATTTAGAGATAAAAGTACATCATTCTCTTAATTATTAGCAGCTGGGAAAAAAAAAAAAAAAAAAAAAAGACTCCCATCATCTGTAAAAACTGGCTAGAGACCTCCAGAAGAAGAATGATAGCTGAAATTGTTCAGATATTTTATTGTATGTTCTCTGAAAGACGATTTATGGTGCTTCTTCCAGAAAATGCTTTTGGCTTGCAGTGCTCAGTAACTGTTCAATTAGGCTGATGAATTCTGGGAATGTTGCTGTTGCTATCTAAATCAGGGAGATAATCAGTTTGCATGTGGAGGTGGGATTTTTATTAATCAGCGGTGCCAGATAGACTTATGAATCAAAAACTTCCCCCAGTACTGTAGTCTCCCTCTCCAACCTGAGCATGGAAGTTCTGCAACCTGCTGCACTGCGCCCTAAAGTTCTAGCACTTAGCAACTGGGACTGGAGGCAGGCGAGTGCTGGAAAAACAACTTGAAACATGCTGAAAACATTCCTTTGCCATTAAAAAGGCAAGGCAGCAAGTGCGCCTGCCTTGAGGAAAAACTGCTGCACAGCTCACTGCATAAATATGCGTGTGCATATGTGGCATAATGGTCTGTGCATAGGCATGCACCTACAAATAGGGAAGCAAACTCTTTGTTGCAAGAGCTGTGAACTTCCCTGAGTAATTAACTGTGCTTTTAGTGCATCTCCTTGGATCTGCAGTCAGTAGCAGAGGCAAAGCCTCTTTTAAATCTTCAAGTGTCAAACTCAGAGATAGCAGCTAGCTAAGCTGAGGCACTAAGAGAAGCAGAAATGTCACTGTAATAGCTTGTGTCTCTTTGGTGCTGTTAAAAAAAAACAACTGTGCCAGTGGACAGAGTTATCCACAGAGCAAAAAACTAATTCTCATGAGATTATTTGGCTTTGTGTACCCAAAGGCATTTGATGCAACTGTGTGTGTGGATAGATAGACACTGTTCTTCTGTTTTGGCGTGGTTACATAAACGTGTGACAACGTAATATTTAGGGGAAAAGTAATCCATGCCTACCTTTGTTTCCTGTCCTGCTGTGAGCATTTTGCAAGCCAGCACGTGCGCTTGGACTGCTCCCCCAGTAGAGAGAGGTCCTGTATGAAACCTGGCAGGTAAGTACATGGCATTGATACCGAGTCTGTTCATTGAGTGGAAAGAAAAAGGGGAACATTTTATGGTATTGGTAATGATATGACGTTTTGGAAAGGAACACCTTTTTTTCTTTTTTTTCCCCCACACATGGAACAACTGCATGTAAATGAGAACTGTAAAAACTGACTTTTTTTTTTTTTTTTTTTACCTCAGAATGACTATTCTAGCTGCAAACTGGAAGTGTGGTTTGCTTAAATAGTTCGCTTGCAAAACTGAACTTGCAGAACTCAATTGAGAACGTGTCTAGGGGATTAGAAATCTCTTTGGCTCGTCTGCAGGGTCTGAGAGCATCCAGGGCACAGTAATTCCCCACTGCACCCAATGAAAAGTGGTATCAGAGAGTGGTTTAGTTAGCATGCACTTCAGTACACAATTGCTGTGACTCAGATATGTCCCCAGCTGTGTTTTATGCTGCAGAACTTGTGCTGGTAAGAGACACCTCTGAAACAGGGCAAATTTCACGTGTGGGAATCAGCAGTGGGAAGCTTTCAGAACAAACATCTCTCTCTCGTGCCTGATCCCTAATTTTAGACCTTTGTTGCATAAAATTGGATGAAGATGTTAATGTTTGTGGCTGTGACACTCACATTCAGAAGTGAACCAGCACTGAACTGCAGATCTTGTGTTTGCAGACAGTCTGGAGCTTCGGCATTCAGAACTGAGTGGTTTTCGAGCCACCTCTCAAATGCAACAATTGTGGAAGCCCACAGCCATATTATATTTACCTAGCTGTAACTAACACTGCTTCACTGTCATCTGAGCTCAAGACTCTGTTGTGAAAGAACATCTACATTGCGTAAGGGCTTTCTGACAAAGGGAAGCTAATGCCATCAAAATACTCAGCGCTTCAGCAGCGGGGTGATGGCACGCTCCCTGTCCTCCAGGAATTTGGATGATTTCTTACTGCTTCAGAGTGTTCTAGAGACAAATCACAGAATCACAGAATCACAGAATGGCCTGGGTTGATCTGGCATGATCAGCCCTTTAATGGTGTAAAATCCATGTTGTCAAATAGTACGTGGAATGTCTGATAAAGCTCACCATAGTTGTGTTTTCAAGCACACAAAAACCTTTCCAAACGTATCTGCAGCATAAGCGTTCTTGACTTTCCTTGGCAGATTCTTGTCTTTGATGGCCTTTGGACCACTTTCACCCTTGTGGACAGGATGGACACAAGCGGACAACTTTCATCCTTGCAATACTGTTGGAAGTTTAGAGATTGGGGTAGTCTTGAGACAGTGTGTTCTATATTACCGCAGCCCTTCCAGTTCTGTCTGTATCAGGATGATAGCTGAGTGTGATTCTGCTCAGCTTTTTCCTGTGAGCAGCCCCACTGCTGCCAAGGAGACTGGAGATGTAGGTCACGTCTGACTCTCAGCCAGATGCTAAGCGTTCATGAGACACCACATGCTTCCCAGCTGCTTGTTCCACTTGGAGTCTGGGATAACCCAGCTTGGAAGGGAGCTTGGGATCTTTCTTCAAACTCTAGCTGAAAGCATGATCAGCTATCAAGTCAGGCCAGGCCTCAGGCCGCACCCGGTTGGGTCCTGAAAATTTGGGTGGTATTCTTACATCTCAACCCTCTGCCGAGTGTCTGGAGAGAAAAAAAACTCCTCAGCTCTGATCTTAGTGTGGAGTCACGTGTGCGTTGCGCCCAAGTCAGGATGTGGAATGTGGAATTTCTGCTCTTTGTTTTGGCTGGCTCGATGGCCTCTGCGGAGTGAGCTCCAAGAACGCACAGACTGGGAGCTGTGTGCATTATCTGAGCTTAATTAGTCTGCTTGGATTTGTGCACTGAGCAGGATGTGATTCTGAATGTGAGTCAGGGCAGCGTGTTGAAGGATGTTGCTTGCCTTTCATGTGAATGGCCCAGAGGCCTGAGATCTGCAGGAGATACCAAAGGACGGACTGTGGTTAAGGCGATCAGCCGCTGCCCGGTTGGGTGGATGCCATCCAAATGCTGCAGGACTCTGAGAGTGTGAAGCAAGTGACTTCAACCAGCTGTGTCAAATGTTTCTCACTTCCTGAGTGCTCACACACAGTGCTTGACATCAGAAGCTGTGCTTTTACAGCTCTGACTAGAGGCAATAGAAGGGCTGTGGTGGTGGTGGTGGTAGTGGGGGTGGTGATGCTGATAGTGCTCCAGCCTCTCCCAGCCAAAGCCAGCACTGCTCTGGAGGATGCAAATACTGCTGAGCTGCCTTCTAAAGGGAGGCAGACAAACTCCGTGGCTTTACCATGACTGAATAGGTGACTGCTGTTCTGTGTGGGAGCTGTTGGTGCCGCAGTGCCCCAGATATCTGCCTCCAGGGTCAGGCCTCCTTTCTGTCCTCCTGCTTCTCACTTTCCATGGCTAAACCAGAGGGAATCGTTTTCAGAGCCCCAGTCTTTCAGCAGAAGTGGTCGCTATCTGCGTTCCCATCAAGTGTAGGAACAGGAGAGCTCAAGTAAGGTTTCCCATGAGGCTTCTGATTTCGGAATTGGAATCTTCTGCTTCTGGAGCTTGCATTGGCTGCCTCATACCTTCAGCATCCCTCTTTATCCCTTTGATCTGTCACTTTATTTCCATCCACGACACACGAGGCGAGGGCTTTATGGGGCAGAGAGCACACAGAGCAGGTGGGGGACTTCAGCAGTCAGCCCCGCCGTGAGTACTTGGAGTGACTTGATGCTGCAGAACAATTTCTGCCCATTTGGGTAGAAAATGTTCTCCCCCAGGTACTGCAAACAAGGACAAACATGTCTTTGTTTTCCTGCTACTGAACAGTGTGTTTTGATAGGAAGCATGCAGCTCACCTTCAGTTTCCATCTAGCACTGGGAAATACAAATAAATGCATAAGCTGCCATGCGTTTGGGAAGGGACAGACTGCTCCCACAGCCCGTTTGGTAGTGTGCACACTGAGGTCTGATTTAAGTAACAGCGGTGTGTCACAGGCCAGGGAGGACTGCACACATCATGCACGATCTCCTTATAAAAAGCTCTAACTAGCTAGAGCGTGATACTGAGCCTGTCAGCTCAGGGCAGGCATTTCAAGCACGCAAGCAACAGGTGTCTCAGCAAAACCACGGGTCTCTGAGGAGGGGCTGTGCTTTAAATACCCAAGTGACCGCATCTGTTTGGTGACAGGTCACCCAGCAACCCGTGCTGCCCCTGGTCTGTGAGGTGGTGTGGCCTCTGCAGTGCTTTGCAGTACGGTCAGCGCTGCATGCACGGCCCCCGCAGTGCTTCCTGGAAGACCAGGCTGCCCGAAAAACTAATAGAGGGGAAGAAAGCAAACAGTGCCTTCTAGAATTGGAGTGTATGTAAAATGCAAAGGTGCACGACTGTGTGACTGAAAACCGAATAAAATCGTGTCCTTGGCCACTTTTCCCTCTGGGAGCTCTGCCCCAGCGTGTGTCACGCTTAACCTCAGCAATTGGGCACACAGAATTTAGCTTAGCGAAGCAAACTGGTGGGTCGTTTGTCATGAGGGACATAAACTCTCTCCTAAAACAAATATCTCTGAGACTCTGACAGCGGGGCCTCTGCTCAGGACATAGTTATGACTTTGCCTGTGTCCTACAGACACCTTTGTGATCAGCAGGATGTGTTGCTAGCAGACCCGCAGGGGAGCTTGGTTCCAAGCTGCTGGAGGGCATACAGGAGCTTGGTTCTGCCCTTTGCAAAGGAGCTGCAACCTTGGAAAGCCAGTGGTCTTAAGGTGCCTTAAGGCGACCCAGAGCATGTTTTTTGTTGCTACAAGGCTAGACAAATATTAGGAGTGAAGTAACTGAGCAGTGTGAAGGCTGAACAAAGCAGCTTTTCACCCTTCCTTTGCCGATAGGAGTGAGCTACCGTGGAGTATGAGCGGCCCCGAGTTGTATGAGCAACTGGAAGGAGTAAACAGTAATGCTGAAATTTATCCCAGGAACGCTAGTGAGAAAGCATCGTGGAGCCACTTAAGAGCACTAACACAGTAAAATGCATTGTCACTGGCAGGAAAGCTGGATGCTGGGCTGCATGGTCATGGCACTGTGATTTTTTGCAGCCAACAAAGCGAGGAGTGTGCGATTCTTTAGTGATGTCACAACCTCAGTGTTGCCGTTGTGTGTTTTTTAGCATATCCACATCGTATGAAACTTAAGTTACGTGAGTGTAGAGATGTTTGAAAGGGAAGGATTTCTTTGTTGCTCCCCACTCCTCCTCAGTGAGAGCAGGACGTGTGCCCCAGTACAGTGCACGTGTGATGTGAAACCAGTGTGACACGAGGGAGGAAGAAACCCTGTTTTCAAGAGATTAATATCAGGTATTAATTGGAGTGGACAGGGCATTTTTACAAGACTGCTCCAGAAAGATGGGTTTGTTAGATAGATGGTGGGACATCTAGCATTTACACATCCACTTTTAAGATCACTTCCTGCAATGCTTTTTCTCTAGCAGCAGCACAGTCAGTGCCTGGTGCCATAGTGGGAGAGTACAAAGGAGCAAAGATAAATCTAAAGTTCTGATGGAGCAGCTGCTTGGTTGTGCCTCCTGAGTCTCCCACTGAGCAGGATGACGCTGGCTGCAGCTCTCTGGCTTACAGGTCCATCCATGTGTTAATACTAAATATCTTCACGCTGCCAGCAGCCCACTCAGCTTTGATCTCCACAGGTCCCGTTGCAGTTTGTCTAAGTGGCCACGATCCAACTGATGCTAACACAGTCCTCACAAGGAAAGTAACGTCATGAACTGCAGTGAACACATAGATGTTTTTCCTTTCCTCCTCTCTCCCTTTCTATTTTCAAACTCCATCCATTTTACAAGCCTTTGGGAGCAAGAGCATGCTGTGTCTAGGAAGCAGTGGGATTGGCACCTGCTGTCAACGGCATGAAAACCCTCAAGAACACATGTGTAGCTATGCATAGTTAGCATTAACCCAGGCCTGAGGGAACCCTTGGCAGCCCCTCTCTGTTCCCAGGCCCACAGGCAGCCCCGTGGGGCTTGGGCTGCACCACAGGCTGTGGTGGGCTGCTCACATAGCTCCCTCACTGCAGCAGGGCAGTGGGTGAGGCCAGGGACTGTTCTGCTGTCCAGAACAGGTCATCTTGCTAGCACCAGAACCTGCTGACTTCTGGTCAGTTAGACCAGTCCGTTAATATCAAATGCATCTCCCTGTTTTGTCTGCCTTAGCTCTTGCAACCCTGGTTTGCAAAAGTGAAACACTCCAAAAACATCAGTGGGAAGCAAGGTTGGGCAAGTGGAGCCCTGGGATACTGCAGGCTGAGCAGAGGGGGCAAAGGACATCCCTGGGAGTGATGAACAGAGTGGGTGCCCTGCAAAAAACTTGAAGATATTGCCATTAAAAAAACCTCACTTTGCTTCCTCTTGATGTGTTTCCTTATTAGTAGTGCTGAGGGCTCAATAACCCTTGGGATCAGCTTTACCCATAAAAAGCAGACTTTAAAATTAACAGAGCTCAGTGTGAGTTGCAGCAGAAATCACAGCAGCACTGCACTTGTATTTTCCATTTTAGTGTGATCCATTCTTTGCTATTCCTGCACGTCTGCTTGTGTTTCTACAGTACAAGTGCTTGTTAGATGGTCCTGCAGGGAATCATGCAGTGGAGAAAACGTGACTTGTTTATCTTGCAATAGCTTCCCTTGAAAGGGAAAATCCTGCGGTCAGCACACGTATGATGTTCTGCAAAATGTGGCAAAGGAGTAGGGTTTGGTACCTGTAATGCTACAAAGGCTCAGGAGCACACTGCTATCCTTGCAGCTTTCATGGCTTCCATCGTCTGCTTGTTCCATCTGTCCCTGCTCCCCACTCTGTGAGGGCACAGGGCTCAGACAGGCTTGGGCACCTGGGCAGACGTGTGCAAGCTATTATGGGTATAGAGTATATTATCATATGTATCACCACATATATCACGTAATATATATCATTGTGTAAATTATTAACAATTCTGACTGCAAGGGTCCTTCAGAAAGGGAACCTAAAAGTAAAACAGAAAGAAACTCACGCGTCTTTGGGTCAGCCCTCCACTTCTGGTTCTGGTTTCTCTGTAACTGTGACACAGAGTTATGACTATGCACGTTCACTTCTCAGGATGCTAGGAAATTCTATGGTAGTACTAGGAAGTATGGCATTTCGTTTCTACCTTCTGGAAGTTAATTTGTTGACACCTTGATTAAATGAAACAATAATAAGTAAATAAATGCAATACATCAGCTGGGATTGATGTTCTGCCCTATTTAATACCAACTGGCAGTCAGACGAGGGTATTACTAAGTTGAGCTATTCATGAGCTTCATAACGAACAAACTAATCTGAAGGTTATGTTGCTGAAAGGTGTGTTTCTGTGAGTACAAAATACCCCAGTTCAGTGTGAGAAACCTATTACCTATTCCAGAAGACTTGAGAGTTGGAAGCTTTCCAGGAAAACGTGTGCTTCTCCTGAATAATTTGTTTAGAGTGGTATTTCCTTGCTGGCACTCAGATGAAGGCGACTGAAGCCCCAAGCAAGGGGAGGGGACTCTGAAGCTGCTCGTGAAGCTGCATCGTTGCTGGAGATGACCCAGACAACGGTCAATGTCAGCAGCACCAGCGCTGGATGTCAATACTACCTGTGCCACTTTGCCACAGTGCTGAGGGACTAAAGGTGCAACTGGGTCCAGAATGGGATTAGACACGTTAAACTGGTAGTTCAGGGCCTGATCCAGCTGCAGCTGTGCAATGCTGGCTCTGTGCCTGAGAAAGAGCTTTCCCTTCTATGTTCTTTATGTCAAATCAGGGCCTGGAACGCCCTGCTCATACTCCCCACGCCTAGACCATTCCATCCTGGTATAACTCATGTCTGAAAGCTGTGAAAGGTGGATAGCAAGCCCCAAGAGGAGCAATTAATGAAGTTCTTGTTCCAGCAATTGTGCTCATCTCATCTCATCCCTACACTAGTCACTCATGTAGTACATGAGCATGATGTCCACTGTAGGGTGCTGTTTCTGGCCCTTTCACCTTCCCAACAGCTTTCAGCAGCTCACTGTCTGGATCCTTCCCCTGCAGTGGTCAAGCAGCCCAAGACAGGGCTCTCCAGGGTGAAGTGGCAGTGGTCTCAGCTTCCCACAAGGTTTGCACAGAACCCAAAGTGATATGGGGTGGGAGGTCAGGGTCCCACAGCTGTGTCCCATGGTGAGCACAGCACGGTGTAGCTGCAGGAACGTAAAGAGGTTGGGTCTACACAAAAAGAGGGCAGTGACTAAATTTGGAATGACCTACTTTGGCCATGCAAATTGAGTTACTAGGTACTAATGAAATCCCACGTAAGGAGTTGCAAGGCAGTGAGCAGAGGGAGCTGCAGTGACCTGTGGAAGGTCACTTTCAGACAGTCCCCTGTAGAGCACGCATGCACATGAGAAAAAAGGGCTTGGAAACAGAAGTAACAGAAGCAGTAAATAAGCCTCCTGCACAGAATTTCAAATCGGTGCACAGAGGTCTTATGACTGCCTTTAAAAATCTGTCAGTAAAGATGCTGAACAAAGGGCAAGGGTGGATCATCCAAGCCTGGTTCGTTCAAAGAGCCTTCCCCCATCCCACCTCGTGGTGACCACCTCTGCCAGTGTAAGTAACGCGCAGAACTGCGATGGAGCAATCTGTGAGAACAGGGAGGGATTTGTAATGTGGTGTTCCCCTCTTGCATAATGTTATGTAAGAGGTAATAATGGATCTGTGCATGGATTTTAGTGAGTATCTTTTAGCCTGACGGGACATGCGATGCACTTTGAGGCAGTGATAACCACACTGGAACACAAACTGAACTTCCTTGGAAGGAAGTCCAAAGGCTGGATTCTGTTCTTGGGAGAGTTTTTATCCAGATATTTGAGGCCTGAAACTTGGATCTTGTTTAGCACTTGCACTGCAGGTGGCAAGGGCTGCAGTGATGGGGTGAGCTGGGCTCCAAGTACTCCAAGACTTTGTTAAGTCACTGAGATCTTTGCTGATGATGGCCTTGGCTCAGTAGACGAAGTGAAGGTGTAGGTGGTGGGATGCTGCACAGCAGATGTGTTTTGTAGTGGTCTCCACGTGTGAAACCTGGGACGTGCATGGTCCTGAAGGTAAAACGCCCTTACTGCACGGTGGAAGTATGAAAGCTGAGCAGACTCAGGGTGACATGGGCTGTCACGATGGGTTTCTGGGGTTTCTAAGTAGCAGGTCTGATCGACTGGTTGCTCCAAATTATTTAACTGAAGAGTGGGGGGGAAAGAAGAGGGATTGCTACCTATTACCTTGTTTCTAAGTTTGAAATCAATAGGTTATGAAGTGGATAGACCCTATCTCTCCTAAAGGAAGCTCCTTGTCTTGCACACACCCTTGCATTCTCAGGCCAGCAGGACACATGAACACTTCAGAGTCTTTGCCCTGTGGAAACTACGTAGGACTTTCCAAAGATAGCCAAGAGCCTCACCGAAATAAATTAAACTGACATTAATAGGCTGGAATTTATAACGAAAGGTTTGCATGTACCCTAGCAGTCTAAATCCAGCCTCCTGGCTCTTATCTGTAACATCTAAGCCAGCTAGAAAGACCTCTCAAAGTGAAACACGATCATTTCCTGGCTATGTGCCTTTGCTTCTCTTCAAAGCCAGTCTTTGCTGAGAAAATAACCACCTGTTCATTTTATGCAGGGCAGCATTTCGTAGGACTCTCCTTGATGCATGGTCACTACTGGGGATTTTGGCCATTCCTTCCTAACTTCATGACTTGTCACCTGCAAAGGAAATACACAGGGAGAGCAGCGTATAGGCAGCACTGGACAGCTGCTGTGAGAGCTGCTGGCTGCATAACCTCTGCACCAGGACTGGGGGAGCGAATTCCAGCCTTGACATTGCTGTGATTGACTTTTGGTGGTGAACCCAAGGGCCAGAAGCAGACCACCCCAAACCCCAGGCAGCACAGGGGGTCCATGCTTTGCATTAATCCCGTGCTGAGACCTCCACTGCCCAGCATCACAAATTTCAGGGCTGTAATGCTGCAGCAGTTTGTGGAAGCAGCATACCTGGAAGAATCTCCCCAGCACAAAATCATGCTAAGTTATCGATAGGAAGATTTTGGAAGGAGTGCAGAGGACATCATCTGGCAGCTTCTGGTTTACATTTCCTCTTTCCATGAATAATTGGAATGCTTCAATTGAATTGCAGTCTTGGCTGTATGTATTTATCGCCAGATGTATTGGAGCCTTTCATCGTAGGGACTTAAAGCAAATTATTTAAGTCACAACACATCCTCGGGAGAAATACTGTCATTTTACTGATAGACAAAGAGTCCAGGAACACAGAAATAGTCAATTGGCCAGAAGACATGGGAAACTATCAGGAGTGGGAGGAAGGGAGCTGGGGTCTCTTGAGGTTTCTGAGCAGCATGCACTCAGATGCAGATTCTTTATGGAAACCAAGCTTGACTGCAGGATACTGTGTCCCTGTTACTTCAAGGTACTTGTACTGGAAAAGATGATTTCTAACATTTCCCTTCCAAGGGCAAAAATACTTTCCTAATCTCCTGAAAATGCAATGCTGATTCAAGGCAAAGTCACTGCACTGTTCCTTCTCCTGTGTTTAAGAATATCTCACCTTTATAACCAAAGTAAACAGGCTGCCAGCTGCCAGGATGGGACTCCAAGGTGCTCAATTTGGCATCACGAATGAAGTTGCCTTTGAGTAAACAACAGAGTAAATAAAGGCATGTTGATGGTTCTGC

Novel01481

GCTAAATCAGAAATAATGTTGCCATGGCTGTTGATACAGTTTGGTTCCTTTTTTTAACCTTCCTGACTCTTATTAATTTCAGGAAACTATGAAGGATAAAGTAGAATACATATTCTACTACAGTGAAACAACAGTGAACTTAGGTGCATTCATCCATTCATTCACAGTTTGTGTTCTATAGGTTTGATCTTTTAAATTATAAGCAGAAAAAGTACCTGCTGTTTCATAACCCTTCCAACACTGATCATTAGTCCTTAGTAAATCCCTTTCATGAGCAATCTTAGTTGGATCTCAAGAATCTAAATTTCAATAATTTAGATGAAGTATCTAAATAGCTGTAAAAGATTATTCTTTTATATCACTGACACAAAGTTTGCTGAGGAACAAGTCCAGGCAAGTCCTGGGCAAAGGTAGAGAAATCTTCTGTCTGGAGGACACTGATGGAGAGGTCCTGGCTAAGGGTTGTGAAATCCTTTTAGGAGCACAGATGGACAAGACCAGGGGTAACGAGAGAGGGATAAGCTGCTGATAATGGCCGGGAAATGGTCTTTTGTGTGGACTTATCTCGAGGAAGATGGCCATCTCAGGAGGTACGCACATGACTCTTGCTCAAGCACCCAGGGATGTCACGTAGGCAGGAAAAAAGGAGGACAAAAGAGAGTCCAACAACCATGAGGATAGGTTTCTGCCATCAGATCCCTCACCACGACGGACTCGAGGTTTCTGCCTTCAGATCCCTCACTACGGACCGTATGCTGACGGACTTCCCTGGTCCTGCTACCTGAGGCCTGCTGCTTCCTCCGGGCTTACTCTGAGGCTTCTTCCTGGACCCACCCGGTACCTGCACCCTCTCATTGCCCAAGACCAGCCTCGCTGCTCCTGCCCTTCGGCCTCAGACCATCGGAACGGCATGCAACGGGACTACTGCCAGATCCTGGTGGTGACTATCCCCGCTTTACGCAATTCTTGCTTCTTTCTATCTTTTCAATCACTCACCTTCCCTTCCCCATCACTCCAATCCGTAATAGTGTCCGTCCTCCCTTTCTTCATCTCCCTTATTAACATTTGTAATAAACTGGTTGGACCAACATTT

Novel01474

GTTAGCTCCCCCTTCCATCCTTCCGATTCGTTCCTCATCACAGCTGGGGAGGTTGCGTGGTGTTCCGCTTTCTGCCAGGTTAAGCAGCGATAACTGCCGGCACCTGCAGCCGCTCAGCAGAGCGGCGCCTCCAGCCCGCCGTTCTTCCTGGCTACCAGCTGGAGGCGCCGGGTGGATGCGTGCAGGGGCGGGGAAAGGGCTGCGGGTGAAGGGGAGGACCCAGTGCTACTGGGCCCGCCCGCAGGTAGGATGGGACAAGACAGAAAATCCCTCCCACCTACCTACACAGGGAGGCGCCATTTTGTTCCAGTCTGCATTCCAGGGTGAGGAGCCGCCATTTCATGAGCCAGCGGAGGCGCCATTTTGTTTCAGCCCGAATTCCACAGTCCGGAGCCACCATCTTGTGGCACTTAGTAGGCCACGTGTTTTCGCACTGCTCTGTGATTGGCCTCTCTTTCAAAGGCTGTCGCTTTCTCAGGAGTCCCTCTGATTGGCTGCCCAATTCCTCTGGGCCGGAGAGCGCTCTGATGTTACAATATATACATTTTGATACAATCGTCCCCGCTGTAGGGGGCGGGTGCCCACGTGCTGGCACTGCGGGTTGCCAGCTGCCTCTGAGGAGAAGGCAGGGCTGCCCCCTGTCAAAACCAGCCGTTTCCATCCAGCTCCAGCGGCACTGCCACAAGGCACAACCGAGCCCCACACCCAAGGTGGAGGCGTCCTGGGGGAAACGTCCCGAGGAAAGGGCAAGAAGGGGCAGAATGCTGCCCAGCAGTGGGGAGAGAGGGTGGAACCGTGTGAGAGACAGCCACGTGAATGCCCGAGTGAGAAGCTGGGGAGCGGGAGGCGGAGAGGGAGGAGGAGAAGGAGGTGGAGCGTGAGGAGGAGGAGGTGGAGCCGGAGCAAAGGGTGGAGAAAAAGCATGGGCAGGAGGAGAAACAGAAGGAGGTGCTCCAAGCGCTAGGGCAGAGATTCTCCCGCAGCCGCTGCTGGAGGCGGCGGGGCACGTCACCACACCCCAAGGGATGCTGCAGCCTGCTCAGAGCCCACACAGGTACAGGGGAGAAGTGGAGGAGGAAGGAGCGGCTGAGCAGAGCTGTGGGGGGACTGACCGCTACCGCCCCTCACCCCTGCACCTCTCACGCTGCGGGGACGTCGAGGACTCGTGCGTGAAGGAGGGTATTTGGGCTCGGGGAAGAGGGGAGGGGGGTCAGGTGCTTTTGTTTCTCACCATCCAAGTCTGTCCAAAATGTGCAATGACTCCAAGCAACTTCCCCCACGTCTATTCTGCTTTGCCTCTGGTGAGCGAGCTCCCTGTCCGTGTCTTGACCCATGAGAATGCCCATCGCCTTCCCTACTCTGTCCCGTTGAGGACACTGAGTGACCGAGTAGCTGGCTGTGCATCGGGCTGCTGGCTGAGGTCAGCCCGCCACTGCAACTTCTACAGTACATACAGACACAGAATTATTAAAAAGGATTTGATAAAGGTAACTGAAAATATTGTCTCCATGCCACGTAGGTACACCACTATACTACACTAGGTAGTTGTCGTGGTTTTGTCTGGGAGACTGCCCATTTTCCTCGTACCGCCTCGCACGGGTTGGTGGGCTCTTTTGCGTTTCTGACAAAAACGGTGGTGCTAACGCATCCACGTTGTAGTTCCTTCCTGCTGTTGTTGCGGAGCAGCGCCCACACTGAGCCCAGGACTTTTCTGCTTCTTACGGCGCCCTGCCAGCAAGGAGGTTGGCAGTGCACGAGAAGATGGCACGCGACACAGACCGCACAGCATCAGGCTGCTGGCCACGGTCGGCGCGACACGGCAGTCTATGTATAGAAATTACACATACATTCAAATGCAGGGAGACCTTCATACAGGTAAGTAAAGCTTTTTTTCGCCTTCATATTCAGCCACGCTGCACACCGTGCTAGATAAGAGCTGGTTACGTGCAGTGAGCCCTGGGATCCTAGAAGCGGCTGAATAGTTGCCTGTCAAAGGGAAGCGGCAAACGTTTCCCTTGGCTTGCGGGCACGCCTTTTCATTTACCTAGCAAACCGTCTTTACCTAAACCCGCGTGTGCTCCCACTTCCATCCTTCCGATTCTTTCCCCATCACAGCTGGGGAGGCTGCGTGGTGTTCCGCTTTCTGCCAGGTTAAGCAGCGACAACTACACATAACAGGCACACAGGCATCCAGACCGAGCAGTTAAACCTATGCCTGTACTCCTCAAAGCGTAGCTGTGAAACGGGCAGGGCGTTCCTTCGTGCCGGCTAACAAAATAAAGAATTCAAGTTTCAGTGGGAATAGAAACAGTCCAACGTGAGCAGAAGGCCAGGTCGGCGACCCGCCCGTTAGCAGTCTGCAGAAAAGCCCCTGGCACAGCAGCGGTGGGGGAGCTTCAAGAGGGCGGGCGCTCGGCCAAAACAGGGGGCACGAGGATGCCTCCCAGGTGGCCACGCTGGGCCACGGCCTGGAGGGACATCTTGCCCGGCTACTGATCCTCAAACTTGCCAGCGGGAGCGACCCTGACAACTCGAGTGACCGGACACATCCCCGGGCCTTATGCCAACCTGCATAACAGACCACGCAGGCTTCCTAGGGCAGGCCTGGGGCTTCACATGGGGATGGGAAAGTCAGGCAGCGCTCCCCGCCGTTTGCATACATGGAAAAACAGAGCAAAACGCCACCTTCAGCTGAGCCTGCAACCCTTTCTTCCTTAATCGTTGCGCTATGAATTTTGCTGCCTGGATGTGACATGGCACACTCACAAAAGAAGGCACTTGGACACAAGCATGCGCATCATCCAGACAAGCGCCAGTGCAATTCAAGGCACATACGCAGGGTCGGGACCAGGCGCACGTTCACCTGCTCTCTCATCTTCCCTCTTTACCATAGGAGCTGCCCACCTTGCAATTTAGAAAGGTGCAGAAAACCGCTCCTCAAAAGGAATGCCTAGCTCCCCCACCCCCCTGCGCCCCGAATCCTAGATGTGGGCTCGTTCAGCTACACGCGATGTGCAGCAGAGCACCTCCTACCACTCGGACGGAGAGCCACCGGGCAGCGCGGCCGGCACCTGCAGCCGCTCAGCAGAGCGGCGCCTCCAGCCCGCCGTTCTTCCTGGCTACCAGCTGGAGGCGCCGGGTGGATGCGTGCAGGGGCGGGGAAAGGGCTGCGGGTGAAGGGGAGGACCCAGTGCTACTGGGCCCGCCCGCAGGTAGGATGGGACAAGACAGAAAATCCCTCCCACCTATCTACACAGGGAGGCGCCATTTTGTTCCAGTCTGCATTCCAGGGTGAGGAGCCGCCATTTCATGAGCCAGCGGAGGCGCCATTTTGTTTCAGCCCGAATTCCACAGTCCGGAGCCACCATCTTGTGGCACTTAGTAGGCCACGTGTTTTCGCACTGCTCTGTGATTGGCCTCTCTTTCAAAGGCTGTCGCTTTCTCAGGAGTCCCTCTGATTGGCTGCCCAATTCCTCTGGGCCGGAGAGCGCTCTGATGTTACAATATATACATTTTGATACAATCGTCCCCGCTGTAGGGGGCGGGTGCCCACGTGCTGGCACTGCGGGTTGCCAGCTGCCTCTGAGGAGAAGGCAGGGCTGCCCCCTGTCAAAACCAGCCGTTTCCATCCAGCTCCAGCGGCACTGCCACAAGGCACAACCGAGCCCCACACCCAAGGTGGAGGCGTCCTGGGGGAAACGTCCCGAGGAAAGGGCAAGAAGGGGCAGAATGCTGCCCAGCAGTGGGGAGAGAGGGTGGAACCGTGTGAGAGACAGCCACGTGAATGCCCGAGTGAGAAGCTGGGGAGCGGGAGGCGGAGAGGGAGGAGGAGAAGGAGGTGGAGCGTGAGGAGGAGGAGGTGGAGCCGGAGCAAAGGGTGGAGAAAAAGCATGGGCAGGAGGAGAAACAGAAGGAGGTGCTCCAAGCGCTAGGGCAGAGATTCTCCCGCAGCCGCTGCTGGAGGCGGCGGGGCACGTCACCACACCCCAAGGGATGCTGCAGCCTGCTCAGAGCCCACACAGGTACAGGGGAGAAGTGGAGGAGGAAGGAGCGGCCGAGCAGAGCTGTGGGGGGACTGACCGCTACCGCCCCTCACCCCTGCACCTCTCACGCTGCGGGGACGTCGAGGACTCGTGCGTGAAGGAGGGTATTTGGGCTCGGGGAAGAGGGGAGGGGGGTCAGGTGCTTTTGTTTCTCACCATCCAAGTCTGTCCAAAATGTGCAATGACTCCAAGCAACTTCCCCCACGTCTATTCTGCTTTGCCTCTGGTGAGCGAGCTCCCTGTCCGTGTCTTGACCCACGAGAATGCCCATCGCCTTCCCTACTCTGTCCCGTTGAGGACACTGAGTGACCGAGTAGCTGGCTGTGCATCGGGCTGCTGGCTGAGGTCAGCCCGCCACTGCAACTTCTACAGTACATACAGACACAGAATTATTAAAAAGGATTTGATAAAGGTAACTGAAAATATTGTCTCCATGCCACGTAGGTACACCACTATACTACACTAGGTAGTTGTCGTGGTTTTGTCTGGGAGACTGCCCATTTTCCTCGTACCGCCTCGCACGGGTTGGTGGGCTCTTTTGCGTTTCTGACAAAAACGGTGGTGCTAACGCATCCACGTTGTAGTTCCTTCCTGCTGTTGTTGCGGAGCAGCGCCCACACTGAGCCCAGGACTTTTCTGCTTCTTATTGTGCCCTACTACAATGCACAACACACCCCACGGATTGCTCTTGCATGTTACTTGCTAGAATATTTTTTGTTTTGTTTTGTCTTCTTCTTATATGTTTGGTTTTTTTATTTTAGTTTCAACTGATTTTTTTTATTATTATTTAATCGTTTGCAGGGCAGTGTTTTAGTTTTCTGTCTTTTCTCCCTTCACTACTGCTCTCTCAGCTCTGGCTTCCTGCTCATTTTAATCTCACTTTACTTTGTACTTTTCCAGTTCATCACTGGTTATTTTCTCTTGAATACTGTTGTGAATAATACTTCCACATTCTTTGCTACAATTTGACTACATCCTGTACTCTTTTATCTCTTTTTATTCACTAGGTGGATAGTCTTCTCATAAAATGTTATTGTCTTTTTTCATTAATTATTTTCTCTTTGTGTACCTTTGTTTTTGTTTTGTTTTGTTTGTTTGTTTGTTTTTGTTTTTTATTAATATTTCTTTCATCTTAGACTAGTCTTCTATTTTATTTTGCCGGGCGAGGATATAAAGCGGACTGCTCCCTTATCACAGTGTTTTTTTCTTATCCTTCTTATCCTTCTTAAAAACTAGTACATTTGCTAGTTCTGTCTGTACTTTTTTTTTTTTTTTTTCCTCTAATCTTGCTTTCTGGGGCATTTTTTGCAATAAGACTCTGACTTATCTAGGAGGCACGTTGTGTGTTTCATCATGTAGCAGTCAACATCACCTTGTGGACAGCTTTACATTTGAATGACTCAGTTTGTTTACTGACAGAAGATCTGGCTTGTGACCAGGTAACTCCAGCATGGTCCAGGAGACTGGGGGATGATACCATTGTATGCTGTGACGTCAAGATATAAGTGGGCACCCATCCTTCTCCCTCTTATCTGCTGGCAAGGTTTGGAAGACAGGAAAAAAAGTAGTTCCTACTGAGGTCCAAAAACCAGAAACTTTCACTCCAAAGAGAACTGACTCAACCACTTTGGACTTACATGTTTGTACTGTCACTGTTAGCTGTTGTCAGCTCAACAACACTACCTCTGCTGACCTATCATTACCAAACATGAGCATCTGAATTACAAACTATGAGCTACGCTGTTATTCCAGTGATGACTCTCTCTCTCTTGCTTTCTACAATGACTCTTTGCTCTTATTTCCTATCTCTTTTTTATCACCCATCTTCCCATTCCACCTCGTTAAGCAACTAGGATTTGTAATAAGCTGTTCAGGCTGACATTTGACTCATTGCGTCATACTCTTGCCATTGTGTATACACATATGAAATAATCTCCACTTCCTCCTGTAATTGGAGAAAGACAACTTCCCAGTTGACTGGGACTCCCCATATTCCCAAGTTGTTCATAACTAACTGTGAGTGTTCTCACGGTAACATAGGCCAACATTTTCAGTCCTCTGGGATATATCTCATCAGACCCCATAGACTTATGTGCATTCAACTGAAGCAGAAAATCCTGAACAAGCTCAGCTTCAGCTGGAAGCTAATTACTCACCCAAACATGGTATTCCAGCCCAGGATTCAATGGGACCGAAAGAAGGCATTAAACATTTCAGCTTTACATTCACCCCTTTTTGTGAGGTGACCAACCTTATCAATTAACTTAACAGTTTTCTATATTATTCCTCTTGCTGTTAAACATACTTTAAAAAGACTTTCTTGTTGTTCACCACTGCACTGGCCAGCTTCAACTACAACTGAGCTTTAGCTGTATGGATTTTCTCCCTGCAATAGCAGACAGCATCTCTATAGTCCTGTCTAACCAGGGCTTGCTTCCAAGGTTCATACACTTTTTTTCCCTTATGTAAGTTCCAAGAGAAGTCCCTCATCAGCCAAGAGAACCTTCTGTCACACTTACTTCACTTCTGACACATTGGAACTGTCCATGTTTGCTCCTGCTTTCAAAAAGAAGGTTCTTAAAAAGTGAGTAGTATTCATGAACCCCAGTGCCTTCAAAAGTGGATTTTCAGGACACCTTACTGTTTCCTTGAGCAATTCTGCTTTCCTAAAATCTCAGGTAGCATTTCTGTATTTTTTTGCAGATCAGCGAGGATTTAAAATTAGAGATTTTATGGTCACTTTTCCCATGACAGCCACTAATCACTTCAATCTCAAGACTTTCTCTATTTATAGATAATAGATCTAGGAAGGCAGCTCTCCCAGTTAGCTCCCTTAATAGTTGGTAAATATATATATATATATATATATCCTCAACATACTTTGTAAATTCCCTGGACTTGTTCATGTCTGCTGTATGATTTTCCCAGAGAACACCTGGAAGTTGAAGTTGCCAATCACAACAAGGGAAACTGATCTAGAGATTCTTCTAATTTTTTTATAATATTCTCTATCCTGTCAGTTCTCATGTGTCACTGACATCATTCTGGCTTGGTGATTTGTAGTAGACTCCTACAATAACATCTGCTTTATTAGTTTTTTCCCTAACCCATACCCAGAATCTCTCATCCATGTAATCTCTAACTGCCAGCCTTGTATAGTTGAACTCCTTTTGCATACAGTGTTACCCCTCTGTCTGACCTGACCTGCCTATGTCTCTTGAAGAACCTATAATCACCCATCATAGCACTTCAAGAACAGGACTCTTCACACAAGTTCAACCAATTGTAATGATGTTGTAGCATTGCGACTGAGCCACACTTTTAGCTCCTCTTATTTATTTTTCATACTGCATGTATTGGTATACAAACACTTCAAATACTTCTCCAAAGTACTCAGCAAAACCCTCACTAGCTTGCTGTCCAACACTGACGTGCTATTCTATTGCTTATCAATTGTAAGCCTGATTTTCTCAATGTAACCCTTCAAACCTTATTTAAAGCTCACCTGTTTCTTTCAATACCACTCCCCACAACTTGAAGGGTAGAAGGGAGTACTGCCTGTGCTCTTGATCCTTTTATCCCTCTCCACAAGGCCCTGAAGTCTTCTTGATCATCACTGGACTTTTCAACATGGCTTTGTTGCTACCTACCTGAAAAAAAGAAGAGAAGAAGGGGATAATTATCCAAGGCTTGTGCCCAGCCAGAAAGTTTCCTCATAACAACCTTAACCCAAGTCCAAGGTTGCCCAGGACTCCATCTAATGTGGCCTTGAACATGTTTCTTTTTCATGAAGGGGAATCAGAAGAAAGTTACAAAAAGTAGACAGCATGTCTAGAATGTCCATGTCTCAGATAATAGTAATTGCATATAAGATATACAGTAATAGGGAAGAATTGGAAAGGAAGGAGAAACAAAGTGAAATTTAA

Novel01464
TTCTGGCTCCCTCCTCTTGGCTCTGTTCCCCAGAGACGTGGGAGGAAGTCTGAGATTCTTTGTCACCACCTCTTCGGTGGCGGGGGGGGAGGAGGTTAGAACGGGGCGAGGCTCAGCATGATGGCGGCCGAGCGTGAGGAGGAGGTTTCGGTTACCTCTGCCAGGATGGTGGGAGCCTTCGGCGGAGTTGAGCTGGGGCGCTACTCCGTCCTATGTCGGAGCAAGGAACTGGGGTCGCAGGGCCACTTCCTGGTGGGCTCTGGTGGGGACAACCCAGGTGCTGGCGGTCCGGCTTTGTGCTCGACCCTTGAGCTTCTGGAGCTCAGCTCAGGGCCTACTCCTCTGGCGTCTGGAAGCAGCGGTGCCGTGACGACTGGACAAGGACTGAAGGTAACCGGAGGGACATTTCCTGAGCCTGGTACCGTCACTTTCTCTCTGCTGCCGCCACAGCCACCTTCCTTGGCGGGGGTGCTGAGGACTGTGGAGGAAAACGACTCACTGGATGGGCCGGAGTACGAAGAGGAGGAGGTGGCTATCCCGCTCAACGCTCCCCCAACGAACCAGTGAGTAGTAACGCTCTGGTGCCTTGCCGGTCGCCCACCACGCCTGGGTGCTTTCCCCGAGGGTGAGAGCGCAGCAGCGGGATCCTATCTTTTTCCAGTTGAAAGTATGTGCCAAAGTTCCGTGGAAGTCCTGTAGAAGTTTTGTGCCCCCCCCCCCGCTCTGACTGTGGGATTTAGACAGGAGAGTTTTGTGCGTAGGAAGTACCAAATCTTGGGACGGCTTGAGACATAATTTTTCGTTTTGGATGAATTTGTGGCCTGGCTCAGCTTTGTTTCAGGATCCTGTTTGACTTGTATGTGCAGCAGGCTCCACAGAAAGTCAGTCCTGCTTATTTGTTGCTTTGAAGCGTGTCTCATTCATCTGTTGGCCATGTCCTGTTTTATCAACTGGCAGTGGGTGAATGTGGGTGTCATAGATGCGTGCCTATATCTCGAGGAGCTATTGCCTGCCCTCTTTCCTCTAAACTAAAGTGGTAACAATACAGAATGTCTGATTTAACGATGACACTCTACAACTGTAGTCAGCTTCAGTAAAGATTGATTCACACTAGTGAACTTTTTCAGCATTTAATACTAATACAACAAATAACCAAAAGGAATGAGCTGTCCTGAATATGATTT

Novel01413

GTTGGTTGTCCCATCGCCCCATGTGGGGTCATCCCATCGCCCCATGTTGGTCGTCCCACTGCGCCATATTGGTTGTCCCATTACCCCATAAGGGGTCATCCCATTGCCCCATGTTGGGTTCTTCCATTGTGCCACGTTAGTTGTCCCATCACCCCATGTTGGTTGTCCCATTGCCCCATGTGGGGTCATCCCATTGCCCCATGTTGGTTGTCCCATTGCCCCATGTGGGGTCATCCCATTGCCCCATGTTCGTTGTCCCATTGCCCCATGTTGGTTGTCCCATTACCCCGTATGGGGTCATCCCATTGCCCCACGTTGCGTGTCCCATTGCTCAGGTTGGTTGTCCCATCGCCCCATGTAGGGTTGTCCCATTGCGCCACGTTGGTTGCCCCATTGCCCCATGTTGGGTCCTCCCATTGCCCCACACTGAGTGCCCCATTGCCCCATATGGGGTTTTCCCATTGCCCCACATTGGTTGCCCCATTGCCCCATGTTGGTTGTCCCATTCCCCCATGTAGGGTCGTCCCATTGCCCCGTGTAGGATTGCCCCACTGCTCAGGTTGGGTGTCCCATCACCCCATGTAGGGTTCTTCCATTGCCCCATGTAGGGTTCTTCCATTGCCCCATGTTGGTTGTCCCGTCGCCCCATGTGGGGTCATCCCATCGCCCCATGCTGGTCGTCCCATTGCGCCATATTGGTTGTCCCATTACCCCATGTTGGCTGTCCCATTGCCTCATGTAGGGTTACCCCACTGCCCCATGTTGGGTGTCCCATTGCGCCATCTTGGTTGCCCCGTTGCCCCATATAGGGTTGTCCCATTGCTCAGGTTGGTTACCCCATTGCTCACGTTGGGTGCCCCATTGCCCCACTTTGGTTACCCCATTGCTCAGGTTGGTTGTTCCATTGCCCCATGTGGGGTGTCCCATCGCGCCATGTTGGTTCTCCCATCGCCCCATGTAGGGTTGTCCCATTGCGCCACGTTGGTTGCCCCACTGCTCAGATTGGGTGCCCCATTGCCCCATATGGGGTTGTCCCATTGCCCCATGTTGGCTGTCCCATTGCCTCATGTAGGGTTACCCCATTGCCCCATGTTGGGTGTCCCATTGCGCCATCTTGGTTGCCCCGTTGCCCCATATAGGGTTGTCCCATTGCTCAGGTTGGTTGCCCCATTGTGCCATGTTGGTTCTCCCATTGCTCAGGTTGGGTGCCCCATTGCCCCATATGGGGCTCTCCCATTGCTCAGGTTGGTTACCCCATTGCCCCATGTTGGTTACCCCATTGCCCCCCATTGGGTGCCCCATTGCCCCACGTTGGTAACCCCATTGCCCCACATTGGTTACCCCATTGCCCCCCATTGGGTGCCCCATTGCCCCACGTTGGTTACCCCATTGCCCCACGTTGGGTGCCCCAGTGCCCCATGTAGGGTTCCCTCAATGTGCCATGTTGGGTGCCCCATTGCCCTACATTGGTTGACCCATTGCTCAGGTTAGTTGCCCCATTTTCCCCACGTTGGGTGCCCCATTGTGCCATGTTGGTTCTCCCATTGCCCCACGTTGGGTGCCCCATTGCCCCATGTAGGGTTACCCCATTGCCCCCCATTGGTTACCCCATTCCCCCCCATTGGATGCCCCATTGCCCCACATTGGGTGGCACACATCACCCTTCACGTTGGCTTTTCCCGTTGCTCTTCACGTTGGGTTCTTCCCACCGCTCTCCACCTTCGGACATTTTGGTGTGGGGAGGATGGGGGGCTGAGGGTGGCAGTCGGACCCCTCCCCAAATTAAAGTCCTTTTCCACG

Novel01384

TGGGGTTGCGCACATTATGGTTTGAGTTAAGGGCATCAAGGTTCAGGCTAGGGGTGGGACATGGGGGTTGTGTCCCCTCCCACCCTTCAAACAGGCTGAAGGATGGAAGCAGAGCTCAGCTGGTCACACCGGTGCCTCCATCTCAGGATTGAAGCCATTCCCACCCATTGAGCCACCTGCAGAGCCAGGCAGCATTCCAGGCATGGCTGCTGTCTGAGTCCCATCAGCTGGGGGTCCAACAAGGCAGACTTGGTGGCTCTGCACCCCTCCAGCCCCTTGCAGAAGGATTTTGGTGCCATTTCCCAGGATCACCTCCAGAAGATGGCAGGTGCGGAGCCGGTGTGGATTGGATTAGAAGTGTCCACAAATCAATTGAAATGGGTGGACAACTCCTCCTACAACAGCACAGAGTTTGATAACCTTCCCGTGATGGAGAATGGCTGCGGGACCTTCAAGAACACAAAAGTGGAGGGCGACGTCTGTAGTGGTGAACACAAGTGGGTCTGCCAGAAAGAACCTTTACGTCTCTATCCATAATGTGCAGGAGATGGAGGGCAACGCAGCCCCTCCCTCTGATGTGTGCCCACCCCAAAAGCCCCAGGCGTTGGGGTGAAGCTCTGCAATACGGCTCTGTGAGGAGTTTGGGCCGTGCAGACCGCAGCACTGCCCGGGCACACGGGGGGACATCGCTGCATGCGCTCATGCAATCAGCTCACAATCCTGTGTTATTTCTCTCAATAAAAAAATACCGGATTTGTTGTTCAGCAGAATGCATCTGTTTGCTCCATCCCCACTCCATTTCTTTTCTCTTCCTGCAATGGGCACTGCAGACATAGACGTGTCGGAGCCACGCTGCTGTCTGCCCGTGCTGCCCATTCAGCCCTCAGAAATGAAAGCAGCAGGCAGGAAGAGAATTATAAAAGCACACAGGCTTTGTTCACACTGCAACCACATCCTGGACCCTCCCCCTACAAAAATGGTTCATCTTCTGTGCAAGGAAAGCAGCGGTTCTGAGACATGCCTCTGGGAGTCCCAGGGAGTGCTTCTGCTGAACCCCCTCCCATCTTCACCATGAACGGACAAATGACCGATTCTGTGGTCACTGCTATGGGCCTCAGCCCAAATCTGGCACTGCTCCCCCGGGGTCCCCTCTGTGAGCTTGCCTGCGTCCCCGTCCCCTCCAAGATGGAGTTCCTATTGCAGGAAAAGAAAGGAAATGAAGTGGAGTGGGGATGGGGCATACAGACGCATTCTGCTGAACAACAAATCTGATAGTTGATGGCTGCTTTGGTTCCTTGAGAATTTGGGGTCCCCGGACCCTTCCCATTGGTTCCCATGCTGTGTATGAGCAGAAGTTGATGCCTGTTTGTTTTCCTCCAGTTCCGGGGTCCCCTGGACCCTTCCCAGTGGCTCCCAGTGGTTCCCAGCTGGGAAGGAGGTGGCACAGAGGGGTGACAGCAGCAGGGAGCATGGGATGGGCTCCCGCTGGAGGTCAGATGGACACAGGGACATCACCTCCACGCGTGGTTCTGGAGGTGGTGGAGGCCCTGCAGGCTCCATAGAGCATGGGGTGGAAGCTGAGCAGCCCCCAGGCTTTGTGAGCCGAGCCCAGTGTGGGGCAGCCGGTGGCTGGGAGGGCGGGGATGTCTGCAGCCCTCATGCCACGTGGATGCAGGGTGCGTTTGCCACCATTTCATTCCATTCTCTCACATTGGGCTTCCCGATCTGGGCTGATTTTTGCTCAAAACCACCACACCTCTGGCCATTCCCAGGCTTCCTTCTCCCCCACCCCTTCCTTTACTCCCCCATACAAGCCAGGGTGGACCCAGAACCCAACCAGCATTGCTGGTGTTTCTCAGAGAACAGCAAGCCCCCAAACCAAGCCTGCGGTGAGCACCCACCACACAGAGCATCCCTTACCGCGCTGCCGCTTAGCAGGAGGCAAACTGCCCGTAGGATGCCTTAAATCAGCATAAGTAATTTCCTCATCCATGGCTCAGAAGTTCACGAGAACGGCCCCAAAACGACAAAGTCCCGCAGCAACTTCTTGCTT

Novel01355

AAGATGTTTTTAAGATAAATTAGTAAAATCAGTCTTGGCTCTTGTAGTTGAAAATGACTACCACACAAAGTACGTGATAGCTCTACATACAAGATGATGACTAGTGAACTGTTGGTAGTGTGCATCTTTCAATTTCCCCTGCTGGGCTGTTTAAGCATAATCAGTAACAAAGAGAATGTGAACTTCCTAGACTGTAGATTTTTATTTGGCAAGGGAATTGTTCAGAGCATTGCAGCTTAGTTCAGACTCTTTGATAATGTTTTAGGAAAAAAAAAAAAAAAAACAAAAAGAAAAACATCTTTGTAATCAAGATGCCTATGGTGAGCTTGAATAATGTGTTGGAAATACTTGTTCTTGCAGCTGTGAATGAAAAAGCACCACTTGCTACTGGTGATGATGATGATGAAGTTCCAGATCTTGTTGAAAACTTTGATGAAGCTTCAAAGAATGAGGGAAATTGAACTAAGTGTCACTGAATACAGTTAGAACTAAAAAAAAAAACACATCTGCATGGGGCTGCTATTTTAAACTGTGACTGATTTTGAGTTTATTTTTATTTCCTGATAAGTTCAAGACTTGTAACTGGAAACTCCTTGAACCTGCAGCTCTTAGTTACTGCTTGTACACAGTATTATTTCTGTGACCTGGTTGAATAAACTTAT

Novel01076

CGGAGGCAAACAACGAGCTGGACATGGACCTCGGGGAGGAGGACACAGAGGAGAGCAGAGACCCCCACGACCCCGAGAGCGGCAGCATCGAGGAGGACAGGTTGCAGGTGATCTGCATGTGATCCCGGAGCTGCTCCTGCTGGATGTACACACGTAAACACACACACACACACACACACACACACACACTGGAGTGGCCCCCAGCACCTCGGAGCGCAGCGAACTGAGAATGGCCGCCATTGCCCCCCCACATCCCCTCTCCATCCCCACTGATGGATCCCCGCTCCACCCCCACTGGGGGGCTGTGATGGGTCCCCTTTCCATCCCCTTTTGGGGGGGGGGGGGGCTGTGATGGATCCCCTTTCCATCCCCTTTTGGGGGGGGGGGGGC

Novel00973
GGATGAAGAAGAGTCATTAAATGAGGTGGGCTATGATGATATTGGTGGCTGCCGGAAGCAGCTGGCTCAGATCAAAGAGATGGTAGAACTTCCTCTCCGACACCCTGCGCTCTTCAAGGCTATAGGGGTGAAGCCTCCACGTGGAATCCTGCTGTATGGTCCCCCTGGCACTGGGAAAACTTTGATTGCCCGAGCAGTGGCCAATGAAACTGGGGCTTTTTTCTTCCTGATTAATGGTAAGTTTTGTCTTATTTGGACCCAAATCTTCTGTGTAGTCATTGATCCAGCTACTGGTAGTGAATGGTTTAGATAAGGCTTATATAGTCACATTGTTTCCTTGACATCCATCCTTCCTTACTTCTTTCAGGAACCTCTTTGGAGATGCTTAGAGACCATTTGTGTAGAAGCATCTGTAGTGGCACTGTCCTTAAGCTTGATTGTTTTGTAGCAGTGGTTGAAAGACAGCTTTGTGATGAGGGAACTCGGAGTCTGTCTAGCTGGGTGTTCATTTATGTTGATGGGGCAGGGCTAGGAGTGAGGCAGTGAGGTTATTCTACATACATGGGCTTGCCTGCTTGTTTCTGTTGTTGCTAACATCTTTTATTCAGAAAAGAAGCTGATCTGTATTGCATTTCTTTTTTCTTCAAGTAAAGCACAACGCCTGTTTTGTTTCACATAATATTAATTGGGAGGAAATTTCCTGAAAG

Novel00952

AGCGCCGCAGCTCCCCGCCTGCCGGCGATGCAGCCGAGCCCGCACAGGCCTATTTCATAACAGGAGGGAGAAAAATCCTGAAAGGGCAGAGGGAGGAGGGTGGACTAAAGATGCCTTAAAGCGCAGCAGAAGCCGGAGCCGAGCTGCAGCCCCTTCTCTGCCCCGACACCTTTCACACCCTGTCACGCTCCAGCACAAACGCCGGCCCACGGGAAGGGGACGCCAGAAAACGAAACGTTGAGCCCGACACGGGCTGATCCTGGACACGTGGCTTCTTTATGACAGGGAACAGAAGTGACCTGACCAACTCAGCGACGCTGCCAGCCCGCCCGTGCCAGCCCATCGCCTTCATCGCTACTCCTCCAGCACCTCAAAAATAAAAACTATGCAAACAATAAGGGGGCGGGGAAGCAAAGCAGAGAAAACCCCAAACCACACGCTCAGAAGACTGCGTGCCCCGAAAATCTAACCAGAGCTGGTGCGGGGGCGGCAGCACCGGGACACGTTCTCACCGCTGCAGCAGATTTATGTCTTCATTGTGTGCAGTACCCACAGGGTTAATGGACAGCCCAAAGATCAGCCATCTACCTGCTAAGCAACGCAGGCATCAACTCAAAGCTCTGCGGGGGGTTTGACACGGGCTGAACAGTTTCTGACCGATGGCAAGAACGTGGGCTCGCTCAGTAGCCATTTTAAGCAACGGATGCATTCACCACGGGAGCGCAGCGACAAAAGAACCGGGGGCAAAAACACGGGGCAGTAACACGGACCCCTAAAAGCTCAGCAACACACGCACGGCTCGATCGCAGCAGCAGCAGCAGCACGCGTGAAATGCACTGAAAGAAACCAACAAAGAAAGAGAGATGATTAAACGACCTTCAGAGCACAGCAAATCCCTCAGAGCAATGAGTGCCGGGCACAAGAAATGTCCTGGTTTCCAGTCAGTTGTTTCATATGTTTTCCAGGGAGTTCTCGTGCTGTGGGAAAGATTTCTGCGTGCATTGAAAGTAAGCCTGCAACATCCCCTTCCACTCCCACAGCAAGTATGCAATCCATAAAAGATTAAAGTGCTGCAGACATATGCTTCGGCTAAGACTCTCCTTTTTTCTTCTTTTAATCATATCAACAGTTTAGCACTTGCTGCATTTTATCAAGCTATTTTGCCATCCCTTTTTAAGGCAGCAAGGGGAAGAGATGGAGTGCAAGTGCGTGAGCCGCAGGCAGCCTGTAACTCAAACGCTGCTTTACAGATGTATTTACTACACAACTTGTCTTTGGGGGCTTTTCAGGCCAGGGCAGCCTTTGTCTCTGCACACTATGCACTTGCCAGCTCCTAACAAATGAGCAATCACATGGAGAGCAGATCTGCATCCTTCCGTGGGGTCCACCGGGAAACTGAGACATGGAGATGTGCCTG

Novel00931

CCCCATAGCCCTGCCTGCCCCATAGATCCCTGCGCTGCCCCATAGAGCCCTGCGCTGCTGCTCCATAGCTGTGCGCTGCCACATAGTGATGCGCTGCCCCATAGATCCCTGTGGTGCCACGTCACCGGTGCGTGCCCCACACATCCCTGCTGTGCCCCACATATCCCTGTGCTGCCCCATAGATGCCTGCTCTGCCCCATAGAGCCCTGCTCTGCTGCTCCATAGCTGTGCGCTGCCCCATAGATCCCTGTGGTGCCACGTCACCGGTGCGTGCCCCATAGATCCCTGCTGTGCCCCACATATCCCTGCGCTGCCCCATAGAGCCCTGCGCTGCCCCATAGATCCCTGTGCTGCTGCGCCATAGCTGTGCGCTGCCCCATAGATCCCTGTGGTGCCACGTCACCGGTGCGTGCCCCATAGCCATGCCTGCCCCATAGCCATGCTCTGCCCCATAGAGCCCTGCTCTGCCCCATAGATCCCTGCGCTGCTGCTCCATAGATGTGCGCTGCCTCATAGCCATGTGCTGCCACATACATCCCTGCGCTGCCCCATAGATCCCTGCTCTGCCCCATAGCCATGCTCTGCCCCATAGCTGTGCGCTGCCCCATACATCCCTGTGCTGCTGCGCCATAGCTGTGCGCTGCCCCATAGATCCCTGTGCTGCCACATAGATCCCTGCCCTGCCCCATAGATCCCTCTGCTGCCCCATAGATCCCTGCTCTGCCCCATAGCTGTGCTCTGCCCCATAGCTGTGCGCTTCCCCATAGATCCCTGTGCTGCCCCATAGATCCCTGTGCTGCCCTATAGCTGTGCGCTGCCCCATAGATCCCTGTGGTGCCACGTCACCGGTGCGTGCCCCATAGCCATGCACTGCCCCATAGATCCCTGCACTGCTGCTGCCCCATAGCCATGCTCTGCCCCATACATCCCTGTGCTGCCCCATAGATCCCTGCGCTGCTGCTGCCCCATAGATCCCTGCACTGCTGCCCCATAGATCCCTGTGCTGCCCCATAGATCCCTGCGCTGCCCTATAGCCATGCGCTGCCCCATAGCCATGCACTGCTGCTGCCCCATAGATCCCTGTGGTGCCACGTGACCGGTGTGTTCCCCATAGATCCCTGCTCTGCCCCATAGCTGTGCGCTGCCCCATAGCCCTGCGCTGCTACTGCCCCATAGCCATGTGCTGCCCCATAGCCATGCGCTGCCACATAGCCGTGCTCTGCCCCATAGATCCCTGTGCTGCCACATAGCTGTGCGCTGCCCCATAGATCCCTGCTCTGCTGCCCCATAGATCCCTGCTCTGCCCCATAGTCCTGCTTTGCTGCTGCCCCATAGATCCCTGTGCTGCCCCATAGATCCCTATGCTACCCCATAGCCATGCGCTGCCCCATAGCCGAGTGCTGCTGCCCCATACATCCCTGTCCTGCCCCATAGATCCCTGTGCTGCTGCCCCATAGCGATGCACTGCTGCCCCATAGCATTGCTGTGCTGCTGCCCCATAGTGATTCTGCCCCATAGCAGTGCTGTGCTGCTGCCCCATAGCGATGCTGCCCCATAGCGATGCTGCCCCATAGCACCACTGGTCCACCGTACTGCTGCCCCATAGCGCTCCCACCCCATAGATCCCTGTGCTGCCCCATAGCACCGCTGCCCCATAGAGTTACTGCCCCATAGAGCTAGCGCCCCATAGATCTCTGCACTGCCCCATAGATCCCTGCGCTGCCCCACAGTGCCCTACAGCACTGCCCCATAGCATCTGTGTGCTGCCCCATAGATTAAAGCTCTGCCCCATAGGATCACTGTGCTGCCCCACAGATTCCCGCACTACCCCACAGATCCCTGTGCCGCCCCATAGCGCTGCCCTATAGATCCTTGCACTGCCCCATATTGTCTGACTGCTGCCCCATAGCATCGCTGCGCTGCCCCATAGCGCCCCATAGAGCTGCCCCATAACTCCCTGTGCTGCCCCACAGAGCTGCCCCATAACTCCCTGTGCTGCCCCATAGCACTGCCCCACTGCATCTCTGTGCTGCCCCACAGCGCTGCCCCATAGAGCAGCCCCATAACTCCCCGTGCTGCCCCATAGCGCCCCATAGCGCTGCCCCATAACTCCCCATGCTGCCCCACAGTGCTGCCCCATTGCATCTCTGTGCTGCCCCATAGTGCTGCCCCATAACTCCCTGTGCTGCCCCATAGTGCTGCCCCATAACTCCCTGCTGTGCTGCCCCACAGCGCTGCCCCATAACTCCCCGTGCTGCCCCATAGCACCCCATAGAGCTGCCCCATTGCATCCCTGTGCTGCCCCACAGCACTGCCCCATAACTCCCTGCTGCGCTGCCCCATAGCGCCCCATAGAGCTGCCCCATTGCATTTCTGTGCTGCCCCATAGAGCTGCCCCATAACTCCCTGCTGTGCTGCCCCATAGCGCTGCCCCATAACTCCCCGAGCTGCCCCATAGCGCCCCAAAGCACTGCCCCATAACTCCCTGTGCTGCCCCACAGCACTGCCCCATAACTCCCTGTGCTGCCCCATAGTGCTGCCCCATTGCATCCCTGTGCTGCCCCACAGCGCTGCCCCATAACTCCCTGTGCTGCCCCATAGAGCTGCCCCATAACTCCCTGCTGTGCTGCCCCATAGAGCTGCCCCATAACTCCCTGCGCTGCCCCATAGCGCCCCATCGAGCTGCCCCATAACTCCCTGTGCTGCCCCACAGCGCTGCCCCATAAGTCCCTGTGCTGCCCTGCAGCGCTGCCCCATAACTCCCTGTGCTGCCCCATAGCGATGGGCATCACTGCCCCATAGCGCCCAGTGCTGCCCCATAGCGCTGCCCCATTGCATCCCTGTGCTGCCCCGCAGCGCTGCCCCATAACTCCCTGTGCTGCCCCATAGCGCCCCATAGAACTGCCCCATAACTCCCTGTGCTGTCCCACAGCGCTGCCCCGTAACTCCCTGTGCTGCCCCATAGCGCCCCTCAGAGCTGCCCCATAACTCCCTGTACTGCCCCACAGCGCTGCCCCATTGCATCCCTGTGCTGCCCCGCAGCGCTGCCCCATAACTCCCTGCGCTGCCCCACAGCACTGCCCCACAGCAGCTGCCCCATAACTCCCTGTGCTGCCCCATAGCGCC

Novel00841

GCCCCATAGCTCTTCCATAGCTGCCCCATAGCACCCTTTAGCGTCACATTGTGCCCCATAGCTGCCCCATAGCACCCCATAGCTGCCCCATAGCGCCCCATAGAGACCCATAGTGTCCCATAGAGCCCCATAGTGCCCCATAGCACCCCATAGTGCCCCATAGCGCCCCACAGTGTCGCATTGTGCCCCATAGCGCCCCATAGCTGCCCCATAGCGTCACACTGTGCCCCATAGCTGCCCCATAGGGATCCATAGAGACCCATTGTGTCCCATAGAGCCCCATAGCGCCCCATAGTACCCCATAGCGCCCCATAGAGACCCATTGTGCCCCATAGCATCACACTGTGCCCTATAGCTGCCCCATATCTGCCCCATAGCTGCTCCATAGTGCCCCATAGCGCCCCATAGCGTCACACTGTGCCCCATAGCTGCCCCATAGCACCCAATAGTGTCACATTGTGCCCCATAGCTGCCCTATAGCGCCCCATAGAGACCCATTGTGTCCCTTAGAGCCCCATAGCTGCCCCATAGCGCCCCATAGAGACCCATTGTGTCCCATAGAGCCCCATAGCACCCCATAGCTGCCCTATAGCTGCCCCATAGTGCCCCATAGCTGCCCTATAGCAACCCGTAGAGACCCATAGTGACCCATAGCAACCCATAGCTGCCCCATAGCGCCCCATAGAGACCCATAGCTGCCCTATAGCTGCCCTATAGTACCCCATAGCGCCCTATAGTGACCCATAGCAACCCATAGCAACCCTTAGAGACCCATAGTGCCCCATAGCTGCCCCATAGCTGCCCTATAGCTGCCCCATAGTGACCTGTAGAGACCCATAGTGTCCCATGGCTGTCCTATAGCTGCCCTATAGTGCCCCATAGCTGCCCCATAGCGCCCCATAGAGACCCATAGCTGCCCTATAGTACCCCATAGCGCCCTATAGTGACCCATAGCAACCCGTAGAGATCCATAGTGTCCCATAGCTGCCCTATAGTGCCCCATAGCGCCCTATAGTGACCCATAGCAACCTATAGAGACCCATAGTGCCCCATAGCTGCCCTATAGCTGCCCCATAGTGACCTGTAGAGACCCATAGTGTCCCATAGCTGCCCTATAGTGCCCCATAGCTGCCCCATAGCGACCCATAGTCTCCCATAGCTGCCCTACAGTGCCCCATAGCGCCCTATAGCGACCCATAGCAACCCATAGCAACCCTTAGAGACCCATAGTGTCCCATAGCTGCCCTATAGTGTCCCATAGCTGCCCCATAGCGACCCATAGTCTCCCATAGCTGCTCTATAGTGCCCCATAGCGCCCTATAGCGACCCGTAGCTCTCCCATAGCGCCCCATAGAGACCCGTAGTGTCCCATAGCTGTCCTATAGCTGCCCCATAGTGTCCCATAGCTGCCCTATAGTGACCCATAGCAACCCATAGAGACCCATAGTGTCCCATAGCTGCCCTATAGTGCCCCATAGCGCCCCATAGCGACCCATAGTCTCCCATAGCTGCCCCATAGTGCCCCATAGCTGCCCCATAGCGACCCATAGTCTCCCATAGCTGCCCTATAGTGCCCCATAGTGCCCTATAGCAACCCATAGCTCTCCCATAGCGCCCCATAGAGACCCGTAGTGTCCCATAGCTGCCCTATAGCTGCCCCATAGCGCCGCCCCACATATGTGGGGTGACGCCCGGCTGCCCCATTGCAGGGGGTTCTTCTCGTGCCTCAGTGAGGACGGCAGCGTCTACGATGACACCAAATACGTGTGGATGCAGGGCCGGCAGGTGGGGCGGATGTGGGGCAGCTGTGGGGCAGGGGGGGCAGATGGGGGCAGCTGTGGGGCAGCTGTGGGGCAGGGGGGGTAGATGTGGGGCAGCTTTGGGGCAGCTGTGGGCCAGCAGGTGGGACGGATGTGGGGCAGCTGTGGGGCTGTGCTATGGGGCAGCAGTGGGGCAGCAGTGGGGCTGTGCAATGGGGCAGCAGTGGGGCAGCAGTGGGGCTGTGCTATGGGGCAGCAGTGGGGCAGCAGTGGGGCTGTGCTATGGGGCAGCAGTGGGGCAGCAGTGGGGCAGCAGTGGGGCAGGCATTGTACCCGCAGGTGTGGATGTACTCCCGGCTGTACCGCACTGTGCCGCGTTTCCGGCGCCCGGAGCTGCTGGAAGCCGCTATCAAAGGCGGGGAATTCCTTCTGCGCTGCGCCCCATTGGACGCGGAGTCTCCGCGCGTCGCCTTCGCTCTGAGCCGCGACGGCCGCCCGGCGCAGCTGCAGCGCAACGCCCACAGTGAGGCCTTCTGCGCTATGGGGCTGCACGAACTGAGCCGCGCCGCCGCACAGCCGCGATATGCGGAGGCGGCGCAGCGGTGGGCTTTGGCGGTGTGGCGTTGGGCGCGGGACCCCCCCCCGGCTTTGGGGCGGCCGCAATTAATGGGGACCCCCCCCAAAGAGGAGTTGGCGGTGCCGCTGATGGCGGTGGTGGTGGCGCAGCAGATGAACGACGGGGGGGGGGGGGGGGGGCGCAGATTGGGGGGGGGGACCCCACAACGGGGGGGGTCCCACAACGATGGGGGGGGGCGCCGCAACGCCGTGACCCCCAAATGGAGCTCATGGAGGAGTGGGGGGCGCGAAGGCTGCTGGCGCACCTGCAGGAGGGGGGACACGGCGGTGCTGGAGAACATCGGCCCCAATGATGCGGAGCTGCCGGGCGCTGTGGGGCGGCTGCAGAACCCAGGGCACGCCCTGGAAGCGGGTTGGCTGCTGCTGCGCTACGCCCAATCCCGCGGCGATCCCTCATTGGCTGCCCGCGTCCTGAAGGGCTTCGTGGACGGACCCCTCGAGTGGGGGTGGGACCCCCGATATGGGGGGGTTGTGGCCTTCTGCGACGTCGATGGGCACTGCCCCACTCAGTTGGAGGCGCGGATGAAGCTGTGGTGGCCGCAGTTGGAGGCGATGGTGGCGCTGCTGACGGCCTTCGCCCACACGCGGCGCCCGCAGTACCTGCGCCGCTTCCACCGCGTGGCGCAGTACGCATGGAGCAGG

Novel00803

GTGAGCCTGATCACATGACTTAAATCCCTTGATTTCCATGATGTATCTTCCTTCTCTGAACGTGAGGTCCTCTGGGATACGTAGTTCTTCTTCTGTTTCGAGAAGCAGGCATCTGGAAGATTCACCTTTGGAACCAAGGTGGGAAGGACCCTACCAAGTACTTCTGACTACCCACAAAGCAGTGAAAGCAGGACTGACACCGTGGATACATCACAGCTGCCTGAAGAAAGCACCCAGACCACAATGGACAGCAGAGGAGGAAGGAGCGATGAAAATCAGGATTCGAAAGCATGTATAAGAGTTTGATGACAGTCAGTGTGATTGTAGTAGCAGTCACAGCTTGGCAAGGAAATAGTTACTTGAGAATGACTGAGAAAAATTGGCCACGCTCTCAAAAGGTCTTGCTGGATATGTACGGCCCTCCCTAGAGGAGAGGGGAGAATGCCAGTTTATGGGATAGTAGCACTAAATTGGACAATTCCAAATTGGGTGGAACATGGGAAGTGTAAATTTGGGTTAGAAGATAAGCCACAGAAGGGCACTAAGTTTGATTTACTAATTATTAACTCTGCTAGGCCTATCTTTGTAGACAGGAAAAACAATAGTAAAGGTGGGATGGGGGTCAGATGGCGTAACTTGACAGGCATAGGGTGTAATTGGGAATACGGAAACTCTGGGGCAGTATCAGAGGTCAGTGAAGCGTCACAATGGGTCTCGGATAAAAACATACTCGGAATTGTAGGAATGGGAACATCATTCGCTCACCAAGAAAGTATGACCAGGCCATGTAAATTACCTCATGGATACTGGTGGTTATGTGGGGATGGACAGGCATGAAAAACATTACCTTTAAATTGGACAGGAACATGCACTGCACGATACTTGGTACCCCAGTCTACGGTGCATGAAGAGACCCCTCGGGGACTCTTGAGAACCCCCTGGATTCAAACTAAGCACTCGTACAACCCCCGTAGTTAGGTATAATACAGGATACCACAGCTTACTGAGAGCCCTTATCCCAGCTCTAGGAGTAGCTCAGATGGAAAAGGCTGCAGTAACAGAGAATTGAAACAGACTTAATGAAAATTTGGGAGCAAACAAAAATCCTGCATAGGACAAGTCAGGATAACACCAACTGGGAAGTAGATTTATGGGGCTTACTTACTAGCTGTTTACCGAATCTAGGATGGTTAAAACAGCTTTTCTTGGCCTGCATCATCTTGGTGCTGGTATGTTTTACCTGTGTGGTAGTGAGGTGCTCTATGTGCCCGTGCCAGAATACTAGGAAGGAGTATGACGTATGGAAGAGACATGAGTTGAGGCAAACGGTGGAGAGTGGGGCTTATTTTGGGACACGTTAAGAAAGAATAAAATAATCTAGCAGGTAGAAGTCCTGCTAGATTAAAGAAAAGGAGGCAATTGAAGAATGAGAACAGGACCCTGTAGTAAAAGAGAGAAGGGCCCCACAGCAAGAAAAGGGAAAGTAGCTTCACATTTTTTCCACAAGTAGAAATAAGGGGCAAATATAGATAGTTTAGGTAGAGTGTTAGAAAACAAGGGATTCCAAGCACTTTCAGAGACACTAGGTCTGGAATAACAAGGGGGATGAAGGCCATAAAGATAAGGACTGGAGAACAGCTCAAAAACATCTGGCATGGAGGTGATTAGATGTCCGCAGGGCAAGCTGAAACCGGTTTGGGGTCAGAAGTTTGCAGTGAGGAAGACTACGAGCCTTCGCAAGGAAGACTACAAGCCTTCATCATCATGACCTACTATGCATGCGCAAGGGAGGGAGGGACTACATGCTAATGGGCTCTCAGGAATGTAATGAATATGTATCCGAATTTCTGTCAACTATTGATTATGTATTAGTTCAGCCTATATCTGTACCATGCTTTTTCCTGACGGTGTGCAAGTTAGGTGGAGTGATCCCCCTTGCACCAGGGCGTATGCACTCAATTCCATGATTGATGTCATTGATAAAGATGTTAAAGAGCACCCGGCCCAAGACAGACCCCTGGGGGATACCGCTCATTACCGGCCTCCACCTGGACTTAGAACCATTAATCAACACCCTTTGTGTGCGGTCTTGTCCTGTCACAGAGTTATCTAGATCAATCTAGATAAATCTATGCCTGTGACAGGGGGCAGTAGTCCCGTGGGCCCCCTGGCTCCCAAACATGGGAAGGGAAAAGGGAAAGGGGTAGGGAAACGGGAGCTGGGCTCAGAGAGGGAAAAAAGAACAGAGCAGCAGCAACGACCTAAGGAGGAAAACTTATTTTACTAACTATGATATCGGAATGCAAGATAACACAATATAATACAATCTAATTGAAAGTAAGGGTAATAAATCAAATAAAATAAGAGAGAGAAAGTTTCCAAAACCGAAAGGCCTACTTGAATGAAGGCGTATTGTAGGAAATCAGCGGGAGAAATGCGACGACCATAGTAGATGATCAGCAAATCTCGTTTATTGTTTCAGCTGCGTGTACGTTTATAGTGTGGATAATAGGCTCATACATATTGCTAAAGTGGAGCTCAGGATTGGCTCGCTAGTCAGTGTCAACTACGCCTATCTTTGTGCTCTGTGGTTATCTCTCAGCAAACACTCTCTTACAGACTTCTCCAAACACTGTTGCCAGCTTTCTCCATCGGCATCCTGTTTTGTTCCTTGTTCTCATACCTGACCTTGCGCGTAACAAGGTTGCTTAACCAGCTGCACAGTGTCTGCATGGCTTTTCTCAGTCAGCCAGTCATCAATAAATCTCTCCACAGCGTATGGCTTGGAAGCCATCTCTTCTGGCCAACGTAACTGTAGAATATCTTCTTATTGTTTTTAACAACCCTTGCCAAGTTCAGTTCCGCCTGTACCTTGGTGTGGAGAGGGCGATGGCAAAACTATAAATATGGGGAGGCGTGGCAGATTGATATATCACCTTTCCATGATTTTGCAATGGTAAGCGTTGTCACGGAGTTATCAAGATAATTCTGTGCCTGTGACAGGGGGGCAATACGACACAATCCATCTGTTTCATTTTTCTGGTAGTTCATTGTATGGTGGGGCCTCTTTAGCACGTGATACCTCTTCTCCTGGTGATGCTCCAAGCTTGTTGCCTTCAGGCCAGTCCATGATCACCTCTAAG

Novel00751

CTAATAGCAGCCACAGATGCAGTGGTGGAAGCGTTTAATAAACTTGTGCATAAGGCCGAACCACCTACTCCGTGCACAGATATTACCCAGGGCCCGAATGAATCCTTTCAAAGCTTTACAGACAGGCTTTTAGCTGCTGCAGAGGGGTCTGATCTCCTGGTACCGGCCCAAGGGCCAGTGATCATTGACTGCTTGCAGCAGAAATCGCATGACAATGTTAAGGCATTGCTGCGAGCCGGCCGAAGTACGCTTAATACCTCGGGAGAAGTTATTCAGTATGTCTTAGATAAGCTCAAGGTGGCTCATTTAACTAATGAAAGGCTAGTCACAGCTATTGTCGTAGCTGTTGGCCTGCGACAGCAAAGATCGCTGCAGCAGCAAGGGCTGTGTTTCCGATACGGCCAGTATGGCCATGTTAGAGCACAGTGCCCCTGTGGGGGAGGCCAGTCAGGGCCTCCTGATCACAGGAAGGGCTTGCTAAAGGGTATCCGTGGTCGGGTATGCAGCAGTTAAATATCATGAATCTGGGAAATTCTGTGGGTACCATCGCGAAAAGTAATACCAGATATAAGTAGCATATCTGCAAAAACTGAGTCTAGGTGCGAGTTTTCTACAGAAATTCTTCGTTGGCAGACCGAAGCCAACTCCAGAACAACTGAGGGACAGACGATGGGATCAAGAACCACTCGTCAAGAGGAGCACTACACCTGCAACGCTGACACTTCCGATGTTGCTGATATTTATGGTGTCCATGGCGATTACAGAGTGTCACAAACAACTACAATGGACACAAGAACATATGCAGAAAATTAAAGAAGAGCGTGATCCCTTTGGAAGCTGGCTGGACGGACTGTTTGGGGGAACGGGTTCATGGTTAAAGCAATTGCTTAAAGCTCTCGCAGTAGGATTTGCAATCTTTGTGTGTATTCTAATCTGTCTTCCATGCTTTGTAGGATGCTTGCAGAACTGCCTTCAACGAATGATGGACAAGACTTTTGACTATCACATTGAGTATCATAGATTGCGTGAAAAATTATAGAGGGGTTTAGGTTGTTGCGTTCGTGCTGTAACGGGGCAAGGCTTGGCCGAGCACGGAAAAGAATTCCCTGTTGCTCTGATGATTGCTTAAGAACTGTAGTAGAAAATAGTAGGAATAGTGTGCTGAAATATATTTAGGATTAGGCGTTTTGCGCTGCTTCGCGATGTACGGGTTAGGTGTGTGTGTAAGTAGTATTTAGCTTAGGGAGGGGGAGATGTTGTAGTAGGCGTCTTGCGGGGGCACGGGATGTACGGGACAGGCCTCTCCCTAAACATAGAGAGACAGTGCTATCGTGCTGACCTTGTTGCAGAGAAAACAGGAGAAGAAGAAGGATGATAAAAGAATGTGGAAACGGCCAAATAAGGCACAATGTTATCTGGTGTGAACCAATCAGAGTGGGACATGACAGCACGGTTATCTAGGTAAAAATGTATATAAGCTGTGTTTAGTAGTGAATAAACGCCATTTTGCCTCACTTACTTCTGGGGTCTGCGTGAGCA

Novel00727

CCGAGCTAGAAATAATTCACCCTTGTTCTGCCAGTCCAAGTCTATTTGAGCCACCTCAATTTTGGCAGCCTGATCTACCTGATGGTTATTTTTCTGTTCTTCCGTAGCCCGACTCTTGGGCACATGAGCATCTACGTGACGCACCTTTACAACCATATCTTTATTCGGGCAGCAATGTCTTTCCACAGTTCAGCAGACCAAATAGGTTTACCTCTCCGTTGCCAGTTATTTTTGCTCCCACTGCTGTAACCACCCCCATAAGGCATTCGCCACCATCCATGAGTCAGTATAAAGATAAAGCACTAGCCACCCCTCCCGTTCAGCGACATCTAAGGCTAGTTGGACAGCCTCGACCTCTGCAAACTGACTTGATTCTCCTTTTCCTTCAGTGGCCTCTGCAACTTGTCGCACGGGCTCCACACAGCAGCTTTCCATCTGCAATACTTCCCAACAATACGACACAATCCATCTGTGAACACGGCATATTTCTTTTCATTTCCTGGTAGTTCATTGTATGTTGGGGCCTCTTTGACACGTGATACCTCTTCTCCTGGTGATGTTTCCAACTTTTTACCTTCAGGCCAGTCCATGATCACCTCTAAGATTCCTGGACGACTGAAGTTCCCCATTCAAGCTCGTTGTGTAATCAGTGCAATCCACTTGCTCCAAGTGGCATCAGTAGCATGATGGGTGGAGGGAATCTTTCCTTTAAACATTCAGTTCAGCACTGGCAGTCGAGGTGCCAGGAGGAGCTGCGTCTCAGTACCGACTACTTCGGAAGCAGCCCTAACCCCCTCATACGCGGCTAAGATTTCCTTCTCAGTTGGGGTGTAGCACTCTTCAGACCCCCTGTATGCTCGACTCCAGCATCCCAGGGGTCGGCCTCGGGTCTCCCCTGAGGTTCTTTGCCACAAGCTCCAGGTGGGACCTTTCTCTCCAGCAGCAGTGTAGAGGATGTTCTTTACATCCTGTCCCGTCCGTACTGGCCCCAGAGCCACGCACGGGCTATCTCCTGTTTGATCTGCTCAAAAGCCTGCTGCTGTTCAGGACCCCATGTAAAATCATTCTTCTTCCGCGTCACCTGATACAGGGGGCTTACAATAAGGCTATAGTTTGGAACATGCATCCTCCAAAAGCCCACTACACCCAGAAAAGATTGTGTCTCTTTCTTATTAATGGGTGGAGACATGGCAGGGATTTTGTCAATCACATCTGCTGGGATGTGACGGCGCCCATCTTGCCACTTTATACCTGGGAACTGAATCTCCTGGGCAGGTCCTTTCACTTTGCTTCGCTTAATAGCAAAACCAGCTTGCAGAAGAATTTGGATTATTTGCTCTCCTTTCTCAAAAACATCCTCTGCTGTATCGCCCCACACAACAATATCATCAATGTATTGCAGATGCTCAGGAGCACTGCCCTGTTCCAATACTGTTTGGATTAACCCATGGCAAATGGTTGGGCTGTGTTTCCACCCCTGGGGCAAACGGTTCCAAGTATACTGAATGCCCCTCCAGGTGAAGGCAAACTGTGGCCTGCACTCTGTGGCCAGAGGAATGGAAAAGAAGGCATTAGCAATGTCAGTGGTGGTATACCACTTGGCTGCTTTTGACTCCAATTCATATTGGAGTTCTAGCATGTCCGGCACAGCAGCACTCAGCGGGGTGTGACTTCATTCAGGCCGCGGTAGTCTACCGTCAGCCTCCATTCTCCACTGGCTTTACGCACTGGCCATATGGGCTGTTAAAAGGTGAATGGGTTCTGCTGATCACTCCTTGACTTTCTAGTTGACGAATCAACTTAAGAATGGGAGCAAGGAATCCCTGTTGGTTCGATACTGCCGTCTATGCACTCCTTATGTGGCAATTGGTACCTGCTGCTCTTTTACTTGCAGCAACCCCACAGCAGACGGATCTTCTGACAGGCCAGGCAAAACAGATAGCTGCTTAATGTTGTCTGTATCTACAGCAGCTATTCCGAAGGCCCATCGATACCCCTTAGGATCCTTGAAATGCCCCCTTCTGAGGTAATCATTACTCAGTATGCATGGAGCCCCTGGCCAGTCACAATAGGGTGCTTTTGCCAGTCTTTACCAGTGAGGCTTATTTTGGCCTCCAACACAGTCAGTTCTTGAGAACCCCCAGTCACTCCAGAAATATGGACTGATTCTGTCCCTTCATGATTCGAGGGCATTAGAGTGCACTGTGCACCGGTATCCACCAATGCCTTATATTTCTGTGGTTCTGATGTGCCAGGCCGTCGGATCCACACAGTCCAATAAACTCTGTTATCCCTCTCCCTCCCCTGACTGAGGGCAGGGCCCCTCTATTCATTACCTGTGGTAACTGGAGCAACTACACTGGTGGTTGGTCTGTTCTGTAATTCTCTCACCCGTGCTCGCAGTCCGGGAGTATCTTGATCGTGCCACTTCCTCATGTCTTCTCCATGGTCCCGGAGATAACGCCACAAGGCAACACGCGAAGCAGTCCTGTTGTTGTTTCTCACTCGAGCAGGAAAGTGTTTGCCTTCAATAGCTGAAATTTTTGATGTTACAGGTGAGGAGGGGGATCCATCTTCTTTAATTAATTGGATTTTCATTAGTTTGATAAGCTCTTTCATTAGGCCTCTTTGCTTATCTTGATCGTCGTCAAACGTTTCCGATACTATTACGGGTTCTACGGGTTCCTCACGATTAATCAATAGGGACATCTGCTCTTCTACTTCACCCAGTCTGTTTGCTAACTCTGTGACGGCTGAAATGGAAACGTGGGTTGGGGGTAGATGTTGCTCATAGTTTTGGAGTGTAAGAATCAATTCGAAGACTAGGGGTCTTCTCTGTTGGTCCTCGTATCTGTGGAATGTGGCTACTAGTGTGGGGGCGTATCTGTCTGGTGCTGCTCTCCTAAGTTTTTGCCATATTTCAGGTGTTGTCCTCACTCTCTCCGGATCATGGTTTTGGCGTGGATCATTAGGAGCAAAATTAGACTCATATAACATTTCCACCAGGGCTATTTCCCTCAAATACTGGATACCTTTCTCAATTGTATTCCACGTTTTCTTTGCAGGCTGCAGAAGTTCTTTGTAGGGATACCTTTCCTTCACAGCTATTAATAGTCGGTCCCAGAGGGTTGCAGCTTCATCCAAACATCTACTAATTGCTCTGTCGATGGATTTGTCTCCGGCAACGTCCCCTAGTTGGCGGGCTTCATTACCATCTAGAGACAAGCTACTGGCCCCACTATCCCAACAACGAAGCAACCAGGTGACAACAGTTTCATTCGGGCGTCGTTCAAATTCCTTTCTTGAGCTTAGGATCTCAGACATCTTGAGAGGTCGACGAACAGTAATAGAGATCTCCTCTTCATCGCTGTCCTCTTCATGCCTCTTAGTTTTTGCTTTTGCTTTTCTTGGTTCTGAGGAAGTCCCCTCACCTGGATCTTCCTCTACCTCCTCCTCCTCCACTTCTACTTCTTCTTCTTCTTCCTTTTTTCACTCTAGACGCGAGGACACCCGCTTCCATTTCTTGCCTTCTACAGGGGCTACTGACACTGTTACTGGTGTCTGTTGTGACTGATCAGGTCTGACTTGGAGATTTCCCCCTTCGGCTTGCACCTCCGCTCGGAAACTGTCTCTCTGCAGAACAGTACTATACAGGGCGCGGTAGGCACAAGCCAAGCCCCAGATGAGCCGTGCCTCCTTTGACCTATCTAAGCCGCACTGCCCCTGACTCAAATGCTGGCTCAGTTTCTCGGGATCCCACAGATGTTCAGGAGTGAAATCCCATGGCATGGCGGGTCCCCACGGTTCTAAGATTCTTCCTAAACCTTTCCATATTCCTTGCCAGTCATCGTTCTCTGGTCTTGGAGGAGGCTCCTTCCACTTAGCACGAATGAAGAGGAATACATTCAAAGATATCGATAACATGCACAAAAATATGAGCACTGACTCGGTATTCGAAAAACGTTCAACAAACTGAGAGGGAAGCCTAGAAACTGGTTCGAAGGTCTCAAAATTCAAATTATACCACATTGCATAAAGCCACCAGGAAGGTGCCTCTATCAGGGCCCTTACTTGGTTGTATATGAACACAACTCCACAGCAAGACATGAAGAGGTTAATTATAGACCGGTAACTGGCAAGGAACATGATAGCTTTTAATCCCTTATACAGTGCTCTGACAATAAAGAGCAACCTCATAGTTGATAACTGCTAACGTAAGGGGGGGGGGGGGTGAAGAGAAGGAGAAAAAAGAAAACAAACGGAAACCACTCGGTACAGTGTCTGGAGTTTGGCGGACTGCCCAGGCTATAATCTAATCTATGAAGGTCACACCAACACCAGCAATGCTATGTAGTCTCACAGTCTGGCCTAACGAAATCCTTTTTTTTTTTACTAACGTTAAAGCTTCAGATGAATCTTGCCCGCATTCTCCACCAAAAATTTGTCACGGAGTTACCTAGATAGATCTGCACCCGTGACAGGGGGGCAGTAGCCTTGCGGGCCCCCCACCCCCCTGGCTTCCCGAGAAAAATGAAGCGGCGGCAACAATCTAAGAAGGAGAACTTAGTTTACTAACTATGATGTTGGGATGCAAGATGACACGATACAATACAATCTAATTGGGAGTAAGGATAATAAACCAAATGAAATGAGGGAGAGAGCGAAAGAGCGATAGAGAGGGAGAGAGAGAGAGAGTGAGAGAGTTTCCGAAACCGAAAGCTTTACTTGATGAAGGCGCCCGGCTTGGAAAGCACACCCACTCCTCTCCCGGCAGCAAGCGAAAGCGAAGAAGAACCGCGCGCAACAAGATGACGTATCTTCTGTGATGTGTCTTCCTTCTCTGACCGTGAGGTCCTCTGGGATACGTAGTTCTTCTCTTTTGTCCATGATGTTATGATGAGGAATACCAGTTGCGAAGTTACAAAACCATGCCACCAAGCGCTTCTGCGTCACAGGCCCGCCTCTACACACGCGCACACCACAACCACACGGTGGCGCTGGGAAGCCAGGCGAGCCAGACGGCACCAGCGACCCAGCATGCGCCACGGCAGCCCCGTGGCACCGAAGGAGTGCGGGTTTTAGAGGAGGCGCTGTAGTAGCGAGCATCACCAGCCAGAAAGGCCCGTCCGCCCGCCCCGCCCCGCGGCAAGGTCGCCGCTCGCCCCGCCGCACTCGCCGGCACCGCAACGGGCGCTCGTTCCAGAGGGCCTGCGAGCGCGCTAGCGTGGGGGACGGGTGGGACGGGAGGGCAAGGGAAGAATCGCGCGACGCACGACAAAGCCGCGGCTACCTCCTCGTCCACAACGGCTCCTCCTCGCGGATAACGTTGGCGGAGAACTCCTGGCGGGCGACTTTTCCCAAGAGAGCGGCGCCACCGCGCCAGGCGGCCGGCGACCTAACGATCCCGCCGGCCATGACGGCACCCGCTCGCTACAACACTCCCTCAGCCCCAAACCTCCCCCGCTCGGCTCGGCTCGGCTCCGCTCGGCTCGGCTCGGCTCGGCCCTCGTCGGCGCTCATTGGGCCGACAGAGCCGTGCCGGCCGTTCTCGCGCCTCGGTTGGCTGTCTCGCCTGCCCTTTAAGCTTGTCCCCGCCCTGTAGGCGGCTCCGCTCCCGTCGGCCCGGTGCTTATCGGGGCTCAGGGACTTAGGCGCTGGGGGCTTTTTGGTGCCGATCCCTCCCGTCAAATGACCGTCAAATGTTGGCGGAGCAGGCCAGGAGTTTGCCATCTTTGGATGAAGGGACGGGCAACTCGGGGAGAGTGCCAGGATGTTGCTAGCATGCGCAGGGAGAAAATTCGACAAGCCAAAGCCCAGCAAGACCTTAGTCTGGCCGCCATTGTTCGAGATGATTAAAACAATGTTTTTACAAACATATTAATAAGAGCAAGAGGAGGGCCAAGGAGAATCTCCCTTCTTTATTCGACGCGGTGGGGAACATCACCACCGAGGAG

Novel00676

CCAGGTTATTCCCACAGCTCTTATTCTGGAAACCAGCAGGGAGGGTCTTCCTACTCTCCTGGTGGATCTTATGGACAGGGAGGCCCAGGTTATTCCCACAGCTCTTATTCTGGAAACCAGGGAGGGTCTTCCTACTCTCCTGGTGGATCTTATGGACAGCGAGGGTCTTCTGTCAATGATAACGGCAGCGAATCCTATGGTCAGGATGGATCTTCCGCCTATGGTGGCACTGGATCATATGGTCAGGATTCACCTTCCATGGGAGTTAGTGAATCTGATTACAGTCTTTACTTATCACTACTGCAGAAACTAGTTCCCTCTCCTTCCATCTTAAATTGTATGTACTTTCTCTGTGTTTTTTTCCAAGTAAGATTTAAGTTCCTTCACATTGCATGTGAATGGTGTGATTTGAAGTATTGTGGTGAGAACTGTTTTCGTTCTGCTTACTTCCTTCAGAAAAAAAGGCATTTTCTTATAAATTCCAATAATTCTGGTGAAGGTGTTTGTAAAACTTTCTGTGTTTAATTTCTAATTTTCATGACGCCTTAAGTAATTAATTGTATTTTAACTGTATTTCTAATATGTCTGTTTTCCAGTGTTCTAGTTAGTAGAAAATGGTTCTTCACAGGTTTTTATAAACTCAGCTGTGCATTACAGTCGTAATTATTAATTCCACAACAGTTTCTTTAATCAAAATTTACTTGTGGATATTCAGCAATGCTAAGATGTATTACTGGACAGGGCCAAAAGGAGTATGAAAGAAACTAAGATAAGAAACGTTGGTGTCTAACACTGAGGTCTTACAAGATCCAAGACATTTTCCCAAGTACTACTGTTATATCTTAGAGCCCTCTTAGGGGAAATAATGTGGATTATAGTAAGACCTCATTCTTTTATTAATCTATATTGGATATCTAATTGAGAAAAAATAAGTGCCACTCCCAGAAGTAAACAAGGACTAGTCTTGAAAGAATGCTGCTTGTCCTACAGCCTCCAAATTTAACTGTTCCAGCAAGTCACACAAACAAAGCAGTAGTTTTCATTGCCTTGTGCTTAGACAAAATTGAAATATTTGCCATGAAAAATCTGGATTCTAATATTTTACAGGATTCCACCTCACCATCTTACAACCGTCAGAGTGAACGGGATTTTTATTCCCCTGGAGGCAGCCAGTCAGGTGGACATGGATTATCTTCGCAAGGTGGTGGCTACTCCAGTGGACAGGGCTCGTCCCCATCAGGAAGCAGCCAGTATGGTGGGCAGGGTGGATGTGATTGCTCTGGTGCAAGTTCTAGTGGATCATCTTCCTCACTCAGAAGTAATTCCTCTTCAAATGACACATCTTATTCTGGAGGAAGCCGTTACAGTAGCTATGATTCCACCTCACCATCTTACAACCGTCAGAGTGAACGGGAGTCATATTCTTCTGGAGCTAATCAATATGGCGGACATGGTCCATCTTCCTCAGGTGGGAGCCATTATGGAGGACAGGGCTTTTCTTCAGGAGGCAGCCAATCAGGTGGACAGGGTGGATGTCATTGTCCTGGTGGGAGCCCGGGCTCTTTTGGAGGAAGCAACTACCACCGTGGACAGGGTTCATCTTATTCTGGAAGCAGTCAGTATGGAGGACAGGGATCTCACTCAGGAAGCAGCCAGTATGCTGGGCAGGGCTCATCATATTCTGGAGGCCAACAACATGGTAAACCAGGATCCTCAGGAAGCACTTATTCTGGGAAGCAGCAACCACCTTCCTCAGGGGGCAGTCAAGTGAGCTCTGCTGGGAAACACTCCAGTCCCCAACTCCTGCTGCTGTTTAGCATTTTGTGTGCCTTCGTACTCTCTGCTGTTACATCAAAATGGTCTATGTGAACATCAGAAGATGTGCTCAGCATCCTAGACATGGATTATATGCAGTAATATTAGATTAATTACTTAACGTGAGAGGGAAAAAAAAAAAAAAAAACAACACCATAAAGTCAAATTGTCAGATGCTACTTGGGCTATCATGTTCCATGTCTGGTTACTATGTCATGTCTTTCATTAGTCTCTTTGGGTTTAGTCGTTAGTAGATATCATTTAGTTTTGTCAGTGTTCTAGGCATCATCTTAAGAGAGCATATTGTTCCCTACCTGTGGGAGTTTTTAAAATTATGTCCAGTGGAAAATGTGATGAACGCCTACCTGAAAACTGATTGGTGCGAGAAACAATTGCTTAGAAAATACTTGGTTAGTAACGAACACAGAATGGCATACAAATTGTTTTATTTGCAAACTGAGCACTGTCACTGCCCTTCACTACATACAATAAAGATGAGCTCTAACTTCTCATGAGCAGTCCTCCAGGGAATGGCAAAAAGGATGCTAAAATAATATCATACTATCAGCTTTAGTCGAATGTAAACAAATTCACATTCAACATGGAGGTTGCAAAGCAAAAAAAAGCCTAAATACGATCTTCACCATTTGGCTGACAGTTGTTTCTTTGGAGTCTGCAGTAACGTTTTTGTTTTTTTTAATTTAAGCGTCCGATCTGAACTGGCATTGCACTTCTTTAATAAACTGATGGTGTATTTCACACGAACCCCCGCGTCCCTTGCGGCTGTAGTGGCGCCCACGCTGCAGGACTTTCGGGTGAAGCGTTTCGATCCGCGTTGCTAGGCGGTTGCTGCGCTGGCGGCG

Novel00673
CTCATCTCCTGCATTATGTTGGGCCAAAACCTGTGAAGGTTTAAAGCTTGAACTTGTGAACTCAGTGACTGGTGCTATGGTCTGCAGTATTGCATTCACCCCATCCGCACTGCTAAATGAAAAAGTTTTTGCGTTTCTGGTGTCTGTGTGGAGGACTCAGCAAGCAAACAGCGTGTCCTGGCAGGCAACATCTGTGCTCTGCACCTGCTGGAAGATAGTCAGTGCCAAGTGGTGGAAAGACAATGACATCTGAATTTCTGCAATGCGTGATACATGCATATCCTAAACATATGGAAATATCTGTGACTGCAGATGACTACCTCCAGCTCCTTTTGAAAAGGAACGAAATTAGTGGAACTTTCTATGCCCTTTAAGGAGAGATGTCAGAAAAAAAAGATAAGTTTTGTGGGGGTTTTTCTGTTGTTTGGTTGGTTTGGGTTTCCTTGTTGGTTTGTTTTTAAATGCAGAGTTAATAGTTTTGTAGTCTTCAAAGTCTGTGTTGTCTAAAACACTGGTCAAAGTGGATCAGTGAGACTCCACAGGAAGACTTGCTTGTTTTGTGCAAAGCTAAATTTTATCCCTTTCATCTACGAAGAGAACTGTCCTTGCTGACACGTGAAGGGCCTTGTACATCATCTTCTGTGCCAAAGCTGCAAGAGGACTGTGGCTATCACACTTAATCTGTCTATCATCATGTTTTGTATTTCCTGACTTTGTTCATCTCTGTTTACTTCTCATCTGCAGCCTGGTTTCAAATTTTTGTGACACTGAGTAAATCCAGAAGAAGGTTTTTTTCCTGTACTGTTAATAGTCTGTGCAGACTATTCAAATCCTTTTAGCACACTTTGTGAAGCTTTTTAATAAATAATTTTTTTCATTAAAAAATTTATATCTTTCCTCCAGGCAAGAAAGAAGATCTATTCAAGATCTTTCAGTAGTTGTTGGTTTGTTGTTGTCTGTTTGTTTGGAAACATTTATTTATTCAAAGTGTTTTATTTCAAGTACATGTTAGCAATCTCCACAGATTATTAACAGTGAATCCGTTCCTCAAAAGGTAACAGTAATTTAGTGATAATCATTAGACCAGAAATATTCTTCTTTGTACTGAAAAATTAAGCCAGAAAAGCTTTTTTTCTTAGGCTCACTGGAAAATCAAGTTGATTAATTGCAATGTGCTTATCAAAACATCTGCCCTTCAGAACTGGGCTGCAAAGTGATGCCATCTTCATGCTGAGATTACAGCTGGATATGATGATGCCATCTACTCCACTGTCCAATAACATTCTTTTTCTAATCTAACCATGTTATTCTTGGATTGTTTGTAGCTGCATTATGATCACTACTTTGCATCCGTAGGTTGAGAGATCGTTGTTGTATTTGAAATGTTACAAAATACTTACTTTCCTCTAGAGTCCTGCACTTTCCATGCAAGTGAAGCTGCTAATAAGTTGACATAATATACATGCTTTTTGATCTCAACCTCATTTAAAACAAAACAAACAAAAACCCCCCCTGCAAATTATGGAGAACTGGAGAACTAATTCTGGGTGAAATCTCTAGAACTACACCCAGGTTTGCTTATTCTGAATTGATTTGCCTGTTCACAGTGTCCTTATGCTTCACAGTTGTCTTATGATCCACTTCATATCTAGACTGGTTTACTGTAGCGAGGAGTGGCAGCAGACATTCCAATGGCTGTATGCAATATCATAAGGGAACAGGAATATCAGAAATGTGTGTGTGTAGAGCTATCTAGAATACAGGTTAGTCAGTTCTGTCCCCTTGTAAGCAAGAGAGCATTTCTAACTTCTCTCAGATACGAACAGATAAAACAACTCTTTACCTCATTACCAGCAAACAAAACTGTCCTCTATGTAAAGGCAAACCCTCTGAAAAAGCTTCAAAAAGACTTGCACTCCACCTTTACCATAAATTTGGCCTATGTCATTTAAGCCCATCTATATCTATTATTTTCATAGCATTTGCACACCTCTGGCACATACAGCAGTCCAGAAGTGAGATATTTTGAACATTTAAAATGCATTTTTTATTATAAATTGCATCCTGCTCGTGGATAGCAAAGCATTTTGTTGATTGTTTGGGGAGCCATTAAAGGTCTCTGATTATACAATCAAGAAAGTCTCTGTGAAGACTTGTCACAGATATTTACTGATTATATAAGGCATCATTCTAATACTCATTGTAGCCTACAATATAAGAAAGGTATTTTTTTTGCCAATTACTTGTGTATCTGACCGTGTAAAGTAATGAAGTCCAGCCATTCAAAATTTTGCTCAGAATGGTTAAAATAATTCTATCACCTGAAACTGATGTGAAATGAGGCAGAGCTGAATTTACACCAGTTGGATGGTACAGTTACTGTTACAGAATTATGATGTTGAATAGACAAGGGAGGAGTGTTCTAAAGAACTCAGAACCATTTCCTCTGGAAACTTCCATTTTCAATGCTGCAGTGTGCAACAAAGAACAAATAAAATTACCAAGTAGGACAAAAAAGGGCTGTTCTTTACAAAAAAGAAAAGAGAAGAAAAGAAAAGAAAAGAAAACTAACTACAGTGCAAGAGAGATTTCGGTTGACAGGCTAAATACTGGCATGTAAAAGCTGAATTTAGCATAGGTTCTCAGCACACACATTACGGTAATTAATGGAATGAATCTATCTTTAGCCAACCTGCATCACCTCTTGAATCCCTCTGTTTGACCTAATACTGAGACAGAGACCTTTAATTAGAATGGTTTATTATAAGTGAGTCCAAGTGCACAGAGAGATTAAGAAGCAGCATGCAAGCAAATGAGAGCATAATAACCAGACAGAGATGCTCAGGTTTTAATAGAAGAAAGTGACCAACCCCAAAAACTCTTCTGTAGAAACTTGGCATTGAATAAATGACCCTGTGTGTAATGGCAGCTACACTGTGAAAAGCTACACACGGGTTTACCCCTAATTGGAAGCCAGATAGCAAAGACTTTCTTTTTGGTGTAGGTTTAATTACTTGGACGTTTTCTAGCATAAAAACTGTACTGAAAGGAAAGAAGGCAAAGAAAAAAAAAATAATTATCTGAAAGGAATATGATTTAAAAAGATGGTTTTCCCAAATTTTAAAGTGGAATTGGATAATGAAAAGAAAACACAAGTATAAATTCAGATCTGGATTGTACTGTGAAACCAAACTACGTTTCTAAACACCATTAGAAACGTAGCCATTCCTGTCTGTGAAATTAATTTTACAAAAAACTGAAGTTTTAATTCTTACTCATGTGCTCTAAAATCTCTAGGTCATTAAGTGCATTTTGCAATAATCATGTACAACAGCGATCTCTGCCCTTTGCAAGCATGTAAGGAAGAAAAATAATTGGAATATAGCAATTTTGCTTTCTTTTTACCTAAGAGCTTATCCAAACATGTTAGGCAAATCCTATCTAAACAAGATAAAAATCCTTATCTATTTTAGCCTGATGAGTGAAAATAAAATGGTTTGAGTTCAAACCATCTACTTCAAAAATATTAAGACGTGTAATATACAGAAATAGCAAGGTTGCATTCATTTTGTTTGTTACTATTTTTCAGTATTTTTGTCAAAAATTCTCAAAGCACACACTCTGCAAACATTTCACCTTTGTCATAGATGAAAGGTTTTCTTGCTAACCTCAGTAGACTGTGAGGGAGAGTGTGGCTCTTGTAGAAAATCGTAGAGGGAGTTGGACTCATTTGTATCAATAGGCAGGAACCTCTGCATCTGCAGTTTAAAATCTCCGGAAAAATTTAGAAACAACTGATGATTGTTCTAAAGGTGATGCATGAAACTACTTCCTGATGATTTTTTAAGTTCTATCTGTATACTCTGATCATTTTTTTGAATCCTAGATATAATGAATTTGTCTGCCAAAGCTACTAATGTAGCTTCATTTTTAACTGTACCTAATTCTCCTTGAAATGAATGAAAGCAGCCTGTTCATGTCTTTATGAAGTGAAGAGTATTCTGTAAAATTTGAGGTGAGCTTTCAGGAGCACACAACGTTGAGTAAACTTTCTTGGAACTGCTTGTAGCTCAAAGGCCTTGAAAAGCCAGTCTGCTTTTCCTCACAAACCCCAGTGAACATGAGAGTTAGCATTCAATCGTCATGACATCAGAGCTGCCATTTGAGAGTGATGGTGGTGGAGAACTCATCCAGGAAGTTCAAAGGGAACTGAAAGGCAAAGGGAATCGTTTCTGTCTCACCTTGTTGATTACAAACCAGTTGGACTAAAGCAGGAACGCCAAGCATAGCACGGTCATGCAAACGTGTGTGAGCTGGAAAGCCGTGGTTGCTGAGGGCTGCTTATCCCATTTGCTGTACAATGTCACCAAACAGAGCCAGGAGCAGAGGAGCTGTGTGCTCCTGAGACAAATAGGAAATTGGAACCCCAGTGACATGAAAAGATGCAGTACAAGATGAAGAGCACTACATGTTGGACTGAAAACAGAAAGCCCATTTATGAGAGAAATAAGGTTTACCTACAGATAATATAACTAAAGTTCTTCAAGATGAACTCTGCTCATTCACAGCTACTACCAGCAGTTCCCTTTTGCTTTGAGTTTTAGTTTCAGCTCTGCTTTTGAGGAACCTGTTAGAGCAATGAGCAAGCACCTCCTTGGGCTCCTTGGTAGGCAGTGCTAGGAAGATACTCTGCTGCTGCGGGCAGTGCTTCTGGAGCATCACCCGTGACAATGTGCTACTATTGCCCTAAAGGGATGCCAGCTTTAGTCTCTAAAACTTTATTCTAAAGATCAAAAAGGAAAGGATTTTATTGCTGAGGAACTTTCAATGAGAGCACACTGCAGTCCTCTACAGATCTGCCTGTACTTGCCTTTTTGCACCTTCATGGACAAGATTTACAGAACAAATGCTTTACTACCTGTTTGAAGACGTCTCCCGGACAGATCTTTGCACTACTCATCTAGTACTATTGAATGGCGGATAGCACTGAACAACTCTGCATGTATCATCGTATTTGACCAAGGAGGAGCTGATGAATCATCACTGTGTTCAACAAGCGATGAAATTTCTAGTCCGGTGCCAGTGAAACTGGGGCACTGCAGCATCATAAACTATCACTACATTGATAACACACAGGCTATTAACCTTCCAAAACATTCACCTCGGAGTCTGAACTTCGCAGTACTTCAGCTTCAATTCTGA

Novel00576

CTATGATTCCATGATTCCTCCATCCAGTGGGCAGCACAAGGCCGCTCGCTGCCCTCAGACCCACACAGCACAAAGGGGAAAGCTCTGCAATGGGCACTGTGCGCTACCTGCGGTTTTCACAAGGAACACTGGCTAGTGACTTAATGGGCATGATTGTGGCTGTCATTAAGGACAGTATCAGAATAGCCAGTGTGAACGACGGTGTTGAACGTAGTTAATGTTTAGCATTGTTCTTGAGCAGGAGATAAAGAAAGGGCGATTAATGCATCCCTCAGTTCTGGACAGGCTGAACCATATGGGGTGCCGGGCTTTCTGAGCTCATCTGGTGCTGAACGTTTACCAGTTTCCCGCATTGCAAGCAGGCTGAGGACCAAAGTTCAGCGGCATTTGGACACGGCCCCCGGGCTGCAGTGCTGGAGCGGCAGAGCTGTGGGTTTGCTTTAAGCCCCGGGAGGCTTTGTCAGCAACTCAGGTGGAAACCCGGCGTCCCGCTTAGCTGAACTTCAACATCTTGAGAAGTAGCGGGCGAGGGGTCGGCGGGGAGTTGCTGGGCTGCAATGCCCATGCTGTGTGACCTTCTCCTACTGCGGGTGTATGGATCCCCTCCCAGCAGCATCCCCTCTGCAGAACACCCCTTCTGCTGATCCTCCTCCAGGCGGCCTGTGTTTGTGTCATGGCTGAGGCCAATGTGGCCCCGCTGCTGACCCGGAGCCCCTGCCAAAGGTATCAACCATGGCTCCCTCCCTCAGCCTTAAGTCCCCTCCTCACTCCTGTGTGCTGCTCTGCTTCCCGGCTCAGCTCGGTGTGAATCACTGCTACATAGCCCAAAGCCCCGTAACCCTGGCCAAGTTGCTTTCTCTTCAGTAGGATGGGGTCAGCTCCTTCCTGACCCACAGCGTGAAACAAAATCTGCGATGGGAGCAGCAGGGTGAGCAAGCCTTCTCTTATTACCTTCATTTCCTGGCTGCCACTGAGCTGATGTGACGCCCTTCTAAAAACCTGCATCCTTGGGATGCAGAGACACTTGGGAACCCGAGCACAGCTCCAAGCCTCCTGTTCTGGCCTTGGGGTGAAGGCCATGCCATGAAGGCAGGCTGCTCCCAGACGTCAGCCTGGCTGGGTGGGGGTTCTTTGGCAGAACCCAGACTTTAGGCTTTGAAACCCATTCTTTTTAAGCCAGCCTGAGGAGGAGATGGTCCTCCTTGTCTGTCTGCAATCTCAGGCCCTTAGGTGTGCTCACAGGCCATGGGTGTCCTTTTTGGCTGGGTCAGTGACTACTCCTTGTTGAGCCTTGATTTTGTCATCTGTGATAAGTGAGCCACGTTCATCTTTTCTGAAACCAGCACAGAACCACAGCTGAGATTGAATTTAACCCAATTTGCTACAGGAGGGGGAGCTTAAAAGCGTGCCTGTCCCTTAGAGGACCAAAGAGGCTGGGTGCTTTTTGGGGAGCTCTGTTGTGTAAGGGAGCGGGGTCCATGGGCTCCTGGCGTCCACCAATGGCTGTGGCCTTCAGCTCGATGCTGCTTGTTAAAAATATTGTCCCAGCTGTATTAATATGACCAGACTGGATTGCATCTGAGCTGAACATTGTGAGCCTAAGGCAGGACATAAATATAGAATCAAAACAGCCTGACACAAGAGTCATCTGTCCTCTTGTGTGCGAGAAGTGAAGGGGAAGGAGGACGAGGGCATTCTGGGGTGGTTCTGGGGCACTTCCAGGCTTTGCCCTGAGCCTCGGTCAGCCCTGCTCGGGGTCTTGGGGCACAAGGGACCCCGGAGTGCCCCTGGTCAGTCTCCATGCCACAGGACTGTGCGCTCAGCACATTTTTATCCTGGGGTTACATTTGCCTAGACAAAGTCTGCAATCATGAATGACTATTTGTTTGATGTGTTTGCCTAACAGGATTACCCTTATCGTAATGATAGAGCAGCCCGGCCTTGGCACAGCTAAAGCCAAGGGCTGGGGAAGGGGGGGAGGGGAGTGCGAGCCCCTGCCTCCCTTGCCCCAGATGCAGCCTGACTCTGCTACCTTGGCAGCACCCAGACAGTGCTTTGCTGGCATTTAGTGCTCCGCTTTTGGAAAGAGCACCCGTGGTGTGTGGTGCTGCCTGCTGACATAGCGCCGGGCATTTCTGTCTCTGCACCTGAAAAACAAAGAGAGATAAATCAATCGTATTAAAAGGTTAGAAAAAGGATGCAGTGTTCTGCAATGAAGACGCCTTGCCCAGGCTCCAAGTGAGAGCCTGATTGAACTCAGGGTGAATCTGTGGCCCTCTGGTTCCGTGGGAATCAAGCTGTAGGGAATGGACCGATAAGCTGCTCGCTTTCTGCATCGTACACCGTGAACCTGCCTAAAACCATTGCAAAAGCATCTGTTTGTGTATGTGTGATTATTCAAATGTGCTGATTTGAAAGGCTCAAGGCAGGCCTAGGCCGGGCCCTCCCCAGCAGTGCTCCCAGCAGCAGTGAGCCCAGCCCTGCTGATGCCCACACCTGCTCCTGGCCGGGGACCTTCTCGCACTCACTGCTATTGTCCCTGCCTGAAGCCAGCCTGCCCTCACCAGGTCCAGGTGCTATTTGCAGAAGCAGAACCCCCCGCCTGCTTCTTGCCGGTTCGCAAACAAGCAGGATCCTCTATTTATAATGTGCTATAATTGGCTCAGATGAAACTGGTGGTTATCACACTGCTGGGGCCCTCAGGGACGCTGATGCCGCAGGGTATGGAGGTCTGCAGTGTGAGCCGTGTGCTCACAGACCCCCGCTTTCCGGAGCACCCCCTATTTGTCAGGGCTGTGCGGGTCTGCTTTTCTAAATGAGGTTTGGTGACAATGAGGCACTGCAGAGCCAGAAAGAGAGAAATCTTCCCACCGCTCCCTGGAGATGCTTTTCGTCAAACCCATCATTTCTAACCCCAAGGCTTTCGTAGCAGGGATGTGAGCAGGGCTCTGCGGCCCCAAAACGACCCGCTCCGTCACCGGGCCTGAGCCGCTCGGGTTCGCTTCATTTGATGTAGCATCTGTTTGGTCTTGATGGGCGTGCGGCCATCTGCAGCCCACGAGGGACCCGGCTGTTCGGACTGAAACTGCCCTGAGCTCATAGCTGGACACAGGATGAGCTGCCGGGCTCCGGGAAGAAGCCGTGGCTCTCTCACTTAGCAGCAGGCCCATGTGTCGTCATGCTGAGCCCAGGCCTGCACTTGGGCTGCTGTTGCTGTCTAAATCCTTCTGCCGTTCGAACTGTGAAACATGATGGATATATGGCCGAGCAGGGGGATTAAAAAAAAAAAAATAGTGGATGCTTTGCATATCTTTGAGGGCTTATTTTTATTCCCGTTTGTTCGTTCTCTCAGCTCTTGGCTGCCGTCGGGTCTGCAGAAACAACAAAGGGGGGTGAGGGAAAGGACAGCGCTTTGGGGGCTCCTCCTGTTGGTGGAGGAGCTGCAGGCAGCTCCAGCAGCACCTGCGGGCTCCATCACGCGCATTGGAACAGGAAATCTGCTTGCTTTGTGTTTGAAAAATCAGATTGATTTATCAGTCAAA

Novel00472
CTCTGTCCAAAACGGGAGGCTGTTCCTGGGACTTGGGCAGCAATTAGTGGCTAGATCCCAAAGGCCTTCAGCACCCATGCTTCAAAGCTGCAAATATCATATTGGCTTTGAACAGCTCCCAGAACATGGACTATTTAGCTTGATTTTCTTGATTTAAAAAACTTATTTCTACTGAAAAACCTTCAGGCATCAGATGGCTAACCATCAATTGACCTGTTAGATCAGATTTGGAAAATAACTGTATCCTTAGTGATGTAGATAGACATCTGGTTAGACATATCGAGCCATTAAACCAGATTATTTCCAGGAGGAGAAAACTATGCTACGATTCCTTCCTAAAAGAAGTTTTCTAAGTAGGTCATAAACCCCTGGAATTCAGTCCAAAGTGCTGACAGACCCCAAACTTTTAGAAGCTCTGTGTCTGCTGCAATCCCTATAAGGAGCTGTGGACAAGAAGACTAGCTAAGACCAGCTAGTGCAGATTCTATTCTGGGAAAGCTTTGAATCAGTTGGGGCAAATGAGTCATATCCATCCTCCATCTCAATTGGAATACATCACAGCGTGAAAACTATCCCAGGATGCTTTGCACTTCAGCTCTCAACAAGCACCCTCCCTGGCTCATCTTCCATAGTTGTACTTCAGTAAATGCTAGGAACATACCTCTTCAGCATCTTTTTTACTCTTCCACAGGAAATGGCCTGTCTCATGGTGCTACCGCTGCTTCTAGTAAAGTATTGTTCCTAAAAAGCTGTCTCCAAGGTATCTGCAAAGGGCTCACAAGATGGGTATAATGCAAGTGAAACTGATCATGTATGATTGATCCCAGGAGCCTATACTGAATTCAGTCCTGTACAACATCTTAATTAATAATCTGGATGGTGGGACAGAATGTACTGTCAGCAAGATTGTGGAAAACACAAAATTAGGGGGAGTGGCTGTACAGCCACCTAGGGGAATGTGGGCAGGCTAGAGGAATATGGGCAGACAGGACAATTAAGAAGTTCAGCAAGGGGAGTTATGAAGTCCTGCACTTGGGGAGGAATAACACAGCTGGAAGGAAGATTTTCAGATAAGGACCTTGGTATCGTGATAGGTACCAGTTTGAACATGAGCCAAACCTTGCCACAAAGAAGTCTAGCTGCATCCTCGGCTGCATTAGACAATATGCTGCCATCAGGTCGAGGGAGGTGGCCCTTCCTTTCTACACAGTACCAGTAAGACCACATCTGGAGTACAGTGTACAGGCCAGGGCTGAGAAATATAAATAAGCAAAGTTTTCAAATGTCATAGCATGATGAAAGATGTGTCTCTTTCATTGTCAAAGTAAATGTGAAAGAAACATTTGCTATTCATACACAATTGTAAGCAGATCAGAGAAACTGAGAAAATGTAAGATAAAATTGATTTTTAAAAATAATCTTCTTAAAAGGGTCAACTTCATTTTAGTAAGAAATATTAAGGCTTTCAAGGAGGGCACTAAAGATGGAGAACAGTACAATCCTTATTAAATAGTAGAATCTAGTTATTTCTGCAAAGTGTAAATATGAGATCAGTGAAATTTAGGATTTGGTACTTAAGCAAAATTGCTCTAAAGTAAATGTTTATGTTTATTAGCAGTACAATTTCAGAGATGCCATTTCAAAAGTAGTTTTCAAAGCATATTTAATTACAATGCTTGTGTGGATATAGCTATTTGGAACTTTCCTTACAGCACAACACTACATACAGATCTGAAAAATATGTTCAGGTCCAAATTCAAATATAAGACAAGAGGAATGAATTAGTAGTTTATGTCAAATGAATTTAATGCATTATCTTCTAAATGTTATAAAGCTTTTCCAATATCAAGTTTGATGTTTTAATTTTTTATTTTTCTGACTGTCAGAAGGCTGCTAGATTTTTTAAATGTACCATTGTTTTGTGGATATTAATGAAAGATGCATTAAAACATTAAAATAAGTCTTAAAGTAAAATCATTACATTTGATTGTAAACCCATTTTAAAATATTTTTACTGTGAATTCTCTTCCAAAACCTTTTACAACGAACTTAATAAGAACAATTACAGCTCAGTTGGCGTAGCTGGATTGAAAATATCAATATCCTTACTGTCTTAAACATGATTTTTGAAGCAATTAGCATTTGCATGTTTTTATTTTTCCTCATATAATTTAACGGACTCAAGCAGATGCTGGTATTATTGCTGCAACATACTACTCCCTGATGTTCAGTTACAAAGATTAATATGTGGCTCTCTTCTTGTCTGTACACATTTATTCCCAGTGCCAAGTCTAGAATTTGCAACAGAAATGCTCTGCTTTCTCAAAAGTAATGTGTGTCTATTTATTCTGTATAGTGGGCTAAATCTAGCACTGCACTATCCTGGCTTTATCTGTCGGTAATTCATACAAGGATTACATTGAGGAAAGACATAAAGTAAACAAGCAGTAAGTCAAAAACATTTTAATCTTCAAAAGAATATCAGAAGCGAAGTATTTCATG

Novel00469

GGGGCATCCACAACCTCCCTGGGCAACCTGTTCCAGTGCGTCACCAACCTCCGTGTGAAAAACTTCCTCCTAATATCTAACCTAAGCCTCCCCTGTCCCAGTTTAAAACCATTTCCCACAGTTCTATCACTATCCACCCTCATAAACAGCCATTCCCCCCCCCCCCCCTCCCCCCCCCATTTATATGCTCCCTTCAAGTACTTCAGGTTTCCTAAGGGAACACACCCATTTCTTTTTTTTTTCTGTTTGTCCAAATCTCATATTACCTTTCCTTTCCGGTAGATTTCTGTGGGTCAGCAGATGTGGAGTTGAAGTTGTCAGAAGTAAATGGCTGAAAAAAGAGAAAGGCTTAAATGCTCTGGTATTAATGACCTGGATTACAGGTTTGAGCACATTGTGTGTTAAAACAGTGAAGTGAGTAAGCAAGTTGTTCTTAATAATCCAGATTATTAATACCACCAATCAGAACATAGAGGTGCTGAAGAACTGTTTGCTATCTCACTTCATATTTCACTGTTCACAGTCTGCATCTTGTGTTCTGTGTGGGTCAAAAGAGTTGACTGAGCTCAGCATGGAAATACCTCCCAGAGCATCAAGATGTGGAGCTCTGTATGTCTTCCCTGCTGTGTAAGAGTGAGGCACCAGCAGCACATCCTGTGAGCAGCCAGCTGCTCCTGCCAGCCCAGGGACCAGATGGAACTGCAGGGAGCACCGCATGGAAACAGGTCTGGAGGGTTCAGGGTCACATCTACCACGACGGGTGTCAGACCCTCCACTGAGGGAGCAGCTTTGCAGGTGGCTCTGCAGCAGAGAGTTTTCACCCAAGTCATCATTCTGCAAGGCACAAGTGCTGTTAGACATTGCCATCAACTGGGAAGAAGGTGGGGTATTCCTAAAACCTCCTGGGAGCAGAAGGACTTTATAGCAACTATTCTGCTGTGGATTCCCAGACATTGTCCCATCCACCAGAGCCCCTAATAAAAGGGATCCTTGGTGTATTATGGGGATATCAATATTTCCCCATATCAGCAGGGTCTTGCAGATAAGTGTATTGCAGATGATTGAGAACGCAGACACTGATGTTATATAAATAAACAAGCACAATAAAATAATGGTGGTGAATAACTATTTTAGCCATGAATCTCTTAAGCTTTCGTTGAATCCACCAGTAATTCTCACCCAAACTTGTCTATAGCCTGGGGGATGTGGTGGTTTTTCAGGAGAAAGGCATAAAGATCCCTTAGGCTGAGCAAGATGCTGACAAGAGAATCCAGATACCTAGTACTGGGCTTGGGCTCGTGCAGCTCCATGAGCAGGATGTGGGCAGGTGAGCTGCGGTGTGGTCTTAAAAGCCCATTAGGTACTTCTTATATCTGCCCATGTGTATTTTCATGCACTGTGGTAAATGCAGAATGACTTATTCCCTGAAAAATGTGAAGTAATCCAGTCCACGTCTGGTAGGATATACGAGTATGGGTGTCAGACATCGGTATGAAATAGCTGCTCGGCTTGAAGTCGGTACTCTGATTGGTTCATGTAAATTCATGTTAAAAGCTTTAGAATTTGTAGTTGTAGCAGATGCTCTATTTTACTATGAGATTTAGCTTAGGAAAAAAAAATATTTTGAATATGAGGCAGAAGTGTCCCGTTTTTGTTGGCAGAAAGGTAGGGCTCTCCCAGATGACTTCTGGAAGCGGCTCTCCCATGTGAAGTGAATGGGAGCACTTCTTGACAGCCGTACAGCTCCGTGCTCTGATGTTCACCGGGTCTCTGTAGTCTTTGCAAAGCACACTGTGTTCTTTCAGTACATATGTCCATATTTTAAAGAATTAATGAGTAGTTACTAGCGGGAAGAAACTACATGTTGATGTTCTGAAATCAGATCTGCAATATAGAATATTTTTTTACTGCTTTTCGGCTTTACAAAAGATTCTTAGTTTTCTCATTACCATAGTCATTGCTGTTTTAGTTCTGAAACTTCCTTACACAGCAGCGTGTGTACTTAAGCCAATATTACATGCAAAAGCAATAATCACATAAATATCTGTCTACTGCAGTATGTATCCCTGTTTTTAACCCTATACGATAGAGGTTGGTTTGTTGTTTTTTT

Novel00425

GGAAATGTTACATAGCCACGCATTCATTCCTGACAATCCAAACCAAGAGCATGACCCTGAGAAAGTCAGGTGTACACCAAATATGTGGAGTAAATTGACAAGAACTGCACCAGAAAATTGCACCAGTACATTAGCAGCAATGTATGGCAGAGGGGAAAGAAGACCCCATTTAAATGAACTCATTAGTAGACTTCATGACTTTGAGCTACGTTTAACCCCTACATGAGCTTGCGATTCAGCCATTACAAAAGTAACTAAAAAACATAACAGAATGGAGAGTAATCAAGAAGATATGGTATACGAACTGTCTACTGTGCTTTATGTCATGGTTTTGTAATTTTGCTATTGGTATTCTACATCGTAACATCATGGACGAAAGAGAAGAACTCCATATCCCAGAGGACCTCACGGTCAGAGAAGGAAGATACATCATGGAAGATACGTCATCTGGTTGTGCGTGGTCTTTTTTTTTCACTTTCGCTTGCTGCCAGGGAGAGGAGTGGGTGTGCTTTCCAAGCCGTGCACCTTCATCAAGTAAGGCTTTCGGTTTCGGAAATTCTCTCACTCTCTCTCTCTCTCTCTCTCTCTCTCTATCGCTCTTTCGCTCTCTCTCTCATTTCATTTGGTTTATTATCCTTACTCCCAATTAGATTGTATTATATCGTGTCATCTTGCATCCCAACATCATAGTTAGTAAAATAAGTTCTCCTTAGATCGTTGCCGCTGCTTCATTTTTCTCGGGAAGCCGGGGGGGAGGGGGGCCCGCAAGCCTACTGCCCCCCTGTCACGGGCACAGATCTATCTAGGTAACTCCGTGACAAATTTTTGGTGGAGAATGCGGGCAGGATTCATCTGAAGCTTTAACGTTAGTAAGAAAAAAGGATTTCGTTAGGCCAGACTGTGAGACTACAAAGCATTGCTGGTGTTGGTGTGACCTTCATAGATTAAGAGTATAGCCTGGGCAGTCCGCAAAACTCCAGACACTGTACCGAGTGGTTTCCGTTTGTTTTTCTTTTTTCTCCTTCTCTTCACTCCCCCCCCCCTCACTTTAGCAGTTACCAACTATGAGGTTGCTCTTTATTGTCAGAGCACTATATAAGGGATTAAAAGCTATCATGTTCCTTGCCAGTTACCGGTCTATAATTAACCTCTTCATGTCTTGCTGCGGAGTTGTGTTCATATACAACCAAATAAGGGCCCTGATAGAGGCACCTGCCTGGTGGCTTTACGCAATGTGGTATAATTTGAATTTTGAGACCTTCGAACCAGTTTCCAGGCTTCCCTCTCAGTTTGTTGAACGTTTTTCGAATACTGAATCAGTGCTCATACTTTTGTGCATATTATCAGTATCTTTGAATGTATTCCTCTTCATTCGTACTAAGTGGAAGGAGCCTCCTCCAAGACCAGAGAATGATGACTGGCAAGGAATATGGAAAGGTTTAGGAAGAATCTTAGAAGCGTGGGGACCCGCCGTGCCATGGGATTTCACTCCTGAACATCTGTGGGATCCCGAGAAACTGAGCCAGCATTTGAGTCAGGGGCAGTGCGGCTTAGATAGATCAAAGGAGGCACGACTCATCTGGGGCTTGGCTTGTGCCTACCGTGCCCTGTATAGTACTGTTCTGGAGAGAGACAGTTTCCGAGCGGAGGTGCAAGCCGAAGGGGGAAATCTCCAAGTCAGACCTGATCAGTCACAACAGACACCAGTAACAGTGTCAGTAGCCCCTGTAGAAGGCAAGAAATGGAAGTGGGTGTCCTCGCGTCTAGAGCGAAAAAAGGAAGAAGAAGAAGAAGAAGAAGTGAAGGAGGAGGTGGTAGAGGAAGATCCAGGTGAGGGGACTTCCTCAGAACCAAGAAAAGCAAAAGCAAAAACTAAGAGGCACGAAGAGGAGAGCGATGAAGAGGAGATCTCTATTACTGTTCGTCTACCTCTCAAGATGTCTGAGATCCTAAGCTCAAGAAAGGAATTTGAACGACGCTCGAATGAAACTGTTGTCACCTGGTTGCTTCGTTGTTGGGATAGTGGGGCCAGTAGCTTGTCTCTAAATGGTAACGAAGCCCGCCAACTAGGAGACATTGCCGGAGACAAATCCATCGACAGAGCAATTAGTAGATGTTTGGATGGAGCTGCAACCCTCTGGGACCGAATATTAATAGCTGTGAAGGAAAGGTATCCCTACAAAGAACTTCTGCAGCCTGCAAAGAAAACGTGGAATACAATTGAGAAAGGTATCCAGTATTTGAGGGAAATAGCCCTGGTGGAAATGTTATATGACTCTAATTTTGCTCTGAATGATCCACACCAAAACCATGATCCGGAGAGAGTGAGGACAACACCTGAAATATGGCAAAAACTTACGAGAGCAGCACCAGACAGATACGCCTCCACACTAGTAGCCACATTCCACAGATATGAGGACCAACAGAGAAGACCCCTAGTTTTGGAATTGATTCTTACACTCCAAAACTATGAGCAACGTCTACCCCCAACCCACGTTTCCTTTTCAGCTGTCTCAGAGGTAGCAAACAGACTGGGTGAAGTAGAAGAGCAGATGTCTCTATTGATTAATCGTGATGAACCCGTAGAACCCGTAATAGTATCGGAAACGTTTGACGACGATCAAGATAAGCAAAGGGGCCTAATGTAAGAGCTTATCAAACTAATGAAAATCCAATTAATTAAAAGTGATGGACCCTCCTCCTCACCTGTAACATCAAAAATCTCAGCTGTTGAAGGCAAACGCTTTCCTGCTCGAGTGAGAAACAACAGGACTGCTTCGCGTGTTGCCTTGTGGTGTTATCTCCGTGACCATGGAGAAGATATGAGGAAGTGGCATGATCAAGATACTCCCCGTACTGCGAGCACGGGTGAGAGAATTACAGAACAGACCAACCACCAGTGTAGTTGCTCCAGTTACCACAGGTAATGAATAGAGGGGCCCTGCCCTCAGTCAGGGGAGGGAGAGGGATAACAGAGTTTATTGGACTGTGTGGATCCGATGGCCTGGCACATCAGAACCACAGAAATATAAGGCATTGGTGGATACCAGTGCACAGTGCACTCTAATGCCCTCGAATCATGAAGGGACAGAATCAGTCCATATTTCTGGAGTGACTGGGGGTTCTCAAGAACTGACTGTGTTGGAGGCCAAAATAAGCCTCACTGGTAAAGACTGGCAAAAGCACCCTATTGTGAGTGGCCCATGGGCTCCATGCATACTGGGTATTGATTACCTCAGAAGGGGGCATTTCAGGGATCCTAAGGGGTATCGATGGGCCTTTGGAATAGCTGCTGTAGGCACAGACAGCATTAAGCAGCTATCTGTTTTGCCTGGCCTGTCAGAAGATCTGTCTGCTGTGGGGTTGCTGCAAGTAAAAGAGCAGCAGGTACCAATTGCCACAAAAGGAGTGCATAGACGGCAGTATCGAACCAACAGGGATTCCTTGCTCCCCATTCATAAGTTGATTCGTCAACTAGAAAGTCAAGGAGTGATCAGCAGAACCCATTCACCTTTTAACAGCCCCATATGGCCAGTGCGTAAAGCCAGTGGAGAATGGAGGCTGATGGTAGACTACCGTGGCCTGAATGAAGTCACACCCCCGCTGAGTGCTGCTGTGCCGGACATGCTAGAACTCCAATATGAATTGGAGTCAAAAGCAGCCAAGTGGTATGCCACCATTGACATCGCTAATGCCTTCTTTTCCATTCCTCTGGCCACAGAGTGCAGGCCACAGTTTGCCTTCACCTGGAGGGGCATTCAGTATACTTGGAACCGTTTGCCCCAGGGGTGGAAACACAGCCCAACCATTTGCCACGGGTTAATCCAAACTGTATTGGAACAGGGCAGGGCTCCTGAGCATCTGCAATACATTGATGATATTGTTGTGTGGGGCAATACAGCAGAAGATATTTTTAAGAAAGGAGAGCAAGTAATCCAGATCCTTCTTAGTGCTGGTTTCGCTATTAAGCGAAGCAAAGTGAAAGGACCTGCCCAGGAGATTCAGTTCCTAGGTATAAAGTGGCAAGATGGGCGCCGTCACATCCCAGCAGATGTGATTGACAAAATCACTGCCATGTCTCCGCCCATTAATAAGAAAGAGACACAATCTATTCTGGGTGTAGTGGGCTTTTGGAGGATGCATGTTCCAAACTATAGCCTTATTGTAAGCACCCTGTATCAGGTGACGCGGAAGAAGAATGATTTTACATGGGGTCCTGAACAGCAGCAGGCTTTGGAGAAGATCAAACATGAGATAGCCCGTGCCGTGGCTCTGGGGCCAGTACGGACGGGACAGGATGTAAAGAACATCTTCTACACTGCTGATGGTGAGAAAGGTCCCACCTGGAGCTTGTGGCAAAGAACCTCAGGGGAGACCCGAGGCCGACCCCTGGGATTCTGGAGTCGAGCATACAGGGGGTCTGAAGAGTGCTACACCCCAACTGAGAAGGAAATCTTAGCCGCGTATGAGGGGGTTCGAGCTGCTTCCGAAGTAGTCGGTACTGAGACGCAGCTCCTCCTGGCACCTCGACTGCCAGTGCTGAACTGCATGTTTAAAGGAAAGATTCCCTCCACCCATCATGCTACTGATGCCACTTGGAGCAAGTGGATTGCACTGATTACACAACGAGCTCGAATGGGGAACCTCAGTCGTCCAGGAATCTTAGAGGTGATCATGGACTGGCCTGAAGGTCAAAAGTTTGAAACATCACCAGGAGAAGAGGTATCACGTGCCAAAGAGGCCCCACCATACAATGAACTACCAGGAAATGAAAAGAAATATGCCCTGTTCACAGATGGATCGTGTCGTATTGTGGGGAAGTATCGCAGATGGAAAGCTGCTGTGTGGAGCCCCGTGCAACAAGTTGCAGAGGCCACTGAAGGTTAAGGAGAATCAAGCCAATTTGCAGAGGTAAAGGCTGTCCAACTGGCCTTAGATGTTGCTGAACAGGAGAGGTGGCCAAAGCTTTATCTTTTTACTGACTCGTGGATGGTGGCGAATGCCTTATGGGGGTGGTTACAGCAGTGGGAGCAAAATAACTGGCAGCGGAGAGGTAAACCTATTTGGTCTGCTGAACTGTGGAAAGACATTGCTGCCCGAATAAAGAATACGGTGGTAAAGGTGCGTCACTTAGATGCTCATGTGCCCACGAGTTGGGCTACGGAAGAACAGAAAAATAACCATCAGGTAGATCAGGCTGCCAAAATTGAGGTGGCTCAAATAGACTTGGACTGGCAGAACAAGAGTGAATTATTTCTAGCTCGGTGGGCCCATGATACCTCGGGCCATCAAGGAAGAGATGCAACATACAAGTGGGCTAGAGACCGAGGGGTGGACTTAACTATGGACGCTATTGCACAGTTTATTCATGACTGTGAAACATGTGCCATAATTAAACAAGCCAAGAGGATGAAACCTCTCTGGGAGGAAGGGCGATGGCAGAAGTATAAATATGGGGAGGCGTGGCAGGTTGATTATATCAGCTTGCCATGATCTTGCAATGGTAAGCGTTATGTGCTCACCATGGTGGAAGCGACCACTGGGTGGCTTGAAACATATGCAGTACCCCATGCTAACGCCAGAAATACCATATTAGGTCTGGAGAAACAAGTTCTGTGGCGACATGGCACCCCAGAAAGAATTGAATCAGATAATGGGACTCATTTCAAAAATTTCTTATAAATACTTGTGCTATAGAACATGGCATTAAATGGATTTATCATATTCCCTATCATGCACCAGCTTCTGGTAAGATTGAACGATACAATGGGTTGTTAAAAACTATGCTGAAAGCAATGGGTGGCAGAACATTTAAGCACTGGGAGAAGCATCTGGCAGAAGCCACCTGGTTGGTCAACACTAGGGGATCTATCAGTCGTGATGGTCCTACCCAATCCAGCTCCCTACATACCGTAGAGGGAGATAAGGTCCCTGTAGTACATGTAAAGAGCATTTTGGGAAAAGTGTTTTTGGTCTTTCCAGCCTCTGGAAAGGGCAAACCTCTCCGTGGAACTGTTTTTGCCCAGGGACCTGGGTCCACTTGGTGGGTGATGCAGAAAGATGGGGATGTTCTGTGTTTACCACAAGAGAATTTGATGTTGGGGAGTGTAGCCAGTAATTCCGTGTATATATATGTGTGTGTTTTGGTTTTGTGATTTTTGTTGTCGATATTCCTCATCATTGCATCATGTAAAGCAGTGTGAGTTAATGTTCTTGTTCTGTGAACTCCCTTATTTGGGCATTTTTGTTCTTGGAGGGGTGGGGAGGAGCAGAATTCCCCAGAGGACTTCATGCCCAGAGGAGGACAGGTGGAGGTCCTGGCAGGACAACTGCCCCTCTCTCTCTCTTTCTCTCTCCCGGTCCGCGGCAGAGAAGCACATGGTTTTCCATGCAGTCTACACAGTAAGGCCTCGGTTTTGGACATTCACTTTCTCATTTTGTTTGATTTTGTTGGGTTTAGTAAATTTACGTTATTCCTCAGATCGTTTCTGTTGCTTTAGTTTTTTTTAGACCCATCTACTATCTCCCTTTCCTTTCACCCATACTTCCAGTGCCTTACATGATGTTATGATGTGGAATGCCAATAACTAAGATCATAAAACCATCAAAGAGTTATAGTGCTCCAAGACAGGAAGTTGCTCATTATGGCATAATGAGCAATGGAAATTTGCCAAACTGGTAAAAAACTGCTAAAGGTCAGCCAAAAGTCACTGTGATGAAGCTGAAGGAGAAATAAAGGTGTGGAAGCCGGTGATCATACGATCACCATAGACTCAACCACACATGCCTAGACGGACATTAGCATGTATTAATGAGTTCTTGGAAAAGAATGAATATGTTTGTCGATGTATTGCATATGTATGTCTCAACTGTTTAAAAGCAGAGCCTGAACTGAGAGTGTTTTTGTGATTTATCAAGTTACACAGTACATAATGTGCGGAATAAAAGAATGTTGGCTTTCTAACACGACATTAGAGTTAAAGAGTTTTTCTTCTCAGATTTGGGATGACATCACAATCTGAGTTTATATGACATTGTTAAAATTAAAAACAATAGCACAGTTACTACAAATACCAATAGTCCTTGACGACATTCTAATTCAGCATACAATAGTTCTTGGTTCCAGAGTCATCTTGGTTTCCTGTATTACACATAGTGCAGTTTGGTTACAACTGGACCAGCGTAGATGCTTTTAAATGCTGGAACTATCCATAGGGCTGGGGCTCTGCTCTTCTGTTTTCCCAGTGAGTCTGAAGAGCAAAATCAGGTCCCTGCAGTATAGAAAGAAGGGCTTCATAGCAAGAAAAGAGAAAGCAGCTTCACAAAGTAGACATAAGGGGCAAATAGAGATAGTTTATGTAGAGAGTATTAGAAAACAAGGGATTCCAAGCACTTTCACAGGCGCTAGGTTCAGAATAACAAGG

Novel00367

TCTACTTTCATCCTGAAGCAAAAAGAACCAAGCATCTGAATCCCTGATGGCAAGAAAGGATGCCAGCTGCTGAATCCCCAGCTCTACAAACCAGCTGCCTTCTGCTCCAAAGTGGATGGACTCTGAATGGTGCATGGAGCCAATTAGCCAAACCCCAAAATGCACCCTGGCTTCAACGCACACACAAATACAGAACAGCCCTGAAAAACGGAGTACTGCACGAAGTGGAGCACTACTGCAGCCTTGAGCTCATGAGCAATGAAACCTGGTTCCACCGGACTCCAGCAGTGCCTCAGTTTCCCCATCCCTCAGATTTTACAAGTCCCGCTCAGCACCTGGAGGCAACAACCCAACAACTGATGCTTGCAGAGAGAAGGGGAGAAGGGAAAATGCAGCCCCGGATGGACAGAATGTGGAGGGTCTGCGGCACAGGTGGGTGTTTGGGGCACGAAGTGGTGGTGATGCTCAAGCAAGCGTCCCAGATCCACGGCAAAAGAGAGAGAGAATTCTGCCAGCAAATGAGGAGAAAGATGGAGGGAAGGCAAAATCTCACGGCTTCAGGGATCTGAATGAAATGCTGAATGGGGAGGAGAGTGGTGGGAATGGACACAGCATGCTTGTCCCACACATCATTCTTCCCAACCAGACGGCAGGGGATGGAGGTGCACAAAGCACTGAGCAGGACTTGGCCAGTGGAATCGCCAGCAAGGAGGGGAGCAGAGGGACAATCCAGACCCTCAGTGTCACCTCGGAGCTGGGGGTAAATATAACAGCAAGCTATAGGGAGGCAGATACCTCCCATTCTTTCTCCTCAATTGCAAAGATCCAGGTGGAGGAGAGCTACATCGCTGAGAGGAAAACAAAGGATGGGGATGGGCAGCTGTCTCCTGGCCTCAGAGGTAAAGTCTATGACTACTACATCCAGTCTATCTCCCATTCAGTATCAACAAAAAAGTCGCTCCCATCTCCCTTTATGGGGACAGCCTTGAGCTGCAGCTCAGAGCATGTGAGGGAAGAGTTGCAGTGCTTGGAGCGGGTCAACGAAGAGCTGCAGAGATCAGCGGGGGTCAACAAAAAGCTGCAGAACTTTGCTGCTCTGCTCCCTGACACTCACTCCCAAGAAACAACTGTGGCATTGCAGGACCCTACTACCTCTCTTACTGTCTCGTCGAATGTGGAGAGCTTAGAGAAGGACCCCAAGCCCTCAGCTCCCACTCGCAGCATCAGCCGTAAGAACAGCGTCCTCCAGATTGCAGAGAACTCTCACCTCCAGTTGCCCTTGGGTGGTTTCTGTGTCACACCAGCCAACATGCCACCAGCAAGCGCTCAGCTGGATCTGGTGGACAAAACCAACTTCTTCAAAACGTCACCCTCCTCCCTAGACACCCATTTGGACCTGGGGAACTGCTACGAGGTCCTCTGTAGGGCCAAGGCAGAGAAGCTTGACTTCCTCCAGGAGGCAGCCTACAAGGTCATGAGTGACAACTACCTACAGGTGCTGAGGACGCCCTCCATCTACTGCCGCCTCAAAGCCAGTGAGCGGGACCTCATCCTTCGGCGCAGAATGAAGGGGAAGATGTGTGTCGTCGTGGCAGACATCAGCACACAGCTTGGCCTCTATGCTAGCCGACTCTGCTGCTATGATGACGAAGGGGACCGCTGGCATAATCTTTGCCACGTGCCACCAGAGGTGGTCTCCCAGGGGTGTGCCATGTGCAGCATGTTCAACTACCTCTTTGTGGTAGCAGGCTGCGAAGGCTTGGGCCGCACACCTTCCAACCGTGTCTTCTGCTACAACCCGCTGACCAATATCTGGAGGGAGATCTGCCCCTTGAACCAAGCCCGTCCTCACTGCAAGCTGGTGGCCTTGGATGGCTGCCTCTATGCCATCGGTGGAGAGTGCCTCTACACAGTGGAGCGGTATGACCCACGGTATGACCGCTGGGTCTTCATTGCCCCACTGCCTCATGACACCTTTGCTGTAGCCCACACGGCCACAGTGTGTGACGGGGAGATCTATGTGACGGGGGGCACCTTGCGCTATGTGCTGCTGCGCTACATAGCCCGTGAGGACATCTGGACAGTCAGCCCAGCTGGAGGCGGCAAGGACAGGACAGTGGAGGTAGTGAGCGTCAATGGTTTCGTCTACCGCTTTGACTACAATTGCAGCATGGGTATTAGTGTCTATCGCTGTAGAGCCAAGGCCAAGCTGTGGTATGAATGTGCCACCCACGCCAAAGCCTACCCGGCCTGCTTCCAGTGTGCCGTGATGGGCAGCCTGGTGCATTGCATTGGTCGCCACTTCCACATCCGCTTCTTGGCTGACCCTGTCTCACCACGCTTTGGGACCAAGGAGCTGCGGCCTTTCCCTTCACCCCGGGGTAGCCTGATCCCAGTCGTCCTGGTGCTGCCATGGGCAGGGATGGAGCAGACATGGGTCTGAGGCACGATGAGATCATGATAACCAGGTAACAGAAGTCACCCACCTTCATTTACAGCTTTCTACTGGACTAATGCTATCAGGACAATGTCACATTCATTTGCCTTCCACTGTTGGACCAATGCCATCAGGACAAGGCCACCACCTCATCTTCCTGTTCCCTGTAAGTGGAAAAGAAGAAGACAGGTTGCGCTTAAACACTCATAAACCCAGCACTAGGGATGCAAGAAAGAGCAAAGCTGGAGGTGGGCGGACTTGTTTGCCTACAGGTATTTAAATGCAGGTGTGTCTGCTTGAATGCAAATAAACTCAGGCTCCCAGCTGCCTCCATAGATTGCTGCTTTGGAGACAGCAGGTTTTTAATACTGGCCCACATGCCCAACATATGGGGGGTTTTAGGGCATAGAAAGATAGCAGTGAAGTTTGGTCCCTAAGAGGTATCTCCAGAGCATGTCCCCATCAGCACTGAGCTGTCACCGAGCCTTGACGTCACCCTAGAGTGGGAGACTGGGGATCAAAGCATCTCATCAAGGTATCGCTCCTTCTATTGCCTTGTTACCCATTTCCTTATCCTCAGCCCAGAATAATATAAATAAATATTTATACACATGTAATGTAAATAGATGCTATAGATTTATAGACAGCTGGTCTTAAATAAAATTAATGTGAACATTTGGTAAAATTAACCACAGGCTACTGTTGCACATCGTTTAAAGACTAATGGGAACAAAGAGGCCAGTGCATGTTTTATATTGCTAATTACTGCACAAGCAGCGACTCCAAGGAAGAGCCCAAAAGCAAAGCAGAGCTCTCATTGTGAGCAGCTGTTTTGGCACTGGGGCCTCTCCTCTCTTTTTCCTGGGGTTGTGCTGTCCTTGCTCTATTTACACCCTAGTGTCCTGCCAGCAGCCCACTTTGGGTAATTGCTTTTTCTTCCCCTAGCTCCTTGAGCAGCTAGAGAAATGGATTGTCATCTGTCATGTCCATCGCTGTACACTGCTGCGGTTCCTGTACCCATATCCTTGGTCAAGCACAAAGTGCATTCAAAGCACTTAGAGATAAGGGATGGCTCTGGGTGCCACACTGTATGTCAATATGGTATGGGAGAATGTACGTTCATGTTTATCCAGAGTGAATTTTCTGGGCGTGAGCAGGGAAATATCCTTTGTTTGTTTGGTTTGTTTTTCTCTCAGTTATTATTGCCACCTTATCTCATTCTCTCTGTCATTGCAATGAATAGCACAAATGGCACAGTTAGTGCACGTGGATGTTCATGGGTCTCATGGTCATGTGTTTTCAAATATTTTCTGACCTGGCACAATAAGTCATTAGCTCAAAGTCCACAGCCCTGCAGTACTTATTTCTTCTCTCTGCAAGGCCTAAAGTAGATTATCTTTTAGGTCCCTTCCAACCCAAGTTTTCCAGAAGATGGCCTTCAAGATCTTCCCACAACATTTGGTGGTATCACTGGGGACCTTCCTACCAATGCTAGGAGACCTTGGGAGGAGGCAAAGCCATGCTAATTAACCCTGTCTCTGCACTTTGCTTGTTGGCACATAGTATATTGTACACTTCTGCTGATGGAGGACGGCAATGCAACTCTCACATGCTAGAAAACATAATGGTCC

Novel00294

CTGAGTTAGTGGTTAAATTATCAATGCTTTTCATAGAATCATAGAATCGCTATTCTCTCACAGGTTGGGAAAGACCTTAAAGATCATTGAGTCCAACCGCAACCTAACCATACTACCCTAACGCTGACAGTCCTCTGCTAAATCATGTCCTTTTCACTTAATAAGGGCTAGAAATATCTTTATTGTTTGGTGAAAATAAACAAAAGTCTAAAAATTATAACTGACAAACTTTTGCATCATAGAATTAAAGTATTTACTGCTAGTTTTCCACTTGAGATATGTCTCTAGTGTGTACAGAAGGACAAATGATTAGACTCAATAACAAATGAATTAGGAAAGCCGTGGAATGCAATTTGCTATCAGTTGCGTAAAGTAGATGTTGACAAACACTGCAATAACAAAAACTGACTCAAACCAGATGGGGCATCTTTTTTGATGGAAGAAGAAAAAGGAAAAAGAAAGCTCATTGCATAACAGAGAAATGAGAGCTTCTAAAATCTTGCGTGAAAAGCAAACTTGTTCTTTCATTTTCTCTGTAACTTTGTCAAATGTGTGAAAGATGCTTGATGCCTTGAGAGGCTGAGTCCTGCTTCCTGCTGCTGAGGCCAGGGATCTGTGGCACCCTGTGCATCCCTTAATATCATGTAACAGAAGAAAGTAATTATAGAAGCACAGAATTATGGAGTGGCAGAAAATAATGAAGACAGCAATATGTGCTACTGTATTAGGCGTGGATTCTTGCAAAACAGTGAAGGAGAAACCAGGACAACCTGTTGCCAGTGGTTATTGATCCAGGGATAGGATGTCTCAAGGACTGGGCTTGTCCTGTGGGAAGTGCAGCAGGGCAGCAATAAGCTGTCCAGCCTTGACAACAGGAGCATATCTAGAGAGGTCACTGTGCTCAAACAAGCTTTCGCTGCTTGATCTTAATCTCTGCGGTGCTATTCATCTAGTTCGCTAATTGTCTGTAACATTACTTTTACTTTCTGTAATAAATGATGGCTTTACCATTTCCAGATGAGCAGGTCATTCATTGCCCCAGCACCGGCATCTACTGTCCATACCTTCCATATGGACACACACCAAGACTCCCATGACCAGAAGGGAGTTAAATGCATGAGACCTAGATTACTTGGGTATTCTTCTCTTCAAAGTAAATTTATTCAGCTGAGGGCAGAAACATTGGGAAACAGTCTCTGCAATCTTACATTGTTTTACAAGCATGCTTTATGAAATCTAGTTGTTACCATTGCTATTCTTATCCAATTAAAACAAATCAAACTGCAATGCATTGTGAATTGAGAATCAAACAGACCTGTCATATAACTAGACTGGTTGCAGATAGCAAGAATCTCTTTCCAGGAAGATGAGAAGGAGACAGAATGGAGAATCCACCGCATCCCAGTTTTTCTGTTAGTTTTGCAGTGAACTTCAGGTTGGTTTAGCTGCAGGATCACAGAATGATTTGTTTTGGAAGGGACCATACAGATCTAGTTTCAATCCACCTTCCATGGGCAGGGACACCTGCCCCCAGATGAGGTTGCCCAGGGCCCCACCCAACCTGGCCTTGAACATTTCCAAGGATGGGACATCCACAGCTTCACTGAGCAGCCTGTGCCAGTGCCCCATTGCCCTGTCCTGGTTTCATGTAGGAAAGAGTAGATTTTCTTTATAGCGTCTAATATGTTTTGGCTTTAGGAGAAAAACAATGTGGGTAATACACAAAAACCTTAGTTGCTGCTGAGCAGTGCTGTACTGAGCCAAGGATGTTTCCATTTTTCAGCTTCTCGTATTGTTCTGCCAGTGAGGAGGCTGGAGAGCACAGCAAGCTGGGAAGGGACAGAATCAGGACAGCTTACATAAACTGGCCAAAGAGATATTCCATACACATGTCCTCATGTGAAAAAAAAACTACAAAACTCAGGGGGGATGGCAAGAGAGGCAGGTGCTGCTCAGTGGCTGACTGGGGCACTGGTTGGCGGGTGGTGAGCAACTGTGTTGTATACTGCTTGTTTTGTAAATGAATTTAGTATTCTTTTTAATGTTATTTCTCTCTTATTTTTCTATCTTAGTAACTAGTTTTTATCCCAACCTATGAATTCTACATTTTTTTTTCTTATTCTCCCCCCCATCCTGTTAGGAGTGGGAGAAGTGAGCAAAAGGCTGTGTGGTGCTGAGCTTCTTGCTGGGTTACACCACAACACATCCTCACCATGAAGAATTTCTTCCTTATATCTAATCTAAGTCTAGTCTCTGAACAAGCCCAGCTCCCTCAACCTTCCTTCACAGAAGAGCTGCTCCAGCCCTCTGATCATCTTCATGGTCTCCTCTGGACCTGCTCCAACATCTCCATGTCTTTCCTGTGCTGGGTGCCTCAGGCCAGGGCTCAGTACTCCAGGATGTGTTCTCACAAGGGCAGAGCAGAGGGGTACAATCACCTCCCTTGCCCTGCTGGCTCCCCTCTTTTGATGCAGCCCAGGATACCATTGGCTTTCTGGACTGCAAGTGCACATTGCTGGCTCATGTTAAGTTTTTCATCAACAAGAGCCCCCAAGTCCTCCACAAGTCTTCTTGAAGTCCAACCTCTGTCCAGCCTATATTTGTCCTTAAAATTGCTTTGGCCCAGGTGCAGAGCCTTGCACTTAGCCTTGCTGAACTTTCTGAGGTTTATATAGGCTCACCTTGTCCTATTTGGCTAAGTGAAATTTTGTCACTTTTGATAAATAAGATATTTAGGCACCTGTTATCTGAATGAGTTCTCTTTTTTTCTGGAGTTAAAAGTGAACCTGGTAACCATCTAAATGACCTAGATCTTATTTGAAGAAGTGGCTTTGTAGGGAAGACGAGTGATTTTTTTAATACTTGCTAGACCAGCAGACCATTTGCAGCATTGTTACTGAGCCACGTGTAGAGAAAGATTCATGTGGAAGACCCGAGTAACACTCTTTAGTGAGGTGTCAGACTTTCAGGTTCATACACTTCGGCATTTGGTTATTTGTCCAGCTTTCTTGGAACAGAGCTGCTGAGAGTGACTGAAACTGGAGGAGATGGTGAAAGGCAGCATTCATAAGGCGTGCTGTATATTCATCCCCGTTAGCTTTACAGGGGGATAAAGTATCGGTAAGATGAGCTTTCAGATTGCAAGCTGACGGTTTTTCACGCAGGCAGAACTCTGATACGACAATTCCCCAAAGCACGTGGAGGTCCAGGGGCCATGAGGAGGTTGAGAAGCCATGCTGAGTACAGCCGAGTGTCCCAAGTACACCAGCAAGGGGCTGGATATCAAAAATTTCTGGTTCTTCCTCTTCTGCTTTATTTGCCTACAACTATTTTTAAGCAAAACAAATAGGACCATGTTTCTTGACATGCTGTGAGGGCAAATGTATTGTTTCTATTAATGTTTAGGAGCTGCAGCTGTGGGCCTGGATTCAGCTGCGCACCAGACAAATAAACAATTCCCACCCTGTCCAGATGACAAGAGAATGGGAAGCTTCAGGCAAATGGAGAAGGACTTGCAAGCATTGAGTCAGTGATGGATGGCTTCACACACAATAGTCTGAGCCTATCAGTAGTTTTCCCTGCATTGTATTTTTATTCATATATCTCCTGGCAGACGGCAGGTTGGCCATGAGTCAGCAGTGAGCCCTGGGGGCCAAGAAGGCCAACGGGATCCTCAGGTGCATTAAAAGGAGAGTGATCAGCACGTCAATGGAGGTGGTCCTTCCCCTCTACTCTGCCCTGGTCAGGCCTCACCTGGAGTATTGTGTCCAGTTCTGGGCTCCCTGGTACAAAAGATAGGGATCTCCTGAAGGGAGTCCAGCGGAGGGCCACAGATGATAAAGGGCCTGAAGCATCTCCCATATGAGGAAAGGCTGAGTGACCTGGGTCTGTTCAGCCTTGAGAAAAGAAGACTGAATGGTGATCTTATTAATGTTTATAAATACTTTAAGCGTGGGAGTCAAAGGGACATGGCCTACCTCTTTTCAGTGATCTGTGGGGACAGAACAAGGGGAAATGGACATAAACTGGAGCATAGGAAGTTATGCACCAATATGCGAAAGAACTTCTTCACAGTGAGGATGACAGAGCAATGGAACAGGCTGCCCAAGGGAGGCTGTGGATTCTCCTTCTCTGGAGACATTCAAGATCTCACTGGCTGCCTACCTGTGCTGTAGGAGGCCCACTTTGCAGGGGGATTGGACTCAATGACCTCTGGAGATCCCTTCCAGCCCCTACAATTCTGTGATTCTGTAATTCTGTGATTCTTACTTCCCTTGTTTAGTGAATGAAAAATTGAGGTTTTGAATGACGCTTGCTCATGAAGGCAGCTGTTCGACCATCCACTTGAACAGAAGCATCCTGTTTTTCTGTGAGGTTGTCATGGAAATGCCTTGATCCATTTAATTTATCAGCGTTAGATGTAAATAACAGTAATGACTTGATGGTCTCTAGAGGTCCCTTCCAATCTTTACGATTCTGTTATTCTGTGATCCTGTGATTCTGCAATACAAGCACAACATAACTTGGTGGAAATAGCAAATATGGCAAAACAGATCATAGAAATCCCAGCCCGCGTGATTTCTCTGTGCCATACATCAGAAGGACTGAGATGGCTCATTAACTGTGTGAGGAGCTGTAGGCTGTAGGGAAATGGCAATTGGGCATGTTCTTGTCTGTCACCCTCTTTATTTGAACAAGGACTTTTGGGAAAATAAGTGGTTACTATCTAAAATCACAGTTGTCTAGAAATGTTAGTGTTACAGGAATTGCCTTCTGCTAAAATTAATGCAGGCAACATGTCTGAAGATTTGCAGGATGACTTATTCAGCATCACTATGTGTGAATTAGTGTGTTTTAGCTAATAACTATCTCAGCTGCCCTTTGCTGTGCCTGTAGACTCAGCATCCAGCACCAAGCCTCAGCCCGTGGTGTGCAGCTTGCCAATGGGGCAGGGACATTGAGGTCAGTCAAAGGCAGCTCTGAGATGAAAATCTGGCACAAACAGGTTTAAAACTGTGAGACTAGGCCCACATTAGCAGCCAACAAATCAAAACATCTTGAACACTACTGCTGCAGACCTAGTGAACTGTGCCGATGGACTGCAAGGGTGGGCCTTCAGCACAGCTGCCAGCTGCATTCTGGAGCAGCCAAAGCTCTCCTGTGTGGTCTGTAGGTCAGTCTTTCTTGCTTGGAGTTGCAAGTGCCTTGGAATGGAGCCTAGATCTGTGGACTTCTTGTAATGTGCTTCCTCCTTCCTTTTCTCTGAGATTTACTCAGTGCCTTTACTAGCTATGGAAATGTAATTACCTGGTAAATTTATGCTAGAGTAATGACTACAAACTCGGTGTTCCCCCACTTATTTTTCTGTCCTTATCCAGTGCTGTTTATGAGCTCTCTGCAGCTCCTGCATGTGTTTTTTTGTCTGTTGTTGACATTGTTGACTGTTCTCCTTTTACATCCACAGCAGGATGTAAACCAATCAAAATGTAAGAAGTGCTTTAAATTATCTTCAGAGTATTCTGTTTACTGATTTAAAGGTATTTCCTTTGAAACGCACACAAAGAGATAAAATTCAGAGCTGACAAGGTGATTCTCTCACAGAACACCTCCGTTGGTTTGGAAGAGATAATTCTCCTTGATCTCATTCCTTAGTCAAAGCAGAAAAATTAGCCCCAGAAACAAACTTCGTGTTCTGTCTTTGTTGTCCCTCTGTACTTATTTTGCAACCCTGTTAGTAACGTGTTTATCTGTGTAGGGCTAGTGCTATAACAAACTTTGATGTATAATATGGCAAGATTTTCCCTATCCAATATTTAAAAGAGATTGAGAACAATGGCTTGCGTCTGGTCTAGATTAGAATGTACAGCAATGCTTGTTCACCAGCATCTGCTAGAGCATGACCAAACCTATCAATACAACCACATACACCCTAGTGAATAGGCGGTGCTTTTGGAATTAAAAGGCGTTGAAATTAAAACTAAAGCAGAACGTGAGGCCCCGGATGAGGAAAGATAAGGAAATGAGCTCTGGAACAGGCTTTTAAAGTCCTGTGCTCTGGGAAGCTCTCTTGATGTCCTTAATGCCTCAAAAAAAGCTGCAAAGTTTCCTTGCTTTCGTCCACTCCATTCTCCTTTTTTTTTCTTTTTTTTTTTTTTTGGCTAAAGAAAAATCACTGTTTTTTTAAATGTCAGTTTTAAAGGACTGGACAAGACATTTTAAGCTGTGCCCCCAGTCTCCCGAGAAATAGCTTTCAAGATCACGAAATTAGTGAAAGATCTCCACTACTCATAATTAAGCCTCTATGGTTGCCTTTCAGATTTAGAAAATAACCCTCCCTGTGCCTGGGATTTGCAGGGAGAAGCTTCACTGCCCAACTTCCAGAAGACAAACAAAGTCTCTGTTACAAACCCCCTCCCGAATGTGGTCCTGCCTGTCACTCATTTATAATTTCTTTTGTTGGTAACCAAAAGAGATAAGAGGGAGGCAGCCTTTGCCCAGCAACTGTTCTGTGCAAAGGGCCCTGTGAGATGGGAGGAGGTTTGTTGTGACTAATCCAAGCTGATAGCCCTTGCATGGGGATGTCAGAGCCTGCGGCCGTGCGAGCCCTGATGATTCCAGCAGGCCTGTGGAAAGAAAGCAGCACTGGGTCATGGAAATCTGCTAATATTCTTTCTCTGGGAGAGAAAACATGCACTGGCATTAATAAGCATGGAAAAATTCCGTAATGCTGCCCATGTATATTTCAAATGGAGGGATTGGGGCTGAAGATAATCAACCATTTTCTTCTTTCTGTTTGAATGGAAGGCAGCTGGTTAATAAACAGATATGCAGACAGAAGAGCTTTTGTAACAGGATGTAGAGGGTCTAGAGGGAGGTGTCCTTGCCTATAGCAGGGGGTTGGAACTGGATGATCTTAAAGGTCCCTCCCAGTCCAAACCATTCCATGATTCTATGATTATATGTACAGGGTCTTCAGTGGCACTGCTCACTAGGAGAGGTTAACATATTGTTTTCTAAGACCATTATATATTAACCACCATTTCCATTTTAGTCCTAATTTTTTCTATGATTTGCCCCAGTATTCCTTCTAGAACCCTTTTGGAGAAAATATATAGAATATATTGTTTCTCAGAAAAGAGCTTACAATGCAACAAGTAAACCATTACCAAATATAATTCCTTATGAATATTATGTTGAATATTATTCAAATATATTACATTCTTCCGAATGATCTTTCCATTAGATGCTCCACACATTTTTCAGGCCCTTTGTGCCGGTTTGTTTTGAACACCATCCTGAGATTCCTGGCTAGGAAAGAAGGAGGTTTTTCTCTGTTCTAACCCCTCCCCACTCCTCCTTCTCCAGTTTTCAGATGACAAATGTGATTACACTGTCCTAAGGAGAGCTTTTTGCTGTACTGCAATGAGCAGCTGACAGCCCCACGGGGTGGTGGGAAGCAGGATCAGCCAGGCTTCAGCATGGACTATCATCTCAGTGGGTTTTATGATGCCACAGGACCAACCAGGTTTCCTGCTTAACAGCAAATGACGATGGTGAGAAGATGAAGACAACAAAACAGCCTAGAGGTTTGCTGTTCCTGCTGTGAGCAGGCAGGAAGTCTGCAGGGTTCCTCCAGGGAAAGGGGCCTCCAAAATCCTTCTGTGATGAAACACTTCAGTGGTAAACTGAGGCCCAATCCCTACCAGCCTGCAAGTACAATTTAGAGGAAAGATGGTTGTGTGAGATGGAGGCAGGGAAAGAGGAAGCTCCAGAAGACAAAAGGGTGGTTGACAGTGATCCATACTTGGCTACCATTTTGGAGCATCCTTGGAAAGATGCAGAGGAGTGGAAACAGAGGGAACCAGGCCTACCATCCTCTCTGTTCCCATGGTGAGAGAATGTGTTGCTACTGCATCATACCTGCAGCTGTTGATATGCCTCAATATGCAGCACCTGTGATTCCCTGATACCAGAAGTCAGGCCATATTGAAATGGCTGAAATGGCTCCAAAACCAGGGCTTTTGGTGTCTGGCACTGGAAGGTATTTATCTCAATGCATGTGAAAAGACGACACAAATGAAATCTGCAGCCAAATTGGTGACTATTTGGAAAATATTGTATGAAAACAACACATTAACTCCCCAGTAAGTCAAACCAGCATCTTTTCATGTACTCAGTTTCTTCAAAACATGTGTAGGTCACTCAGAAAGTAATGCCTCCTATTTATTTCCATGGAAACTACAACATATACGAAGAGCACAATAACACTGTTTGATAGAGCGAATTGTCAGCTACAAAACACTATTTTTCAACATGGTCACCACTATTAGCTATGCATTTTCACCAGTAATGAACAAGAGCCTGCATGCTACATTCATGAAAATCTGCACCAGCAGAGGTGACCCACTGTTTCAAAGCTGTTATGATTGTGTTACTGCTAGGAAGATGTTGCCCATGCAGTCCATTTTTCATCAACCCAAACAGGTAGAAGTCAGAAAGTGCCAAATTTGGACTACACAGTGGGTACAGTAGAACAGTCCAGCCATGATTGGTAATGTGTTCCATGGTCTTCAAACTGGGATAGGCCCTGATGTTACTGCGTTGCAAGAGAAAGGTTGTCCTTCTCTGGTCTGACTCTGAAAGTTTAAGCCTTCAGCTTAGTCAGTTTGATGATGCAGTGGTCAGAATTGATGGTTTACCCAGGTTCCAGGAAATCCACAAGGATCACTGCTTTCCTATCCCAAAAGACAGTGCACATCACTTTACCTCCTGAGAGCTGCATCTTGAAATTTTTCTTTGATGGGAAATTTGAATGTCATCATTCCATGGACTGCCATTTTGACTCTAGCTCGTAGTGGTGACACCATGTCTCATTGCCAGTAATGATATGATCCAAGAAGCTGTCACCTTCAGTCACGTATTTGTTCAATAGAGCCTGAAAAACTTGCATATGGTGTTCTTTATGTGCCTGTGTGAGCATTCATGGGACAGACCTACCAGGAGCAATCTTTGTGACATTTAACCTTGCCACCATTTCCAACACACTGAAGCCAATATTCAGCTCCATACACTCTGTTGCCTAATCTTAATCTGCCAGTTCACGTGGATGAGCTGATCAAGACCCTCCTCATTTTGTACCGTGACAGCTGTGCATGTCCATCTGGAATGTAGCTTATTTCTCATGTCGCAGCCGCCACTACTGAAACACACCACCCACCACCTCACTGTGCTCACATCCACTGTTTGGTCTCCACAAGTTTTTAGTAAGCCTTGATGAATATCAGTGCCACTTTTTTCCACATGGAGTTGTTCAGTAACACAGCTTTGACACTTCCATGTCAGACGCCATTCTGTCAGATTGTACCTCTGCTGTCACCTGTTGCACGGCAACAAAATATTGGATATTGGTGGGAACTTTCAGCTCCTACTGTCATACCACCAACATCTGCTTCTGATGTTGTGGGCCAACATAATAAAATTGAAGGCCACTACTTTCTGAGCAGCCCTTGTAAAAATCCCTTTTTTGGAGGAATTTTCTCACGAGTGTAGGTAAAATCAGAGGACGAGGGAGGTTGTCCATACTTCTAGAAACAGATCTGTAGTGACACAACAAAAATAAGTTCAGTGAATGTTGGATTCACATTTTTTGCTTCTTTCCTGTCTGCCTTTGCTATTCAGAAGTCTCCATTCTTCTGCTTGAAGCAAGCAGCCCGATGCCTCTCAGGGCTTGCCCTCATCTCTGCAGCTCAAGAGAAATTGAGCTGCAGAGCTTTTCAGAAATCCTATCCAGAATTGTCAGTGGAGCTCCCAGGCAAGAAAAAAGTGAACCAGTTGTGCACCGTGTCATGCCACAGGAGCCATCACCTGAGTCTAAGCCTGGCACTTCAGGCTGTTCACAGTGCAGGGTGGGAGCTGGAGCAGGAGGCTGGCAGTGGTAGCCTGGGATGTTCCCGGTGAAATCACATATCCCAAGCTTCACCTATGTAAATATTTGCTTTTTTAACCCAATGAAAGGGAAATACAGGAAGTAGCAGAAATGATGATGTGAATTCAGAAGACATGCATGGGTGCAACTGCCCAGATAAAGATTCTGCAAAGAGCAATCCATTCCAGTTACAGTTCAGAAATGTTTGAGCTTATCTTTCTAGTTCACAGTAGAAGAATGAGTCTTATCTTAAGCAAAATGTGCTAGATCATTACTAATCATAATGGAATTTTTCAATTTGATGAAATTGATCTGGCCCTAGTGGTTATTGCTAAAGTATCAAAAAAAAATAACCTCCTGCTATCAGCTCAAGTCTGTGGTGAATCACTAGTTTCCTATATCTTCTTCAAAAAAAGGTTTGAAATGCCTATACTATTGCCTATACTGATTATTTTTTAGACCTTGGTGTTTTGGAAAGGAATTACTTACTAGATATTTATCTTTTTTTTTTTTTTTTTTTTTTTGTAAGACTGCATATCTTACTGATGCTAAAAATAAAAGTAATTCAAACTGACTGAGTTATAGAATTATTGTTACCATACCGAAAATGTCACTGTGACAGAATAACAAATTCTAGAATAATGAGCCACAGACATCTGCTGGTTTCTGAACTGTTACCATTCTTCCACTGCCGTGTATGATTCTTACTTTTTCTGTACCATTTTTAGGTTGTTTTTGTGCCATATGAGCATCTTTTGATATTTGGGAAAAACTTAGAATGGAGTCACTGTGGTATACAAACTCTGAATTGAGGTGAATTCTTTCTTCTAGTTTTGCTCCAAATTGCATGGAGCATGGTCCATGCCTGCAGCAAGGGAAGAGAAACTGGGCAATGTGTTTGTTTATGTTACAGAAGGTGCTGCCTGCTGTTGTACCCCTGTTAAGTCATTTAATACCTCCTCCAATTTTCATCTTTCCTTCCTCTCTCCTCTGTCATGTGCTCGGCATGGCCTGCATGAAGAGGTTTGTATGATGGCTCTGCTGTTGTCATCGCTGGTGCGTGATCTGCATCATCTAGTTACAGCTAAATGTTTACCAAGATGAGAAGTCACAGTGTTCAAGAGTGACTGAGAGGTCAGTTTGACTATTCCTAATACTCTCTGCAATCCTTGGGTTAAATTCAATTTGTTTTTCTAGAATTTGTAACATTAACATTCAGGAAGACATTTTTTCCTAAATTCTAAGTCTAGAAAACTCTGGCTTTTTTATCCCCTCCCCACAGAAATAGGCTGCTAGCATGCTGATTTATTACACTCAGATCATCAGTGATTTCCATTTCTCCGATGGAATCAAATTCCACTGGAGTAAGTGTACTGTGAAGTGTATAGCGTTCTTCCAACAACGTGTATCTTCAGGCATTAGAGAAGTAATGATTCTGAAACAAAACCAAGTGTAAGCCCCCACTGTGAATGGGGAGGAAGCCAAGCATGTTTCTGCTGAACTGACAAGCTTGGGAAGTCAAGAGACAGCTTAACTGAACAATTGGTTCATCGACTGGATGAAGTCTTAAGTTACATGAATGACAAAACTACCAATTATTTAAAAAAAACCCACAAGGTATTTCTTGCAGCAGAAGCAGCATGTAGTAATTAAGGGTTGGGCTGTCTTGCCTTCACAGCTGAAGAGAAGGACGAGACTCCAGTTCCAACCAACGCAACATGGATTAATTGTACTTTCTCATAATGCAGCTTTAGAAATGCTTCATATCTTACATTTCACTTCATGTTTTTACTTGGTTGTAAGCAACAGTTTGAAAAGAATAAATGCCTTATATCAGACAGAAAACAAATCCTGTTCCTCCCCTCTATCCCCCTTTCCTCCCTGTGGTTATTGTTCTGCTTTACGTTATTTTCCTGTGTTTGCAGGCAGGTTGCTAGTGAACAGACCAGATAAGCAGAGTGAGAAGGCAGCAGCTGCAGCTCAGCATCCCCACTGTTACAAGAGGAATGCCGCCTATATGGTTTCTATATGCTGCCTTTAAATACAAGACTCTCAGAAAAATCCAGGAAACAGAAGAGTGGGAAGGTGCTGAGAGGGAAGTGGGAAGCTTAGATCTGCTTACAGCTCTGCCCCGACCCTCTGACCTTCTGCAAGTCACTGCCACGCTCTGCCCTCCTATACATACCTTTGTCCTTAGTGCTGCTCTGACAGCACAAAGAGTAACCTGAGAGATGTCCCAAGCAAAAGGAGAACCTTGTAATCTATGGGTTTATCTTCATTCCTCCTTATCTACTTAAGCTTATCTACTATCTACTCAGTGCTACAGCTAGCTGTTTCCAGGGTTGACTGGAGATCCCTTTATTCTGTTTGTCTACCAATCTATGCTGCCCAAGGGGCCCCCAAGAGGTCTATTACATGCTTTTTTCACCTGATTTTTGGATCTCCTTGAAGTTTCATAGTACTGTGTGCAACTTCATGTATGGAACTCTACTGAAGATAATATGATTTGTCTGGTTATCCTGGAGCTTCAAGGTGTCGGTGGACAGAAAGTTAAAAAACCCATTACTGTTTGGTGAGTTCTCAGCCTCGTTAGTGCTATGAACTCACACGTGTGGGAAATTCCAACACTTTTCTCTTTAAATCTCACCACAAAGACAAGAAGAGCTCTCTCTTATCCCTCCATGCACTCTGTATTCTGGTGATTATATCTAAACAGTACCATTCGGAAGTTTTAAAATGTAAATGAGACAGAGTGGTTTCATTATTGTGGGACTGTTTTCTTTTTCTTTCTTTCTGCAAAGCAAAGCCCTTGAAACAGACAGAAAACCCCATGCCTGCTTCCTGCTATTTTATTCTTCAAAGCTGCAAAACTTCACCAGTCATAAGGGATTTAGGTAATAGACAGGCAAAGAAAAGAGAGCATGGCATCTCTGTCATTTTGCTACCAATTAACTTTTGATCCTCTTGTTGCTCAAAAACACGTTTTAAATTAGAGATGCGGTGATTCTGTATGCAATCAATTTATACACTGAAGGGAGCTCACTGGTGGGACATCACCAGGACCTCATTTATATTCTTAAATTTATGAGGAAATTGACAGAATAGCAGTGATAGTATTTGCTAATGATACAAAATTACACAGAATAGTCTTCAGAAATTGATTATTAAGAGCTGCAGCAAGATCCTATGGTAATGAATACAAAAGGCAGATGAAATTCAATGCTGCTGAGGGCAAAATATGCATATGGGGAAAAAGAAGTTTAAGTATGCCCATGCATTGGATTTTCGTAGCCTATCAGCATGAAACTTTGGGGGAACTGCTGCAGTGTCACACAGTGACAGCTCAGGATGCAGCAGTGCCACAAGGTGTACACACATTAATAAGAATCTTGGAAGCACTACTGGGAGAAGTTACAGTACTCTGTTTCGCAACATGGAGGATGTAAATAAAGTGCAAGTTATTTGTGTGTTCTTAAGGTCAGGAGTTCTGAAATGAGAAGAACAAAATAAAACTTTATTGTCTCTCTGCTTAAAAATGTTACTAGAAATCTAACCTGTCCAACACACTGAATCTAAAACCTCCACGAAACATCATTTTATTTTGATGTTTTGAAATACAGAAAAAGAATGTCTCTGGAATAAAGAAGCAGGAGCCCTTGTTATTAGAAGTAAATAAAAATCTTTCTTGAAAAAATGCAGAAAGAATTAAAAAGAAAAAAGAAAAGAAAATTAAAAAAAAAGGAAGAGGAAAGGGGAAAAGAGAAAGGAAAAAGGGAAAAAGAAAAAAAGAAAGTGAAAAAGAAAGAAAAAGAAAGGGAAAAAGAGAAAGAAAAAGTGTTTGAAAATGCAGAGCTCAGTTGTGAAACTGCTTTGTCTTTTGCTGAGCTTACTGACATCTGTGAGAGTGCTCTTTAAAGCACACAGCCTTAAAAACAATAAGGCAGTGAGATTAAGAAATGAGTACAATATTTGACATTTCAATTATTGGTAGGAAGCTACGAGACATGAACACAATGCTTTAGGAAATGAAAACTGAATTCAGGATGTACTCCTCATTGTTTACGCCCGAACTGTGCTGGTCTGACAGTAAAATGTGAACTGAAACACTCCACTTCTTTTTAAGAAGCTTATTAAGTGTTCTCTTTAGCTGATTAAATTAGTTTCCTTTCAGCTGTACAGCCTGCAAGCTAAAAGTCTGTGTTTAGGTTATAGAACTTCCATCCCGAGACATTCAAAACGCATGAATCAGAATGTCAAAATCATGACATTTCGAAATACAGCAAAACCCTGGATTTTCTCACTTCCTGGATTTTCTTGAGCTTGAAAATGCCACGTTTTCCTTCTCTTCCTTCTCTCACAAGAGAGTTTTATTGATTTACGTACTTATTTTTAATGGAATTGTGTGGCTTCGGGAGTTGAAGTCTCACTTTCTAGTCCTGGCAGACACAAATGAAATAGTGGTGAATATGAAATTATCCAAGACTTGTAACAGGAAAATGAGAGCTGACAGTTGGACGGTTGACATTTAGGTAGTGTAAAAAGAACAGAGCCCAAAGCCTTACTCTCAGGGCTGTGTCTGTGAGGTTGGTATGGGGTTTGTCAGCTGGACTGCTCCAGTCAAAGCGCAATACCAAGCTCCTGAAGACCACTGGTTGCTCTGTTAGTGGCCATGACTGCAGTGTGCTGCCCAGCCTTTTACCTATAGGCTTCTCCTAAAAGCTCTTATTTAACTTCAGAAAATTGTGCCCACTCAATGCAATTAGGATATAACAAACACCAACAAGGCCGTACTTCGAGAAATCTTTCCCCTCCAGAGTTCGACTGCTTAACAATTTTCTTTCTACATTCCTGAAGTCAATGCAAATGCAAAAATAAAAGCACTTAATAAAAT

Novel00270

GCACCACGTCCTGCTCCACAGCACGTGAACAAAGAGTGGCATTAGAGGGCAGAAGACAGAGTGAGCAGAGAAGAGTGGAACTGACAATTCGTGGGGCAAAGAGTGGCCAGCTGTAGGCATGTGCTGAGGATAGCATGCAGGATGTATGTTTAGGGAACTGAAAAGTAGACCATGTGTTTCTGAGTCAGAGTCACTCCTGGAGGCTGCTGTGCTTGTGACTGGAACAAGCTTGCTATTTAGCCCTTGGTACTGAGAGGACACATGAGCCGTGCTTCACTGATGAGTGGTATCATTTATACATCTGCTCTGGTTTTCACCTCTGGGCACACTGTGAGCCTGCAATTGCACTGTTTGCAGGTGCTGGTTTTTACATGTTAAAAAAAACTAACACCTTTCCATAGAGTTTTGGTAATGTTTAATTCTGAGGTGTCAAGAATTTCTTGGAAGAAACAGCTCCTTAGACTGATCTTGAGAGCATGAAATCAAGCAGGAGAAGTAAGGCTGCTCAGGGTTTCAGGTGCCGTCTGTAAATAGTTTTGGCAGCAAATCTGATCCTGTCTTCCATCTTTCTCATTCCATCTTTTTCAGCACGGTCTGTATGTTCTAATTTATTCCATGCTTTGAAATCTCATTACATGAAATATGTATGAAATACTAGGTATGCAGGTGGATTGACATGCTCAAAACATGGAACTTATCCTTCCTCACTGCAGTCTTTTGTGGCAGTGATGGGAGGAATGGCAGCCTCACAATTCTTAGGACTAGCAGCAAGGTTGGTGACTCGTAGTGCTCTTAAACTTTCCTGACAGAAACAATTTGAGGAGAATCTGCTCAGGTATATTTCTTTTTTAAGTGCTTGCTGAGAAGCTGCTTACAGACCCTCTTTGCCAAGGCCTTTTGGGGTGATCTTTCTGCCTTAAGATGCTTCTACTTCACTTTGGAGGTGGTACTCCAACCTTCAGGGCACCTTCTGCTAATACTGAAAGAATTCTACCTTGCAGGTGGCTTTAACTTTATTGTTGGTTAACTTGGCTAATCTCCTATCGCAGCCCGCAGCATCCCTCACATTATGATCTTTCATCAGTGTTTATTTCCTGTTTCCCCGGGTGGTTGTGCTCAAAAATGTTTAGAACTCAAGTAGTACTTCCTTTAGTTGAGAAACTACAGGAAGAGAATCAAGTCTCATGATAACAGTAAAAGCCCTCAGAGCACTACTCAGGCTAACCAACGTTGTTTCCCATTGCAACTGCTCTTCTGTGAGAAAGTGAGAAATAATTAGTGTCAGTACACAGATCTCTGAATAGCAGTTATCCTATAGGTATAGTCTTTACCAAGAGAACTCATCTGTAAATAATACATTTTGAGATGTTGCATGGAAAAGATGTGACAATTTAGAAAAGCCTTGAAGAGATCGGGTGTTAAAATTTCACAGCTGGATTTCGCCATAGGTTTTGGAACCACTGATAATGGGACCAGGTCTTCTGCTGCCTTGCCTCAGCAGCATGGAGTACCACATCCAGATGTGGAGTCCTCAGCTCAAGAGAGATGTGGACCCATTGGAGCACATCCAGAGGAGGGCCACAGAAATGATCCCAGGGATGGAACATCTTTCCTAGAAGAACAGGATGAGAGAGCTGGGGCTGTGCAGCCTGAAGAAAAGAAGGCTCTGGGGAGAACAGAGAGTGGCCTTTCGGTATCTAAAGGGGGCTGTAAGAAGGGGACAGACTCTTTAGTAGTAGCCCCTGCTGTGAGAGGACAAGGGAAAATGGTTTCAAACTAAAGGAGGGAAGATTTAGAAGTTTTTTACAGTAAGGGTGGTAAGGCACTGGAACAGGTTGCCCAGAGAGGCCATTCAGAGTCAGGCTGGATGGAACTCTGAGCACCCTGATCTAGCTGTAGGTGTCATTACAGGGGAGTTGGACCAGATGACCTTTAAAGGTCCCTTCCAACTCAAACGATTCTATGATTCGATGATTCATTACACTGACTTATGCAAGCTTGATGACTTGACTCTTTGAAAAGTCAAGTTGTTTCCCAAGAACAACTAGTTCAAAATTAAAATGCATAGTCCGTGGGAAATTGTCTTAGTTTCATGTGGGACAGAGTTGTTTTTCTTCATGGTGTCTGCTGTGATGCTATGTTTTGGTTTTAGGAGAAAAACAATGCTGATAACGCACCGATGCTTTTGGTTGTTGCTGAGCAGTGCTGTATAGAGCCAAGGATGTTTCAGTTTTCTCAGCTCCTCCTATTGCCCTTCCAGCAAGGGAGATGTGGGGCACAAGGAGCTGGGAGGGGGCAAAATCGTGGAGTCATAGAATCATAGAATGGCCTGGGTTGAAAAGGACCATAATGGTCATCTAGTTTCAAACTGGCCAAAGGGATATTCCGTACTATATGACATCATGCAGAAGGAAAGTTTGAAAAAGGGGGGAAGTTCATTGGACCAATGAACATGGATATTGGTTGGTGGGTGGTGAGCAATTGCTTGTGCATCACTTGTTATACATATGTATGTGTGTGTGCATTTACGTATATACATAACACATATTTGTATATATAACTGTCATAACTATTATCCTTTTTTCCTCTTCCTTTTCTCTACCTTACTAAAGATTTCTTACCTCGATCCACGAGTTCTACTTTTTTCCTGATTCTCTCCCCCCATCCCACTGGGAAGGAGGGCAGTGAGCAAATGACTGTATGGTTCTTAGCTACCTGCCGGGCTAAACCACAGCAGAAGTGCTGGTATTTTTATGTTTGAATCTATTATAAATACCAGCATTCCTTTCAGAAAAAGGATTAGAAAAACAAAACTGGAAAAGGAGGCTGTGTTCTGAACTAGCTCAAGATGAAAACTTCTTTTGAATACTCTTGTTTTGTGGAAATGCATTTTTCTGTCCAAAAATGCGTTATTTCTGTAGGAGAATCCCAACTACCTCACCTCCACTGTCTGAAACTGAAGGTGTGGGTACAGACAGCTGAGTGGAGGAAGGTGGGTGAGAGTCATGTCGCCTCCCCAGCTGTGAGCAGGCCGGCAGAGCCTCCTACGCCGAGACAGCTGCTGTATGGGGCCGCAGCTCTGGCAGAATGGGGTCAGTGGGTGGAGAGGCTGCCGGCTGCCAGGGGGAGGCTTTAGAGAGACCAGCTGAAAGAAGCTGTGGAGGGAGCAATATGGAGTGGAGCCTGTCAAGTAAGGGAGTAGGAAGTGACTGCAGGGAGCAAGGGACAGAAAAGCTCTCAGTTGCTTAGCGTGTGGTCATTAAATATGGAGGAAAGATTTCCTGGCCTTCTGCCTTGTAAAAAGAAATAATAATAATAAAGAGAATTTTCCTTGGGGGAGAAGAAGCAAAGAATTAATTAAGCCCAGACTTGAGATGGTCTCCAGTGTCTCATTCAGTGGCATTCCACTCTATTAATTTTGCCCTCCACTGACACTTAAATACCATGCCAGCTTTTATCCCAGATTGATTTCTTTCATCCCAATGTAAGGCATGGAGTGGAAACATCTGACATATTGTGGCAGCGGAGAATGAGAGTCCATCTTTTTGCTGGTGCCTTTGACTTGGAATTTGGAGCACAGTGGTGGGAGCTTGAGCCAGGCAGGAGCCAGCTCCTTGTGATGGCAGTCACACCTGCATTTGGACTGCGCAAGTCCAAAGGAACGTTTGATTCATTCTGCACAAGAGGAGGTGAGCCCTGATTTACCTCAGCTCTGTAGTGTGAACATTGCCTAAGATCCACCCAAAACACCTACACAGCTACTTCCAAGAAGCCTTTGGAAATCCCTTGTGTAGTCAGGGTTTTGCTACCAGTCCAGCAATTTGAGATCCACTTCTGCTGATCTGTAGGAGAGAGGCGCCAGGAGGTTTCGGTTTCAAACTCAGCTATCCTCCAGCTCCCCTCAGCTGTGGGGACAGAACTTCAAAGGAAACCCAGCCCCTGCCCTGAGTTGGGACTCTGGTGATAGTTCATCGCTTTCAGTCTGAGTATGTAGATGGGGTTAAAGTAATCCCTGTTCTAACTGCTCACCTATCTCATTTTTCAGCACTGTGCCCTCTGGGAAGTGTGACTGGCTGAAAGAAATGCTCTTGGAAGCTTAGCGTTGTGACAGACAAGAAATTGGATGAGAAAACAGAAACAGCTTAGAGGGTAGGAATGTGGATTTGACAACAGCATACGGCAAAAATGATCTAGTTCCAACGATAAATGCCTATCTCATATCTGAGATAAACTGAGGTTTCCATGTTTCCCAGATTTTTCTGGCACTGAAGTGCACCACGAGGCCACGTGGTGAGAATTAGCTCAGTGCCTTGAAAGTTGCTGGGTTACACCGACTGCTACACGGCCCTTTATGCGCGTAAAATGTATCGTATGTATTTTTCTTTTCTATATTGAATTAAGTTTTCTATATTACATTTCTCTAGATTGTTGTGTGGTTGGTTGGTTGGTTGTTTTTTTTTCAAACTGAAAGTTCAGCAACTCTCATTTCATTGTATTCAGCCTTACTTGGTTTCAGGCTATGGCAAAGACAATTCTTTTTTCCTCTGTTATGTGTGAGTCCCTTCTGACACTGAAAACATTACATTGTCTTCATAAGGAGTTGGTGCTGTTTATTGAACATAAAGCTAAGGGGTGCACCAAAACAATAATAGTAATAATACAGTATCTGCAGACAAACAGCTTTTCCGACACAAAACAACAAAATCAGCTATTGCCATAAGTATCAATTAATGCTTTGAAGATGCTTTAATTGCACGGTATCTATTTCAGAGCATTGAAATGCAGAGTTAATTTGAGCTAAAAGAAGATATGATTCACAGTCAAAATGTAAGCTGCAACTTGACAAGGGGTTTAATAGCTGCCTCAGGCAGATCAGAATGTGTGATAATGAGAATCCAGCAGAAAAACATATTATGAACGGCAGCATTTTCCAGGGTAAGCAGTGTCAGTGACCAGACAGCAGCGTTCCCTGTGGGGCAGAGGGGCAGAAATGGCCACTCTGGGTAACGGTGACTGCTCCAAGCTTTCAGTATAGCTACAAAACATCCCTTTTTG

Novel00254

GGCGGGGAGCGGCGCGGCGGGAGCGCGGCCATGCGGGGAGCGCGCGTCTCTGCCGCACAACGGCGGCCCCTGCAGGAGCTGCGGCTGCAGCAGGGAGCGGAGCGTACCAACATCACCCCGCTGCTCCGCCGCTTGCGGCTCAAGGACCACGATGGAGGCACTCCCGCACCCCGCGCCCGGTGCCCCTCTGGCCGCGTTGCCCCGGGGCTCCCCCGCACTGCTGTCCCGTGCGCCCCCTGCAGCGCCGACCCGGAGCCCCTCGGCCCCCTGAAGATCCCCTCAGGCAATGCCACCCCCGACCCCATCAGCATCTCAGAGCCCACTGGCCACGCCACCCCGGTTACAGATGCCCCAGAGCCCCCGGGTGACGCCACCTCAGGGCCTGACACCCTGGTGCCCCCACGTGATGCTCCCCAAGAGGACAGCCCCCCAGAGACCCCCAGCGGGGCCCCCCGAGAGCCCCATGGCAGTGCCCCAGTGCCCATCTTCCCATTCTCCCCCAGCCACCTCCCCCCGCTGCCTCCATCTCCGGACCACCCCAGCAGCACTCCCTTGGAGCAGCGTACTCCTGACCCTGTTGGCAGCACCTCTATAGGGCCCTGCGCTCTGGACACCCCTGGCAGCCCCTCTCCAGGCCCCCCCGGTACCTCCACCCCTCAGGACACCCCGTTCCGCAGGTGGAGAGTGGCCCCCCCTGACCTCTCCTGCAGCCCCGTGGCTTCCCAACCCCTGTCTGAGAATAGAGATGTGGGTACTCCTCCACATCGCAGCACCCTGGGGATGGCTGGATCCCCGGGCACTGCCCCACCGGAGCAGTGCTCCCCCCAGGTCGAGGCAGTGGGCTCCGAGCCTGTAGAGTCAGCAGTAGCAGCAGCGGCCACCCCGGTGCCTTCGTCTGAGCCGTCCCTGGGGGCTGTGTCCTGGATAATGCCACTGGTGTGGCTGGAGAGGACCCTCCCTGCCTCGTCCCTGTTGGAGTCCCTGAGGCACAGCCTGTCCGTGTCGTGGCGGGATGCTGGCACCAACATCACCCCAGTGCCCACTGCAGCTGTCAGTACCGGAGTCACCCCAGTGCCCACCATACCTGTGGGGAGCTGTGTCACCCCAGTGCCCACTGTATCTACGGGTACCACCGTAACCCCGGTGCCCACCATACCTGTGGGGAGCAACGTCACCCCAGTGCCAACCATGGCCACAGGCACCAGTGTCACCCCAGTGCCCACTGGATCCATTGCCACCAATGTCACCCCAGTGCCCACTGGATCCATTGCCACCAATGTCACCCCAGTGCCCACTGGATCCATTGGCACCAGCGTCACCCCGGTGCCCACCATGTCTATGGGCACCAGCATCACCCCAGTGCCCACCATGTCTACGGGCACCAGTGTCACCCCAGTGCTCACCCTATCTATGGGCACCACCATGACCCCAGAGGAGAGGAGCGCTGGCACATCCATGCCTGCTGGTGCCAAGGACAGCGCTGCCGAGACCGACTCTCTGCTCTGGCACTGTCCCCGGGAGCAGCTGCGTTCGCTGCCACGAGCAGAGCTGGAGGGGCGGCTGGAGAGCACCCTGATCATCATCGAGGCGCTGTCACTGCAGCTGCGTGACTGGCAGGACTGCCAGCGCCCGCTCCCTGCTGTGGGGCCGGCGGGGCAGAGGGATGCACACACCCAGACTGATGTCACCCGGCCCCGGGGGGAGGAGGGGATCTACCACAGCCTCTACGTGGAGCTGCAGAAGAAGGTGCAGGCTGTGCAGCGGCAGCGTGGAGCAGAGCAGGAGTTGGCGCAGCAGCTGGCACGGGCAGCGGAGGCAATGGATGCATGGGCCGGGCAGCGCCGTGCGCTGCGGGACGTGGCAGACGCCGCTCTGCAGGGCGTGCAGGATGAGCACGCGGCGCTGGAGCAGGAGAGGCTGCAGGTGCGTGCCATGGTGTCCCGGTGCGAGGCAGTGCTGCACGGTGTCCCTGCCAAGCTGCAGAGCTGCCTGCAGGAGCGGGATGCTGCACAGCAGCGAGCGGATGAAGCCCTTCGTGCTAAGCAGGAGAGTGACTGTTTCCTGGAGGCGTTTCGCTCCCACGCTGCTGCCCAGATTGGTGCCCGCACGCAGAGCCTGGAGCTGCAGCAGGAGCTGAGCGCACTGCTGCCCTGTTTTCCTCTCCTCAAGGTGTCCCTGGCTGCAGAGGCTCAGCCTTTCCGGGAATTCATAGACGTGACCTTTGCCAGCCTCCAGGAGGAGCGGGGAGCGCTGGACGGAGAGGTGAGGGCTGTCCCAGCAATGTGTGCTACCCACCCCACCAGCGCTCAGCTCAGCTCAGCCCAGGGCTGTCCCGTCCCTTTGCAGCGGGAGCAGGTGCGTGCCCTGGTGTCTCGCTGCATGACTGCGCTGCGGGACGTCCCCAGCAAGCTGCACAGCTGCCTGCAGGAGCGGGATGCTGCGCTGCAACGGGTGGAGGAAGCCCTCCAAGCCAAGAAGGAGGTGTCCCAGCAGCTGGAGGAGACCCTGGAGGCTCTGCAGGACGCCGTGGCCCAGGGGGAGCAGCTGGCAGTCACCAACTCACGCCTCAGCACAGATCTGAGCACGGTGATGAAGCAGCTGGCCAGCCTGCAGCAGGAGCAGGACGCGCTGCAGCAGGACTACGAGGAGCAGAAGGAGAAGATGAGCTGGCTGACCAAGGAGAGGGATGCTTTGCAGCGGGAGCGCCGGGAGCTGCAGGAAGCAGTGGAGTGCCGGGAGTTTCTGGACCAGGAGAACCGCATGTCCCGCAGGCAGCTGATGGAGGTGGAAGCCAAGCTGCAATCCACGCTGGCTGAGCTGCAGGAGCGCAGCCTGCAGCACGAGGAGCTGCTGGAAGCCCACCAGAGCCTGCAGGAGGAACGAGCCGCCCTGCACAAGGAGCTGGAGAGCACCAAGGCGGAGCTGCAGGACCTGCAGCTCAAGAGGGACAGGGTGTCCTGGTGCTCAGCAGGCATTGCCGAGAGCAAGGCGAGGCTGCAGGAGCTGGCTGACTGCCTGCGAGCCGCCCTGCCCCACCAGGATGACGATGACGAGGTCCCATCGAGGAGCAAGACCTGGACCCCAGGCTGGAGGACCCCCCGTGGGCCCTGGACCCCCCGTAGCAGTGCTTGGACCCCCGTGTGCCGCACGCCTGCCCGCCACACACCGCACCACGCCAGGGCGTCCTTTGTAGGCAGTGTTCTGAAGGCAGTGTCAGGAAGAGATGGTGATGAAGGCACTGAAGGTGAGAGCACATCTGCCAGGGATAGGGCTGCATCCACACCGAAGCCCCCAGAGCCCGAGGACGGTCTGATGGAGCAGGCAGCCGAGCTGCGGGCCGTGGTGTCCGACCTCTCTGCGCTGAGCTCCCGCATCCAGGAGTTGGAGCAGAGCGAGTTCAGGGCGCTGCAGGCGGAGATCTCTGACCTGCAGCTCCAGCTGGAGGAGGTGACGACTGAGAGCCAGGAGCGGATGGACGCGCAGGCTGCCACCATTGCCAAGCTGAACAAGGTGCTGCAGGGCAAGCTGCAGAACGAGAAGGAGCTGCAGGATGTTGTGAAACAGCAGGAAGCAAAGATGCTGCAACTCATCGATAAGAGCGGGGAGGTGACGAGGCTGAAGGAGGAGGTGACGCAGCTGAAGCGCTCGCTGCAGCGTGCAGAGACTGAGGCCAAGGTGCTGTGGGAGGAGATGAGAGGCCAGGAGGCCAAGGGGGACGCAGCCCACGTGCAGGAGCGCGTCCTGCTCCTCTTGCAGGTGGACAAGCTGCGCTTGCTGCTGCTGGAGAAGGAGGATGAGAAGGTGGTGGTGTCGGGGAATTACCTGGAGCAGGTATGGGGTGGGGAGGGGACGCTCTGGTGCTCCCTCTGCTCAGGGCTGTGTCCTTACGGCCTCCCCCCATTGCAGGTGCGAGGGTTGGAGCTGAGGCTCAGCCAAGCACAGAAGATGCTGCGGAGCCAGGAGGAGCTGCAGGAGAAGGTGAAGGAGGTCCTGCTGGCCCTCCCCGAGGTGCCCCCGGAGCTCCGTGCCCTCCTGCAGCACGTGGGGTTGAAGCCAAACAGCGAGAAAGCTCCGGCTCCTCTGTAACCCACAGCGCCGAAACGTTCTTGTGCTTAACGCCGTCGTTATTTATTGAATAAAGTTTGTTGGCATTCACCCCATCCCCTGTCTGGGGACACCCCCCCTATGGAGCCGTGCTCCCCCCCAGCACTGCTCTCAATAAGGTTCCGTGCTCCCAAAGCTCCCACCCCATCTGACCCAGCTTCCCTCCCCAGGGCCCCCGCTCCTCCTCCTGGCCTAGATAGATTTCC

Novel00197

GAAAAATTATGAAAATGAATATAGTACGCACGTTTGTGAACAACGGTTTTCAAGCACAGGAAGAGTAAAATTTCATCAAAAATCTCTGATGAACACCTTGAGAGCTCAATTGCAGCTGCTGCCATCGAACTGGCCTGATGCACCAGCTTCACAAAAACAAGGTCATGTGTCCCACTAGATTTATGTTGTTGTTGCTTACTTGTTAAAATAAGATTTGTTACTTATATGCATGAACTATATTTTATGGGGGCTGCTCTGAAAGTAGTGCTTCCTGTATTATGAAGTTGGCCCGTGACGTCAGAGGCGGATGTTGGTGGGATGGCAGCAGAGGTTGAACCTTCCCACCAACATCCTGTTATTGTACTGCCATGTGACAGATGGCAGCAGAGGGGCACTCTGACAGAATGGTGTCTGATATGGGAGTGTGGATGAAGCAAATATGTATCACTGCATTCCTTCGGGAGCTGAAAATGGCTCTCAGTGACCTTCATCAATGCTTGCTGAATGTTCATGGAGACCAAACAGAGGCTGTGACTACAGTGAGGCAGTAGTGGTGCATTTCAGCCACAGCAACAGTGGGTCGTCTCTGCCGGTGCAGATGCAAAATGTGTTTAGTCATTAGAATAGGTTGCCTGTGTTCTCTTTTTCATCTCCATTGTTTTGATAATATTATGAAGAGTCTTTTAGATATAAAGTGTTATTTTTTAAAATTTCCATTATTATTTCTGATTGCTTGTTGAGCATCTAAAATGGTCGCAGACAGTGTGCAGAGAATATAAAAAACACAATCCTTACCCGAAGGGATTTGATCTAATGTTAGATGGTTGCAGAAATGGAGGCTAGCACTAAGCCATGAAGTGAGATAGAGAGGAATCGCCTTAAAGTAGGAAAAAACATTGAGCTGCAGTGAAATTTTAGTTCATGTTGTTTATCCTTAATTTGTTTTCAGTAAAAAAAAAAAAAAGGATTAAATTCTAATAAATGTGAATTTTAATGCAAGTTCACTGTTTTTATTCACTGAAAAATGAAACAAAAGTGCCTCAGCATTTTGTCCTATTATATCCTTTGCCTAAAGAAGCAATAGTAAGTAATTGAGAGGAAATAAAAATAAGTTCTGAATATATTACAGCCTTAGAATAACTGAACAAATTTGGTGTTCCTTTCATGTCCAATATCCTGCACTAAGAGCTTGCTGTTTTTTTCTGCGTTTGTTTTGTTTATATTGTCTGCATGTGTCTGCTCTGTTTGAGTCTAATATACAGGATATGAAAGTAGAAAACTGAGCATAATGCTTTGCACTTTATTATTTTTCAAACATCTAAAAGGGGGTACTAAAGGAAGCACTTCGGTTATTTCTTAGATTTACAAGAGTCTGAGAATTAAAAGGAACAACATTTGAAATTGTAAGCAGAACATTTTGTTGCTGATCACGTAGAAAGTTTTCTCTCATTTTTTTTTTTTGGTAGCAGCAAGCACCGTGATAACATAAAACAATGATCTCTCAGTTATTCTAACTTTTACCTTTTAATGGGATGCGATCTACTTATCTCTGTTTCCATGAGGACCATGGATGGTTTCCAGGAGTAATTTCTGTCACTAAAACTCAAAGCATCTCCCCTCGCTATAGCAACAAAAATCTCTCATTAGTTCAGTATGTAATAGATATTTGGAAGTCAAGTAAATAGCTTTCAGTGGCAGAGTTGAACTACACAAATGCAGACGTTTTTCTAAAATTTGCTTTATTTCATGGCACAGCCCAAAAAATAGTTTGCCTTTTTTTTTC

Novel00178

AACAGGGACTCAGCAGAGAGGAGGGAGGAATCAATCCTTGAGCACGAGAAGGCTGGAGGTCAGAACCAGGAAAGAGAGGATGCACCCTCCTCCATCCATACTTTTATACAGTGATCAGCAATCACCATCCATTCCTCGGTGGATTGTGTGCATCTCACCCTCTCCATCTCCTTGCTCAGGCCTGGGGCTGCTTGCCAAGGGCACGGGGCTTCCATGCATACCTGCTTTTCTACAGACATCTGAGTAGAGAAGAGATAGCGCCGGCAAACAGATGTCGGCAGCTCCTCGCTGGGGAGCGGTCCCTCTGGACGCGTCGGGTCGGCCAGGAGCACCGGTGCCTTTCAGCATTGTCCTAAAACTCCCAGCCCGAACCGTGCCGCGGGTTCCCACAGCTGCGCTCACAAAGGAGACACAACGGGCTCCTCTGGGGACAAAACCTAATCTGAGAGATACCAGGCTGAGAAAGCTCTTCCTAAAAAAGACACAGTCAAAAGCTCTCGGATGCCCCGCGTCCGCTGATACCCGATCCGGCCGCCTGTGCTACCATTACCTCCAGGCAGGCTTTGTCCGCTGCGGCTCCCCTGAATGTCACACTTGTTCCCAAATACCAGCGGGGAACAATGCAGGGGCAACGCAAGGAAAAGCCTCCTCTGTCCCTCCCAGCCTGAGAGTAAATATCAAATGCCTGTGCTGAGGGGAGGGGGGCGGAACAGCGAGCAGGGGGACGAAAAGGAGAAAGTCCACAGTTGAGACACGTCACAGTGAAAATGGGTCATGGCGATAGGGTGGCAGGCAGAATAGACCCCATCCCTCTGTGCTGGGAGGCCTGGTGAGCAGCTCCTTCACACACAGCCCCAGCATCCTCTGCCCCATGGGGCATGAGCTGGATGCAATCTGGTTTTCCATCCCTATCTTCTCTCCCCACTGATGGTGAAGTCAAGTCCTTTTCAGCCCCCTCCTTCCTGCAGGCTGCACAGGGTTTCTTCCTCTCTTAACGTGTCCCTTTGTCCCTTGAAGGGAACGTGTTTCCAGCTGAAAATGCCAGCAGCCCTGTCCTGTCCCAGATGGGAACATGGTCCTCCCACGTCCCTGTGCTCCCCAGAGCTCAGGCAGTGCAGGCAGGAGCCCGGCTCTATCAAAAGTTGTTGGAAATGCAAACAAACACTGCCATGAGCGCAGTGGCTATCAGCTGTGCATCCTCTTTCTTGTCTGAGGGTGAAAGGGAAAATCAGCCCAAAATATGAATTTCTGCCTGCACAGCTGTCCTTGCCCATAGACCAATGGAGGGGCTAGCAGGTCCATGGCTTGGACATTGCCTGGAGCAACGGGACAGGAAAATTGTATTTCTGACCCAAGACTCACTGATGTTAGAGGCCGATTTCCCAGAGATTTTGCCAGGCTTTTATGCAATCCTGTAATGAATCACTGGGATCATGGAAGGAGGCAAAGCAGCAAGAACAGAGGGCAGGTGAGCTTGAAAAACCATTCCAGCTGAGCACAGAAGACCAATATGAAAAGTTTTATTTCAGAGAAATTGGAGAAGTAAGGGCTGGGTTTGGGGTGTCCTGTTCTGAACTAACACAGATGCTAGGAAAAGACATCTGTTCTCATTTCATCTGGTATATACAGCAGATACGATTCAGAGGGATCCCATTTGGGTCCTGGAGACAAGGCTGGACACGGTGCCATGTTATATCCTTGGTGAACCGTAATGGGAAGTTTGGGAAGTGACCCCACTGAACAAGCCAAGGCAGGGCCAACTGCTGAAACTGCATTTTGGAGATGAATAGAGTCTCATGTGTTGCAAAAGTTGATGGAAGACTTCCTGCAAAGAGAGGACAACCCAGGAGCAGCCCCATCTGTTGCCAAATGGCCACACAGGGAGTGTGACCTTGTCCCATAAGATAACGAGATTTATTCTGCTCCTTTTGATAGGACATCGATTAAGTCTTCCATGCTTGTGAGGGCACCGGGGGTTCAAAGCGCCAAAGGGGCAGCATGGAGTAATGCAAGACGAGGCTCCCTCGGCTTTGCCCAGAGATTTATGGATGCTGTTTGTCACAGAGCAGGATTGGAATGAATATCCAACTGCTTGTGACCAGAAAGGCAGACTCCATCCAACCCGCCGCGTGGAGATGAACTCCAAACAAGCCTTGAGACCCCTGGCACATTGGGAACCCAAACAGAGCGCTCTGCCAGCATTGGGCCCCGCTCAGCTGGCTGCTGTCCCAGTGCTGTGCCTTGCACTGCTGGAGTGATGAGTTCTCTGCACAACCAAGCTGGGGATCCACCGAGCCGGGAGGGTGTCTGTCTGTCTGTCTGTCTGTTGCACTGACAAACGGCCAGCGCCAGTTTTGACACTCATTCAAGAGAAATAGCTGTTTTCAGTGGACGCAGCCTTGCATTCCTCATGCCTTTAGAGTGAGATCTTCAGTTAGGGCAACCCAACAGGACATCACCAGCGGAGCAGCGTTGTGCTGGCGGTGCTCCTTGGGAAATAAAGCACAGCCACAGGTGCGACAGGAGCAGAGGAGCCCTTTGTGGGTGGAACTTGGTGCTGCTGGGAGATTTCAGCCCAATTTTGGGCTGGGAGCCGGGCCCACCTGTGTGCAGGAGGAGCACAGGGCCATGTGTGCTCATGGATGGCTGCCTGCAGGAAGCTTCCCTGTGCTATTTTTGCAGCGCTGACCTCACTGCTCGGAGCAGGCTGCCCTTCTCCAGCTTCCTCTCCTTGATAGCTGCCTTTTGCCCCGTATTTCCTCATGTGTTGAGCCATCACCTTAAGACTCTTCTCATTTCTTGAGTTCTTGTTTATCTCCTCAAGCGGCACAGGGCTCTGACGGGGCTCTGCCTTCCTGTCCTGCCTTGCCAGTTCCTCTGTTTACCTATAACACAAATTAAGCTTTTGTTTTTTGTTTTCTTGTTGTTTTTTTTTTCCTTCTGTTTTGATTTGTTTTCTTTTTAACCCAGAGCAACAACTTGGTACCGGGAGAAGTGTTCTCCTCTCTCCTTTCTCCTCGTTTCTCCACGATCCCCACCTCTTCTCCAGTTCTTACCACCTGCCACAAGCATTCCTTTTGTCTTCACCACAGCCAACGAGCCCCGAGCGCGTCCCATCGTCCCCACTGCCACTGACAGGGAGGTAAAGGCTTTGATCCCAAGGGGACTCGGAGGGGAAATGAAAATCCCTTTGCTCCCCCACCTCCCTGCGCTCCCCCATGACCCTTCTCCCCTCGGCGGTCCCACACTGTTCCCTCACAAAACAAATTCTTCCTGGATTATATTTTAATATCTGCAAAGCCTCAAGTTTGAAAGAAAAACAAATGTGAAACCGGCTCTTGAATTTCCTTGATGTGAAAAAAGAGAAACCTCCTCCACGGCTCCTGAGAGCCCTCCTAGGAGCCCCATGAGCGGATTAGGGTGGGCAGAGCTGACAGCCACGCACTCCATCAGACCGGGGATTGAGATGTCAGGAGTTTAAACCAAAAAGAGATGGGACAGGCACGCAGTGCAAGGGGAGAGTGTGGTAGTGAGCATCTCGGGAGCCTCTATCCCCATGCACTGCTGATTTGTGTCCCTGGGGATGCACCAGGAGTAAGGTCCTGGGGAACACCTGCCTGTGGTAGAGGCTGCAGTCTGGCTTTTGCATTTGCCCCAGTTTGGGCTTGGGGGAAATGACATCTTGTCGGTGCCCATCCCTCAGACTGCTCCCTGCTCAGCACAAGGGCTCTGTGCTTGTTCCAGGGTAAAAGCCACGTTATTCTGACTGTACCAACCAAGAGAGCTCCTTTTTGGTCAAAGTCCTTTTTCCAGCAGAGGGAACAATCACACACAGGTGGTCCCCAGCTGGGAATGGCTGCTCATTCCCCGTTTGGTCAACACCTGAGATAAACCCAAAAGAGATGCCCTTGTCATTTTTAGGGCTGCTCTTTTGAGGTCAGGGAACAGGGGACACTCGCTGTGTGCGGGACAGAGGCTGCAGTGAGGGGAACGATCGCTCTGCTGAGGACGCCTTGTCCCTTCCAAGAGTACAAACCACGTTGCCTCCACCCTTCCTGATGAGAACCTTTTCAAGCTGCAGGGAGTGGCAATAAAGAGTTTCCTCGCGTTTTGCTAGCAGGCTTTTGGAGCAAAAACATCGACCCTCCCCAGGTGACAGCGGTGTGCAGAATGACAGCATGTTGTTTCCCAGCCCATGGGACTCCCTCCGCGTCTTGGCTCCAGGCAGTGGGTGGCATGGGCTGCCCACGAGCCCCAGGGTACCTCCTGCAGTATTTTGATTTGGATTCTTCTTTGTGCAAATAAATCCAGGTCTGAAGGCTCTGGGTGATGGGAACATCTTTCCCCATCACCTGGCTGGGTTCTTTGCACTGAGCTTGTACGTATGGGAGAGTAATTCCAAGGGGACTGCTCAGACCTCTGGATGGGCACCGCACACTGGGCACCTTGGTGGGAGGTTCCAGCACATAGAGATGGAAGCACAAGTGGGGCCTGGTGAGCCTGGAGTCAATAGTATCCAAGAACAAGAACATGGGAAGAAATGTTCTGAACTCTCCTCTTATTGCCACGTGGATTTGTTCTGGCTTCTCTACGTTTGGGGCTGAAAATATTCTCTTCCCAAAAGGATCACCTGTTGTGGGTTGGCCACCACAAGGCCATCCCATGGCATGGATGGATTGGAAGGAAGGATTTGTCATAACAGGCTGACTCCCCAGAGGCTTAAAAATACATTTTTAATGAAGTTTAAGGGGTGGTTCTGAGTTCTCCTGGACAAAGCAATCTCGCAGCAGCTGGGCTGAAACACAGCAAGAACAAATACTGACTTATAAGTATTCCACTTGCATCTATGAAGAATTCTTCCTCTCTTCTTCAAGTCTCAGGCCTTTTCACCTGGAGCCTTTC

Novel00075

CCTCTCCTGACTTCATTTTTCCATTTTTCATTTGATTGGAAAGATAAAACTCACTGGGAGTCATGAGACTGCCCCTCTTTGTTCCCTGTCTGCTTTGCCTACAGCATTAGAGTAAGAACTTTGGCTTCAGAAACTCTCTGTTTCTCATTTTATCTGGTTTATTAGCTTCAATTCCAATCATATTGTATTATATTGTGTTATTTCACATTCAACTACTATATTTAGTAAATCAAACTTTTCCGTAGCTCATTGCTGCTGCTATCCTTCATCCAGGCCCATCTCCCTACCTTTTCCCTTCCTTCCTCCCCCCTTTCCTGGGGTGGGGGTCTGTGGGTCCCTTCACCCCCTTGTCATGGAACCTAGGCCAAACCAGGACCATGACAGCAGTACACCTCTTGGTTTGGTGCTGTGCACATATATCTGCTGAAGTAATTGATCTTCTGTGGTAAGAACTTAATAGAGTATTGATGGTATTTCATGCGAGGATGCGTCAGTGAGAATGGTATGTAAATACATAAATGTATAAAACAACAGGGGTAAAACAGAAATTCATAATGAAATCCCTGTTAAATAAACACACGACTTCAGAAGCAGTTGAAAAGTTAGATAAGAAAATGGCGTTGAAAGTGAGGAAAGGAGAGTGTGATAAATGGAAAGGTTACTAATGAAGGAAAAAGAACACTGTGCCCTCCTTCCCCACCTGGATGTGGTGGAAAGGTTTGCAGGGTGCAGTGACACTTCAGTATAGGCCAGCAAATTATAAAGCTCCCAGACGATGACTCACACCAGACAATTCAGAGCACAGGAGGCAAACAGACTGAAGTTAATCCACAAACCCTTAAAACAATGGAATATTGCTGTTCCTCTCTTTGCAGTCCATGTTCCATTTCTTTTCCTCTCCTTGTCATTGTTGGTTTGCCCTATCTGGACCTCTCAGCTCTCCATCAGTGTTGGCCAGTACTGCCCTGCTTTTTATAGCATCACAGAATCAGAGAATCACAGAATGGTTTGGGTTGGAAGGGACCTTGGAGATCATGTAGTTCCAACCCCCCTGCCATGGGCAGGGCTGCACCCACTAAGTCAGGCTGCCCGGGACCCCATTCAATTTGGTATTAAACACCTCCAGGGATCAACCTCAGCTTCTCTGGGCAGCCTGTGCCACTGCCTCACCGCTCTCAGTAAAAAAATCTGTCAGTAAAATAAAAATGAAAATGAAAAAAATCTGTCAATTAAAAAAAAGCAGTCGTTGCTCTGAACCACCACTAGTCAGTGTTATTGTCTCATATTGTCTATGTGCCGCTAGTCTTCACTGTCACCCCTAGCCCCTCCTGCCACCTCTTGTAACTCTATATCACCACCTCAGCTGGACCTACTGAGCTAATGTTGCTATTCCCTCTTACTTCTCACTTCCTCTCAGTGCAACATCCCAACACTTCGGGCCGTAATCCTGGTTTTGCCACCCGATGGTCTTCACTACTGTTAAAATCCCGCTCTCTGCCTTTTGTTGTTCTCCAGCTACTTCTCTCCTTCCCATTCTGCTTTGGCACACCTTGCCGTTTTGACCTATTACCCAGCACCGTTGCTCTATACCAGCCCCTGCTGCAACTGTTTTCTTCTGGCTGCCCAAAAGTAACACGCTGTTACTTCCACGGGGAAGTTAAGGTTACGGCAGAAATCTCTGCGCCTCTGCTCCAGTGTTGACAGAACAGATCATCCTGGCAGAACCCATTCTTGTGCAGGGTGGGGTTGGTCTATAAATGTGCCTGAAACTGAAGTGACTGTGAAAAGGCCCATCAGCACGGTGATGACGAGAGCAGTGACAGCAACTGCTAGGAGCAATGGCATTTATCCAAGGGTTTTAAAGCAAGTCAAGAGTACAATAGCTGAGCTGCTCCCAGCATTGTGTGACCCATGCTTCATTTCATTGTGGCACCTGGGGAGGGGAAGGCAGTAAATGTGATGCCAGCTCTTAAAGACTTTACGGGGGCACTGGATAAAAATGGACGGCTTCAACCCATGGAGGTGACTAAACCACGGAAAGCACATTCATGCAATTATAGAATTATAAAATCACAGTCACAGAATGTCTTAGGTTGGAATAGACTTAAAGATTCAGCCAGGAAGTCCATTACCTGAAAATGTTTGAAGGCTGGGAGAGTATGGCTAAGAACTGTGATATATTTGCCCCGTTCTTCCTCTTCAGCTTGAACTTTCCCTGAGAAGGGAGAGGAGGAGTTTAGAGAAAGAGAAATCAGGAAGAGATAAAATGATTATGGTGATGAATGGATAAACAGAAGGAGAGAATAAGGACCAAACAATTCAAAACACAAAACAGAGCACAACAACAGTACCCACTGCTTCCTGAAGACATCTGAGGGTGCCAGCTGGTGGATGCTGTGCTGGGATCCTCTGCAACAGAACAGACAGCAGAAGGGAATAGAAACATGCTGTGTAATTGCCAGGACCACCCTTACATCATGGATCCTCCTCCTGGAGCTGCAAGTGGCCAGGAACAGGTGGTGGAGGAGGTTCTGGGGCTTCTTGAGACTTCAGATGAAGATGCCATGAATGAAAAGATGAGGGATAAGTACAGATAGAGTCTAAGAGGGTCTGGATAAACTGGAAGAATGCCTAAGTTTGGCACACCACTTGATATCTTATGTCCCCAGTAGGCTGTGTGTCCATACTATCATCTACCAGCCTAAGTATGGTGTGCGCTGTGGTCCATCTCCTGAGGACTGAATATGTACTTGGATGAATCTTGCCTGGTAGCGTTGGAGCCACCTTGCAGAGTTTTTGGATCTTTGGGTGTTATGGTCCGGGCACTTTCACCCAGCTGCTAGTGAAGAGGACAGCAGCAGTGACAGAGGGGTGTGTTTGTGTAAGCACCAGGAATCTAACTGTGGGTGGTGGACCTCTCCTTTCTCTTGTTTCAGGGTTTGTAGGGGCAGGGTATGAGAGAAGGAGCTCCTGATGCTGCAGACATTGAATCCAGATGTGGACATTCCTCTGCTGTCCACTGCTGGGGAGTGAGAGATTCACTTCATGCGTCTGCACACGTGTACCTGACTCTGCAAGAATTCCTTGTTGCCTCTGGGCACAACTCAGCCCCACACTGCTTGAGCTACCATGGGCCTCATCATGGACCACTACCATGGATCTCACCAAGAACGGCTGGCAACTGCTTCTGGTCCAAAAGACAGGGCTGAAACCATCCATCCTCAGTGCAGGTCAGATCTGTGAAGAAGCTGCCATTGTATGGTTGTGAGATGGTCTTCTCTACAAACAGAGGTACCCAAGAAGAACTGTGAGGACTGCAATGCCAAGCTCTTGAAATGGAGATTCTTGCATCAAGCTTCTGATCTTCAAGACAATTACAGCAGGAGGGCATGAATGCTTGCCCTGATGCTTTGTCCCATCCCACCCAAATCACATGGGTTGCAGTGATTCCTTGCAGCTCATTGACAAAGACAGGAAGATGTGGGGAGGGCTGGTGTTCCTTCCAGGGTCACTGTGGAAATCTGTTTCTTGTTGCACACTAGGATCAAATGAATAACTGTCCATTGTTGCCTTGTGGACTGTATGGAATATGTGACTGATTGATTTCTGTACATTAGGCCTTTTGCTATAAGTGTGTGAGAGGTGCAGCTGACTGGTGGAATTCGAAGCTTCTCAGCCTGAGCATGTGGTACCAAGGTGGAGATTAGGTTTATGAAAGCCAGTTCTCCCTGCTGTAGGGAGCTGTATAATCATAACACTTCAGGCACAAAAGAAGGATTCTTCCTAGTCAGATCTATGATGGCCTTGGAGATTACAAGTAATGACAAGCACAGCCTGGGGGAGAGTGGACTCCTGGTACTTTCAGAGATGTCATCCTCAAATGTTTCAAGAGTGACATCTCCGTGGTTGAAAAAGCAACCCTTGTTCATCCTGTCCTTGCTGTGGTGCCTTAAAACAGGACCACTGAATTCCAGTAGTGTGTATACAGCTGCTGCACGGCTGGAGAGGGCTGCACACTGGTGGTCTGTGTGACAACTGACCACTCATATATGTCATCTGCCATATTCCAGAAAAGGGCTTTGAATCAGCAAAGCATCTGTTCTGCAGTCTGGCTCTAAAGAAGCAGTGTCCCCAGTTGCTCTGAGTGATGATATGCTGCCAAGTGGAAGCAAGCTCAGAGTTTATTTAAACACTTTACAGATGCCTTATTGCTGATGCACATTTGCTTTGAAATGCATACCAGCCAAAATTTAGCTATGTTCATAAAGTGCATCTAAACAAATGTAGTTAGACTGCTGGATCAACGTTGCTAGCTTAAATTGATTGAGCGAAGGAATAATTAAGCAAAGGGAACCTCTGCAGAAAAGATAATCCTGAGCTCAGATTTACTCACAGTGACAACTCTTTCAATATCACGTTAGGAGTTTTTGCAGTCTTCATTCACAATACGACAGTCACAGCTCCTAAGTAACCATGTATGTAGAGAACTTGACCTTACACTTTGCAGGTTGACAAAATCCCTAACAAATCTCACGTCTCGTATCTCATATCAAATTCCAATTCCAAAAATTGGAATTCCAAAAATTCCAAATGAGCTGATTTAATACTTCTATATTTAATATTTATTTGTATGATACAGCATACCCTGTGGCAGGTTGGTCTGGCTAACAACTGCTCAGATGGTGACCAGATCCCTGTTCC

Novel00038

CTCCTGAAGCCTGTCGGTTCTCTGCAGGGGAGAGGCCTTCTACATAAGAGCCTAGAGGGGGCAAAACATTTGAGCCACTTTCTTGCAAGCTTTAGAAGCTGGAGGAGTTGAGTGGTGCCTCAGAGCTGGACTTCTTCCAGGAGCTTAACCATGTGTGCAGCAGCAACTAATAAACTCGGCAAGAAATGAGCCAAAGGCTCCTGTGGAGCCCTTAATTCAGCCCATGTGCATCTTCAGAGCTCTCTCCATTAGTCAGCTAAATTAGCTACACTCCTATGGGCAAGACACCATCTCTCAGAGGCAGCCCTTTGGGTCAGGCTGCGGGAGTAATGGCCATTGAACATCCAAGGTGTTTCTGACAGTTCTCTTAGAAACTTCAGGAAGCCTTTCTGGCGTCAGTAGAAATTGCTTGAGTGCTCATAAATTCTTCACTCAGACTTGTACTTTTGGCACTAGTCTGTAGCTCTACCCATTTGCTTTAATTGGATACCACTTTCTTCTCCTAATGGCAGACCTGAGAGGTTCCTTCTGAGAGGTGCAGACAGGTTGCTTGCTCACATCAAGACAGGGGTGTCACTGAGCACATGTGATGTGCGTGTGACAAGTAGCTGTCGTAGACCAGGGTGTTGGGTGGTAGGGAAGCATTTCCATATAAATGTTACCTAAAAACACATTCTAACTCTTGTACTTCTGGTGTCATTAAAATCTATGTGCAGTGGAACGGGTTTGTCAGAACTGTGGGAAGACAGACCAGCTAGGGAGACTTTCCCTGTGGCCCTCTTCCAGATCTATTTTTTTTTGTCCAGAAAATCCTTGTCGATGATACAGAGATTTTCATGATTAAAGCTGGGTAAAAGATTGTAAGGGGAATTAGATACTGTAATCAAAATATTCTGTGAAATTGGTCAAAGAACTCTATTCCCTGCAAACCTATTGGGTTCGGGTTTGTTTTTAATTGCCTGGCTGGCTTAACAGCCTGTGATCAAATCAGCAGAGATTTGGTTTTTTTTAAGTTACATGTCCAGAAAAATGATGGTGAAAGGAATGGGTAGGTAAGAAAGAATTTTTGATTAGTTTCAATCTGATGATCATCTGATTGTCCAGAGCAAATCTGGTGAGTGCAGTGACTGCCCTCCACGGGGGCAGTGCAAAAACGAGGGGATGGGGACAAGAATGGAGGCTGAGCATCCCTGTGCACTGCCCTCTGCCAGCCCGCTGTGCAGCTGTTGTGGATTCCCGGGGGACATGCTGACATGATGCAGCAAAGGCAGCCACTGACAGAAACAGACGGGAAAGGATGTGCCCCTGGTTTATCCTTGATACTCATTGGGGTGGCTGCTGGAAGTTTTGCTGGAGCTGTCCTTGATGAAACGTGTGGTCATCTGTTGACGTTTATGTTAGGGAGCTAAAACGACATTTGCCTTTTAGGAAATGCATAATAAAGCCTGATCTGTTTCTGTGCAGGATGTTTCTGCAGCTGGGTCTGCGCTCTGTGGTGAGCTAGTAAGCTGCTAGTAGGATGCTCACAAGTAGCGCCTGTGCTGCTGTCTCCGTACATACAGAATCCTAGCTGACTGCCACATACATTGCATTAATTACTAAGAAGTCAGACTGAACCTCTTAAAAGAGCTATCTATCTTCTGATGGAACAGCTGAGTTTTCCTCTGTCTCTCCTGTTCCTATCTGTACTTCCAGCAGTGTGATTTCCAGGCAGGGAAGACAAGGTAAAGTATGCTGAAATTCTGCTGTGTTCACTTTTAGATAAAACACGGATTCATGCTGCTGAAGTCCATGGAGATGCTAAAGTCAGTCATGTATAAACAGTGATCTATGATCTAAAGTTTCAGGAGGGACGAAGAATGCACAGGCCAAAACGTTGGTCATCTACCTGAGAAACACATTAACAACAGGGATCAGAAGAGCCTTTTGGCATGAAGGTAAGAAGGAAAGGAGGGCTGGCAGCATGCTCCTGTCTGACACCTCCAAACTCTGCACCTGGTATTTCCCTGCATCTCTAAGATGGGCCTCATGGGGTGCTACCAGGCTCCATGCTTTGCTGTGAGGATAAGAGGAGTGCCTGGAGCACAAGTCACCGCAGCTTATTCGCTGTAGATCTGTTCCTTACCCTTTCTTAAAAACAATCAAGGATGCATTTGGTCCACACTAAGGGGAGCATGTACGCAGGCAAGGTGCTTTCATGCTGGAAGGCAGCCACTCTGAGCATGGTTTCAGGAATGGGATGGTCTCAGAGCCATCACGCTGCTGATATTTCACATTTGGCACATCACCGACCCCAGCCTGCGCCTTGGCTGTGTAGGGAGACATTCTCGGAAATGACCTGCATGCGATTCAAGTTTTAAGCTGGTTAAGGAAAAGACCTCAAATGAATCCTTATCTAAGATGGAAGCACTTTTAAGGAAGAATGAAACCCAGAAAGATAAGGAAAAACATTCCCCCCCCTCCTTTACTCTATCACATCAGATTGACTTATCCTCTGTTATGTGGGTTTACTTTGAAACAGATCACTCTCATTGCAACACGTTCAGTGCTGCCAGCCACCACATGGTTATTTGGCAGCTATTTAAAGCTCAGATCATATTACTCAGTCGCAGTGCTAGGCACCCATGTTCCAACTGGTATACAACTGATCTAAAGAGAAATCAGCTTTAGGTTTTGTTCTCTGAAATTAAATGTGACGGTATGATCTCTGTGATCCTTTTTCCGCCCCTTTTCTCCCCTCCCCCCCTTTTTTTTTCTTTTTTTGTATGCATTATTACTTTTTTGGAACAGTGTTCTGCTAGCACCACAGGAGAAATTCAGTTAGGCTGTCTGCCCGATGTAGTGTATTATCAGACACCTTCGCTTTTCCTCCTCTTACATTTGCGTAAGAGCTTATAAACCACAGAGAATAAAAATTGTTAAATAGAATGAAATGACCTGTCAAGGGTAGATATAGTATTTGTGAATGACTTAAAAAAATTCCACCAGCTTCAAGGACACCAGCTTCACTGACTGACTGTGCTCTCTTGAAATCTGGCCTTTCTTTTTTCCCCAGGTAAAATGCTTTGATGGTCATCAGTCTTAGATGACCTGAAAAGGAATAGTTTTGTCTTCCCATATCCAGCCCAGGATGAGACTGTGTAGCAAGAGAGGATGCCATTGTGAGATTTGGAAGAGATTTGAATCCTGCTCTTAATATAATGAGCCTAAATCTTGAATTTTGCAAAACATTGAACAAAGTCAGATGAGTCAAGATGAGCGGCTCTTAAGAAGAAGTAACCAACAAATATTTTACACTGCAGGAAACTTTCATCACTGGAGAATAGGAAAATTTTCCATAGGAGTAAAGTGACTGATTTCAAACCCAGTTAGCTGTTGACCCTGATAGATATATATTCAGATGAGGAGCTGAAGCGATCCACTTGGATTTAACCTATCTCAGGAAGTATAGGGTAGTGCTATACCCAGAAGCACAATTTGGTCAGTAAGTTGGAGTTTTGAATGTAATCCTTATTTGATTATTTTATTTTCTGAAGTTCTGTCATCTCCTCTCCCTCCCTGTACAATTTCAGGCTTTAAAGCTTTGCCCTGAGCTTCAAATAGCAACTTCTACTGCTAATCAAAGCGACAGAAAAAGTGTATTTATGTATGTTCCTGGACTAGTGCAAAGGTTATTCTGTAACTTGTTTCTTCAGTGTGAACATCACATAATCCAAGACTGTCACATTTAGCCTTCCCTGAGGAGCAGGCTTTATGGAGGGAAGCACCGAGGTTCTCTGAGCACCACAGACAGCAGCCCTCAATGTGCCAGGACCAACACAGGCAATGAGAAATAGGAGGATATGAATGTAGCTTAAAATATGCGTGTAAAACTGAAAGAAGGCACAGCGGGCTGGCATCGTATTCTCATTCATATAGCTCCAGCTTTTTCTATAGCAAACACTTTTCAACACTGTGCATTGGAGATGCACAACGCACAGCTCATGTGAGGCTCTCCCAAGTCATAGAATCAGAATGTCCTGAGTTGGAAGGGACCCAGAAGAATCATCAAGTCCAACACTTGGCATCACATAGAACCACCCAAAAATCAGACTGTACATTTGAGAGTGTTGTTCTGACACTTCCTGATCTCTGACGAGCTTGATGCCATGACTGCTGCTCTGGGGAGCCTGTTCCAGTATCCAATCACCTCTGCTGAAGAAGCTTTTCCCGATATCCACCCTGACCCTTCCCTGTCAAGAGTCAGCTGTGTCTGTGGAAATGAACCTGGTGGGGTTCAGAACTGGATGGTATGGGTAAGCTGTGATTCTTCAGTTGGGTTCGTATGGTATTGGACAGAAGCTGTGCAGAGTTTCATGCTTCGTTGGAAGATATTTCTGATGAACTTCTAATTCTTTGTGTCTACAGAGAATGCAATCCACAAGAGGTACCTGCTTCTTATCACTGTCAGGAAGAATGCTCTAGGCTGCTCAGAAACAAACAGCTCCTACAAGCTAATGAGAACCCCAGTAGTACTTTATTGTAGAAACAACCCTGTAGAAGTCTGTGTCACAGGGCAATGACATACATGCTCAAAGACAGAAAAGAATCCCGACAAAAGAACCCCGACTGGGCCCGGGCCGGGGCAGGGCCGGGAAGGCGCGGCGGGGCCTGCAGGGCGGCCGGCGGCCACAGGGGGGCTTCGTTCAGCCTCTCACAGAGGAGCGGCGGCGGCCGCTTGGCTTTCCCCCTCCCTCACGGAGGCTGTGCTTCCAGCGCTGGGTTATCCCGTTCATTCCAGGTTCTTAAATTCTGCCCCCTTAACGCCGTTCAGGTTAAAGGCAGTTCAGCACAACTTGCCCTTTTCATAAGTGTTGTGTTACCTTAATAAAAATGTGCTAAAAGCACTTACAGCCGTCCAGAATTCTGGGGTACTGCTCTACTTTTTACTACCAACGGGTACTTCAACCATTTATTCCTTAAATAATTAGCCATGAGAGTAATCACAGAATCATAGAATTGTTTGAGCTGGAAGGGACCCTTAAAGGCCATCTAGTCCATCTCCCCTGCAATGAACAGGGACACCTACAGCTTGATCAGGGTGCTCAGAGCCCCGTCCAGCCTGACCTTGTATGTCTCCAGAGACCCCATCCACCACCTCTCTGGGCAACCTGTGCCAGTGCCTCACCACCCTTAATGTAAAAAACTTCCTTACATCTAGTCTAATTCTCCCCTCTTTTAGTTAGAAACCATTTCCCCTTGTCTTAACACAACGGATGCTACTGGAGGGTCTGCCCCCTTTTTTTTCTTACAGCCCCCCTTTAGATACTGAAAGGCTGCTAATACATATGAACAACAACAATACATCTTTTTGTTATTATTATTACTTAAACAATTGCATTCATTCATAGATCTCAAAGTGCTATTTAAATGAGGTCAGCACAATTAACCCTGCTGAACCACTGCTGTGCAGAGCTTCCAGACTGCTGTCACTGTGCATCAGCCGAGCACACAGTCCTGTCTCTAACACCTTTAAGCAAAGACTGGAGCCAAAACCAAAGCGGCAGGAAGCTTCCATATTAGATAAGGTTTTTGGACACAGAAGCTCTCACTGCCTCGAAGAGAGAGAGAAAACAGCCTGAAAGCATTTGGAAACTGCCCTGCTGTGGTTGGAACAGTTGGGGATCACTGAAGAACTGCAATGAGGGGGAGCAGCAGCTGGCAAAAGCTAGGAGAGTTAGGTGCAAGAGGAGAGCAGAGCTCCTTAAGTAACACACAGCCCAGGGAGCTGTCACGGGTCTGAGATAGGTTCCTGTTCTTACTCGGAGAGAAACGTTCCCGGTGATCTGGAAGAGCGAGGGGGCTAAAGCCCTAAAAGGAGGCCTCCAATTTTAGAAATGCCTCTTGTCCCCACAATCCCTCCATTTTTATAACATTTTTTAGGTATGACATTATACAAACTATGTAACTGACAAATCCTCCATTTGCATTAGATTTAAGTGGCTTCCCTCCCATCACTCCTCGCTGTTAAAAATACATTACAGAAATTACTATCCCGCCTAGGAAAATGTGTGATGACTCCAGTGTCTGTCGTTTATGTCTAAAACATTTTGGCACTTATCCTGCCAGGTTGTCTTTTCCTTTCAGCTTTGAGTGAGAGAAGGGGAAGTGCAGGGTGAGGGGCCAGATTTTTCTATTTCCCTAGTTGCTGCCTGCATGCATTGCAGCCCACACTGCAGGCGTGATGGGATCTAAATGCTGAGCAGGAGCAGTGAAATCCTCGTGAGGAAAAGAGAATAGAAAGCAGAGAGAAGCCACACTTCAGGCTACCCTAAGAGTGTCTGTATCAAATCAGAGCAAAGATACTGCCCAAGATCTTGTCTTCCTACAACAGTCAATATTTACAGAGAAACTATCAGCAAACAGCAAGCGTGACATGATCTTTCCTCTTGAGTCACCTTTCAGCTTCTGCAAAGGGGACAGACACCTTTCTGCCTTCATCTCATTTGCATGTGGACTGAATTTGGCCCTCTGCATCTGAACAAGCCCCCAGTCTCCTTCACTCCCCTCTCCTGCAGCCCCCAGCTGCTACATCAGTGCCATTGGGGCTGCTCACATCAGGCATTTCTAAACACTAATCAAGCACAGAGATCCCTTTTCCCTCTGCTAAGAGCCTGGCTCAATCTGGAAGGAAAGGAGGAGAATCAATCTACCTGATTGCTGTATCCCTTTCATCTTGCACTTTGCAGCCCCTTGCCAAGCAGAGCAGCGGGAGAGTCTCAGCTCGATCAGCTCAGCTGCTCCCGGCAGGTGTCACTATGGAATGGCAAAATGCAACTCGAAGGCTGTGATTGCTGCTGCAGGCAGAGTGTCCGCTGAAGTGCCACGTTGGTACCATGGCGCTGCAGCCAGGAAAGTTTCCTAATTAGATCTGAAACAGAGGTGCTGCTGCCCTCTGCTTTCCCCATGTTTGTCTCTCCACTCTCACAACCCTAACAGTGGGTTGGAGGAGTTTTCTGGGTTTTTTTGTGTTTTCTTAAATATATATATATGCAACATTGCTTAGCAATGAATGAAGAGTCACCCTTCTTCATGGATCGCTGCCTCGTGCAGTAACATGAGCACAATGACGTGTAAAGTTTCCAGAACTGTTGGTAATTACTGATAAGCCCAGTTCAGCTCTCAGTTACATACAAAGCTGGGTAAACAATGGGAGGAAAAAATTAATTAGAGCCCATGTTTGCTAAAGCTGGCTAGCGATTCCATCTGATCACGTTCCTGCTCCTTTCATTCCCTTTGCACAAATGTTTGGGTGGTCAGGTTATGCTGGTGCGTAGGTGTACAGGAGGAGCTTTTGTCTGTGCAAATAGTGCATCTCTCCCAGGGCATGGGCCCTTCTGGTAAGCACTCGCCCTTGCCCTTGTGTTTGCAGCTGGGAAGGTGTCTGAAGGCCTGGGCAGGCAGAGGTTAAATGGTTGCCTCTCCCACGCAATACATACCTGAAGTCTCCATACCACCAAAACCAGCAAGGGAAGAAGGAAGAGAGAACAGGTGGGAATAGGAAACAATAGGAAGGACACACATCTAGAGAAATTTCACACATGTTTTGACCCCATAGTACTCTTTGGCAATGTGTCTTCTGTGACACAGCACAGCCTATGTGAAGCGGAAGAGGAAGATTCAGAAAGCTTCTCTTTGGCATGGGCTGGACACATTACTAGCACAAACATTGGGCCTAGATCACATTGCTTTACTTGCTGGGAACGCTGGGACTGGTCAAGTTCTTAGAGACTTCTTCCAATCAATTGTAAGACTTCATGAGTACTACACGGCACCTTGAACACTGCATGGGCTGCTTGTATCTGCCTGATTTCTCATCAGGGACTCAGGCAACTTCCAGTGTGACAGGAGTCAGGGTGTTGCCAGGGATCTAAACATCATGTTCTCCTCCATGACTGAATCCTCTCACTGTGGCAGGCCTGATTTCCTTGTGTAAAAGTAAGTCTGGTCTTGACAACGTGGTCAGACCAGACGACCTCCAAACATCCCTTCCAACTAAAATAACTGTTAAGTCCATGATTGTGGGGTCATTTCCTGATATACTTCTTTCCCTGAGGTTCTTCCCTCAGAGCCAGTGCCTCAGTGATTTCTTCAGATTCAGCTCAAAATTGTGGAAACCAGGTCAAGCCATGATGGGAAAGCTACAAGGCCAAGCTTACCATGAGAAATCATGGGGAAAATACAAAGCCTACACCATGATTCTTACGGCATGAATGGAATGGGATGCATCCACTAGAAGAACTGGTTGCACAGCAAAATATATTTATATATATATATATAGGAGACAATGCAGTTCTTTTGGAAATAGCTTGTAGAGAGTACCAAATTGGACTGATTTTTTGATGTCAATAAACCATCTGATGAGTCCTGGCATCTTCAGAGTCTTGTGATGTCTTTCTCCTCTTGTGAAGAAATGCTTGGCAGATGAATCCATAAAATCCTGTCCTTTTGTGCACTCATTTTACAGCACTGTTTGTGCCCTGGTGCACAAAGTTGTGTTTTATGGAGTGCTAGGAAAGGCTCAGATCAAAGCTAATGCGATTTATCCATGATACTTTCTGCAAGAAGAGTTCAAAAGATAGGAAATCACATATGAAGAAAATTAGGCTTTGCATATAATTTCCTGTACTTTATGGAGAGCTGAGACCACAGAAAGAAGGATTCTGGTGAGAGGCCTGATACTGATCCCTTCTTGCTCCCACTAGCACATTGGATAATATAGATCCCATCTAATTTAACCCGTGGGGACTCCTCTTTGCCTCCAACCGCTGACCTGACAGTGTGCTTCTAACACAAACTCTGAGTGTAATTAAATTCTCTTATTTCCCCATTTGAGCTACTATTAGGAAAAAAAAAAAAAAAGCCAAAAAAACATTCCTATGCTCCTATTTTCGTTTACTTCAAACAGTTGCTGGATATAAGCCATTCTAATTAAACTGCAAAGTTCTTCTGTGGGCAGCTCAGATGTTGCTAAAATTGATGTCAAGTGTTACAGAAATCTGTATTGTTTGCAGTGATCTTTCTGAATTCCACTGGCATAGAGAAGTAGCAGCCAAAAACAAACAAACAAACAAACAAACAAACAATCAAATAAATAAATAAAGCCCCTAGATCTATTTACT

Novel00030

CATGATCTATCTGATCACTGTCTTTTTATAGGGACCACATTGTGGTGGTATTTGAGCATCCCTAACAGTAAGAAATCAGGCAAAGGTGACAGGAGATCTGCATGGATGAGCAAGGAGCTCATGGAAAAACTCAAATGGAAGAAGAATGCCTAGGGAATGTGGAAAAGGCATCTGTTCACTTGGGAGGAATACAGGGATCTTGTCAGGGCATGCAGAGATGCCACAAGGAAAGCTAAGGTCTACTTGGAATTAAATCTGGTGAGTGAGGTTAAGGACAAGAAAGAGTTCTTTAAGTATCTCAGTAGCAAAAAGGAGGACTACGGAAAATTTGGGTCTGCTGCTGAATGGAGTGGGTACCTTGGTAATGGATGACTCTGAATAGGCAGAGATACCAAACACATTCTTTGCTTTAGTCTTTATTGCTAAGGCTGACCCTTGGGAATCCCAGTCCCTGAAGATAAAAGAGAGAGTCTGGGGACTCCCCTTGGTTGAGGAAGATCTAGTTAGAGACCTTCTAGTCAAATTGGACTCACACAAATCCGAGGGCCCTCGTTGGATATATCCCCAAGTGCTGAGAGAGCTGGAAGAAGTGATTGCCCAATCTATCATCTTAGAAAGGTCTTGGAGAATGAGAGAGATGCCTAAGGATGGGAGGAATGCTGATGTCACTGGTCTTCAAAAAGGACAGCAAGGAGCAGCAGGGGAACTACAGGCCAGCCAGCTTCACCTCTATCTCTGGGAATGGGACGGAACATCTTACTCTGGATGTCATCTACAAGCAATTGGAAGAGAAGAAGGTTGTCAGGAGTAGTCAATGTGGCTCTGCCAAATGAAAAGTTATGCTCAACAAATCTGGTAGCTTACTACAATGTCATTGCAGGCTAGGTAGGTGGGGGGGAAAGAAGAGAATGTTGTGCATCTTGACTAAAGTAAGGTGTTTAATACTGTCTCCTAGAACATCCTTGTAGGTAAGTTTAGGAAGTGTTGGATAAATGAGTGGACAGTGAACTGGATTGAGAACTGGCTGACTAGCAGAGTCCAGTTGAAGGCCTGTAACTAGCGGTGTTCCCCAGATGTTGGTGCTGGGTCTGGTCTTGTTCAGCATTTTCATCAGTGACCTGAATGATGGGATAGAGTCCATCCTCGGCAAGTTTGCTGATGATATGAAGTAAGTGACACACCAGAAAGCTGTACTGCATTCAGTAGGACCTGGACAACGTGGGGAGATGAGTGGAGAGGAATTTGATGAGGTTCAACAAGAGCAAGCGTCGAGTCCTACAGCTGGGGAGGAATAACTGCATGCATTAGTACAGATTAGGGGCTGACCTGCTGGAGAGGAGCTCCGGGGAGAAGGACCTGGGTATTCTGGTGGGCAACAGGTTGGCCACGAGCAAGCCAGTGTGCCCTTGTGGCCAAGGTGGCCTGTGGTGTCCTGGGATACATTAAAAAGAGCATGGCCAGCAGTTTGAGGGAGGTGATCCTCCCCTTTGCCCTGCCCTGGTGAGGCCACTTCTGGAATACTGTGTTCAGTTCTGGATTCTTCAGGAAAGAGAGGGACCTATTGTAAAGGGTTCAGTTGAGGGTGACAGAGATGATGAGGGGCCTGGAGCACCTCCCACACAAGAAAAGGCTGAGATCTGAGACTGTTCAGTTGTGAGATGAGAAGGCTGAGAGGGGATCTCGTTAACATCTATGACTATCTGAAGTGTGGGAATCAAGTGGATGGAGTCAGGCTCTTTTCAGTATTACCCAATGATTGAACAAAGGGCAATGAGCACAAACTAGAACATGAGGAAAAACTTCCTTAATGTGAGGACTACAGAGCACTGGAACATGTTGACCAGAGAGGTGGTGGAGTCTACTTCTCTGGAGAAATTTAAGACCCTTCTGGATGCTTTCCTGTGTGACCTGCTCTAGTGAACCTGCTTTCGCAGGGGGTTGGACTTGATGATCTCCAGAGATCCCTTTCAATCCCTACAATTCTGTGATTCTATGAGGGGCATACAATATTTATGATACAACTAATGTCATGTTCTCCGATTTGTAAATTCTGTTAGGATAATGTCAATGCACAGACATTAACCCAAGAATTCTAGGTATGTTTTACTAAAAACTTCAAAACCTAGCAACGATGAACAGCCACAACACATTTTTTGCTTCTATGTTGTTGTTATTATGACTTTTTTAAGGAATCAAAGAGTCAGTATAAAAATGATATAGACCTTAAATGGTATTAGTTATAGTTTCAAAGTAAGCACTTGAATGCAATATTGGGAGTGTAGTAAGCCACACAAGAACTTAGGACAAAGGAAAAATATTTAACATGCGATGCAGCAAGAAAAGTGGCAAAGCCTTTAAAAGGAGAATAGTGATACATGAAAAAAACTTCAGTGGTTAAGTTTAGGGGAGAACGATTCTGTGAGGCTCTGTTTCCACCTTAATTCCTGACACTCATCAATTTTTTTTGTCTTTCAGACAGAATGGGCAGCAAAGAATAAAGAGAAAAGGGCAGGAATGTTCTGATTTTAACCTTTTACCCTTTTTTCCGATTGTCTTTTTGTCAGCTGAAGAAAGGTAAGGAGCAAGGGAGGCTTCTCAGTAATGCTTACGTGTTTAAAACAGAAGATTTTCAATCTGTGGATGCCTGGAAACCATGAACTACTTTAAGGTTTATAAAGTTAACCAAGGGAAGGTAGTCGCTTTTTGTATGCTGCAGGAAGTGCAGGGAATTGGAGCAAAGCACGACATGAGACGGTAACGATAAAAACTTACATTTGGGATCTCGTCACTGTGAGTTTTGGAAGGTGAGTTGCAAGCATGAGATGAATTTGATAACTTCAAGTTAATTAACAAAAATGATTTTAAAGTGTTTGGAAATCTGTTAGTGAACTCCTGTGAGGAAAACAAATAACATGAAATCCAAGGGAGGGTTGCAAAGCTGGGGAAGGCTCTGAGGGCAAGGTGTGTGAGGAGCAGCTGAGGCCTATCGGTGTGCTCAGCGCAGAGCAGAGGAGCTGAGGGGAGGCCTGATGGCGGCTGCAGCTTCTCGCAGGGAGTGGAGAAGCAGCGCTGAGCTCTGCTCTGTGTGACAGCGACAGGGCCCGAGGGAACGGCATGGAGCTGTGTCAGGGGAGGGGCAGCTGGGGGTTGGGGACAGGGGCTGCAGCAGAGGGAGGTGGGCATGGAACGGGCTGCCCAGGGCTGTGGGAACAGCCCTGAGTTAGTTTTCTTGTATACATTATGAATTGTTGGTGCGAAACTGTATTTATGCATGCTGTGGTTTATGGTATATCTGTCATTAGTTCTTTCTGCCTTCCAGCTTCCTCCTTTGGTACCCTTCTTTCCTTAAAATTCCAGCTATCAGGAAAGCATATTGTACGTATAAGTGAGATATGGAAATCACGTAGAATTTTATGGCCTATAACCAGTGAAGAGAAATCGTGGCAGCTTTCTTAACCATGAAGACCTGAGGCAGCCTATGAATTGGAGAGATTTAAAATGGAGCCTGTCCTGTCACTGCCCACGCTTTTCTTCTCTGTGAAGAAGCACAGAATTTTATTCTTTGTCTCTTCTTGAAGTGCTTGAGGGTTTTGGTCCTGCCTGTGAGACAAGACCTCATCCCTGAGGATGCTGAGATGTATTCCTTGATAATCTGAGTGTTAGTGCAATCACTGAGCATGTGTCCATAAGACTTGATTTCCAAGAGCACCTTTGGAAAGAGGATTGATTGCTCAGATGCTTGAGAAAAGCAGAAACCTAAGTGGCCTCAACTCACCTGAAGCAACCTTTCTGCTCTAATGAGTCAATGTTCAATGGGCTCTCTGACATTTACACAGTTCTAATTATTCAGATGATTTTGTACGTGCTGTTGGCATCTGTGGGAAATCCATTTTATCACCTTTAATCACCTTTCTATGCCCTAATACAATGAGCATAGCTAGTCATCGTTTTTCCTGAAAATGAATCTGCCTGATCATTGCTGATGTTCCTGTTGAGGTTTTTAATAATAAATGGTTTTAGTAGTTTTTCTTTGTAAGAGTGAAGGGCTTGTTGTACCATAGTTCTTTGCTCAGCTTCTTTATACTACGTGTAAAACCAGAGTACCCTGCAGTAAGATGATATGATTTTAGACATTAACCTCTGGAGATTTTACTCTCTTCCAAGTACCAGTTGTATGGGCCCTGGATGTAGCAAACATAACACGCTCTCCTCTATTCTGGCAAGATTTTGCTTCTGGTTCTGGCCACTGGAAGTTGAGGAAAACTTCAGTTTGCTCAAACTATGCCAGGTCATTGTGACTTTCCCTTGACAAAAGAAGCGATGTGGTGATTTTTTTGTTTTGCTATTCATTACCTCTTTCAGAAAGTAAAGCATGGGCAGTTTAGTTTCAGTAAGGGCTTTTCTTCATTCTAACATCTGTTTTTTGGCTCTAGACTTTTTGGCTAGACGGATAGAAATGGATGCAAAGCATATGCTTAAAGAAAATACTATTCTTAAAGCAGAAAGCACTGGAAATTTAGTCTTTCTAATTGGAGATGAGTTGCAATTGCTGATTTGCTATATGTAATAGTTATAGATAATTGGAGTGAATGACTAGAGATAGGAACTTACAACAGAGATAATTTACATAGCCTTATCTCCATCAAGCACCATCTATTTTACTGTAAAACAAAATAACATTTCTGGCATCTTGTGTTTTGTGGCTTTTTTTTTTTTTTTCTGGACAAATATTAAAAGTAATTTCAGAATGATACTAAAATATTTATTTAAACAAGTTATTGATTTAGACAAACACTAATATGATTTTTTTCCCCCAAGTACGTATTGGTAATACAGTTGTAACTTATAACTGTGTGTTCACTTGGCACGCACATCTTTATTAAAACAAGCTGGCTTTTCTTCCCTACTGGCTCGTCTAATCTTACCCCATGTGCCATCTAGAGGCAGTTTTGGTGAAGCAGCCTTGAATATCTTGGGTTAATATAATTTATAATTTCCATGAGTAGAAATAGTAATTTGATTCTCAACTTACTGTTAAGAACAATCTGTACGATGAACAACATAATTGGGGATCAACAAAATGTATATTTTACTTTAACTGTCCCCTGAAACTTATGCCCTCATTTAGCTGCTTATAATCACTGCTCCTCCTCATGGATTAAGGATGGTAACAAAGGTGATAACACAGAGAAGAAAATGTAATGAAAGGTGTGATGATTACTCGAGAAAAACTCTGTTCTACTCAGGTCAGATTTCCACATTCACATCTTCTTCCTCATGACAACAGAGTTGGAAGTCTTTCTCCTTGTCTGTCCACCATTTTGTTCCTTCTCCACAAAGGATTCCTTTTCTCAAGATTTTCTAACGTTAGCATAGACAAATAAATAAATAAGTAAAAACTGAAAGACCACAAAAACCCAAGAAATGAGAGAACACAATGAATCTAGTTATCCCAGTGCTCTACTTACGTTTTTAATTTGTTTAAATGTTGTTTACATTTGTTTAAATGAAAAGCAATATAACATTGTTCTTGACAGAGAAGTGTAAGTGTTTTGCTCTACGCCATCATCTGTAAAGCAAAGGGAACTGAATGGTTCCTGCTGTCTGTGTAGCTGTGACTGTGTAAAAGAGACCCAAAGCCATCTGCTTACATGGTTTCCTCTGTCAAGGAGGCACTCAGCAGGAGCCTGGGGCAGGGGAGGAAAGCATGATGCTATTTTAGAGGATGGAAGTTACAGCAAAATGAATGAACATAGGAACAATATCTGAGGTGACACACGAAAGGTCCTGGGTTAGCACCTGTGCCTTTACTAGACATCTGGAAACAAGGTGAGGAAAACAGTGGCAAATCCCATAGAGGATGCATGGTGGTAAACTTGGGCAAAGTTCATGGAAGCTGTGAGGAACCTGAGAGGCACCTCACCAAGATGGGGAAGACACCCAACAGCATGCTGTGTGCATTTGGGTATCTGCAGCTCAGCACAAAGATCCAGTGGGAAGGAATCTGAGAGGAGTGTGAAGAAAGGGTGGAGGGAGTGGAAAACTCTCACGGAGGGAGGCTGAAAAGACTCGGGCTGCTTATTTAACAAGAAGGAGCAAAAAGCCTAAAATATACCTCTGAGTGACTTAGCAGAAACTTTTGTTCACCTGTTGATTCGTATAGTTAAATAATGTGAAATTATTTTGCTGAAAAATGGAAATTGAATTGCAGTTGAAAATGATAAAGTAAAAGCTTTTCTATCCTTGACGCTGCTAAGCCCATATAACTCCTTCCCCTAACTTTAATTGAAACCAAGGACTGAAGAGACAGCGTTAGATAACACAGGGATAATAGTTAATCAAAATGAATAATAGATAATTATGAGGAAAATGAACTCTCAACCTTACAGGCACTCATCAGACCTAATCCCTGAAAGTTAGGAGAAAACTTCCTTGCCTCTTCTTGGGAAAAAGAGCTGCCTGTACTGCTTTCTGCTGTAGCTGCTCTCTTTGAAAGTTTGGTGCTTGCTATAGTTAGGGACAAGACTATGAGGATCTTTTCTGTGACCATGTACGGGCTTTCTCAAAGCCTGCCTGTCCTGCAACCTTCTCCTTCATCACTGCATCCGCATTTGGCTTCCCTTGTATTACATAAAAAGCTTCTTGTACCCTTTTTTTGAATGTGGGCCAAACTCTCACAGCTGTGAGTTCAAGAAGGCATGTTCCAAAATCCGAATGAGAGAGGAGGATTTAGAAGGGGTAATAGGAGAACCAGGAAAAAGCAGATCTAAGCACGCACATTTCTACTGATTTGTTTTTCCTTTTGTTTGGAAAAAAAAAAAAAAAAGAAAAGAAAACCGAAACGTGAAAAGATAGCAAACTCTTCATAACCATCTGAAACAGGTTTCAGTCTAATTTGGTAGAGCGGTGTACCTGAAAAAAGGTACTTTAATTGGAATTAAAGTAGGTTCTGAAAATATGTTTGTCCATGCCTAATTATAGCTGCTGCTCTGCATGTGTTTGATTTTGCAGCACGGATGGAGCAGCTATTTCACTTGGCTGTGTGTGCACTGGCCTGCAACCTGCCCCCTCATTCACTGCATTCTGACACTATGTTTAGAAGCACCAACTATCCAAATGGTAATGGGCCTCATGCGTTGCATTTTGACTAGAAAAGTTAAAGCCAATTAAAACTAATGCCACAGCATTGATTTTTTTTTTTAATTTCATTTTAAGATGTTCTAAAGTCTATTTTGGCGGAGTACGCTGAAGATACATTTACTAAACAAACCTATATTTTATCCATTACATTTCTCTTGCATTTGATGCCATCTCTCCTCAAGTATTAGCTTAGAAGAAAGACAGAATTTTTCTCAAAAGTGGGTTTGTAGAGAAGGGAATTAAATGTCTTCAGCTGTGTGGCCTTTTCATTCTCCCCATAATGCTGGCTGGAGCAGCACCTGTCTCTGGTCATGGCAGGGGAAAGTAAAGTCGCGTATGCAGCTCCCTTCCCATATGCACTTGCGGCACTGCCAACCTCTGGTTATTCTTTGTGGTTGTGACATTGTCTTGAATTCTTTCATTTACGAATCTTCTTCTTCTTCTTTTTTTTTTTTTCCTGAATGCAGTCACTTACAGGCAAAAGTTGACATTGCAAAGAAGGTGAAATTTTGTACTTTTTCTTGTGAAAGGACATTACTGAAAGGATATTCTGATATTCAGTAAGGAATTTTAAAAGCCTTTTGCTAGAATGCGGTGAAGGTAGAATTTGTGCTGTATCTCTATGGGCAGGAAGATAAAAGTTAAAACAGTCTAAGATGTTTCAGGAGAACATAATTCAAGAAAGGGAGATGTACACAGCTGATTGCATAAAGTGCAGAGGCTGCAACTGTCAACACAGAAATAACTGAAAGTCAATTTGAGTTAAGAGACATGAGGGATAGACAGCTATTAATAATAATGAGACACCGAAAAAGATCCCACAGCAGCCAATGGAAGAAATGTGAGAACGTAATGCTATTAGAAGGATTTGTAAAAATAGTAAGATATGTTTGCAGGAGTGATTTTTCAGTCATGTTGGTGGAGTAATGGAACATAAAATGTAAGTTTCTGGAGTTGGAGCATCAGAGATCCATAAGCAGTCAGAATTGTGGAACTAACCAAAATCCATACCAAAGGAAAAGTCTTACAGAAATAGCTGAACTGCTGTATCAGATAACTGTAGCAAAATTACATCTGATCCAGCATGGAACACTTGTACATATCACTCAAGAGTGGAACGTGAGAGGAAGAAAGCAGAGTGGATACAGCTCAAAGAAAGTAAAAAAACAGTGCTAGCCAATAAATAAATAAGTAAAAATATCCTTTGAAATCCATCTGCATTTCTGGTGCAATTATAATTTGCAGTGAGTAAGAACCATTTTTTTCCAGTCATTGTGTTTGGACAAGAAATAGAAAAAAAAATAAAGCAGCGCAGGCTTGGTGGAAAAGGTGAGAGTCTTAAGTGAGGCTGGCTTAATTGAATGTTAAGAGCCACAAAAGTGGTCCAAGAGAAGGAACACCTCCCCTACAAGGACAGGCTGAAAGAGCTGGAGCTGTTCAGCTTGGACAAGAGAAGGATCTGGGGAGACCTTAGTGTGGCCTATCAGGAGGGATATAAGAAAGATAGGGACAGATCCTTTAGAAGGGTCTGTTGTGATAAAACAAGAGGAAATGGTTTCAAACTAGAATAGGGGAGATTTAGACTGGGTATAAGGATGCTCTTGGCTGCTGGTCATCGCATAAGACCATCCCTCACCTGGAAGGAGGTGTGACTCTCCTAGCTCATCTCTTGGACAGTAATGCTGCATGCAGACACGATGTTGATCACCTTGGACTTAGGCTGGTTTGCTTATATGGTAACTCATGTGCCCTAGTTATTACTGTTCTTTACATTAATAACTTTACAGCCCACCATGTTAAATGGCATTCAACCATAGGTCATTTACAAAATGAGATAGTTTTAAGATGATCCCGCAATGTGCTGAGTAGCTCCAATAGCGCCCATAGACTCTAATTTGGGAAAGGTATTTGTACACACCCACGACCTTAAGTGCCTCTGTTCCTTTGCCTAAAGACAACATTTCCTAAACTGATGGCACAATGCGTTCAACCCTTATTTAAAAAAAATCCCCAAACAATGTGAATGTTGATAAAAATGTTACTGTCTTGCTTTTCTTCCAGTGCATTTCAGAAAAACATGAACAGCATAATTGCTGCATGTTACGCTAGTAAAATATGCATGTTGTTTGGAAGTTGCATCCTTGTGATTATTTTGTATAGCAGTGAAGGTTGTATTCTTGTGATTATTTTGTATAGCATCAACGTTTTACTCAGTTTTGTACAAGGTATTAATTTTCTCTGAAGACTTAAATTACTTTTTTTGCTACTATTGTTCCTTTTACTGAAGTTATCTAATGTGATGTAAAGTGTGGCATACAGGGAGTAATAATCTACTGACATA
