# Supplementary material for: Transcriptome Analysis of Differentially Expressed Genes Related to the Growth and Development of the Jinghai Yellow Chicken
Source: Genes (Basel). 2019 Jul 17;10(7):539. doi: 10.3390/genes10070539 (PMC6678745; doi:10.3390/genes10070539)
Supplement: Supplementary file 1 [file genes-10-00539-s001.zip › genes-527003-supplementary/Supplementary files 1-12/File S6 M8FvsM8S differentially expressed new transcriptsí» sequences.doc]

Novel01067

GCCGTGCTGCCGCTAGGGGGCAGTGCTGTCCCCATTGGCTGCCGCTTCCCCTATGGGTCGACATTGGTGCCCCTATGGGGCGGCGTCGCCCCCTTCTATGGGGCAGCGCCGCGGCAGTGGGGTGACTCCTCCGTTGCTATGGGGCAGAACCGTGGCTACGGGGTGGAACTGTCACTGCTATGGGGCAGAGCCGTGGCTATGGGGTGACGCTGTCCCTGCAATGACGCCGTGCTGCCGCTAGGGGGCAGTGCTTCCCCCGTCCCTGCAATGACGCCGTGCCGCCGCTAGGGGGCAGTGCTGTCCCCATTGGCTGCCGCTTCCCCTATTGGGCTGTTGGTGCCCCTATGGGGCGGCGTCGCCCCCGCTATGGGGCAGAGCCGTGGCTATGGGGTGGCGCTGTCACTGCTATGGGGCAGAGCCGTGGCTATGGG

Novel00995

AAAACATTTGGCAGGGAGGTGATGAGATTTCTGAAGTGTGAGCTGAAAGCGGTTCGGGGGATGCCCCCCCTTCGGGGGGCATACCTGAATGGGGACGGGATCAGCTCAGCCCTCTGCCACAAGGAGAAGTTCTGGGTGTGCAGCAGAGCCGACAGCTACGTCTGCTGGAGGAAGGAGAAATACCCAGAATGACAAATCCCATCCGCACCAAGCTCGTGCCAACACTACTGGTCTCTGTGATGCCACTGGTGTATACTGGAATCTGCCTCTTTCCAGCACTGCTGCAGCGGTTCCTGCATGGATCCACACAACTCTGCTGGTCGTTATCTCTCAGGCGCTATGTCTGTTCCTGTGTGCCTTAAAGGGAGGGTCTTCTGAGGGTCCTTAGAGGTCGCAGCAGCTTGGAAGGATCCCACGGTGCTGCCCCACTGCGGCCGGACCCGAGGGCGGGGTATTCCTGCTCTCCTTTCGGGCTCTGTGTGACACTGCGGTGCCACCACCATGTCACATCGCGCTTCCATCGCTGCCAGCCACTGTCTGACTTTGGCTCTGGGCAGTGATGTATGGAATCAAACTCTTTAACCTATCTAAGTTAAATAGGTTATAAGGCTGTACCTCATTGTGAACTTCCGAGCTTTTAAGGGGGGTGTAGCCCAAACCAGCTTGTATTGCTGTGTTGAGCTGAGGCATATCTTTACTGTCCTGTCTCAACAACGGGGAATCTCTTCTCTGTTCTTGTTGTTTGGCATCGAGCTTCTGCGGACTTGAGCTGATGTGTACACAGGGACCTTGTCCTTCTGCTTGTATCCACTCAAGTTATGTGAAAAAGGCCGTTCTCAAAATATGGAAAAGCTGTTGTTATGATTCAGTCCACCCTTTTCTATATCCCAGGCAAATCCTTCTGTCTGCAACGCACTGTTGTCCATTAACACCGTCCTGAATTACTCTCCTGGTTCCCTCCGGTGCCTCATCCGATGTCTCTTCCAGGTGTTGCAGTCATCTGGATTTTTTCAACAGCACCAGAACGCACACCTATGCAGGCTGCTGCTGTGCAGATAATCAATACAATTACTACAATTACAAATAGTTGCTTTAGCCAGGCCCAGCTGGGGAGCTGTCGCGAACCCCAATTCCAGGTTGTCGCGGATTCCCCAGGGCTCGGAGAACCAGAATCTAGTCAGTTCGCTGGCCAAGCCGCGTGGGGAGTGCTGACATGACCCAATACGAGTGCATCAAAATCTCCGTTTGTTATCCGTTGCTTACCCATGTATATAGATTGTGGCAAACGTGCGTCAGCTCGTAATTGGTTGAAACATTGCGAGAGCCCCCGGCCCTACAATCCCATTGGCCGTAGAGTCACACATCTTGCTCGCCTAGCTACAGATACAGCATTAATCGTCCCATTGTTCTTTGTATTTCATTACTCAGTCTCTTCCCAGGCTAACTTCCCCCTTATCTTAGCTGCAACCTCAGCTCCTTCTCTGTGCATTCATAACCTCAAGGTCTCCTAGCCTGAGCTGTGCACGGAGCGCCCGAGCTCAGCAAGTCCTTTCTTCCTGAGCCATCATAGCATAGCAAGCTAGTACTTTTCCAATTGTATCTCCTGCCAACTATAAGGCCCGTCCTGCTCGTACAACCGTTCAAGAGACCCGTGGCCCCCTTGGTTATCGAGCCATTCCTCAACAGGGAGCCGAGATGTTGGCTTCTTGCAGAGTTCCGAAATTGCACTCGTTACCTCACGGTGTGTCATCCTGGGTTGCCTGGTGTAAGACTTTCACACGATCCCAGGTTTTCTGAACGCCGACCTCTATTTTACCATACCCATCTATGTGAGTGCAACAACTAGTGTTAATCAGGACACGTACCCCCCCGTATGAGCCATTATTATATCCAAAGCCATTCTATTTTGAAAGACCGTCCCTTTCAATGAGTCTACCTCCCAGTTCGAGTGATAAGGAACTTCAGTGCTTGGATTTGCTGTTACCTCAATTTCTGCTGAAATATTCACTATAGCCCATTCCAGCTGTGCTATACGTAAGGAGGGGATTGAAGGGCAAAATCAGGTCCCCGCAGTATAGAAAGAAGGGCTTCATAGCAAGAAAAGAGAAAGCAGCTTCACAAAGCAGACATAAGGGGCAAATAGAGATAGTTTATGTAGAGAGCATTAGAAAACAAGGGGTTCCAAGCACTTTCACAGGCGCTAGGTCCGGAATAACAAGGAGAGTGAAGGCCATAAGGATAAGGACCGGAGAACAACTCGAAAACATTTGGCAGGGAGGTGATTGGATGACTACAGGGCAAGCTGAAGCCGGTTCGGGGGATGCCCTCCATCTTCAGGGTCAGAAGTTTGCAGCGAGGAAGACTACGAGCCTTCATCATCACGACCCGCTACGCACACGCAAGGTGGGGAGGAACTGCATGCTGATGGGCTCTCGGGAATGGAGTGAATATGTCTGATATAATATATATATCATATATATATATATACTGATATATTACTATATATCATAATACTGACGATGTATTAGTTCGACCTATATCTATAGCCCCATTTCTGCTGACGCTGTGCAGGTGAGGTGGAGCGATCCCCCTTGCACCGGGCTGTACGCGCGTCACCAACAAACACATCCCTGCTCTATATCCTTACCGGCTATGGGGTCTGATTGCCGCACGTCAGTTTGGCACCCCAGGTGGGACCCACTCTGTTCGGCTGCAGGAGCGGCTGAGAGAGGGGACACCTTTGGCGCACCCCGAGATTTCTGGGAAGGACTCCCTTCCTCACCCGATCACTGCGGGGACAGACAAGGACCGTCTGTAGGCGGACCAGGTCTGTGTATGGGAAAGGGGGAACCCGTAAGGCGTGAGGAGACGTCCTACGCAGGGTATGGAGGAGCGTGCCTGCTCCCCCCCCGAGCTACGTGAGCTCACGGGTTCGGTTGCCGGCTGGGGTAGGGAGACCCCTTGGGGAGTTCCTACAAAGGGGAGCTGTAAGTCGTATGCAAGGAAAGAGCAACAGCTCTCCCTTCGGTGATTGGTAACAGCGCGGATTTTGTCAGATGATAGCGTGGATTCTCCTGGAGAGAGCAGTGGGAGTTATAGGTTGTGTTATTGTTATCACCATCTGCCTGAGTGTTCGGCACCGTGGATCTCATCATTGCAGGTCCAGCTGACTGTCGGAAAAATCTTAAAGCTTGCAGCTGATCGACTGATGTTATTCGTGTTATTAAGATTCCAGCTAAAGGACATCGTCAGCACTGTGCATGATGTATAAACCATGGCTTTTGTCCTAAATGTCAGAGTGAGTGTGAGTGCGATGTGAGTGAGACGCGTTCCGTTCAGGAAATGACTCCATTTATGTAGTACTGCGTTCCCATTGTGGGGAAGAGTGTAAAGGAAGGTGGACGGGCTTCTGTGAGATCTGTTTCCGTGTTTATAACTGAACAATGTCACTATACGGTGTTAAGG

Novel00289

TTGCAGGGCAGTTCTGAAGATGGTACCATCTGCATTCAAAGTGGAAACTGGAGAGAGAGTTTAGCAAAGAACCATAGTGGGACCATTGCTTGAAGCTGTAAGAAGGCATTCTTAAAGAAAACAATGAACTGATCTACCAAAAATTGGAAAACTGTAGGGTATTTTGGGTATGATATAGAATATTGGAAAGAAAACATCTGCTATTAATGAGCTATTTTAGTTTGAGAAAGGTCCAAGAGCAACCATGAGAGCTGGAGCTGACAGTGTTCAAACACAGCATTGTAATGGTGAGTATTCTTACCAATTGGAAGAAGCTGTCATACAAGGACACACCAAGAGAACTGAGACTGTTCAGCTTGGAGAAGAGAAGGTCCAGATCCAGAGTGGATTTGATCAGTATGTATAAATGTCCAATAGGAGGGTGTAGAAAAGACATTGTCAGAATCTTCTTGGAGGTACTGTGTGACAAGACAGGAGGAAATGCTCATAAATTGAAATATGGGAAAATCTATTTAAACAGAAACAAAAATGTCTTGATAGTGAGGGCAGTCAAGCTGGAACAGAGAATGTCTCCATCTTTGGAGACATTCAGAAGCTGATTGACAAGGTCCTGGTTGGCATAGATGGTCCTACTTGAACAGGAGGCAATCTCCAGAGGTTCTGTCCAGTGTCAACCACTCTGTGATTCTGCAACATTTTTCATGCCAAGAACATTCAGTGTTGTGTTTGTAGATATGTGGGTCTTCCAGTGTCTTTGCTTTGGATCTGCTTGGCATTGCAGGCAGAGGTCTCAGCAGAGCTGGAAGCAAGTTGAAAAACTGGCTTGGTGAAAAAGGATGAGCGAAAAGAGTGAGTTTATTGCACAAAGGCGTAAAGTTTATTGCGAGTTAAAGCAATCTTGAAGTGGTGAGACACTGACATCTTTGGGCAACAGGTTGAGCTGCCCAGTTCCATCAGTGCCAAGGCTGTTAGTTGCAGGAGCCCATTATGTTGATGAGGATCAAAGTCTTGGTTCTGCCTGATGGACAATCTGACAAATACTGTTGGTAGTGACCAGCAGCAATGACCAGGATTGTATGCCTATCTCCTTTGCTACAGTCCTGAAATTTTGGTGGAAGGTGTTTCTGCTGCCAGAAAGTTGTGCAGTATACAACTTAGTGATGAAATCAGTGCTGTGACTGCACCAAGCTCTTGATGGTGGTGCAGATAGAGTCACAATGAAGCAAGAGCAGCAGCCAGCACTGTGGCCAAAGACATGCCTGGATCCAAGTGCCCAGCCTGGGTGCTTTTATCAGTGGCTCACCCATAATTGCAGCTGGATTTCTTAAATCAATGAATAGGCAGACCTGTTGAAGCCTATCCACACGGGGGGTCTGAAAAAAGATCTGCTGTCCCCATCACCCCCGCAGCAGCTGCTGGGAGAGGCCAAATAGAATGGAGCTGCGTGCAAACACGCTGGCCCTTTGCACGCTCTTGCCGCTTGCTGCAAGCAGGCTGGCTGAGTGCCTGGTGCAGTAAAAGGGCAAGACTTCAGTTAATGTAGGCACAAGGTATGGGGCAGATCTGGTGTCGTCCCCCAAGAGGCATCAGGTGACTGCAAAGGGGAAGGAAAGACGTGGTACTTCACTCCTCTCTGAGATGGAATGAAGAGGTAAATTGGTCAGTTACAGAGCTCACTGCACTGTGTGAATTCTGAATGCAACAACTGCAGAACAAGAGGTGTGAACACAGCCCCTGATGTCCTAGTGCATGCATCCTATGGCTCTCTAGAGAACATGTGGTCTGTACAGTTGGATGTTGTGGTCAGTGCCATCTCTCCCTTCTACAGGAAAACTGAGGAAAGTCATGCTATTAGGCTGACTGACCCCGTGACAATAGGCAGAACACAGGTCCAGCTTTCAGGATCTCTCCAGTTAAGCTTCTGTGCTCATTCCTAGAAGCTTTCAGCATATAATCTTCCTTCTACCATCTGCCACGTCAAGTCTGACACTCTGTTACTTCCATCTCTTCCCACTTGAATCATTGCGAAGACTGTGCCTTAAGTTTCTCAGGAAAGTTAGAGAAAGACAAATCCTCTGTATTCCTCAGACTTCTCTTGTAGTATTGTCTTCTGAACAAGGCTCCTACCTGACCCTCCTGTGATAACAGGTGTTGAGAGTGTCAAAGAAATAAGCAACAGAGCCCAAAAATTATTGCAGGAAAGCATAGCAAGCCAGGAAATATAACAGGGCAAATACGAGACTACATATTGCCCATTGCTGCCTCCTCCTTCATTGCTTTTTCTTGACTTTTGACATACACCTCAAAGGCAAAACAGCCCAGAGCTTCGATAAACACAATCAATCAAACAGAAAAAGTTAAGACTCTGTTCTGAGCAAGTGTCCTTCTGAGCACAGGAGTGGAAAGAGACAATAACTGGAGGATAGAGATGGTGAGGATTTGTTACCTTTCATCCTTACTGACACAGGGGCAGGCTGGCCACAACTGCAGCCCAGTTGCTTTCTTCTAGAGTTTCTAGAGCTGTTCCCTGACTGGAAAATATGAGATGGCTCATGGAGTGTCAGCCATAAATCTTTTATTAATTTTTTTTATTACTGAATCTTATATCCTTGAGCGGAGATGTGTGAACAAGAGAAAAATGGCATAGGCAGAGAGATCAAAGGAACAGGTTCCATATTTCCCCCATCCCATACCTTGGGTTGCACTGTACCTTGCTGCCCATAGTATAACATTAGGTCTCCTTGTGCTCTGATGGGCTGTCATTGTCAGTGTTGACCACAAGATGAGAACTGACTACTACTAAATCCCATAACCGTTCTCAGAGTCCTAGATTTCCCTGCTCACAGCCCAGCCAAGCTCTGCCAACCAATAGTCTGGGCCTGCCCATATACAGGCCTGCCCATATACAGGCCTGTCCGTTGCTGTGGCTGTGTTTCTGTTTTTCCCTGGGGCTGACAGGCAGTCGGTCATGTGTGTAAGCCCTATCCCTTGGCTCAGCTTTGTGCAAGAGAGGGAGGGAGGGGGATTTGGCTGAGCCTAGGGTGGTGACAGAAGGTGGTCACAGTTGTCACACAGAGTAATTGCACTTCTAACCCCTCTTTGCTGTTTACAAGCTGCCCCCCACCAAGGAGCTGTGTCTCTGGCTGACCCCTGTACTCCAGGATGGAATAGCACAATAATCCGGTTCTCACGTGGGGCCCTGGTAGTACCTTTTGGGTGTTAACTCTGACCAAGGTCAACCTCTGCTAGAGCTTTCCTGACAAAATTTACTGAAGGGGATTAATACAGTCTGTTTTTCAGTTTAACCCTGATGTACAGCTACTGGTTACTGGTCACTGGTTACAGGCTGCAAATAGTCCCTTGAACTGGCAGCTCTCAGCCCCCTATCCTTGTTCCCAGCCATGCTCTTTCCCTGCCTCAGCTGATTGTCTTCTCTTCCACTGATGGAGTGGGAGATGGAGGCACAGCAAGGACAGCCATGTGCTGACATTGCTTGGCTCTGCTGAATCTGCTTTCTCCAAAGCCAAGTAGAAAACAGTGACAGCTTTCACATCCCCTTGTCCTTCTGCTGACAGTTGTTGCTATTGACACTCTCTCTTGGGAAGCTGTTTCATCTTATTAAGGGCAGTGAATTTTGAAGAGATTGCTGAACACAGGGCTAACATAACCAGCATTCCCAGGAGCATGCTGGGCAGGCTTAAATCCTGGCAATTTCTTCTGCAGAGCTTCTTTGCTTGCACCCCTCTCAGAATCTACTGAAAAATTCATGGCTTCTCAGCCTTATTGGTAGAAATATATTTATCAGCCTGCACCGTTATGATGTGCTGCTACAGGTCAATGCTCCGAGGCTCATGGTTGTGGACAAGAGATGCAGGGACAGGACTGAAGAGGCAGACAGGTGAAGCAGAGACCAAGAAAGGGAAAGACAAGAAACAACAGCGCACAAGGAAGATCTCTGAAAGCTCCTCAGGACATGAAGGAGAGAAGAAGTCGTTGATAAGTGTCTTCAGTGTAAAGAATGTTAGTCCACAGCAGCATGAGCACTGCAATGCCATCCAGGCCCCACATGCATGGAGCTATTCTCACCACGCAGATGGTTCCTTGAGCATAGCACAGCGGCAGCTTTCATGCCTCCATGATCCGTGTGGATCATTGCACAGAAATACAGTGCTACTGTGAGCCTTGGGCTAGGACAGTCTTGTGTTGTCCTACGGTTGGATCTATTGCATAAATCAGCTGTACAGTGCAAAATTGCTGTGTTCAGTCCATATTGACTAGTCCAAAGGCATGGAAGTTCACCATCTGCTCATGTTGCTCAGTCTCTGGACTTTGGAAACTTATGTCCTCAGGCTTGTCCTCCTTCGTACTCACACTGATCATAGCACATCAGAGAAGACTGCAGCACCTTTATCCCACTAGGACTGCTCATCACTCAGCTGGAAACATATGAGCACATTTGCCTGAGGAGCAGCTGCTGTTTGGGTAGGATGGGCACCTTCTATTCCCAGTGCTGGAAGAGTTTAAGGCAAGGGGATGCTGGGGCTCCTGGCAACTTTCTGCATGGCTTTGAAGGTAGCCATTGCCAATCTCTGTTTGAGTTTACTGTTGCTTGGCTACCTCCAGGAACCGCTGAACTGGTTGGCATCCTTTGGACTGCTTCATAATGGAAATCTGGGATTAAGGCGAGCTGTGATAGGCAGCAATTCAGACCAGCCCCTGAGCAGCCTGCAGTCCTCCCACTAGTCTTTGGCTGCACAATGGCATGTCAGGAATTGGATAGGGCTGGCAGAGACTAGGCTGTAACAGGATGCCCCTGTCCTTTTCTTTCCTTCCATCACTGGGTTTATGGCACGTGGTATGTGGGGGCTGCCAGGTGGTCATAGAAATGTTGAGACATCAAAAGGGAAAGTATTGTTGCTGCAGCGGGGTAAATGGATGTCACTGTCACATTCTACAGCCTCTGCATAACTGGCCACCCACCACCAAAACTGGCTTGGTTCAGTGGGTGAAATCTCACCGATGAGAAGTTTGGACACTGAACTGTAAATTAGGTCACCCAGAAGATGGGACCAGGGAACAATATTCAGTAATTCTGACTTCAGAGCTCCTTAACTGGCAGATATGTGACTACATTCCCAGAGTTGGGCAAAGAACCAGAAGTCCTAACCCTGAGTCTCAATTTTCCTTTCCACCACTTCCTTTCTATTGCTGTTAACCCCCACCCACTGGACTTTGTCTTGGGATTGTATTCTTTGAAGGGAGATTTTGACACATTAACAGCATAAGATTGAGCTGAGCCAGGGAAGTACAAATGCCTAGGACTGGGGCTAAACAGATCAGTGACAGGAATGTGTTTTCCAAAAGGGAGGGCACCACTGAAAGGATCCTAGCCCCATCCTCTTGATATCCTCTCTTAGGGTTTTGAAAAGCATTGGTAAGATCCCCTCTCAGTCCTCTCCACACTGAACAGTCTCACCTCTCTCTGCCTTTCCTCATAAAGAAGTTCCAGGTCCCTCGTTGTCCTTGTGGCCCTCTGCTGGCCTCAGTCTTGAAGTTCCCTGCCTTGAAGTGGGGAGCCCAGAACTGGGCATGGTACTCCAGATGTGGCCTCAGAAAGACAGAGTTGAGGGAGTGGATTACCTCCCTTGACCTGCTGGCCACACTCTTTTAATGCACCCCAGGATACCACTGGCCTTCTTGGCCATAAGGGCACACTGATAGCTCACGAGTTGACATCATGTCTCACTGTCTCATTTCAGTCCATTGCCTCATCTGTGCAGAACAGCCTAGGAGTTCCTTGTCCGTCAAGGGAATTAATGTCTTGTGTGTGCTAAATGTGGATAAAGGCAGAGACCTGCCACCCAGCTTCATAGTAGATTTAACCTTTGGATGAACTTGAGTTTGACACTTGTAACTTAAATTGCACAGCTCTCATCACACGCCAAATAACTAGAAATAATGACATGGAAGTCTGACGTGGTGTCATTCAGAAACAATACCATTTTGCTGTAGCTGAAGAATGAGGGGCTGATTGGCACAGATGTGTATCTATCTACACAATCCCACAAAGAATTCCTACAGAAGCAAAACGTTTGAAGCACTTCTATTGCTGGAGTTATGTTATGGCAAAAGGCTCTGAAAGCAGTACCTCTCTACGTAGTGAAACAAATTTCCTCTGACCAGCTCTGGTAGTTCCTTTCCAGGTCACTTAATGACTTATTTAATTTGCCTTGTTTAAAACAGAGTTGTCTATAGTCGCACTGTTTGGAGCTTATATCAGCTGTGTTAAGCACATGCCTCACCACAAACCATATAGGCTGTGAATTCCTAATGCTCAGGAAAGCAGGAGCAATAACTTCTGCACAGCCTCATGTACTGTTTCCTGTGTTCCAAGTATTTGGCATGAGTACTCATGCTAAGCTCTGATACAGGTTTTGTTTCTCTTTAGTGGCTCTTTGGACTCTTTTCAGCTTGCTGCCTGGAGGATGGCTGTAGCAGGTGCATTTGCAGGAGCATTTGCAGGTTAGAATTGTGTGAGGGAAGGCAGCGGGACAGTATGAATGCTCAGGATGGACTGGGTTGGAATTCACAACCATGCCTCTAAACTTTCTGCTTGTTCTTAAGGTCTGGGAACAATTGTATGGGGAAATTGATGTTCTCACATAGATAATCAGAGATAGTAAAGAAGAAAGAGTAATGGGAACTGGAGGTCAGGGACCTTTGCTTACCAAGCAGAAAAGCAGACATCATTTTCAAGGCCGGCTCACTTTAGGAAGGATAGTGTTCAGCTCCCTTTTCCAGCTCTTCCCTCTATCCCAGGGTCTCTCAGGATGCTGGCAGAAATGATCACGATGATTCACACAGCCATTGCCTTGCAGATCTCCCTCATTAGCCTTAGTTAGCTTCACTGCCTTTATTTGTCTTTGTGATTTGGACAAATCACACGTATCACATGTGCAACCTACATGCATACTGCTGCCTCTCACTCACTTTCTGCCTTCCACGGAAATTCCGACACACAGACCATGAACTATCAGGACCAGGGCTCCGATCTGGGCGACTGAGACACCAGAGCTGCTAAATTTCTTTCTGTGTGGGCTTGAAAAAGCATCTGTTATCATCTCATCAGTGCCGCTACTTAAACCTGTGTAGGAGCTAGCCCATCTTGAAACCTTGCAGGAGCTGGTCTGTCCCCATCCTGCCTCATCAACAGAGCAATTCTTCCTCCCCACTTAGCTTCTAAGTGGTATCCTGTTTTTTGCAGGTACCTCCAGGGGGTGTTTCACTGCAGGAATTCTCAGGACCACTTCTCAGTCCCTTGGATGCTCACACTGCTGTGTTTCAGCAGCTAAGCCTCCAGTGGTGGGAGTGAGCCAAGGACCTGGACACCCATACCCACTAGCTCCTGTCTCAGCAATCCAGATTGGCTCAGGATTTAGTAATACGACCTGCTTTCCACTGCTGTGAGCTGCTCCCTCTAGAACCGAGTTCCACCTCCTCCCATGCTCAGTTTCAGATGGAAATCGAAGCTAACACTTCAAATCAAGCTAGCACTGCTAAGTATCCACTGAACTGTGTTAGCCCTGTTAGCTTCTGCACTTCTATTCAACAATAATGCTCAGTGCTCAGTTTTGAGAACTGTATGCCTCTGTTTAGGTCCTCTGCTCTTCCTAAGCAACCAGGAGCTAGGTAGTGACATGCTTTTTGCCATACCAGAAAACTTGCTGCAACTTTCCAGTGTCAAGGAAGATGGGACTAGGCAGCTCTGATCTGTCCCTCTCCCATACACTTGCATGGTAACAAAATTAAGGGAACAAGAGAAAGATCCTGCCAGAGATCCCGAGGAGTCAGAAATGGAGCTCCCATGAGATTTGTAGCAGTAGCTTTTCTTAGAGACTGAGTCACCATAGCTGAAAAGCAAGGCAGCACAACCAGCATTAAGATGGTGTTCCAGTGCAAGCCTGTGCCAAATCTACACAGCAGAGGATCTTTCCTAAGCAGGTTTTCAGACATGTCTGCTTTGCCAGCCCTCTCCACGCATCACCTTCCATCTCTCTGGGCTACCTTGCCAGTCACTACCTCTTGTAATCACTTAACTTTTGGCACTGCCCTTAATTCAAAAGCAGCACTGGAGACTTGCCTTTCCTGGCCTTTGGCCTTTTTCCACTTACTAGAGACGTGTTAATGAGAAGGCCTCGTATCTAATCACCTAGTCAAGGCACTAGGAAAATGCCAGCCAGCACAGTAGCTAGATGTGAAAAATAAGAAACCAGCTAGTGTCAGCTTAGACTGCTGCTTGAAGGGTCCTTAGAGGAGGGTTAGGTTCACTGGAAAACAAGGCTGAGGGTGAGGGCCATCAGCTCCTGCCCCTAGTTGGAGAGACAGACCTGGCAGTGAGCAGCAGGGAAGGCGGTGATGTATGCGTGGGAGAAAGGTCAGGAGATTGCAGAGGGCTTTGCACCTGCGGCCTGGGTGACGGGAATGGCCAGTGAGTGATGAATAGGAGCAGCTGGCAGAGAGGCTCACCACCAGACAGTCCAGTTAGAGAGTGGGAACTCAGCGATTTTCATGTTGGATGGGTTTTTTACGATACACTGAGAACTAGGTGACATAAGGCACATAAATCAAGGCAGCTTCTTGAAACTGAGACAATCTGTGGCCCTGATGCCCTCGGGCTGGAGCAGCTGAGGTGACACTGGATAGTCCCTGTGTACATGGGCCCTGTTTGTGTCAGCAGGGAGACCACCCTCCTGCTCTGGAGGAAGGAACACGTTGTTTTTTGAGGATCCAAATTGGTTACGTATTAATTTTACAGAAACCAACAGTTTTCCTCACATGGTGCAAAAAGGCACTATGCCAGATCAGAGGTCGTGAAATCTAAATTCCAACCAAACATTTAAATATTTCATGTGCGTTTATTTTTTGGTTGACGATACTATTAAACCACATGAATGAATTTTATTATATTTGCTCATTCACCTCCATCTGCTTTAGTTTTCATGGAATCATCAAGGTTGGAAAAGACCTCTAAGATCAACCAGCCCAACCATCAGCCCATCCCCACCGTGCCCACTAACCATGTCCCTCAGTGCCACATCCACACTGTTCCTGAGCACCGCCAGGGATGGTGACTCCACCACCCCCTTGAGTAGCCTGTTTTTGCTATTTCTTGTCTCCTCTGTTTCCCTCCTCGGGCCTCTCAACTGCCCACCTCAGAAAAAGAGTGTACAAATTTCTGCCTGCCCCACTGTCTTTCCTCGAGTCATTCGAGGCAGGCCCTTGTTCCCTTTCAACACCTGTCATTCCAAACCTCGCGTATGGCATTAAAATTTCATTCTCTCCCAAGAGACACCAGGCAGAGCAAGTGCTGCCTGTGGATGTGACATGGAGCCACTGTCAGGAAGCAGCAGGAGCAGGCTGCTCCACAGGAAAAGGCGGGCCAAGGACTGAGGGTGACAGCAGGGCAGGCAGGGACAGCTCATGGGCAAGGGGGACAAGCAGGAAAAGCAGGGCATCCAGTGATGATGGCCAGGGTCAGCCAAGAGCACAGATCACCAGGCAAGTCCATCACAAGGCAAGAGCGAGTCACTAAGACAAAACAGCACTGGGGTCAAGGCACAGACAGATAAAAGGTAGCTAAGGTGAGCAGCAAGGGCCTGGGCCTGGACTTAAATGCAGCTCCTGAGGGAAAGGGAGAGGCATCTCCCACCCCACGCTTTTGATAGCAGTCTAATTGAAGATGACACAGCTGTGCCTCCAGCACAAGTATCCAAGCCCCTGGGAGGGGAGGCAGGGAGAGTGCTGTCTGGGCCCTGGCAGTAGTGTCCCATATTCATATTCAGGGAGCAGGCAGACGCCAGGCGGCTGGCAGGGACCCGTTCCTTTCAGTGCCTGGTTCTGCTGCTGTTCCGTACTGAACACCAGGAGCCTCACTGTAGCACAGAGACTGGTCCTAAATAGCCTAGGGAGAAATATCATAAAGATGCAGATTCTAGCACATGTACAGCAGGTTCAATGTCTTGCAGAGAACATGCTAAAGGATTACCCTCCCATCACGTGAGTTTAGCAAACTGCCTCTGTTGTATCACAGAGAGGACTTGTTTACAAAGCAAACGCTGTCAGTCCATTTAATTAGATCTTTAACATGGTGAGAAATGTTCCCTGGCACTGAGCTCCTGGGAAGCCCTCCAAGATGTGAAATGGAGAGGTACTTCTTCGCAGAGAGGACAAACAGGGCTAGTTCCTCCCAACTGAATCATCCCTCCTGGGATGATGGTACAAGTTCAGGAAGCTGTAACAGCCACGGCCCGTAGAATAGCAATTTGTAATGGTGCTTGGCATAGTTGTGATGGAGGAGGGAAGTTTTCCCAGCTTGTTCTTTACGGTAAAATGGCTCAAGGTAGATTGTTCCCTGTCCCTCTGAACTGTATAAGGGACTCGCTGCTGCCTATGAAATGTGGCATCTATGAAAAGGGAGCAAATGTGCTTTATACAGCAGAAGACAACGGATCCCAGAGTCTCTTCTTTTCTGTACAACTTTAGCCCAAATACTGCCTGGACTGACTCTGGAGCCAGTCAGCTTAGCTTGTATAGGCCACACACCTTGGTAATACGGAGAACACTAGCATGACTGGATATTGCCAAGACTGCTCAGCTCCATTTGAGACCCTGCTTGTGGTTGCACTTTTGTCAGATTAGATTGCTCAGGCTGGAACTGCTCATACCACGATGCATCTTCCCCATGCAGGACAGAGCCAGAAAACATCAGTCTGAAAGAGAGCAGTTTTTTTCCTAAGTGTAGGATAAACACATTTGTAGTAGAAAGGGTCAGTTTGTAGAGAAACAAAAATGTTTCACAGCTCTGCATGATCTTGTTCCTAGAAAAACCCACCCAGACACGACACAGCTGAGTCACCACTGTACACCCAAAGCCAGGATCCCAGCCCTGGATGAACTGCACCTAGCAGCTGCTGGCTGCCCCAAGCTGGGTGTGACCAAGGGATGGAGAGGAATCTGCACACTGTGCTCTAGGAACCTCCTCCAAGCATCTGCAAAGATGAGTGTTGTGTCAGTGCAGAGACAGGAGATGGAAGAAGGAAAGGAACTAGGCCCAAGAACAACAGCAGGAAAGGCTTAGAAATGGGTCTGGGTTCTGATGCCAAATGGCTTTTCCATGAGTTTAGCTGCACAGTGAATGGTCAGGGAGGTTCCTAGGATGACATAAAACACTGGATTTAAGAGAGAATAGTTATGAAACCAGTTCATGGTGACAAACGTGTGCCATGCTGGTGATGCTGGGAGAAAGCCGCGTGGCTTCAGCTTACTATTCAGTCCAGCTGCAGTCCCCTTATGGCAGTGATGTACTTACATTGCCCTCCAGTGGTGAGCCTTGCATTTTGCCAGGTTAATGGAAAAACTTGGAAGGCCAGTGAAACACTCCATTTCACTATCAGTGTTTATCTCCCACAATTCAGATTCAGCCCCAAATCGTAACAGTGGGTTTGTGGTTAAACAGAACAGCGGGCATGACAATCACTCCCATCTTGAAAGCGTATCACCTTCAGCTGCACTGGTTTCAATAATCTGGTTTAACGCTTTCAGTTGCATGTCGCTGTCCTCCAATTACCTATGCAAGACTTACATGTAACTAAGTGTACGTGTTAAATAACAGGTAATAAAGCTACATGTTATTTTGAAAACCAGAAGTAAAAGCCTCAAGAAAAACCCTTCGTTTTTAGAATGTTGTCAACATTCCACTACCAGTCTTCAAACATTCCACATGAACGTCTCATTTAAGATTAGATCATATTTACATACATACGAAGACTTGTTTCCTAAGTCAGATTAATGAGAACAGGAATCACCTTTTCCCTGTTCCAGAGCCTCCCAGCCTGCAGGGCTGCAAGTCTTTTTGGAATTGGGAATGATGTGGTCTACGTTACTACCTGCAAGCTGCAAACTGCTGTGAACC

Novel00192

GGGAGACGGTTGCAGGAGCGGTCCCGAAGGGAGGAGGCTGCGGCAGGGCTGGGCTGAACGGGGCCGAGGAGAGGGAACAGTCTGGACACCACATTTATAATACCACGGGAAAACAAACAAGAACTTATCCAAGACTCTATTTTGTAAGAGGAAAATGCTGAAACGAGGCCGGGTAGAAGTGGGACGAGCACCTGGGTGAGAAGCAGAGGTGACCGATGGCATCGTTAGCTCGGCTGCAGGGGCGCGGTGTGCTCCCAGGCACGGCACTGAGCAAAACGGCTTTTGTGTGTCTGCAGTGCCTGAAAAGGCAGCTGACAAAGAGGGAGATGCTTTGAAAGGGCAGGAGCATCCTCCACGGAGCGAACGCTGGATTAATGAGGGAAATGCCTGCTGGCTGCCCAGCAAGGAGGGACGGTCCCCAGCGGCATCACCCCACGGTGCTCAGCGGTGGCCTGGGGACGTGGTGCCCGTGGGGAGCTGGATGCGACCCTGGGGATCTGCTCTGCTGGGCACCGAGCGCTGCGTTAACGCATTGCTTTTAATCAAGGTAATGCTCCCTTGTGCCCGAGCCCAGGCGGTGTCGCGGGGGAGGATTCATTGCTGAGCCATCGTGCTCCAAACCCTGACCACAGCTCTGCTCAGACAGCCCCACTGTCTGCCTGCCCTGACGCAAACTGCGCGGTGCTTTTGCTGGAGGCGCAGCAACCAAAGGGAATTCAAACGGCTGCATGGAAAATCGCGACTTTTCCACAGGAGTTCGGTGTTTGTGCATGGCTTTGGAGGTTTCGGGTTGCCAAGGTGTGCTGCAGCTCCCTCCTGCCACAGCACAGCTTCGTAGAACCAGAGCTGGAAGGATCATTGAGTCCAATCCCTCGTTCAGCCCCAGGCAGGAGGTGGCACGAGGCTGGTGATGGGGGTTCCATCCCCACGGTTCTGAACCCCGATTGCTGCTGCTCCTCTCCACGGAGCCCTCTCTGCTGCAGCAGCATTCGAGAAGGAGGAAGGAGCAATAACGTGCCTAAAGGGTTTTAGCCCATCCTGCACTCAACAGCCCCACAGTCCTCTAAACTGCAAAGAAACCCAAAGCCTGCAGTATCA

Novel00075

CCTCTCCTGACTTCATTTTTCCATTTTTCATTTGATTGGAAAGATAAAACTCACTGGGAGTCATGAGACTGCCCCTCTTTGTTCCCTGTCTGCTTTGCCTACAGCATTAGAGTAAGAACTTTGGCTTCAGAAACTCTCTGTTTCTCATTTTATCTGGTTTATTAGCTTCAATTCCAATCATATTGTATTATATTGTGTTATTTCACATTCAACTACTATATTTAGTAAATCAAACTTTTCCGTAGCTCATTGCTGCTGCTATCCTTCATCCAGGCCCATCTCCCTACCTTTTCCCTTCCTTCCTCCCCCCTTTCCTGGGGTGGGGGTCTGTGGGTCCCTTCACCCCCTTGTCATGGAACCTAGGCCAAACCAGGACCATGACAGCAGTACACCTCTTGGTTTGGTGCTGTGCACATATATCTGCTGAAGTAATTGATCTTCTGTGGTAAGAACTTAATAGAGTATTGATGGTATTTCATGCGAGGATGCGTCAGTGAGAATGGTATGTAAATACATAAATGTATAAAACAACAGGGGTAAAACAGAAATTCATAATGAAATCCCTGTTAAATAAACACACGACTTCAGAAGCAGTTGAAAAGTTAGATAAGAAAATGGCGTTGAAAGTGAGGAAAGGAGAGTGTGATAAATGGAAAGGTTACTAATGAAGGAAAAAGAACACTGTGCCCTCCTTCCCCACCTGGATGTGGTGGAAAGGTTTGCAGGGTGCAGTGACACTTCAGTATAGGCCAGCAAATTATAAAGCTCCCAGACGATGACTCACACCAGACAATTCAGAGCACAGGAGGCAAACAGACTGAAGTTAATCCACAAACCCTTAAAACAATGGAATATTGCTGTTCCTCTCTTTGCAGTCCATGTTCCATTTCTTTTCCTCTCCTTGTCATTGTTGGTTTGCCCTATCTGGACCTCTCAGCTCTCCATCAGTGTTGGCCAGTACTGCCCTGCTTTTTATAGCATCACAGAATCAGAGAATCACAGAATGGTTTGGGTTGGAAGGGACCTTGGAGATCATGTAGTTCCAACCCCCCTGCCATGGGCAGGGCTGCACCCACTAAGTCAGGCTGCCCGGGACCCCATTCAATTTGGTATTAAACACCTCCAGGGATCAACCTCAGCTTCTCTGGGCAGCCTGTGCCACTGCCTCACCGCTCTCAGTAAAAAAATCTGTCAGTAAAATAAAAATGAAAATGAAAAAAATCTGTCAATTAAAAAAAAGCAGTCGTTGCTCTGAACCACCACTAGTCAGTGTTATTGTCTCATATTGTCTATGTGCCGCTAGTCTTCACTGTCACCCCTAGCCCCTCCTGCCACCTCTTGTAACTCTATATCACCACCTCAGCTGGACCTACTGAGCTAATGTTGCTATTCCCTCTTACTTCTCACTTCCTCTCAGTGCAACATCCCAACACTTCGGGCCGTAATCCTGGTTTTGCCACCCGATGGTCTTCACTACTGTTAAAATCCCGCTCTCTGCCTTTTGTTGTTCTCCAGCTACTTCTCTCCTTCCCATTCTGCTTTGGCACACCTTGCCGTTTTGACCTATTACCCAGCACCGTTGCTCTATACCAGCCCCTGCTGCAACTGTTTTCTTCTGGCTGCCCAAAAGTAACACGCTGTTACTTCCACGGGGAAGTTAAGGTTACGGCAGAAATCTCTGCGCCTCTGCTCCAGTGTTGACAGAACAGATCATCCTGGCAGAACCCATTCTTGTGCAGGGTGGGGTTGGTCTATAAATGTGCCTGAAACTGAAGTGACTGTGAAAAGGCCCATCAGCACGGTGATGACGAGAGCAGTGACAGCAACTGCTAGGAGCAATGGCATTTATCCAAGGGTTTTAAAGCAAGTCAAGAGTACAATAGCTGAGCTGCTCCCAGCATTGTGTGACCCATGCTTCATTTCATTGTGGCACCTGGGGAGGGGAAGGCAGTAAATGTGATGCCAGCTCTTAAAGACTTTACGGGGGCACTGGATAAAAATGGACGGCTTCAACCCATGGAGGTGACTAAACCACGGAAAGCACATTCATGCAATTATAGAATTATAAAATCACAGTCACAGAATGTCTTAGGTTGGAATAGACTTAAAGATTCAGCCAGGAAGTCCATTACCTGAAAATGTTTGAAGGCTGGGAGAGTATGGCTAAGAACTGTGATATATTTGCCCCGTTCTTCCTCTTCAGCTTGAACTTTCCCTGAGAAGGGAGAGGAGGAGTTTAGAGAAAGAGAAATCAGGAAGAGATAAAATGATTATGGTGATGAATGGATAAACAGAAGGAGAGAATAAGGACCAAACAATTCAAAACACAAAACAGAGCACAACAACAGTACCCACTGCTTCCTGAAGACATCTGAGGGTGCCAGCTGGTGGATGCTGTGCTGGGATCCTCTGCAACAGAACAGACAGCAGAAGGGAATAGAAACATGCTGTGTAATTGCCAGGACCACCCTTACATCATGGATCCTCCTCCTGGAGCTGCAAGTGGCCAGGAACAGGTGGTGGAGGAGGTTCTGGGGCTTCTTGAGACTTCAGATGAAGATGCCATGAATGAAAAGATGAGGGATAAGTACAGATAGAGTCTAAGAGGGTCTGGATAAACTGGAAGAATGCCTAAGTTTGGCACACCACTTGATATCTTATGTCCCCAGTAGGCTGTGTGTCCATACTATCATCTACCAGCCTAAGTATGGTGTGCGCTGTGGTCCATCTCCTGAGGACTGAATATGTACTTGGATGAATCTTGCCTGGTAGCGTTGGAGCCACCTTGCAGAGTTTTTGGATCTTTGGGTGTTATGGTCCGGGCACTTTCACCCAGCTGCTAGTGAAGAGGACAGCAGCAGTGACAGAGGGGTGTGTTTGTGTAAGCACCAGGAATCTAACTGTGGGTGGTGGACCTCTCCTTTCTCTTGTTTCAGGGTTTGTAGGGGCAGGGTATGAGAGAAGGAGCTCCTGATGCTGCAGACATTGAATCCAGATGTGGACATTCCTCTGCTGTCCACTGCTGGGGAGTGAGAGATTCACTTCATGCGTCTGCACACGTGTACCTGACTCTGCAAGAATTCCTTGTTGCCTCTGGGCACAACTCAGCCCCACACTGCTTGAGCTACCATGGGCCTCATCATGGACCACTACCATGGATCTCACCAAGAACGGCTGGCAACTGCTTCTGGTCCAAAAGACAGGGCTGAAACCATCCATCCTCAGTGCAGGTCAGATCTGTGAAGAAGCTGCCATTGTATGGTTGTGAGATGGTCTTCTCTACAAACAGAGGTACCCAAGAAGAACTGTGAGGACTGCAATGCCAAGCTCTTGAAATGGAGATTCTTGCATCAAGCTTCTGATCTTCAAGACAATTACAGCAGGAGGGCATGAATGCTTGCCCTGATGCTTTGTCCCATCCCACCCAAATCACATGGGTTGCAGTGATTCCTTGCAGCTCATTGACAAAGACAGGAAGATGTGGGGAGGGCTGGTGTTCCTTCCAGGGTCACTGTGGAAATCTGTTTCTTGTTGCACACTAGGATCAAATGAATAACTGTCCATTGTTGCCTTGTGGACTGTATGGAATATGTGACTGATTGATTTCTGTACATTAGGCCTTTTGCTATAAGTGTGTGAGAGGTGCAGCTGACTGGTGGAATTCGAAGCTTCTCAGCCTGAGCATGTGGTACCAAGGTGGAGATTAGGTTTATGAAAGCCAGTTCTCCCTGCTGTAGGGAGCTGTATAATCATAACACTTCAGGCACAAAAGAAGGATTCTTCCTAGTCAGATCTATGATGGCCTTGGAGATTACAAGTAATGACAAGCACAGCCTGGGGGAGAGTGGACTCCTGGTACTTTCAGAGATGTCATCCTCAAATGTTTCAAGAGTGACATCTCCGTGGTTGAAAAAGCAACCCTTGTTCATCCTGTCCTTGCTGTGGTGCCTTAAAACAGGACCACTGAATTCCAGTAGTGTGTATACAGCTGCTGCACGGCTGGAGAGGGCTGCACACTGGTGGTCTGTGTGACAACTGACCACTCATATATGTCATCTGCCATATTCCAGAAAAGGGCTTTGAATCAGCAAAGCATCTGTTCTGCAGTCTGGCTCTAAAGAAGCAGTGTCCCCAGTTGCTCTGAGTGATGATATGCTGCCAAGTGGAAGCAAGCTCAGAGTTTATTTAAACACTTTACAGATGCCTTATTGCTGATGCACATTTGCTTTGAAATGCATACCAGCCAAAATTTAGCTATGTTCATAAAGTGCATCTAAACAAATGTAGTTAGACTGCTGGATCAACGTTGCTAGCTTAAATTGATTGAGCGAAGGAATAATTAAGCAAAGGGAACCTCTGCAGAAAAGATAATCCTGAGCTCAGATTTACTCACAGTGACAACTCTTTCAATATCACGTTAGGAGTTTTTGCAGTCTTCATTCACAATACGACAGTCACAGCTCCTAAGTAACCATGTATGTAGAGAACTTGACCTTACACTTTGCAGGTTGACAAAATCCCTAACAAATCTCACGTCTCGTATCTCATATCAAATTCCAATTCCAAAAATTGGAATTCCAAAAATTCCAAATGAGCTGATTTAATACTTCTATATTTAATATTTATTTGTATGATACAGCATACCCTGTGGCAGGTTGGTCTGGCTAACAACTGCTCAGATGGTGACCAGATCCCTGTTCC
