# Supplementary material for: Transcriptome Analysis of Differentially Expressed Genes Related to the Growth and Development of the Jinghai Yellow Chicken
Source: Genes (Basel). 2019 Jul 17;10(7):539. doi: 10.3390/genes10070539 (PMC6678745; doi:10.3390/genes10070539)
Supplement: Supplementary file 1 [file genes-10-00539-s001.zip › genes-527003-supplementary/Supplementary files 1-12/File S8 F8FvsF8S differentially expressed new transcriptsí» sequences.doc]

Novel01335

GGGGGGCGGTGGGATGTATGGGGACCCCATAGGGAGACATGGGGACAGCATAGGGGGACATAGGGACAGCATTGGGAGACATGGGGACACCATTGGGAGACATGGGGACAGCATAGGGAGACATGGGGACCCATAGAGAACCCATAGGGACACATAGGAACACATAGGGACACCATAGAGAGCCATAGAGAACCCACAGGGACACATAGGAACACATAGGGACACCATAGGGAGACACAGAGAGCAATAGGGACCCCATAGGGACTACGTAGAAAACCCATAGGCACACATAGGGATGAATAGGGACCCCATGGGGACACTGCGCCATTGCGGTGTATGGGACCGGCGTGGAGCCCAGGGAGAGTGGGGGGGGGTGGGAGGGGGGCGGTGGGATGTATGGGGAGCCCATAGGGAGACATGGGGGCACCATTGGGAGACATGGGGACACCACTGGGAGACATGGGGACACCATAGGGAGACATAGAGAACCCACAGGGACACATAGGGACACCATAGGGACACATAGGGACACCATAGGGAGACATAGAGAACCCATAGGGACACATAGGGACACCATAGGGACACATAGGGACACTGCGCCATTGCGGTGTATGGCACCGGCGTGGAGCCCAGGGGGAGACATGGGGGCACCATTGGGAGACATAGAGAACCCACAGGGACACATAGGGACACCATAGGGACACATAGGGACACTGCGCCATTGCGGTGTATGGGAGCGGCGTGGAGCCCAG

Novel01285

CCCTGGATTTGGGGTCCCAGCCCCCCCTTTTTCCCCCCCAGATCGAGGAGATCTTCAAGAAGGCCATTGGGAACTTTGGGATGCTGGGTTTTGGGGTCCTGGGCTTTGGGGTCCTGAGGTCCCCAGGTTTTGGGGTCCCCAGCTATTGGGGTGCCTGGATTTGGGGTCCTGGGGTCCCCAGGTTTTGGGGTGCCTGGATTTGGGGTCATGTTGTCCCCAGCCATTGGGGTGCTGGGCTTTGGGATGCTGGGCTTTGGGGTCCTGGGGTCCCCAGGTTTTGGGGTCCCCAGCTATTGGGGTCTTGGATTTGGGGTCCTGGGGTCCCCAGCTATTGGGGTGCCTGGATTTGGGGTCCTGGGGTCCCCAGCTATTGGGGTCCTGTGCTTTGGGGTCCTGGGGTCCCTAGCCATTGGGGTCCTGGGGTCCCCAGCTATTAGAGTCCTGTGCTTTGGGGTCCTGGGATCCCCAGCCATTGGGGTCTTGGATTTGGGGTCCTGGGGCCCCCAGCTATTGGGGTGCCTGGATTTGGGGTCCCCAGCCCCCCTTTCCCCCCCCCCCAGATCGAGGAGATCTTCAAGAAGGCCCTTGGGAACTTTGGGGTGCTGGGCTTGGGGGTCCTGGGGTCCCCAGGTTTTGGGGTCCTGTGCTTTGGGGTGCTGGGTTCCCCAGCTATTGGGGTGCCTGGATTTGGGGTCCTGGGGCCCCCAGCTATTGGGGTGCCTGGATTTGGGGTCCCACCCCCCCTTTTTCCCCCCCCCCAGATCGAGGAGATCTTCAAGAAGGCCGGGCACCCCTTCATGTGGACGGAGCACCTGGGTTACATCCTGACGTGCCCCTCCAATTTGGGCACGGGGCTGCGGGGGGGGTCCACGTGAAGCTCCCCAAACTCAGCCAGCACCCAAATTCGAGGAGATCCTCCATAGGCT

Novel01239

TGGGGTTATTTGGGGGTTATTTGGGGGCATTTGGGTCATTTTGGGGTTATTTGGGGGCAATTTTGGGGTTATTTGGGGGCGGTTTTGGGGTTATTTGGGGCCATTTGGGGCAATTTTGGGGTTATTTGGGGGCAAATTTGGGGTTATTTGGGGGTGGTTTTGGGGTTATTTGGGGCCATTTGGGCAATTTTGGGGTTATTTGGGGCCATTTGGGGCAATTTTGGGGTTATTTGGGCTGATTTTGAAGATATTTGGGGCCATTTCGGGCAATTTTGGGGTTATTTGGGGCAATTTTGGGGTTATTTGGAGAGAATTGAGGGCAAATTAGGGTCAGTTTTGGGGTTATTTGGGGCAATTTTGGGATTATTTGGGGATAATTGAGAGGAAATTTTGGGCAATTTTAGGGTTATTTGGGGCCATTTGGGGCAATTTTGGGGTCATTTGGGGGTAATTTTTGGGTTATTCGGTGGCAAATTTGGGGTTATTTGGGACCATTTGGGGCAATTTTGGGGTTATTTGGGTGATTTTGGGGCAATTTTGGGGTTATTTGGGGACAAATTTGGGGCAATTTGGGGTCATTTGGGGCAATTTTGGGGTTATTTGCGGGCGGTTTTGGGGTTATTTGGGGCCATTTTTGGGCAATTTTGGGGTCATTTTGGGGCAAATTTGGGGCAATTTTGGGGTTATTTCAGGGCGGTTTTGGGGTTATTTGGGACCATTTGGGGCAATTTTGGGGTTATTTGGGGGCAATTTTGGGGTTATTTGGGGTGATTTTGAAGATATTTGGGCCATTTCGGGCAATTTTGGGGTTATTTGGGGCCATTTTGGGCAATTTTGGGGTTATTTGGGGGCAATTTTGGGGTTATTTGGGCTGATTTTGAAGATATTTGGGGCCATTTCGGGCAATTTTGGGGTTATTTGGGGCCATTTTGGGCCATTTGGGGATTATTTGGGGGCAAATTTGGGGATTATTTAGGGCTGATTTTGGGGCATTTGGGGGCAATTTGGGGTTTTTAGGGCCGATTTGGGGGCATTTGAGGGCAATTTAGGTTTTTTAGGGTTGATTTTGGGGCATTTGGGGGCAATTTGGGGTTTTTAGGGCCGATTTTGGGGCATTTGGGGGCAATTTGGGGTTTTTAGGGCCGATTTTGGGGCATTTGAGGGCAATTTGGGGGTTTTTAGGACTGATTTTGGGGTCAATTTGGGTCAATTTGGGTTTTTTAGGGTCGATTTTGGGGCATTTGGGGGCAATTTGGGGATTTTAGGGCCGATTTTGGGGCATTTTGGGGCAATTTGGTGTTTTCAGGGCCGATTTTGGGGCATTTGGGGGCAATTTGGGGTTTTTAGGGCCGATTTTGGGGCGGTTTAGGGCAATTTGGGGTTTTTAGGGCTTATTTGGGGACAGTTTGGGTCATTTTGGGGCAATTTGGTGTTTTTAGGGTCGATTTGGGGTCATTTGGGGGGCAATTTGGTGTTTTTAGGGCCAATTTTGGGACATTTGGGGGCATTTTGGGGTTTTTAGGGCCGATTTTGGGGCATTTGGGGGCAGTTTGGGGTTTTTAGGCCGATTTTGGGGCATTTGAGGGCAATTTGGGGTTTTTAGGGCTGTTTTGGGGTCATTTTGGGTCAATTTGGG

Novel01054

AACCAGACCCCTCCGGCACCCCAAATCGGAGTCCTCCAGAACCAAACCGGACCCCTCCGGCACCCCAAATCGGAGTCCTCCAGAACCAAACCGGACCCCTCCGGCACCCCAAATCGGAGTCCTCCAGAACCAAACCGGACCCCTCCGGCACCCCAAATCGGAGTCCTCCAGAACCCCAAACCGGACCCCTCCGGCACCCCAAATCGGAGTCCTCCAGAACCAAACCGGACCCCTCCGGCACCCCAAATCGGAGTCCTCCAGAACCAAACCGGACCCCTCCGGCACCCCCTGCACGGCGGTTCTGCTGAGCCCCATTTCTCCCCGGCACCCCCCACCTCCAGCAGCCCGGATCGTCCTCCAGCTCCGTTCTCCCCCCCCCCCCATGGTGCCCGGACCCCCCCCGGCTCCGGCCCAGCCCTGTACATAGCCTGGCACACAGCTGTGTGCCTTTGTACTTTTCTACCATGCCGTAACGTCGCGCCTCCATCCCTGCTGGCACAGCTCCGCCTCGTGCCCACCTGCACACGCATGTGAAACCAAGCGCAGCCGCTAACGAGCAATTAATAAACCGTACACCGCCGCCGGGCCACGCTCAGAGCAATGGGGGGAGCTGAGCCAAAGGGGTGCGGGTGGGGACCCAAAGGGGGTCCGGGGCTCTCCGTGCACCCACCCGGCGCTGAGCGAAGAGGAGGCGCTGCGGAAAGGATCCGTTTAATAGCGGTGTGGGTGTGCTCAGCACTGCATGCACACGCGTGTGCACGTGGGTTCACGCGTGTTCCCGTGGGTTCCCGTGAGTTCACGCGTGTTCCTGTGTGTTCACGTGTTCACATGTGCCCGCGCGGCGGTGCGCATGCAAAGCGCGTGTGCAAGGGGCGTGGGTGCGTTGCACACACGTGTGTGTGTGTGTGTGTGGTGTGTGTGTGCAGGAGGGTGGGTGTGCAAATGCATGTGCACAAACCCACGTGCACAGCTGTGTGCCACCCCAAGCCGAGGAGCTGCGCCCTCAGAATGGAGCGCGGGGGGCGCCGGGGGGGGTCCTGGGGTCACCCTGTTGTGGCATTGGGGACACAGAGGGGGGGTTCTGTCCCCATAGCGGAAGGGGGGGGTGGGGGGGGGAGATAAAAAAAATCCGTCGCGAACGCCGTCGGAATCATAGAAAATGATTGGTAGAAAAAAAGCCACGCGGCGCTTCGGCTCCAATGGGGACAGCGGCACCTGGGGACAGACGGACGGACAGAGGGACGGGTGGGGGACAGGGACAGCGGGGGGGTGCAGCCACCTCCCTGTCTGCTGTCACCCCCCCCACTGCCAC

Novel00723

AATGCGACTTCCGGAATGCGGCTTCCGGCTGCTCGCTGTGTGGGGTTTGGAGGTCGTGCCCGGGCGCTTCCGGGTGGGTGAGGCCTTAAAGGCCGTGCGGGGCCAACGCGGGCACCGCACCGTGAGCTGGAGGACCGCGGTGTGCTCCGGGGAGCCCTGAGGTACGCCCGTAGATCCTAATTCTGCTCTGGCGTGCCCTGAAGTCCTTCTGCTCTGCTATGGAGGCCTTCGAGGCCTATCCCAGCACGTTGGCACAGCACACAGTGCCCTCAGGAGCCCTCCGTGCGCTCCAGGCCGTCTTAGTGCAGGCCTCCAGGAGCCCCCCCCGTGCACTGTGGGGGCCCATGAGCACTCTGCAGGTGTCCATTTGCTTCCTGCAATCGATCCAGGGCGACAGAGCGGCCTCGCAGTGGTGTCTTCTAGTTCCTATGGGCTGCATCCTATGATCCCGCAGACTCCGCCACTCTGGATTTGATCCAATATTCCCCGACCTACTTTGTTTTCCCAAACGCGCTCCCAGAGGAGCTTCTTGTTGGCTTTGCTGTATCTGCTGACCGACGCAGTTCTGTTGGAGTTTTGCTTTCTCAGCTCCGTCCCTGGGGGCTTGGATGATGTCTGTGTATTCCCAGTGTTGTCCCAGCGTAATTTGTGCCCGTCTGGGTGAAAAGTGGGGTGAAGTGCTCCGAGGAGGGGCAAAGCGGGGGGGAGGTGATGGAGGACTTTGCAGAGAGGGGACGCTGACCCCAAGGAACAGGAAAACACGACCTCTGCTGCAGTTCTG

Novel00671

GCCCCGCAGCGGTGGGGATGGCAGCATGAGGTGTCTGCTGCAGAGCACAGAGTGAGATGAGCGTAATGAAATCACAGAGTTTACCAGGAAAACATCATCCTGCAAGAGGGCAAGAAGCGCACACGTGAGCCTGTTGCTTTAAGGGATGCTGGGTTTTCCCATGGACTGACTCAAAGTGGAAACTGTGCTTTCTCAGCACGCAATGTAGGTCAGGGCTGCCGGCAGTCGCTGCTGCAGCTCGCTGTGACCAGCAGTGGGGCTGCAATGGGTCCCCATCCCATCGCCACGGGGCAGCAGCCCCTCTCCTGTTGCCACCGCTCGGCTCCCACGTGCTCCTGACCCCGGTAACCATCTGCAGGGTTGCAGGGCTGGGACGGCTTCTTTCTGCTTTGCTCTTTGCTTTTCATCCATTCAGCAACAGGGGGCTGGGGGATTTGTAGCAGAGATGGGATCAAAGAGACATATCTGGAGATGAGACCGCGCTCATTCCTTCTGGCTTTGGAATTTGCAAGCGCTGTAAACTGGAATGAAAACGTGAGCGTAAGAAATGCCCCGAGGAGCAGAGCTGTGACCTCACGCTGAGGTGTGCGGGCAGCTGGGAGGCCTTCAGAGCCCCTCCCTGGGTCGGTTTGGCGCCTGGCTGTTCCTTATGAACGCTTTCACTGTGCTTGAAGCAAATGAGGTCAGCGCACACATCCCGAAGGCTATGCCCCACTGCCCCACGCAGGCAGCAGCCGCAGCCCAGGCCATGGTGGGAGGCGGAGAGGCTTTCGTGCTCCATATCCTGCTCTGCATTCTGGGCAGAGGTGCTGCCCAAATCAGGTGCCTGGGGCACGTGAGGATCGAGCCGGGCTCCGTGGTGCCAATGGGTTCCAACGTCTCCATCACCTGTGTCTCGGCGCTGGACTGCCCGTGGGCGAGCCTCTCCATCCTGCTCAACCTCACAGCCCCCAAGGATCCCCCACGCCCTCTCAACAGCAGTGCAGCCCAGCTGCAGCTGCGTGGTTTCCGCCTGCCCTTCAGCATCATCACCTGCCTCGCCCGCTGCCCCAACAGCTACTGGAACGAGGTGGTCTGCGGGACGGAGCTCTGGGCCGGCTACCCCCCGGGCCCCCCCACCAACCTGAGCTGTGCCATCCCCGAGCGCTCAGGGAGCCTGGCATGCACATGGGACGCCGGGCAGCCAACACTGCTGCACACCAACTACAGCCTCCACCTGCGCAGCATACAGACGGCACAGGAGGATGTGTTCCCCACGGGCTCACCAGTGCCCTTGAGTGCTCTGAATGGCAGCAGTCGCTACCTGGCATGGGTGCAGGCCAGCAATGCACTGGGCATGGCACGCTCAGCCCCTCTGCACCTCGACCTGCAGCAGCTCGTGGTGCCCGCCGTGCCCCTGGCTGAGAGCGCAGAGACGACGGCGGGGTCACCTCCCACCACCACCATCCGTTGGCGACAGCAGACAGAACTGCAGGACGTGCGCTGCGAGGAGCGGCACAAGGCAGTGGGTGCTGCGGAGTGGCATGTGACAGCATGGGACAGTGCAGTGCAGCGTGGACACCAGCTTCAGAGTGCCACCCGCTACGTGTTCCAGGCACGGTGCCGGCTCAGCGCTGCAGGCAGCCCCTGGAGTGCTTGGGGCCTGCCCTTCACCTACAGCACACCCGAGGCTGCCCCCGCGGCAGCACCCGACGTGTGGCGGCGGCTGGGGCGGCTGTTCCCCAACGGCAGCCACGAGGTGACAGTCTTGATCAAGCCGCCCCGCGATGCCCGCACGCCCATCCTGGGCTACGCCGTGTGGGCTGGAGCCTCGCGTCCCCTCTGCAATACATCGAGCACCGCCTGCAGCCTCCTCCTGCCTCCGGCACTGCGCGACCTCCGCGTCACCGCCTACAACGCCCGGGGGGCTTCGAGCCCCGCCCGTGTGACGCTCCGCCGGGACCCCAGCCCGGAAGCCTTCCCATCACCACTGGCTGTGGATGTGCAGCGTGAGAACCAGAGCACCATATCTGTGGCCTGGCAGCCCCCCCGGCACAACGGGACCCCCCCACTCTGGTTCATAGTGGAGTGGGTTTGTACAGCTCCGTACAGCCATGAGGAAGAGTTCTTTTGGAAGAAAGTCCCAGGCCAGGACAGCCACACCTACATCCAAGAGGACGCAGCAGCAGGAAGCCACATCAACGTGTCGGTGTATGCAGTCTACCCAAATGGAGTGAGCAAACCAAGCTCTGGCCAAGTTTCTTTGGAGGACCAGATGTTGGGCATTATCTACCCAGAGGCTTTGCATGGTAAGAGCAGAGTATTTCAGCCTTGGTAAGCAAGAGTTTCTGCCTGAACAGAGAAGTTTTGGCCAGAGCAGCAAGTGTGGCATCTGGAGATATAGGAAAAGGGAAGAGAAAACTCCACATACTTTGGAAAGGTCAGAGGCAGAAAAGCGATTTTCACTTGACAGGAAAGTAAGCCACATAGTGAGAAAGGCTTCCATACTGGTGTGGGTGAGCAGAACATGCATCATCCTCCAATTTAAGCTTTGTTTTCTTGTGAGACATGTAATAAAATGTACATTCCAGAAAATTGGAGGTAAGAGAACAAGGAAAATAAAAGATCACAGAATCATAGAATGGCCTGGGTTGAAAAGGACCGCAATGACCATCTCGTTTTAACCTCCTGCTACGTGCAGGGTCACCAACCACCAGACCAGGCTGCCCAGAGCCACATCCAGCCTATGAAAAAAGAATGTTAAGAGATACAGTAGCATAACAGTGACTGACTTCTGCCCTTACAATCTGAGCCAAAGACTTAATTAAACATTTCCCATTTATTTTTCTGACTGCAGTTGTGCTTTGCATGTGTTCTGGTAAAGGTTATGCTATGATCTGTTTCAGTATTTAAAACAAAAACAAAACCGATGTGAACTGAAAAATTCACTTGAGCATTTCTGGAAGGTACCTCTGCGTGGGAAATGGTTAAACACAATGACCACACTTCTTAGAATTACTTGCCTCCAGCTCCCATCTGCACAGCAAAGGGCCCTCAGTGCTCAGCCTGGTGCTTCACGGCCTCCAGCTTTAAATGCATGGTGTTCTTCCTGCAGACCATCAGAACTGTGAGGGTAAACCTGTAGGGACAGCAACAGAAATGCAAGCAGTTGGGTTCAGCATGACTGACTGTGGATGCAGAATTATTCACAAATGACATCAGGCTTTTCCTGGGAACGGGTGTCAGTGTGCTCATATTATCGGTGGTTCTTGTAATTCTGATGTTTAAAAAATCAGCTAGAAAAAGAATTAAGGCAGCAGTGGCACTGGTTTTGCCAAAATGGCTGCTTGAAGATTTCCCCCACATGGAAAACAGCAAGGTGATAAAGTCCTTCCAGGAGAAGAGTGAATTCACAAACAGCATTTTCAGTGAGCCGTTCCTGGATAACAGTGACCCCACAATTACGGAAATAAAGGAGCTGTCAGGGGACAAGAAATACAAGAACGTGGACATCAAAAAGGAACCCAGCAATGTGGTCCCTGAGAACGTGGAGCACCCACAGAGCTCAGCACCTCCCCATAACACGGCCACGGAGGACGTCAGTGACTACAAACCCCAAATCTCTGATGCAAACACACTAGGATATGTAGCTGCCAACATGGGCCTGATACAGCCCTACACACCAGCTCCAGAACCAGAGACAAATATTTTCTTCCGAGACTACAGCAGCCCTTTTAGCTGCCTGTGGGATGCTGAAGGAGCAGGGGCCCAGGTGTGCCTGCTGGACAAGATCAACCTGGTTTTAAACAACGACAGAAGTGGACAAAACCACGCATTCAGCTCCGCCCAGGAGGAACAAAATGCCCTTTTGGAAAACCAGTGGGAAAAAACACTGTACAGTGAAGGAGTTCAGGAACAAACCTTGGTTCCAGATGAGCTGGTTTCTTGCTTAAGGGCAATGAATGGAGGATCTGTGGATATACAGACCTGCTTTCCCCAGAGCATCGGAAGATTGTTTTGAGTGAAATTGTGATGAGAAGCCCACAAACTTGTGACAATATGATGGTGACACATATCTGCTTTTGAGATCTCAAACATCAGAGCTGGAACAAAACTCCAAATCTGAAACTCTTCCCCTTAACTGACTACAGATAGCTTTGGATGCATGGAACGCCTTGGCCAGTAAAACATCATTTAATTTTACCTCATGTTTTTACAAACACAAAAATGTGAGCTTAGATTCCTGTAGGAGCTGAAATATAAAGCAGGTGATTCTTACAGGATGCATTCTGCTTCAATTATTAGCCTAAGGCACTCTCTTGCTAAATATACAGAACAAGTAATACCTGGAGCTGATTGCCTTCAAGATGGATAAAACCATCCCCTAGGTATTCTGTCTGAATTGTGTAAAATAGCTTGGGATCACCCATAATGAAGAGACACTACAATGAGGACAATTTACACATCACGCAGCAGCCATAGATACCATACGAAATCTTTTCTGTTTGACAGAATAGCCCAATTTCATTTTCCTTTCCATTCAGTAAGAAAAAATACATCAGATAAGAACAAACCTTCATCATCTTGGTCTGCCAGCCCGAGTGAAAATCTCTGTCTCCATTTGTCACTGGGCAGATTCTTCGTTTTGGATGAAGGATTTATCATCTCACATCAGTTTGTCTGCAGTTCGGCTTTGCTGCTCTGTACGAAGCAGAACATGGCAGAGAGAAATGGCATTTATTCAGTGCATAAGAGGAGATTTGGATCTTCTGAGATGCAGAAAACAAAATATCAGAGTCTCTTTCCTGCTGAGTGGTCTTGAAAGCAGTTAAAAGATTGCCCAGGATGTCAGAGGTAGCCTTGCAAGATGTTCTAGAAAGCAGAAACACCCTGGGAATGTGAGAGGTTGGATCATTATCACTTCTTTGACCGATCACCCCAACTTTTTGAGGAAACACTTGAAATGCTCTGAAGGAATACAAGCATAGAAGTTTATCATACCCTGTAAAATGGCTGAGCATTCCATGAGCAATGCTCTCCCTAAGGTTTGTTCTACTGGTCCTTCTCTCCTACCTCTTATCACAGCAAAAACTGTTCTTATGCCAGAGCTGCATCAGGACGTGGCAGAGCTGCATGCTCAGGGTGGGCAGGAGTTAGTGCCATGCAGCCAGCACTGGATGGGGCTCAGGGTGGAGCAAGGGGAGGGCACTGGCCCTTAAGCCTCCTCCATGCTGCTCAGAGCAGGACCTTGGGCACTGCGGCACTATATAACAATACGTAGCACAAAGGAGCCCCTGAACTACGTACCAATACGTGTGATTTCCACTCACTGAGGCATCTGTGCATCTTACACAGCTCTGGGTAACGGAGCTGGCTATGCACGTTGCATGGAACACACACAGGGGATGTATTTTGTAAGACTGACATTAACAAAACAGCAAAAAAAAAAAAAAAAAGGGAAAAAAGATCACCTGACTGTGCATTACCAAAGCAAAGGCTGTAAAACAGTCATAGCAAGACTCAGTACATTTTTATAAGACTTACAAAGCTAACATAAATGCTAACCTACGTGTAATTAGTTGTCTAATATTACCCACTTGATGACTGTTGGCAATAAAACCTGGAGATTGTAATTTTCCTTCTGAATGTTGCTAGGCAATGTCAGTTTTTCTCTGACAGCTGTTGCACAAATCGATCAGTTTTGTCATTTAGTCCTGGACACCTTTAAGCCAAAATAGTTGTGTGCTATGGTTGCTTCCTCTTAGCAATCAAGAGCTCTGCCATCTGTCACTTACATTCACAGAGCAGCATGGCACCTGGAAAGATGGGATAGGTTTGTTCTTAAGAAGTAAGTAGCAAAACTGTTTCCAAGCCTGAAATGTCACTCTCACATGTAAGCTAGTCTAAAATTAGTGATTGCTGCCTGTGCAGGAAGGAAATTGTAAGAGAACAGCCACATACAATCAGCAGCCAAAATGTGAGGTGTAGAAGGTATTTTTTTCAATGTCCACATCCTGAGACTTGAGCTGCCTGTGTTACCTGAGCTACCACAATACATGGGGTGTTCGCTGCTGCACGGAAATGCTCATCCTTTCAAATACCAGGACTGAAAGTGTGGTCTTACCTTGCAGGTTGCTGGCTGAAGGCTGTCACACTAGGAGAGATACCTGTGAGTATGTCAGTGCTCAGGAGGAGGTCAGCTCCTGCCCTGCTGAACCTATCGCACACTGGTTTGGGGCTGAAACCTTCGCCCCTCTCTCACTGTGCACACATTAACAGCAGAGCTGCCTTCGGTTTCACTGGGGCTGTAGGATTTGTTTCTTGGCATGAGTGGATGGTGCAACCAGAAAATACGGGCAGGTTACCAGGAGAAAATGGAAGGAGAAGGGCAAAAAAAATAGACAGTTCTGCTCCACCTCTCCCTTTTAATCTATGTAGATGGTGGTAACGGATCAGGTCTGAACAGATGCCTGCGATGCTTGCCTCTGGCAGTGAGTGCTTTGGGGCATGAGGACAGCTCTCATTTTGTCAGTAGCCCAGTGCCATGTTGTGACCTCAGCTTCACATTTGACTTGTTAGATTTGCTCTACACTCCGCTTTTTGTCTCTGCCTGCTTTGACCTTACAAAGGCCTTTTCACCAAGCCTGGGCCTTCTTTCCACTAAGTGCAGTACTTAGTGGTAAAACCCAGCATCCTGAGTGCCAGGAATTGAAGTAGCTGTCGTGCAAGCACAACACCAAGATCCTCGTAACGGGAAACCTTGAATATATGGATGGCTCAGCATTTGTAATCAAATAACAATTACATTCTTAACACACTTATTTTAGCATCTCCGAATTCACCTCTTCCTCAAATATGACATGCTCTCTAATTCTTGGTATTTGTGTTTTCGAAGCCTTTTTCTAAGCTTGCTTATATTGCTGGCAGCTGTGTTGCGTGCCAATGAATTCTACAGGTTATTTACCAGTTTGGCAGTGAGCTGCATGGGTTCTTCACCGTAACTGTAACATGAGCTCTGAGTACATTGGAGGCACACAAAGGAACCATAAGCCAACTGGTGTCCCTACACACAGACAGTATACACCAACAAGCACCAATCTCCCACGTAACTATCACTGATCTGTGTGAAACTGCAGAGTCGTCTAAGCATAGAATGGCCTGGGTTGAAAAGGACCACAATGCTCATCAGTTCCAACCCCCTGCTGTGTGCAGGGTCACCAACCAGCAGCCCAGGCTGCCCAGAGCCACATCCAGCCTGGCCTTGAATGCCTCCAGGGATGGGGCATCCACAGCCTCCTTGGGCAACCTGCGAACAGTGCATCACCACCCTCTGAAAGAGCTCAATGATGGGAGTATTACTTGTGCAATCCACATCAGTTGCCACTCACTTAAATGTAAAGTAATTAAATTTCAAGGCACTATAGCTCATGACACACAGATGTACCACGGTGCTGTTTCTCATTGCTGTTCAAACCAACCTCAGTGAAAAGGGTCTTTTCATACAGATGGCAAAAGAGAACAAACGAAAAAGGAATATGAAAATGAAAAGCAGTCTTTTCAAAGCTTAAACAAGCTCTGACCACAGCAAAGCCATTCATTGCTAGCTCTTATCTCCAGCCCATCGTGTCAGCATAGGGTGTATCAAGTGTGAACTTGTACCAGTGTAAATACAGATGGGAGAGTTACTGACAGCACGGCTCAGGCAGCGGCACTGCCCCTCTGGGATTTGTTGCATACTGTCATCCCTCAGAAAGTATGCTTGAAACGAAATGTATGGCAAGTTATGTATTAAATATGCAGAACTAGTTGCAATTTTTAATCATGTATGCTGCTGCATAGCCACCACACCACGCGTTCTCCTGGAGCACATAGCATTCTGTCTGCGACTGAAGCACAAAGGAGATGACTGCTGGTCTCAGCTCAGCCACTGGCTGTGTGACCTTCAGCGAGTTACTCTGCCTCAGCTTCTTACTCACAAAGCCGGGACAGCAATAATTACTATTTCTAATAAGGCCTCAGGATCTAGAGTCCAAACGCCGTGTGGCATCCAGTAGTTGGAACGGTACAAGAATATGAAGATAACGGAAACGTTCAATGAGTAATCTCGTGCTGCTCATTTTATTCTGCACATGCATTTGCTTGACTACTCCATGCTGAAAGATCTACAAACACTTGTCTGTCTGTAAATTGTGAGAGATTTAGCTACAAATATGCTGCAGAGACTAAAATTGGATGGTAAACTGAATTTAAAGGTTCACAAAGCTAAATAACAGCTGTAAACTGGGGATTTCTGGATGCAAAGGCAGAAGACCAAAGCCGAGGTTGTGATCAGGACCTGCACACAAAGCTATGAACAAATGCCCCAATCAGCAAAATCATTCTGAAAACAAGGCAGCAGAAGAATGCCAGCAGGTAAAATGGTCATGCAATGACTGTATGAATATTCAATTAAAAATATTCTGTTTGGAGCGTCTCATCACTGTTAGCCTTCAAGAGGATCCATTCTTTAAGAGGGAAAAGTAGTTTTTCAAAGGGTAGGAGGGCTGAGCACAGCAGCAGAGACTGAAACTAAACTTGAATTTTGTAGAAGAGGATGTGAGAAGCGAGGCTGTGGTTTTACATCTTCAATGGCTTCACTGAACGCTAGCTTCACAAAGTCAAATCCTTTAGAAATCATACTTATAGAACAACGGGAGCAGCACTGTGATAAAAGATCAAAGCAAGCATTAAACACAATGAATATAACACAAATTACAGGAGTGATCTATCAAAGGAGATTTCAAGATTGATATTTACCAGAACAGTCACATCTTTCCTTAAGAGAAGGGAATTAATCTTGCCACAAAAATATTAAAAACAATAATGACAAACCCCCTCATTCTTAACTTCATAGAAGGTGCAGTATGACACTGCATCCTCCGTAGGATTTTCTAGAATTGCCCTGTTAGACTCGTACTCTCAGAGTGAATTCAGCCACACTTTGCTTGGTTTCAAGGAGGCAGAGCAACTGTGCTTGCAGAACTAAGTAACCAATTTCATTCTGCTAGGTAAAGGCAAATGCAGACTCACTGGCATGAACAACGGTGGTAAACCCAGCACACGGACTACCTGAAATACTGAAGAAACAGACACAGAAGTCATCATTACCCGAGGTACTGCTACAGCAGCTGCTTAGTATTACTCTTGCACAGCAGGCAAAACCTGACATGTGAGCAGCTGAAGCTCACTGAAGGTCTGGTGGCCAGAAAGGCAGATGGAATGAATGGACCAAGCACAGGGCAGCACATGCTTCAGCCATGGTTTGACATTCAGCAGCGGGGCTGCTTTGGCCATCCTGAGCAGTTGGGTCTCCTCAATCACCCTTAGGGTTTCTTCTTCTACTACGACATGAGTCGTGGTCTTGCACAGTAAGTTGGATAGCTTTCCAGCCAGGTGTTTCCCTGCTTTGAATCACCCAGCTAACACCAGCACAGTGGCCTCCGTCACCAGGGTGGGAACTGCACAAACCACAGGTGCTGCCAGAAAAGAGATCACTGAATGGTGCCCTGAAAGAAACTTTTGCAAATGGCTGGCTGGAGAAAAGCATTTAAACAAAAGGGCAGGGAAACCTACTGCTAATGTGGGAACTGGGAGGGCATTGAGCAAAGCTGACCACGAGGCCTAAAGAGAAAAGGAAAGGAGGGAGGCTGTGACGTGCATGCAACTTGCTGAAACGTGGAGCCTGAAGGAGAGTGCTGCTTCTAATCTGTAATGAGCTCAAACCATTACACTAATCATAATTACAATACCAATATTTGTGCTTGTCAAGCATTACGACTGCCTTGGTGTTGCCTCGTGCACCATTTGGATGTGCAGAAGCACATGCAAAAAAAGCAACCTTTACACGACACCAACACATGAAAAAAGCAACCATTTACCTTTCAGGACTCTATCACTGATGACAGTCACAGCAGGTTTTGGGGGTCGACTCCCATTGCCTCCCATGATTTTTCAAGCACTCAGTAATTAACTTGCAGCGAGCTGCCCCTCCTCACTTATCATTTCATTGTTGATGACTCAGTACCTTTCCTTCACAATTACTCTTTCTCAAGGTTATTTCCAGATCTACAACTTGACATAACCAGAGAGTGTGATGTCTGCACCTCCTGATTTCACTTTTGAGTTTGAATGCTGTGTTCTCCAGCAAAAACTTTAACACGAGCTCTCGTCTGTATTCTGACCCGGCCCCAGTCTTCTTCCACCATCAGCTCTAACTTTCTCTGATGCTCATTAAAATATTTGGGAAACAGGTGGCTTTTCAATATTTGGAGCTTACTGTACTTTTAGATAGCTTTTTTTTTTTTCCCTTAGAAACCCAATGTAGGCGCTGGCTGAGTAAGCCCTTCCCAGCTCTCTGTTTTGCATCACTTTGCTTTGCAAGTCTCCTTTTACATGTAAATTGATCCCAGGTAAATTATTTCATCTTTTTCCTTCCAGCAGTTGAGCAGGAATCACCAATGTCACACATTCAAATTTCATTACATCATTTATATCTGATTCCCTCCCCCACAGCCTCCCACAACCACCACCACCACTATATTCAGGAAAATCCATATTCTTTGCTCACACTTTTTCTCTAATCCCAGCATCCATTGGATTAGGAGAGTGAGTAGAAGGATACAGCACCTTGACCTCAACACGTTATTTACTTACTAACGGTGGTTCCTTTCAGATTTTTATCTCTGGAAGCTTGGCAGCACCAGACCTGTGACTCATATCCCACCTGTGAGAGAAGCCGTCCCTTCCCCTGTTTCTGTAATCACCATGATTTGTTTTAGGCAGTAAATATCTGATACAACACACTTTGTAATTCTCCCCCCACAAGTGAACTCGCACTCTGCAGGTTGCCTTATCTCTTGCACGTACAGTTCTGCTTTGTCACATTCTCATTTTGATCATACAAACTTCACCTTTGAGAACTGACTTAGTGGAAGAGCAGCACTGCTGCTCACCGCTGGCAGCTGTGGATCTCAGAAGGCACAGCTTTGCCTGGGCTGCAAGAGACAACACTGCAACCATTGGTAGTGCTGTGTTAGAACCTTTTCCTTTGGCCTCTTCTGCCACAGCCCTCTCTGCTTTGCTACAAATACTCCACAGCCCATGGTCACCATCACCACCGAACAGCACCTCATGTCAGCCACTGATGGTGCTTTCCCAAGCTGCATTTCAGATCTGCAGACACCACCTCAGCTCAGAGAACTGATGACTCTGAACTTCCCTTGACATCACCTTCTGCCCCAAGAAGCTCTGGCAGCGCAGGCAGGTTGGTACAGAGCTCAGTACACGATACCTCCATCTGCAGCAGAGGCCATGCCCTTTCGCTCTTATTTTTGCAGACAAGCACAAGGTTAAGCTTTTGGTGAGCATCTGCTCATGCAGCACTGTGCCTTAACTCAGACGCATGAGCGATGACACAGAGTTACTTTGGGCTCAGCTTTGTTCCCATGCCTTGAAGGAATGCCCTGCACATGAAGACCTTCAAAACCAACAGCAAAGCTCTTCCAATCCGATTCTGGAATCCATTAACAGTTGAAAT

Novel00500

AGGTGGCAAATGGAGGACTACAGAGACACTTGACAAGGCAGTTTTCATAATTAAGAAAGCAAAATGTCAGCAGCCAGCCTGGGTGGGCCATACCAGGTGCCAGACATGGGCCAGAGGGATCCCAGCAAAGGCCCTGCCTTGAAAATGGAGCCTGGAGGATGGACAAGGCCACAAGGAAAGTGGCAGTGTTGTCTGCAGCAGCGTTAAGGCTTTTAGCAATGGCAAATCCTTTTTTTTTTCAGATGATATGCACTTAGTAGCAGTATAGCCAACTAGCTCAGATTTGATAGCTTACAGAGCTAAGAGTTTGTTAACATTTCATTGATAAAGGATAAGAAGGGAAAATTGTGTTCCCAGAAATTAAGTAAATGGGAGGCTAGGACCTTGAAGAAAGGTAAACAGGGGAGAAAAGAATGAGAGGGAGAAGTGTGGCCTTCTCTGCGAAGATATAAGGCCATTGCTGCTTCTGTACCTGACTCAAATACCCAGCTCAACCATTGGCAAAAGAAGTGGAAAGCTTCACCAAGGACTTCAGGGAACGAGATAACTGGTGCTGGGGAGCCATAGAGAGAGCTGGGAATCAGATTCTCTGTACTGGAAGATGAAGGGGAAGATGCTTCAAAACTAGACCCTCCTTGCATATGCGAAAGGACATATGCCACGCTGGCTGGCTGATGCTGCAGAGAGAAGGGAATGAAGGTGGGGGAAAATTAGAGTCCTTTGAGTCTGCTTGTTTCCTTCCAACCCTCGGAGAGTGTGTTTATTCTGAATTATGGATCATATTTTTTGTGAACCATGGAACTCTGGAGTTACACTGTCTGTAGTTACACTACCTGCTTGCTGTGGTGGTTCATCTGTCAGGATGGAAAAGGTGAACCATACATTCAGAAAGAATGTTTTAATTATATTCCTGGTGTGAACTGTGGGCCAAATTTCAAATCTAGAGGGAGAGAGGTCAGCTGTGTTTGTAGGTCCAAAGATTACGTGCAAGAAATCAAGCATTTAGACAAACAACCAAGAGATCAGGAATTCATGGATTTGTTGCCACACAGTTATTTATTCAGCTAAGATTTTCTTGCATAACTTCAAGTAGTGTTATGAATACTAGAGTTTCCTGGCATGTACCCACAGAAGTAAGTATTAATGTGTGATTAATTCTTAAGTTTAGTATAGCGATACCTCTCAAGTAGTTATTTGTTTTTAAAAGAAAAAAAAAATTACATTTAACTGGGTGGGAAACCAAATCTTTCTATTACCCGGGCCTTGTTCCCCAGCCTTGTGACAAGCTCCCAAGTTAATACATTTTAGTCACCACAGGCATATTTTGTCAAAACTTCTGGAGAATGCTGACCCAGGATAAGCCGAAGAAGAAAAACCGACGTAAGGCATTGAACTAATTGTGCAATGCTTTATGTGGAAAAGTGACTTCCTGATGCCAGCGTTAGTAGGTGGTTCTTTCAAGCATGGTGTTTAGCGATCTACATTTGGTTCTTCCAGATATAAATATTTTGCATATAAATATAACTTGTTTTATCCACGTATATTAACTATTGTATTCAATGCCACTTCTCTCTTTTTACCTAATATTTTTCAACACAGATGATGCTCATATATACTTTTAGATTTCTTATTGCTTAAAATGTCATCTACCAGCTCATAGGTATTTACTTTGTGAACTCTTGTTCTAGAGAACCTAAGTAGTGCCCATGTAGCTGGAAAGTTGTGCAATGAGCTTCTAACGTAGGGAGCTTAGTGAGCTGACTGAAATGCAAGCTGTAAACAGACGGATGTGTAAAAATAGCTCTAAGTCCATAAGTTTGGGAACATTTTTGCAGCTTCAAGAATCTTAGATTTGATAAGCAACATAAACTATCCATTGCATATTTGATTATTTCTGAATAGGTAAAGCTAAAATCAAGTTTACCTCAGAGTGTTCTGTCATAACTCTAGGAAAATGGCATTTGATGCAAACTTGTGTTAACAAAAACAAACCACAAAATCCATTTTTCTAGAGTAGGGTGAATGACCGCTCTTACATCAAAAGTACATATATATATTGGAGCCAAAGATGTTTCCCATGATGGAAACATCCTGGAGCCTTGCAGGCAGGTAGACCCTGCCCTCCTTCTGAAGGAACTCAGAGGAGGTTTGGAAAAGATGCAAAGATCTTTGGGCACAAACATCCCTGTGGGTTCAACACACTTGATTTTCCAGAGGAGTGAATTTTGTAACTCCAGAGGCTAGTGGGGGGCTTCAGAGGGATTTTATTATGCCCCCTGTTTGTACTCCTATTTAAGACCACCTATTATTTCAGTTAGCTTGCAAACTAATAATGCTGCTAAGTTAAGCATCAATCTAATTCCATCATCTTTATAACTTTATTTTTCTGGAGGCAAGAAAAGAGATAAATACAGTGAATGAAGTGTATGGATGTCCTACAGAATGGATCTGTATCTGTTACAAGACATTAACCATGACAAACTACAGATTATCCAATTCTTGTTATTTTAGGTCAACAACAAAATGTGACTTTGCCAAATGCAGATTCAGTTCCTGGGGTGTTCAATACAGTGACACATATCACGGCATTTCAGAATTTGCTGTGTACAGAAGCACCGCAAAAAGTTTTTTCACTTAGGTAGAGAATAACAGAAAAAACCCAAATGGGAAGTTTTTCTATACTGTCTTGGATGACAAGGTTAAGCAGAGAATCTGTTAACACACACACACACACACACACAAAAAAAAAAAACAGTGACAATTTAAATAAATCAGATGTTTTTGCTGAAGATTATGCTGTCATTTTCTCCAATTATTCTTGACATTTCACCCAATTGTATTGTGATCTGAGTTAATAATGGAAGAAAAAGAACGGCTCTCGTTGCCTTGTTTATTCTTTGTTCTCAAGGAGATAAGCTGTTTATAAGGATATTTGTTATTTAAATCACTTTTACTGAAGTTGTTTGTTTCCTTGTTTGAGATTCTAAAAATATTGCAACAATTGTGATGCATCTTTGAAACGCCTATTCACTTCACTATGATTTGTACTGAAATTCATTCCTAAATGGGCACAGGTGTTGATAGCTAGAATGAGTCAAAACCAATAAGTGCTCAATAAAGG

Novel00174

AGCGATTTTTTTTTTGTTATTATTATTTTTTTTTCCTGCAGACTATTTTCTTTTCATGAAAAACATGAAATGGTCTGAGAGATAAGGAGCGAATTTATAGCAGTTCACACCGAAGCAGCCAAGATGCTGGCAAGGAAAAATGCCTTTCCCCGCTGCCCGCCTCCTCTGCACGGTCCCCACCCGACAGCTTCCTCCCATTCTCTCCCAATTCTTGTGGCTCTCACGGCCGCCCCACTTCCAGCTCTTCCTGCCCTACGCTGGGATTGCAGTGGGGATGCTCGTCAGGCCCACGCAGGTCCCCGCAGCTTTGCTCCATGGTTGTGCAGCCTGGATCCATCCATTGCCTTGGGAAGCATCCCCGTGCAAAATCCGTCCGCTGCTAAAAACGAGTCTGAAAGGAGCTTTGGGGAAATCCTTTGGGGCATTTGGGTTGGCGAGGAGTGAACACGGTCCCTGGGAGGCACAGCCCCCCCATCCCCACCCCAAGAAAGCCCCCTTGTTTGGCTCCTGCCCGGCACGCCGGCCCTGCAGAGCTACAGTGCCGAACTCACCCAAACCTGCCATAAATTTCCCCAGCTGCCCCCAGCCCGCCGGCCCCTTACTGCAGCTTTACGAGCTCTGAAGAGCCATAAAGGCCCTTTACTGGCTGCGTTGGTCTTTTTTTTTATTTCAAATTATAGTGAGCCTGGATCAACACCGCACAAAGTACCAATTTACCGTGGGCTGGCGTCCCGTGGGCTCACACCTCCTATAGGGGAAGAGGGAGCGATGCGCCATGCCGGGCTCTGCTGATGTCACCCCAATTCATGGGCGTGGGGTTCCACGGAGGGAGATGGAGGGGGAAACAGCACAAGGGCACCCACCAGGGGTGTCTTCCAGCCCTGCTGCTCTTGGGGATGGTTGTGTGCCTTGGGGCGTGGGGTGGGATGGGGCACATGAGTCACAGCGCCGCAGGATTGGGTGTCCCCACTGTGCCTCCAAGGCAGGAGGGCAATGGAGGGGCTGCTGGGGGGTCCTCCCACCCCCCCCCCCCTTGTTCTCATCCCTCCTTGCAGTGTGTCTCTAAAATGGGGAGCTGCAGTTAGATTCCCCTCCCTGGAGGAAGGGATGTGGGGGACAGGGGCCGCCTCCAGCTGTGCCGCGCTCCCCGCTGCCGCCAGCCCTGGAAGCACATAGGTGGTTAATGCCTTCAGCATGTATGAAATATCCCATATGCCGGGAAACAAAAGGGGCTCTGTGCTATTAAAGTTTCATTTCCCCGCTCCCCTCCTCCCGCCCCTCTTCCCCCTTTTCTCCCTGCCCTCCGCGGCGGCTGGAGCAGAGCGGGAGAGGAGAAGCGAGCCGAGCTGCTGGCAGGGACCGGGTATGGCCACAGTGGGGACACAGGTATGGGCACAGTGGGGGGAGCACGGCCACGGCCCCCGAGTGGGTGGCCATAGGGCTGCAGGGGGGAGTCTGGGGGGTTTGCTTCTCAGAGGGAGAATGGGGATGGTGGGGACGGGGAAGAGATGAGCGTGGTGAGCCTGGGTGTCACCAGGCTGCCCCCATGGGATGGAGGTACCGTGGGGTACCGAAGGGTGGGAGAGACTGGTGGGGGGAAGCAGGAAGAGCAGCACTGGGGTTGCTGCAGGCGGCAGAGCCACCCACGGCAGCAGTGGTGTCCCTGTCTCTGTCCCCATCCCATCCCTGTCCCTGTCCCTATTTCTGTCCCAGTGTCCCCATCCTTGCCCCCACTCCCGTCCCCATCCCCACCCACAGTCCCCAGGGCTATGCAGGAGCTGCGCAGCATTTGGCTGTGCGGCCCCACCGCACCAATCCCGCAGGATCCTGAAGGGGTTAAGAGAGAAAAGCAGAGAAAGAAAACAGAGCACGGGGAGAGCCGGGCTGGGCCAGGGGCTGCCCTGCTGCGGGACGTGTCCTCACAGGGTTCCTTTCCACTGCACGGCGGTTTTGCCAGGACGAGGGCTGATGCTGAACACACACACACACACACACACACACACACACACACACACACACACACACGCTGCTTTATGTCTCACATACGTGCGTGGCCCCAACCCCCAAACGCTCCCGGCTGTGCTCACTGCCACTCACCTCGGTGGCACCTTGGTCTCACCTTGGTGGCACAGCAGCTCTGCTGGTGGCACCCAAAGTCTCCACCAAACCTGGTGCTTTCTGCCCCCAAATTGCCCCGAGAATGCCCCCTGACAGCTGGCGTGGGGCTGTCCTCACCTCCAGGAAGCATGGGGGACAAAGTGAGGCTGGGATCTGCCCTCACAGGGTCCGGAGGGAAGCAATGTATAGGACGGGCTGCCACCCGCCCAGTGCTGAGCAGGTCCTGCCCTGCCCACAGCCATGGACACAGTGCTGTGAGTGGCACCAGCAGCTCTGGTGGCCCTGCTGAGGAGGACGGAGGGCAGAGGGACCCTCCCTGGAGCATCCCCCAGCCCCGATGCCCACACATCCCCATGCAGCCCCAGCAATGTTTGGGGACGCTGCAGTGGGGTCCTGGGGTGGATGGAATGGGGCTGGCAACGAGGGACGGCAGGGAGCCCGGGGTGGTATTGGGTTGCAGCAGAGGTGACAGCAGCCGGGCACAGGGCGGTCCAGGCACGGCTCCAAGTGACAGCCACACCATGGGGACATCTCCGTATCAGTTGTGGAGGCAGTGTTCCCTTAAAGAAATAGGGTCCAATAGGGTCCTGCAGGGGTTTGCCCTTTGGGGACGCATCCCATCCCGGCCTTCTGCTGCACTGGGATGGTGCCAAAGGGTCCCTGAGAAATGCCCCATTACAGCATAAAGCAGCAGGTTTGGGGGCTCTCTGCTTCATGGTTCACACCAACAGTGCAGGAACGGTGTAGGAGCCCCTGCCCAGGAACTGCGGTGTTGGAGAGGAGATGCTGTGCCAAGCTGGCCCCGTGGGACATTCAGCTGCTGGCATAGGGAAGGTGCAAAAATCCCCCGATGTATGGGTTGCACTCGGTCTATTTTGGAAAGGGAAGGCGCCGATCTTGTGACCGGAGCTGGGGACTGAAAGTCTGGGCAGCAGGGTCCTGCCTGACTGACAGCGACCCGCTGCGTGACCCGGGGAAACCCGAGAGCCTTCACACCACAGCCCCAATCCCACCCCCCTGGGCTTGTTGAGAGATGATGGAAACCGAGTGGCGATGGGGACACGGGGACAGCATCCCCGAGGCAGGGGAGCCCCGATGGGATGCTGCTTCTGCCCACACACCTGGGCTGGGATATGGGCAGGATGGGTCCCCCGTGTCCCCCATGTCCCCTTGTTGGGTTTGGGTTGCTCAGAGCTGGGAGGAGGGGCTCTCTGCAGTGCTGGCAGCCTCAGTGCTGGCAGCGCCTGCCAAAAATCTGTGTGTGGGAGGTGGAAAGTTAACTTTAGTTTTCCTTTTTGGATGGGGAGGGGCGGCAGGTTGTGAGGAAGGCAGAGGGGGGTGCACGTCAGACCCCATCGAGTGGGCGGGGAGGGAGCAAGAGGTGTTCCTGCCGCCAGATTTGGTGAACGTAGGGATATTTCCATCTATTTTTCACTCTTCCCTCCCCCGGCACCACTTGCAGTTCATGCATTAGGAGCAGCAGAGGCTCCTCTTGCAGGGTCTTGCTGCAGGAGGGGCTTCAAAGCCACACTGCACTCCGAGCAGCCCACCCTCCTGCAGCCTCTGTGCCCATAGGGCTGGGAGATGGGTGCTCCCATCACACCCCGGCACCCTTTGCTCCAGCACAGCAGCAGTAACGCACGGTGCCCTGCAGAGCCCAGTACAGCCCCCGTGGGCGGGTGGGGCTCAGTGTGGGTGGTGGGAGCTGCCCACCTGGGATCCCTCTGCAAATCCACATTGAACGGTGGTGGGAGGAGGGATTCCAGAAGAGCAAAGCGCGGGGTACAATCCTGAGCTCAACAGAGATGGCGCTGAGCTGTCTCTGCCAGCATCACCTAAGTCCAGGCGATGGGGTGGGCTTTGGGTCATGCAGAACCCTTCTGGGCTCAGCACGCAGGAATTTCCCCGCTGTGCTGAGCAGCATCCTGTCCCCACGCGCTGATCCCTGCTCCCAGCCCGCACCTTTCCTCTTGGCTGCGGTGCTGTGTGCGCTCATCACTCAGCAGCATTTCTGCTCAGCAGAACAGAGGGAAGTGAGTGTGTTGGTGCTTGTCCCAGAGCCGTGAGTCATGCAATGGTTAAACACGGCCCGGCTTTGTGAGCTGGGTGCAGGATGGAGGTGGGAGCTGGGGCTCCTGCCTTGAGCAAGCGCCAGAGTGAGCCGAACACAGCCCCCAGTGCCGTGGGTGGGGGTGGAGAGGTGAGGTGCCCCATCCTTCACCTGGAGAACTGCGAGTGGACTGATTGCTCAGTGTTCCCTTGCCCTTTGCAGGTCCTCTGTTGGGCTGGAAAAGAGAAAACCCCGCAGACCTCTCCTTGGTGACTCTGAAGACACGGAGCGAGGGGCTGAGGAGTGAGGACTGTGCCCTGCTTCCTCCTCGGATGGAAGGGGACACTCCCAGAGCAGGGCCAGCCCTCGGGCCGTGAGCGGCCAGTTGGATCTTTGGAGTCACTGAGCATCGTCCCCGAAGGTGGAGAAAGGAAGCCGGCAGGATGCTGAAGGCACCGCTGCGACAGGAGAGCGACAGAGGGGACATCCCAGACCCTGAGCTCCCCACAGGGAACGTGAACACTGAGCCAGCAGCCCCCCCGGATTGCTGTGCCCCCAGCCCTGCCCCTCAGCTGCCCCCAACAGCAGTCAGCCCTATGGATGACCCCAGCCAGGAGTGGAGGTTGGTGAAGATCAAGCGCGTCCTCATCGCTCCCTCCACTGAGCCCAGGAAAGCAAGTAAGGACCCCCTTATCCCCCCGCAAAGCACCTCAGCACCATGGGGCTGCAGCATCCCCAGCACACGGTGTGGTTGGGGTTCCTCCCATGCAGGGCGGTGGGTGCCGGGTCCCAGCTGTGCTCTGGGGCTTTTTTAGCTCCAGATGATCTCAGTGCCCTCAAAAAGCAGAGGAATCCGTGCCATCCCTGAGGAGGGGAGGGGAGCAGCGGGTGGATTCCCACAGTCTCTGAGAGCTGAGTGGGGCATAGGGGTGCCCCGGGGTCAGGAGCAGACCCTGGGGTGCTGTGTGGGATTTGGGCATCTCTCCCCACCCAGAGCATGGCACAGCTGTGTTGCGTATGGGAGACAAGTCCGCATGGATGCTCCTGCTATGTGGCTTCGGGCCATCTCTATTTCAGGCCCCCATTTAGAAAGTAAGTACTTTATTTCAGGCTGGTTGCTCCCTGCAATGCTCCCTGACACCGCTTTGCTCCGGAGCCCTGTTTCACAGCCTGTCTTCCATGGGGACCCCAAATGGGGTTACAGCAGCCCCAGGGGGAGATGCTGGCCCCCACCTTTCCCTACAGCCATGGGACCCATCTTCAAACCCTACTTCCTCCGCTCTATTTCCCCCCACCAGCTGTTATTTCAGCTACCGGGGCAGGTGTGCCACATCCCTGCTGGGAATGAGCCCTCGGGTGATGCTGTGGAGTGGTTTTCCCAGAGCAGCCCCTTCGCCCTGGGGACTCCAAATGGGCTTTCCCAAACCCATCCATCCCGGGGCACTGCTCGCCCTCACACCAGCAGCCCGCGGGATGGGATGACAGTTTGGAGGTGGGAAGCTCAGCCAAGCTCTTGGCTCAGTGCAGCCATGCAGCGTCGTGTTTCACGGCTGCAGACAGACTCAGAGCATTTCTCCCTCTGTTTGGGATGGTGGATCCGCAGTGGGAAAGGTCAGGCCCAGCTCTGTCCGGGCTCAGCATTTTCCTATCCCGAGCCGCGATCCAGCAGTTCTGCTGCCAGCCTGGAATTTAAAGTAACTCAACAGGTTTGCTGGAAAGCGTTTGTCTGCCCCGTTTCCTCCTCCCCCTGAGTGAGATCACTGCCATCCACTGCCTGCGTGACTGTCAGACTTTCCAGAGCCACAGGGCAGGATGTCACCGAGGGTCCCAAGCCCTGGCAACAGCCCTACAGTAACGCTATGGGTCAGCGGTGCCGGGCTGGCTTTGGAGCTGGGCTGCAAGCCAAGTGTTAGGGCTACCCTTGCAGTGGGGTATTTGCAAGAGCATCCCCAGGAGGTGTTGGGGATCCGCTCCTTTTCTCCTTATCTGTCGATTTCATATGTCGGTGCATCCATCCCTACCCCGATGCCTGCAGGAATGGCACAGGGAGTGCATCGCCGAGCGGTGTGCACACACCCTGCAGGGTCAGCAGCCCCATCCAGCCCCATCTCCTGGCTGCGATCCGGCCCAGCGGCCCCAACGGGTTGGCACCTCTCAGGGAGCCCCGGGTAGAGGCACTGGCAGCGAGGTGCGGGAGGCAGGACTGGTTTCTCTGTCTGGTGTGCAAATAAACAGAAGGTTTCGCTCCCTGGGGGATGCTGAGCTCGCGCCGGGCTCCCTCAAAAACCAGAAAAGCACAGAAAAGCAGAGAGCAGCAGGAAGAAAGGAAATTAGAAAGGCAAAAAATAAGGCCAGGAGCTCATCCGGGGGCGCAGACGGAGGGGGAGCGCAGCAGCGTTGAGGGCTCTGCGTGCTGCTGATGGGTCCCAGCGCTGTCACCGCCGTGCCGGGGGGGCTCAGGATCAGCGTGACCCCACTGTGCTCCTGGCGTGAAGGCAGGGAGGTAAGGAGGTGCTCGGGGCTGTGTTAGGGGGTGCTCAGAGATGAGTAAAGGGATGCTCGGGCCGCTCTCGCGGTTGCTCAGAGCAGACGCAGCCAGGCTGGGACGAGCAGAGGGCAGAGCCGTCGGAGCTGCCCTCACCACCGGGATGGCGGCCCCGTCCCGTCGCATCTCGCGCGGCGCGGTGCGATCCGCCGTACCGGGGCGGGCACGGGAACGAGCAGCGGCACCCCCAGCGGCCCCGGCCCCCCGGCCCCCG
